# European ash pangenome reveals widespread structural variation and genetic basis of low ash dieback susceptibility

**DOI:** 10.1038/s41467-026-77194-9

**Published:** 2026-09-17

**Authors:** Daniel P. Wood, Mohammad Vatanparast, Dario Galanti, Catherine Gudgeon, Katherine Wheeler, Emma Curran, Levi Yant, Richard Whittet, Richard A. Nichols, Richard J. A. Buggs, Laura J. Kelly

**Affiliations:** 1 Royal Botanic Gardens, Kew, Richmond, UK; 2 Centre for Novel Agricultural Products, University of York, York, UK; 3 School of Life Sciences, University of Nottingham, Nottingham, UK; 4 School of Biosciences, University of Sheffield, Sheffield, UK; 5 Linnean Centre for Plant Biology, Department of Plant Biology, Uppsala BioCenter, Swedish University of Agricultural Sciences, Uppsala, Sweden; 6 Northern Research Station, Forest Research, Roslin, UK; 7 Centre for Evolutionary and Functional Genomics, School of Biological and Behavioural Sciences, Queen Mary University of London, London, UK; 8 Centre for Biodiversity and Sustainability, School of Biological and Behavioural Sciences, Queen Mary University of London, London, UK

**Keywords:** Plant genetics, Plant genetics, Genetic variation, Biotic

## Abstract

European Ash (*Fraxinus excelsior*) is a keystone tree species, whose populations are being decimated by ash dieback disease (ADB) – better characterisation of genetic variants associated with low susceptibility to the disease is needed. Here, we develop a *F. excelsior* pangenome to more fully capture sequence variability within this species compared with a linear reference genome, using a geographically diverse set of fifty *F. excelsior* samples. We identify 362,965 structural variants (SVs), including 174 Mb of sequence absent from the linear reference genome (22% of the linear reference size), and identify 3,412 high-confidence dispensable genes (those present only in some individuals). We use the pangenome to analyse existing genomic data from over 1,200 individuals, revealing 220 single nucleotide polymorphisms (SNPs) showing consistent allele frequency shifts between healthy individuals and those highly damaged by ADB, across UK seed sources, explicitly demonstrating the existence of a shared genetic component to low ADB susceptibility.

## Abstract (web summary)

European ash (*Fraxinus excelsior*) is a keystone tree species throughout temperate Europe whose future existence is threatened by ash dieback disease (ADB). Here, the authors report the construction of its pangenome, revealing structural variation and the genetic basis of low ADB susceptibility.

## Introduction

High-quality reference genomes constructed from single individuals have been used to great effect to assess genetic diversity within species, allowing mapping of short reads from many individuals and discovery of genetic variants^1^. However, this approach provides limited insights into larger structural variants (SVs) between individuals and can lead to some miscalling of single nucleotide polymorphisms (SNPs)^2^. Pangenomes—reference genome data structures incorporating highly contiguous sequences from a representative set of individuals—have revealed the total sequence contained in SVs can exceed the average genome size of a species^3–6^. These SVs frequently encompass genes, with some studies identifying >50% of genes within a species as dispensable (i.e. not present in all individuals^5,7–9^). Pangenomes thus often contain genes that would be entirely missed if using a single reference genome approach^10,11^. Indeed, novel disease resistance genes are frequently identified in pangenomes^12–14^, including in trees^15–17^, suggesting pangenome approaches may be particularly valuable when seeking to detect loci contributing to pathogen resistance. Analysis of existing data using pangenome references has uncovered the basis of missing heritability in some systems^18^ and has enhanced our understanding of the genetic basis of a variety of traits, often adding complementary information to that uncovered by analyses using a linear reference genome^5,19–21^. Thus, to better understand genetic variability within species and its impact on traits, pangenome references incorporating diverse samples are required^10,22^.

*Fraxinus excelsior* (European ash), a keystone tree species within Europe, is under severe threat from ash dieback disease (ADB) caused by the invasive non-native fungus *Hymenoscyphus fraxineus*^23,24^. Populations of ash are undergoing substantial and rapid mortality^25–27^, with high associated management costs and biodiversity loss^28–30^, attracting substantial public concern^31–34^. A small fraction of trees (as low as 0.5%^35^) remain healthy under *H. fraxineus* inoculum pressure, with quantitative resistance to ADB having a narrow-sense heritability of approximately 0.4^35–39^. Understanding the genetic basis of this trait is important for ascertaining the feasibility of producing new individuals with lower susceptibility^40–44^. Stocks et al.^42^ identified 192 SNPs significantly associated with low ADB susceptibility when using a *p*-value threshold of 1 × 10^−^^13^ in mainly UK populations by mapping short reads to a single reference genome. Studies in other European populations did not find SNPs strongly associated with low ADB susceptibility^43,44^, with one study indicating many of the most significant SNPs from Stocks et al.^42^ were at very low frequencies in Austrian populations^45^, raising the possibility of diverse mechanisms of low susceptibility between populations in different parts of the species’ range^42–47^. A pangenome reference for *F. excelsior* that captures variation from different genetic backgrounds would represent an invaluable resource to enable improved understanding of this critically important trait. A recently published superpangenome of the *Fraxinus* genus identified interspecific SVs associated with salt tolerance, suggesting a role for these variants in adaptation to abiotic stressors over millions of years^48^. However, this study did not capture intraspecific SVs in *F. excelsior* that may contribute to low ADB susceptibility.

Here, we construct a chromosome-level, phased genome assembly for European ash and a graph pangenome using long-read sequencing from a diverse sample of 50 individuals across Europe. This reveals 174 Mb of additional sequence captured in SVs, a size increase of 22% compared to the new linear reference genome from a single individual. By explicitly associating putative dispensable genes with SVs, we identify 3412 high-confidence dispensable genes in *F. excelsior*, including 141 with Gene Ontology terms associated with defence response. Reanalysis of poolseq data (i.e. sequence data from a mixture of approximately equal amounts of DNA from multiple individuals) from over 1200 individuals^42^ reveals 220 SNPs with broadly consistent allele frequency shifts between healthy and unhealthy pairs of pools across a set of mainly UK seed sources, providing explicit evidence for a shared genetic component to low ADB susceptibility.

## Results

### A high-quality linear reference genome for European ash

A phased linear reference genome was produced for a single *F. excelsior* individual, with total sizes of 793 and 787 Mb, N50s of 32.9 and 32.2 Mb, and L90s of 22 for the haplotype 1 (henceforth referred to as BATG-1.0) and haplotype 2 assemblies, respectively (Supplementary Data 1). *Fraxinus excelsior* has 23 pairs of chromosomes^49^—the top 23 scaffolds of BATG-1.0 and haplotype 2 contain 93.7% and 94.5% of the total sequence in each assembly, with the 24th scaffold for each of the haplotypes being 2.1% and 1.5% of the size of the 23rd scaffold. Three short scaffolds were identified as potential contaminants (with lengths between 14,626 and 35,091 bp), representing 0.009% of the total assembly size—these were removed from downstream analysis. Telomeres were detected at both ends of 19 of the top 23 scaffolds in BATG-1.0 and 17 of the top 23 in haplotype 2. Together, these results indicate the assemblies are pseudochromosomal. Unlike other recently published pseudochromosomal assemblies of *F. excelsior*^48,50^, this assembly is not scaffolded using the *F. pennsylvanica* genome assembly^51^. We also assembled and annotated the *F. excelsior* chloroplast genome; this was 155.6 kb in length with 130 annotated genes.

The BATG-1.0 and haplotype 2 genomes had 98.2% and 98.3% of eudicot BUSCOs completely assembled; 21% of BUSCOs were duplicated, a similar proportion as in other *F. excelsior* assemblies (Supplementary Data 1) and consistent with the fact that there are several whole genome duplication events in the Oleaceae, the family to which *F. excelsior* belongs^48,52^. Ninety-eight point three percent of PacBio HiFi reads mapped to one or both of the haploid assemblies. The total assembly sizes of 787 and 793 Mb are smaller than the 1C genome sizes of 877 and 840 Mb estimated by flow cytometry in Sollars et al.^53^ and Siljak-Yakovlev et al.^54^, respectively—different estimates could result from methodological variation or inter-individual genome size differences. Analysing 27 bp kmers from the PacBio reads using GenomeScope^55^ gave an estimated genome size range of 823–825.4 Mb for this individual (Supplementary Fig. 1)—other kmer lengths tested (see 'Methods') gave very similar size estimates. The coverage of read mapping to both phased assemblies did not show clear peaks of higher depth beyond the main homozygous peak, which would be indicative of collapsed sequences (Supplementary Fig. 2). This suggests the remaining difference between estimated genome sizes and assembly length may be due to highly repetitive regions such as centromeres and ribosomal DNA that are challenging to assemble. The assembly sizes are similar to the size of unpublished pseudochromosomal assemblies of *F. excelsior* that do not use *F. pennsylvanica* for scaffolding (i.e. FRAX_001_PL and daFraExce3.hap1.1; see Supplementary Data 1). The long terminal repeat (LTR) assembly index (LAI) was 14.85 and 14.62 for BATG-1.0 and haplotype 2, respectively, with 5.35–5.44 intact LTRs/Mb (comparable to other recent *Fraxinus* assemblies), with the top 23 scaffolds in each having average LAIs of 20.74 and 20.62, respectively (Supplementary Table 1)—a score of >10 indicates a reference standard genome^56^.

Repeats constitute 65.6% of BATG-1.0. LTR elements occupy 33.0% of the sequence, most of which comprises Ty1/Copia (15.3% of genome sequence) or Gypsy/DIRS1 (15.9% of genome sequence) families, with DNA transposons covering 6.2% of the sequence. Unclassified repeats covered 19.9% of the sequence. The total repetitive fraction is similar to the estimated repeat content of the assembly of another *F. excelsior* individual (69.12%) and assemblies for some other *Fraxinus* species such as *F. pennsylvanica* from the *Fraxinus* superpangenome^48^, but is substantially higher than a separate estimate for *F. pennsylvanica* (48.4%^51^). Olive, also a member of the Oleaceae, has a lower estimated repeat content than *F. excelsior* (56.2%)^57^, although tomato (a fellow asterid with a similar 1C genome size) is more similar (65–72%)^58,59^.

Annotation of BATG-1.0 was performed using BRAKER3^60^. Ten runs of BRAKER3 were performed with short-read RNA-seq data and protein orthologue information, and 10 runs with long-read RNA-seq data and protein orthologues. These identified 31,926 genes annotated in every run, with a further 1736 genes annotated in every run of the short-read annotation only and 1246 genes annotated in every run of the long-read annotation only. The median length of genes exclusively identified by long-reads was 43% longer than those identified only by short reads (Supplementary Fig. 3), although different underlying tissue types were used to generate the long-read and short-read RNA-seq data, making these differences challenging to explicitly link to the length of RNA-seq input data. A further 3075 genes were inconsistently annotated in either the short- or long-read runs. To reduce the chances of erroneously identifying genes as dispensable in the pangenome, we initially retained all these genes, giving a total of 37,983. This is a similar number to that reported in the BATG-0.5 assembly (38,949)^53^ and the *F. pennsylvanica* assembly (35,470)^51^ but lower than the 43,392 genes reported in the recent Fexc_ONT_UoY *F. excelsior* assembly^50^ and substantially lower than the *Fraxinus* assemblies used in the *Fraxinus* superpangenome (48,389–53,538)^48^, including their *F. excelsior* assembly (50,327). These latter two *F. excelsior* assemblies have more genes annotated than are plausibly missing from BATG-1.0 due to being dispensable (see pangenome annotation below), suggesting differences are likely due to different annotation approaches rather than intraspecific variation in gene content. Using OrthoFinder^61^ to identify orthologues from BATG-1.0 and available annotations for *F. excelsior* assemblies (BATG-0.5, Fexc_ONT_UoY) as well as *F. pennsylvanica*, *Olea europaea*, *Solanum lycopersicum*, *Erythranthe guttata*, *Sesamum indicum* and *Salvia miltiorrhiza* (Supplementary Data 2), 0.6% of BATG-1.0 genes lacked an orthologue in another assembly, compared with 1.1% in BATG-0.5 and 7.3% in Fexc_ONT_UoY. The total BATG-1.0 protein set contained 98.7% of eudicot BUSCO proteins, suggesting the annotation captures the vast majority of genes in the assembly.

### Structural variant calling and pangenome construction

**Fig. 1:**
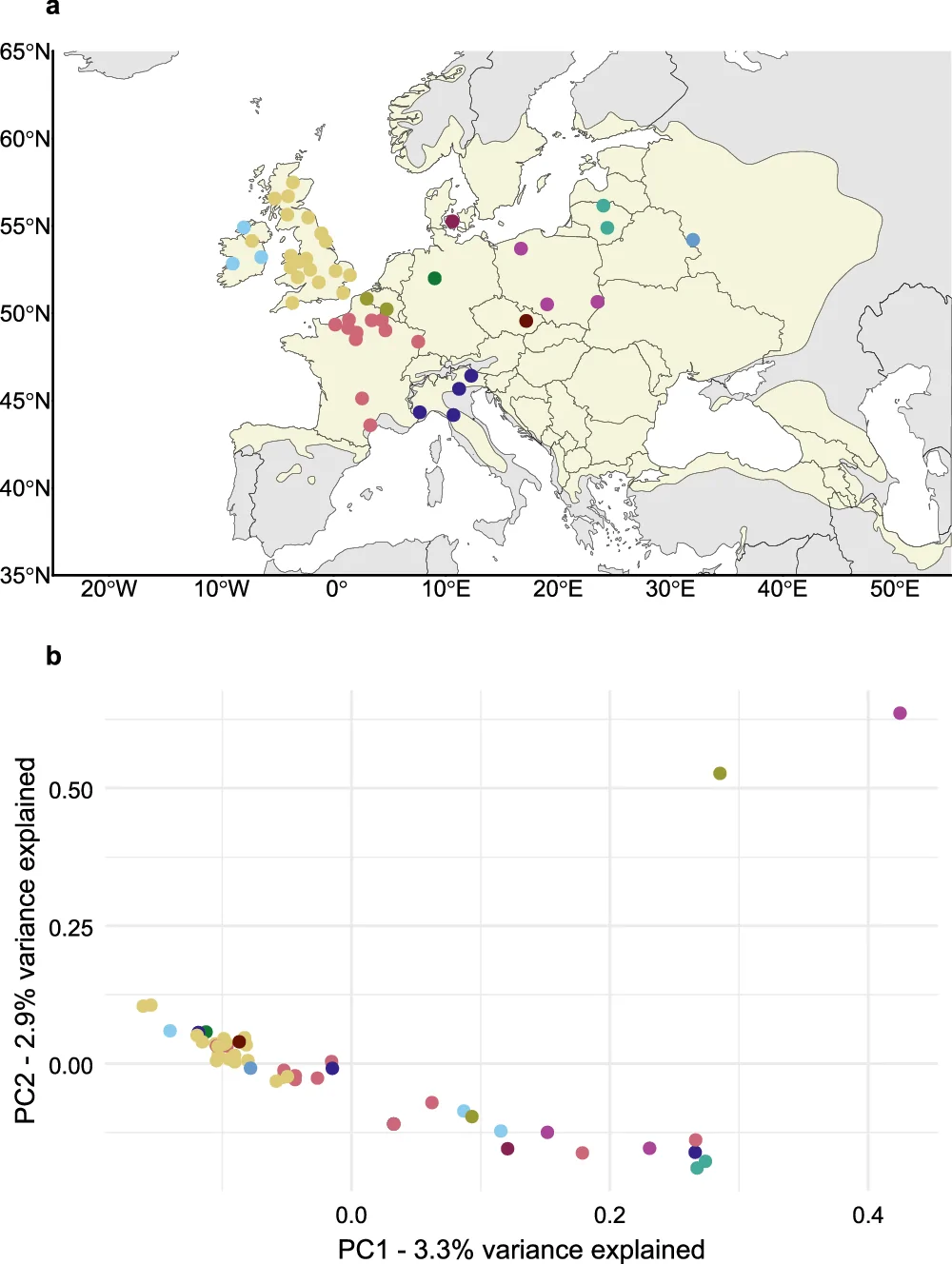
Geographic origins and genetic structure of samples. **a** Map showing location of origin for individuals used to construct the pangenome; see 'Methods' and Supplementary Data 3 for full details. Longitude and latitude are denoted on the X and Y axes, respectively. Points represent individuals sampled or approximate sample locations for seedlots from which individuals were grown. Light yellow areas represent the native range of *F. excelsior* using shapefiles from Caudullo et al.^136^. **b** Principal components analysis of single nucleotide polymorphism (SNP) calls from long-read sequencing data from individuals sampled. The first two principal components are plotted, with the percentage of variation explained by each principal component shown on the axis labels. In **a**, **b**, individuals are coloured by country of origin (see Supplementary Data 3). Source data are provided as a Source Data file.

Low susceptibility to ADB is likely a highly polygenic trait^36,47^, so we prioritised sampling a larger number of individuals to construct the pangenome instead of focusing on generating fewer highly contiguous assemblies, even if this restricts the types of SV that can be analysed. Oxford Nanopore Technologies (ONT) data was generated for 49 individuals from a variety of European provenances, as well as for the reference individual (Fig. 1a and Supplementary Data 3). Principal components analysis (PCA) of SNPs called from the 49 individuals revealed little geographic structure (Fig. 1b), as was also found to be the case when a similar set of provenances were analysed in earlier work^53^.

Heterozygous structural variants called between the two higher-quality haploid genomes of the reference individual were used to assess the accuracy of calling SVs using lower-coverage ONT data from the same individual with a variety of methods. These included mapping the ONT reads to the reference haploid genome and calling SVs using Sniffles2^62^ and cuteSV^63^, de novo assembling the ONT reads with Flye^64^, Shasta^65^ or nextDenovo^66^ and calling SVs from contigs mapped against the reference using svim-asm^67^, and the combination of read mapping and assembly mapping approaches (see 'Methods'). This analysis indicated that the read mapping approaches had higher recall of SVs detected using the higher quality haploid genomes, while assembly mapping approaches had higher precision, and a combination of read mapping and mapping of the assembly produced by Shasta gave the highest recall (84.4%) and F1 score (77.9%), with a precision of 72.2% (Supplementary Fig. 4). For insertions, deletions and tandem duplications, this method also gave the highest F1; for inversions, using svim-asm with a Flye de novo assembly gave a higher F1, although the overall precision/recall for inversions and tandem repeats was low (Supplementary Fig. 5). The mapping and/or Shasta assembly based approach was therefore used to call SVs for all 50 samples. Within each sample, a median of 41.6% of SVs called with cuteSV and Sniffles2 were also called with svim-asm, and a median of 72.6% of SVs called with svim-asm were also called with cuteSV and Sniffles2 (Supplementary Fig. 6).

**Fig. 2:**
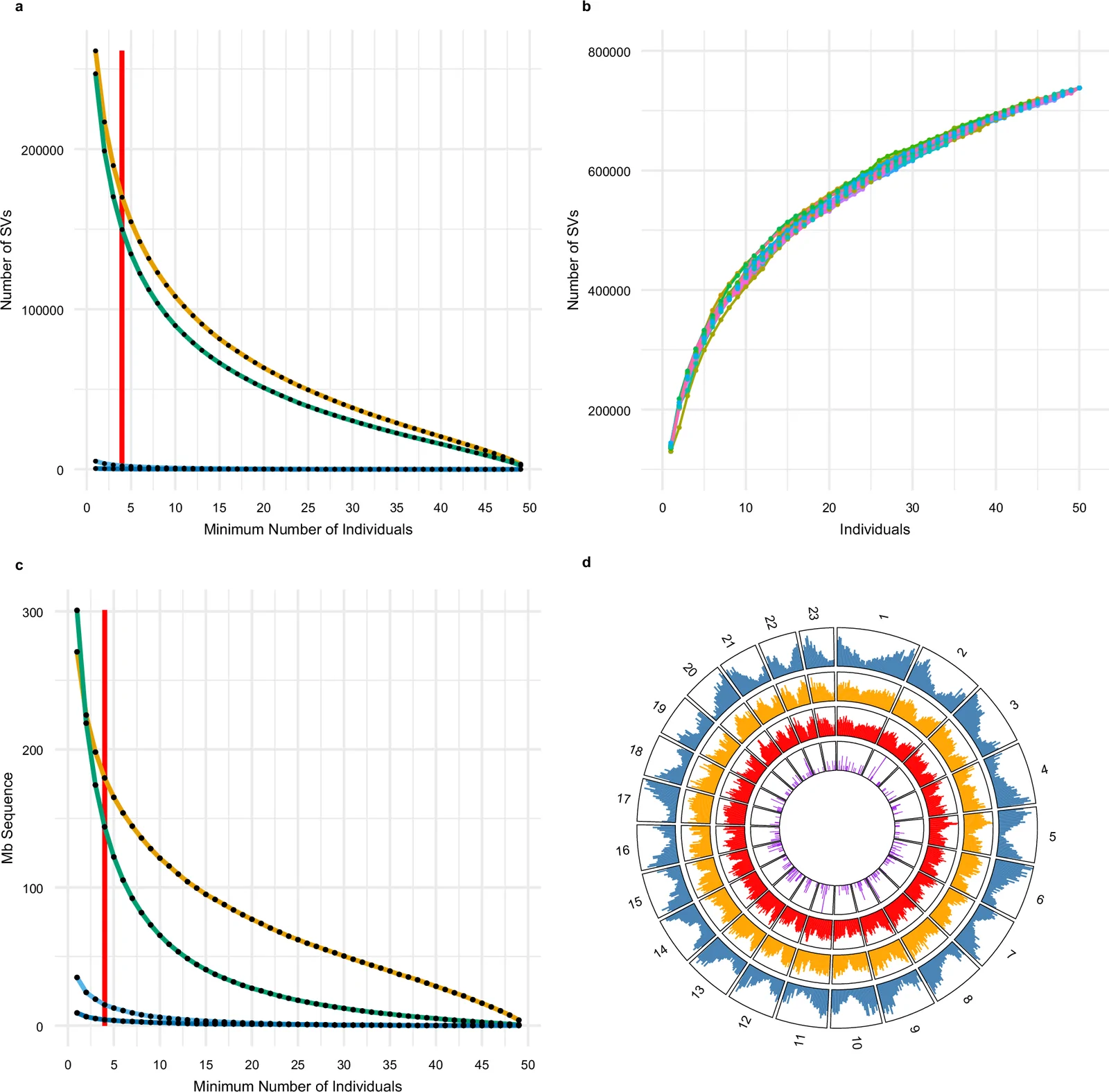
Structural variant properties. The total number of SVs (**a**) and total quantity of sequence (**c**) of different SV classes, at different minimum numbers of individuals called from long-read data. Colour-coding indicates different types of SVs: insertions (green), deletions (orange), tandem duplications (light blue) and inversions (dark blue). A vertical red line at *n* = 4 indicates the cutoff for SVs being included in the pangenome. Note: to aid visualisation, separate plots for inversions and tandem repeats are available in Supplementary Fig. 7. **b** Saturation curves of sequentially merging SVs from different individuals. Each sequential series of SVs was generated by combining individuals in a random order, one at a time, with the total number of SVs in the group being displayed; separate series of random combinations are designated by colour, with points in the same series joined by a line. The X axis represents the number of individuals merged, the Y axis the total number of SVs in those individuals. **d** Circular plot of feature densities per chromosome in 100 kb windows. The 23 largest scaffolds are labelled 1–23 in descending order of size. Tracks, from outer to inner, correspond to density of (i) genes (blue), (ii) insertions (orange), (iii) deletions (red) and (iv) inversions (purple). Larger plots for each individual chromosomes, also including repeat and tandem duplication density, are presented in Supplementary Figs. 30–52. Source data are provided as a Source Data file.

Merging SVs across all individuals identified a total of 737,995 SVs (Fig. 2a). Merging samples sequentially resulted in fewer additional SVs discovered in the fiftieth sample compared with the first, but the number newly detected did not plateau, indicating that more samples would be required to exhaustively sample SVs in *F. excelsior* (Fig. 2b). It is also possible that sample-specific SVs represent sample-specific assembly or mapping errors—to attempt to control for this, only SVs present in more than three individuals were retained for further analysis (Fig. 2a), although this may result in rare SVs being missed. This resulted in 362,965 SVs, of which 170,202 were insertions, 189,724 were deletions, 295 were inversions, and 2744 were tandem duplications (Fig. 2a and Supplementary Fig. 7). The total number of SVs represented 396 Mb of variable sequence across the *F. excelsior* individuals, including 174 Mb of insertion sequences not present in the linear reference genome (Fig. 2c). Ninety-nine point nine percent of SVs were on the 23 pseudochromosomal scaffolds. Insertions and deletions were generally more frequent towards the ends of chromosome arms (Fig. 2d). Sixty-nine percent of SVs overlapped with an annotated repeat in the reference genome; the distribution of base pairs overlapping different SV types closely reflected the makeup of the genome as a whole (i.e. mostly LTRs and DNA transposons), although rRNAs and tRNAs were relatively unlikely to overlap segregating SVs, likely due to their core role in the cell machinery (Supplementary Fig. 8). The set of 362,965 SVs was used to construct a pangenome using vg^68^ which had 30,710,268 nodes and 31,069,469 edges.

### Pangenome annotation: explicitly linking dispensable genes with SVs

**Fig. 3:**
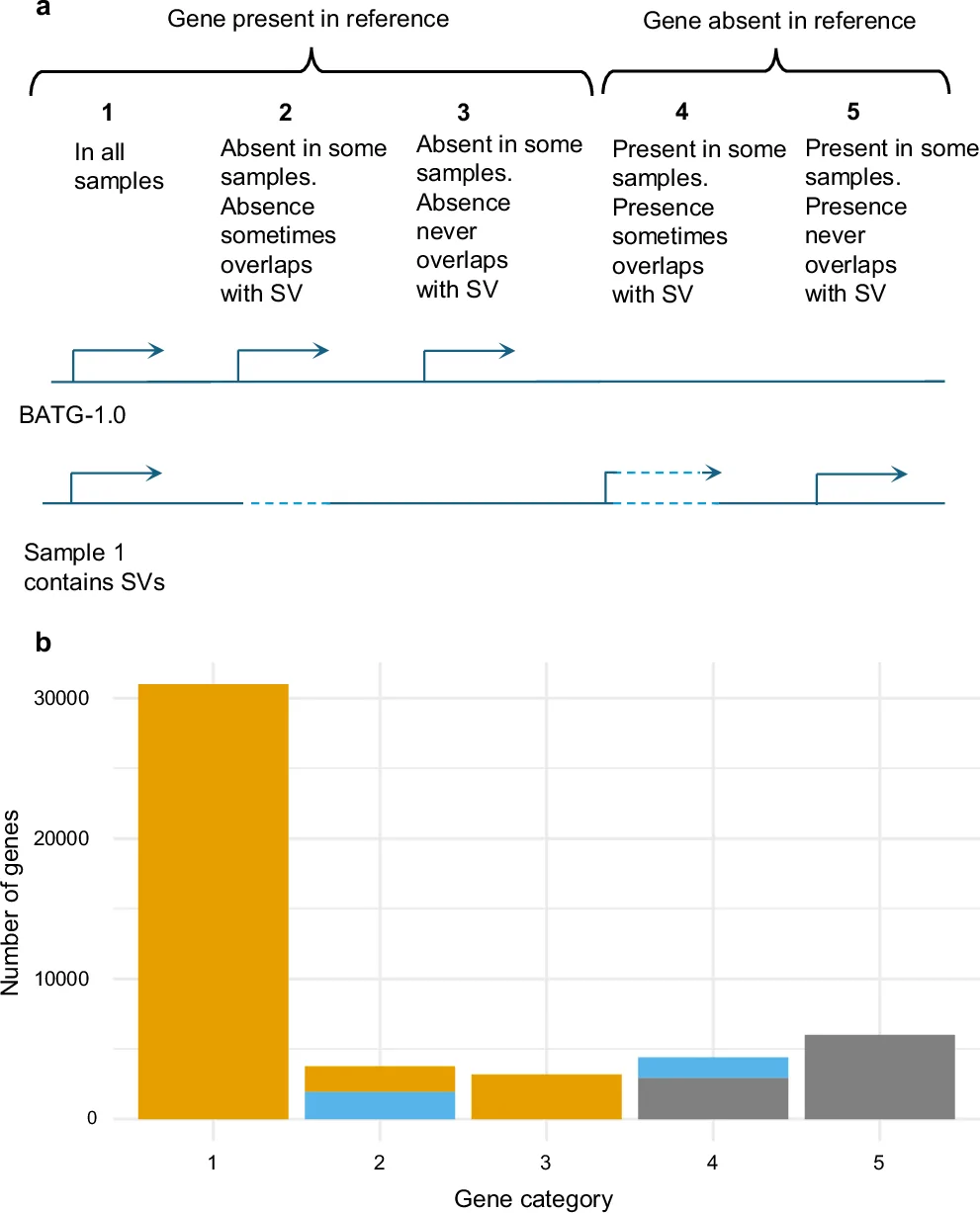
Consistency of association of putatively dispensable genes with structural variants. **a** Illustration of classification of genes in the pangenome based on their presence/absence, and how this corresponds with structural variants (SVs). Genome sequence is depicted as a blue solid line; SVs by light blue dashed lines (representing deleted sequence relative to the reference in this illustration). Genes are depicted by arrows. For illustration, each gene in the reference sequence (top line) is homologous to the gene immediately below them in the example sample (bottom line). For those present in the BATG-1.0 reference, genes can be present in every other sample (1) or sometimes absent (2, 3). The absence of a gene can either coincide with a SV overlapping the gene in at least one sample (2), or with no overlap with a SV (3). For genes absent from the reference but present in at least one sample, this presence can either coincide with an overlapping SV in at least one sample (4), or never coincide with an overlapping SV (5). **b** Barplot of the number of genes in each category in the pangenome, where category numbers correspond to those in (**a**) following reclassification of genes in each category as invariant (orange), dispensable (blue), or discarded from the analysis (grey) according to how consistently they overlap with SVs based on an F1 score. Genes in category 2 with an F1 score >0.8 are classified as dispensable (light blue); those with a score below that threshold are categorised as invariant. Genes in category 3 are never associated with an SV, so are all categorised as invariant. Genes in category 4 with an F1 score >0.8 are classified as dispensable; those with a score below that are discarded from the analysis. Genes in category 5 are never associated with an SV, so are discarded from the analysis. In an approach where genes absent in some individuals are treated as dispensable, all the genes in categories 2–5 would be treated as dispensable genes. Source data are provided as a Source Data file.

To identify dispensable genes with an explicit link to SVs in the pangenome, a genome sequence was generated for each individual by using the SV genotype calls to modify the BATG-1.0 reference FASTA (i.e. deleting deletions, inserting insertion sequence, etc). Genes were de novo annotated for each individual genome sequence and consolidated across individuals using OrthoFinder to identify a set of unique gene models. Of the 48,356 genes, 30,992 (64.1%) were present in all individuals (invariant), including the reference (Fig. 3). Of the 10,411 genes absent from the BATG-1.0 genome (i.e. putatively undiscovered dispensable genes), 6009 did not overlap with a SV in any individual. For those that did overlap with SVs, only 1474 did so with an F1 score (a measure of how consistently a gene presence or absence was associated with a SV, see 'Methods') of 0.8 or above (Fig. 3 and Supplementary Fig. 9). As in each case the non-SV sequence is identical to the reference sequence, these putatively dispensable genes may be the result of stochasticity in the annotation process, or the result of SVs elsewhere in the genome altering the mapping of RNA-seq data and thereby reducing evidence to support the gene annotation. Similarly, of the 6953 genes present in the reference assembly but absent from one or more of the other assemblies, 3190 did not overlap with a SV in any individual. For those that did overlap with a SV, only 1938 did so with an F1 score of 0.8 or above (Fig. 3 and Supplementary Fig. 9). Putatively dispensable genes with an F1 score <0.8 were classified as invariant if present in the reference genome, and if absent were removed from the analysis. This brought the total number of genes in the pangenome to 39,419, of which 3412 were dispensable (8.7% of the total). This is a substantial reduction in dispensable genes compared with the estimated 35.9% of genes without filtering for those consistently overlapping with SVs (Fig. 3). Putatively dispensable genes without a consistent SV association (i.e. those excluded from our final annotation) could be affected by the non-comprehensive RNA-seq dataset (only 72% of individuals in the pangenome were represented in the RNA-seq data) or the particular annotation pipeline used. The final pangenome gene set included 99.0% complete eudicot BUSCOs —slightly more than the 98.7% captured in the BATG-1.0 protein sequences alone.

**Fig. 4:**
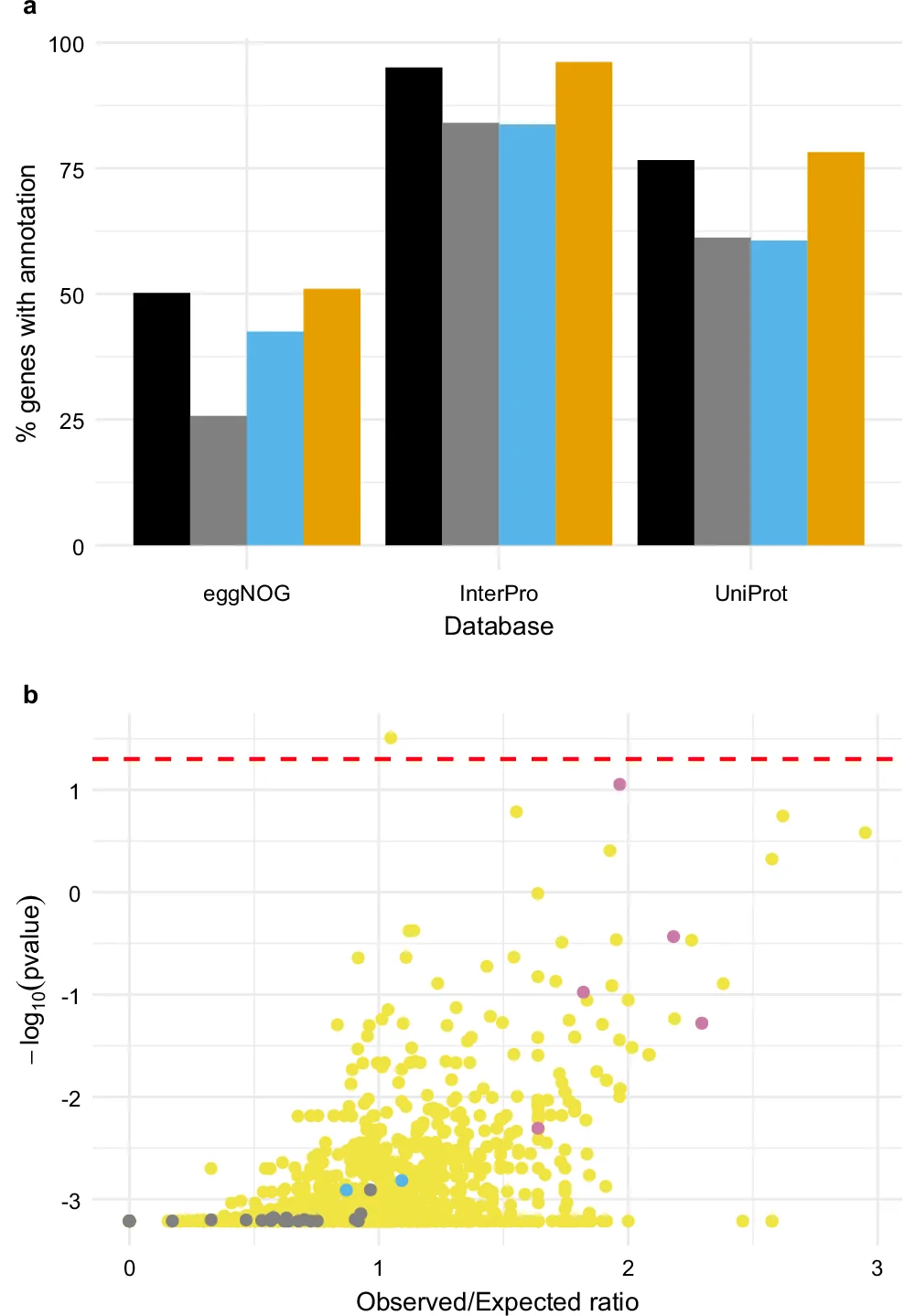
Annotation of putatively dispensable genes. **a** Barplots showing the percentage of genes annotated in the eggNOG, InterPro and UniProt databases, for genes included in the pangenome after filtering (black), putatively dispensable genes removed during filtering (grey), included genes classified as dispensable (i.e. the high-confidence dispensable genes; light blue) and included genes classified as invariant (orange). **b** Results of Gene Ontology (GO) enrichment analysis of the included dispensable genes, against a background of the included dispensable genes plus the putatively dispensable genes removed during filtering for not being consistently associated with an SV (see Supplementary Data 14), with points coloured by significance in other GO enrichment analyses. Values along the x-axis represent the ratio of the observed number of genes in the included dispensable genes to the number of expected genes in the total background set. Y-axis values represent the -log_10_-transformed *p*-values from the GO enrichment test (Fisher’s exact test, one-sided; *p-*values adjusted using Bonferroni correction are plotted). The red dashed line indicates the significance threshold. Points are coloured according to whether those GO terms were enriched in GO enrichment tests for other sets of genes. Grey points represent GO terms identified as significantly enriched in the genes removed during filtering, against a background of excluded plus included genes in the pangenome. Purple points represent GO terms identified as significantly enriched in genes classified as variable after filtering, compared to a background of all genes included after filtering. Blue points represent GO terms identified as significantly enriched in both the previous comparisons described. The remaining GO terms are coloured in yellow. Source data are provided as a Source Data file.

Functional annotation indicated that high-confidence dispensable genes overall had fewer annotations than invariant genes; putatively dispensable genes that were filtered out also had a similar reduction in functional annotations compared with invariant genes (Fig. 4a). This may be a result of these gene subsets being less conserved across species and/or less well annotated. High-confidence dispensable genes were enriched for eight GO terms, including 'RNA-templated DNA biosynthetic process', 'retrotransposition' and 'floral meristem growth' (Supplementary Data 4). Putatively dispensable genes that were filtered out were enriched for 28 terms, including 'defense response' and 'regulation of auxin polar transport', that were not significant in the high-confidence dispensable genes (Supplementary Data 5). However, a direct comparison of the high confidence dispensable genes with the filtered genes provided little evidence of GO terms only enriched in the dispensable genes, or the filtered genes, differing meaningfully in frequency between these two sets (Fig. 4b). The 3412 dispensable genes include those with GO terms indicating a role in response to stress (355), developmental process (341) and defence response (141), with 26 genes linked to a function in 'defense response to fungus' specifically (Supplementary Data 4). Dispensable genes with a 'defense response' GO term belonged to a variety of protein superfamilies (Supplementary Data 6), including cytochrome P450s (three genes) and flagellin-sensing 2 (FLS2)-related serine/threonine protein kinases (two), with Nucleotide Binding Site, Leucine Rich Repeat (NBS-LRR) superfamily being the most frequently annotated (eight genes). Genes annotated for functions in leaf development (42) and leaf senescence (16) were also included within the dispensable set—variation in Spring leaf flushing and Autumn leaf senescence has been linked to low ADB susceptibility in some studies^38,44,69,70^.

### Read mapping, SNP calling and SV calling using the pangenome

We next tested whether using the pangenome reference improved read mapping for short-read sequenced individuals, compared to the BATG-1.0 linear reference sequence. While overall mapping to the pangenome produced little effect on the total number of short reads mapped in proper pairs (Supplementary Fig. 10), we observed general improvements based on other metrics: this includes increases in mean mapping quality and alignment score (1.4% and 1.2% on average, respectively Supplementary Fig. 10), a reduction in the total number of substitutions (3.0% on average, Supplementary Fig. 10), and a large reduction in soft clipped sequence (33.5% on average, Supplementary Fig. 10). Overall, this indicates the pangenome improves the accuracy of short-read read mapping. Mapped reads in each case were used to call SNPs—overall numbers of SNPs and quality metrics were similar using both approaches; only 5.6% and 3.4% of SNPs called in the reference-based and pangenome approaches, respectively, were not called with the other approach. The quality of SNPs that were not detected with both approaches appeared similar to the general background set of SNPs (Supplementary Figs. 11 and 12, Supplementary Table 2). The median distance of unique SNPs from one approach to SNPs from the other approach was <30 bp for both callsets, although the reference-based callset included some SNPs >1 kb from the nearest SNP in the pangenome-based callsets (Supplementary Fig. 12). This indicates that while the improved overall mapping to the pangenome results in some SNP-calling differences, the overall impact on SNP-based analysis is likely to be minimal compared to that associated with using a high-quality linear reference genome.

We next called SVs using short-read sequenced samples. Examination of allelic depths of homozygous alternate, reference and heterozygous sites showed SVs and SNPs exhibit similar distributions of read support for reference and alternate alleles (Supplementary Fig. 13). The minor allele frequency (MAF) distributions and deviation from Hardy–Weinberg equilibrium (HWE) for the two variant types were also similar (Supplementary Fig. 14a, b). Patterns of linkage decay with distance were broadly similar in SNP-SNP, SV-SV and SNP-SV comparisons, with slightly faster linkage decay between SVs and between SVs and SNPs, compared with between SNPs (Supplementary Fig. 14c). Together, these suggest SNP and SV calls behave in a similar way for individually sequenced samples.

To investigate the accuracy of allele frequency estimations from short-read poolseq data, we constructed an in silico pool with an equivalent number of individuals and depth to the average in vitro pool from Stocks et al.^42^. Allele frequencies for SNPs were estimated using Popoolation2^71^. Similar software does not exist for estimating SV allele frequencies in poolseq data using a pangenome. Whilst vg^68^ does not explicitly support poolseq allele frequency estimation, we called SVs for the pools using vg and used the depths of reference vs. alternate alleles to estimate SV frequency in the pools. Correlation between the alternate allele frequencies from the individual based calling and estimates from the in silico pool was high for SNPs (slope = 0.94, *R*^2^ = 0.93; Supplementary Fig. 15a). For SVs, whilst the overall correlation was also high (slope = 0.96, *R*^2^ = 0.85), there were a minority of SVs where the pool-based estimates differed substantially from the individual based calling (Supplementary Fig. 15b). Poolseq data from Stocks et al.^42^ was mapped to the pangenome, and SNP and SV frequencies analysed. PCA recovered similar population structures from SV and SNP frequencies estimated using these pools (Supplementary Fig. 15c, d) as has been found in other studies^68,72^. Together, these results suggest that allele frequencies of most SVs can be captured accurately by mapping poolseq data to a pangenome.

### GWAS with SVs and SNPs

We reanalysed poolseq data from Stocks et al.^42^ using the pangenome. In that study, for 13 seed sources, a pool of healthy trees (no visible signs of ADB damage) and a pool of unhealthy trees (for which the majority of individuals had a dead/partially dead main stem; 42 trees per pool on average) from field trials heavily affected by ADB were sequenced (see 'Methods' for details). We performed a GWAS for low ADB susceptibility using SV allele frequencies estimated from this data, with a two-sided Cochran–Mantel–Haenszel (CMH) test^73,74^. This test identifies associations of treatments and outcomes while taking into account stratification—it is a commonly used statistic for identifying associations from paired poolseq data^42,71,75–79^. This identified 531 significant SVs with a *p*-value cutoff of 1 × 10^−13^ (the same significance threshold as used for the CMH test in Stocks et al.^42^; Supplementary Fig. 16a). However, many of these sites showed inconsistent allele frequency estimates from the in silico pool vs. individual genotyping as assessed in the above section (261 had a difference in estimated frequency of >0.2; see Supplementary Fig. 16b vs. Supplementary Fig. 15b). Whilst some of the 531 significant SVs may be truly associated with low ADB susceptibility, many are likely false positives resulting from errors in the estimation of allele frequencies. These results indicate SV allele frequency estimates from poolseq data are insufficiently accurate for a reliable GWAS.

We next considered the estimated allele frequencies for SNPs called from the Stocks et al.^42^ poolseq data using our pangenome, and used these to perform a separate GWAS for low susceptibility to ADB using the CMH test. This displayed high *p*-value inflation (Supplementary Fig. 17a). 866 SNPs were identified as significant after Bonferroni correction with *ɑ* = 0.05 (uncorrected *p*-value threshold = 1.52 × 10^−9^), and 404,503 with 5% false discovery rate using the Benjamini–Hochberg procedure. 92 SNPs had a *p* < 1 × 10^−13^ (the significance threshold used for the CMH test in Stocks et al.^42^). Wiberg et al.^75^ demonstrated that heterogeneity in SNP allele frequency shifts among pairs of pools can be identified as a main effect, causing *p*-value inflation in the CMH test. Deviation from homogeneity, as measured either by a significant Woolf test^80^ *p*-value (*p* < 0.05: 53.4% of significant SNPs), or by an *I*^*2*^ statistic >0.25 (73.2% of SNPs)^81^, was common, and was particularly pronounced for SNPs with the lowest CMH test *p*-values (Supplementary Fig. 18). Indeed, many of the SNPs with the lowest *p*-values (<1 × 10^−13^) had high read depth variation between pools (Supplementary Fig. 19), consistent with the presence of rare SVs distorting estimates of allele frequency (see Supplementary Note 1 for further discussion).

A quasibinomial generalized linear model (qGLM) test—a GLM for proportion/binomial data including an overdispersion parameter—has been shown to be better able to identify consistent allele frequency shifts across heterogeneous pairs of pools^75^. This analysis did not identify any significant sites, although substantial *p*-value deflation suggests this test may be overly conservative (Supplementary Fig. 20a). Indeed, the lowest *p*-values displayed consistent allele frequency shifts, but with shifts in allele frequency between healthy and unhealthy pools similar in magnitude to random subsets of SNPs (Supplementary Note 2 and Supplementary Fig. 20b–F). Different approaches to modelling the effect of provenance using GLMs either led to a similar lack of significant SNPs, or substantial *p*-value inflation (Supplementary Note 2 and Supplementary Fig. 21). This indicates limited evidence for SNPs behaving in a highly consistent manner across pools. We attempted to explicitly assess heterogeneity in allele frequency changes across pairs of pools using the fixation index (*F*_*ST*_) or Fisher’s exact test, and considering sites where multiple pairs of pools showed a strongly supported allele frequency shift. However, this proved ineffective for identifying sites shared by subsets of pools (with minimal overlap found even between biological replicates from Stocks et al.^42^), likely due to the inherent noise in individual allele frequency estimates from poolseq data (see Supplementary Note 3).

**Fig. 5:**
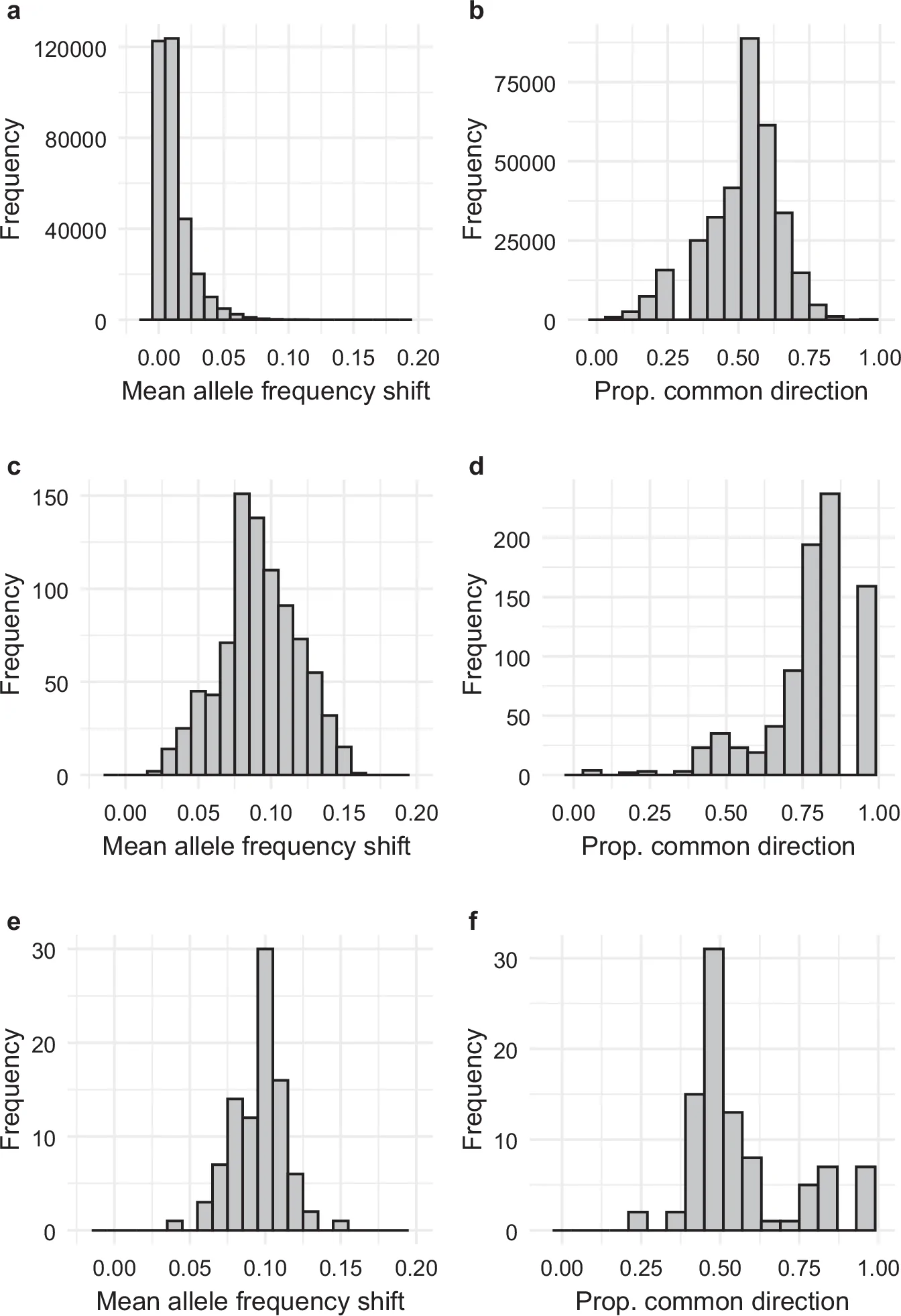
Shifts in direction between healthy and unhealthy pools in candidate SNPs compared to background SNPs. For a random 1% of SNPs estimated from poolseq data (**a**, **b**), and SNPs with significant *p-*values from a two-sided Cochran–Mantel–Haenszel CMH) test, with Bonferroni correction, *ɑ* = 0.05, (**c**, **d**), and SNPs with a CMH *p* < 1 × 10^−^^13^ (**e**, **f**), the mean absolute value of the allele frequency shift between healthy and unhealthy pools (**a**, **c**, **e**), and the proportion of all pairs of pools shifting in the most common direction (**b**, **d**, **f**). Source data are provided as a Source Data file.

Despite inflated *p*-values, the 866 SNPs passing the CMH test Bonferroni correction displayed large allele frequency shifts compared to the genome-wide average, also displaying a consistent direction of change in most pairs of pools (Fig. 5), suggesting they are likely enriched for SNPs associated with ADB resistance in the majority of seed sources analysed. Of these SNPs, 220 did not violate the CMH test assumption of homogeneity, or display high standard deviation in read depth potentially resulting from SVs not captured in the pangenome (see 'Methods'), suggesting their low *p*-values are more likely to represent a true association with tree health status. Thirty-four genes contained one or more of these SNPs, including two encoding pentatricopeptide-repeat containing proteins, ribosomal subunit proteins, and transcription and translation factors (Supplementary Data 7). A further 177 genes were within 10 kb of these SNPs (Supplementary Data 8), of which 16 were dispensable. No GO terms were significantly enriched in either the 34 genes overlapping the SNPs, or the entire set of 211 genes within 10 kb, after correction for multiple testing (one-sided Fisher’s exact test; Bonferroni correction with *ɑ* = 0.05; uncorrected *p*-value threshold of 1.53 × 10^−5^; Supplementary Data 9 and 10), with 'immune response-activating cell surface receptor signaling pathway' and 'brassinosteroid metabolic process' among those in the 10 most enriched terms.

These 211 genes formed 206 clusters in OrthoFinder—those containing sequences identified in six other genomic or transcriptomic studies investigating low susceptibility to ADB were identified^42,82–85^ (Supplementary Data 11 and Supplementary Fig. 22). Two clusters contained genes differentially expressed between uninfected leaf tissue of low vs. high susceptibility genotypes^50^, four and twelve contained genes differentially expressed between infected and uninfected rachises of (i) a low susceptibility genotype, and (ii) a high susceptibility genotype, respectively^82^. Thirty-seven and 20 clusters contained genes differentially expressed between uninfected and infected petiole tissue in (i) a resistant genotype, and (ii) a susceptible genotype, respectively^83^. Nineteen clusters contained genes differentially expressed between infected lesions following artificial inoculation and distant uninfected bark tissue; one contained genes differentially expressed between lesions in low vs. high susceptibility genotypes; 14 contained genes differentially expressed in distant uninfected bark tissue following artificial inoculation between low and high susceptibility genotypes^84^. None of the clusters contained *F. excelsior* homologues of genes displaying signs of convergent evolution to ADB in other *Fraxinus* species^85^. Twenty-one of the clusters contained 22 of the 55 genes identified as near to significant SNPs in Stocks et al.^42^. Excluding those from Stocks et al.^42^ (which were identified with the same samples as this study, so do not represent an independent source of evidence), genes identified across all these studies were found in 3655 gene clusters, of which 57 were among the set of 206 clusters containing our candidate SNPs. An overlap of 57/206 was higher than 99.2% of 10,000 sets of randomly selected groups of 3655 gene clusters (Supplementary Fig. 23a). The 3655 clusters with genes identified from other studies contained 584 dispensable genes in 382 clusters—of 10,000 randomly selected sets of 3655 clusters, none contained as many dispensable genes (Supplementary Fig. 23b).

## Discussion

Here, we present a chromosome-level linear reference genome (BATG-1.0) and a pangenome for European ash from a geographically diverse sample of individuals, enriched for those with low susceptibility to ADB. The pangenome of 50 individuals captures 362,965 SVs, representing 396 Mb of variable sequence, including 174 Mb that is absent from the linear reference genome (equivalent to approximately 22% of its length). This is broadly similar to other recent pangenome studies, although there is substantial variability across these^3,15,86–99^ (Supplementary Fig. 24a, b and Supplementary Data 12). Our analyses likely underestimate the total SV diversity in *F. excelsior*, as each additional individual contributed novel SVs to the collection. A comparison of SVs called between the chromosome-level haploid assemblies and ONT data for the same individual indicates our SV calling approach has a precision of 72.2% (although a recall of 84.4%) compared with using chromosome-level phased assemblies, suggesting some SVs included in the pangenome may be false positives. However, it should be noted that (i) as heterozygous sites only have half the read support of homozygous alternate sites, it is likely the latter can be detected with higher recall and precision, and (ii) the fact that we only included SVs called in more than three samples in the pangenome potentially reduces the number of false positives, although at the cost of excluding rare SVs. Our results highlight the substantial diversity of SVs in European ash, consistent with high SNP genetic diversity^53^, but also underline that more extensive sampling is required to fully capture segregating SVs.

We identified 8.7% of genes in the pangenome as dispensable; this is substantially lower than most other plant pangenomes, which typically report 20–60% of genes or gene families as being variable^3,15,86–99^ (Supplementary Fig. 24c and Supplementary Data 12). As noted above, our pangenome only contains SVs called in more than three individuals, so potentially misses the rarest dispensable genes. However, most of the difference in the proportion of genes that are dispensable is explained by our filtering of genes not reliably associated with SVs; without this filter, a more typical 35.9% of genes in the pangenome are classified as dispensable (Supplementary Fig. 24c and Supplementary Data 12). Despite leading to a dramatic reduction in dispensable genes, we believe this filter is justified as variability in the annotation process has previously been found to underlie putatively dispensable gene sets^96,99^. Indeed, previous pangenome studies have identified core genes as being more highly expressed^7,88,91,94,100,101^. This relationship could be due to these genes performing critical biological functions, but also could be the result of more highly expressed genes having more RNA-seq data evidence and being more accurately annotated as a result. Functional groups typically found as enriched among dispensable genes in other studies, such as defence and auxin response^3,7,88,96,98,102^ were enriched in our initial set of putatively dispensable genes, but not following our filtering process. It should, however, be stressed that there was no clear functional distinction between the final set of dispensable genes and those removed during filtering—a smaller set of genes will inherently have lower power to reliably detect enrichment in GO terms. More comprehensive RNA-seq sampling from tissues/conditions across individuals, and manual curation of repetitive elements, may lead to more genes being annotated—including as dispensable—in future studies. We should also note that we aimed to identify genes specifically within our characterised SVs; in studies where de novo assemblies are explicitly incorporated (e.g. all-against-all pangenomes and comparative genomics more broadly), specific testing of DNA sequence differences underlying presence/absence of genes should be performed, as has been suggested elsewhere^103^. Our results emphasise the importance of confirming that putatively dispensable genes are driven by underlying sequence differences, to distinguish them from possible non-biological annotation variability. The genes we identified as dispensable with high confidence include 141 with homology to defence-related genes, one of which was within 10 kb of the 220 SNPs associated with low ADB susceptibility, emphasising the importance of pangenomes in understanding the mechanistic basis of disease resistance traits.

Using a pangenome reference allows improved short-read mapping for *F. excelsior*, as has been found for other species^11^, and we were able to successfully call individual SNPs and SVs from individually sequenced samples. However, there was not a clear improvement in SNP calling compared to using the BATG-1.0 linear reference genome. SV genotyping from short-reads is generally more challenging than for SNPs^104–106^, and we are not aware of previous studies estimating allele frequencies of poolseq data mapped to a pangenome graph. Our results indicate poolseq data can be used to estimate allele frequencies accurately for most SVs (Supplementary Fig. 15b), but the approach used generates more discrepancies with individual-based genotyping than is the case for SNPs. SVs showing these discrepancies were particularly likely to be identified as significant in a GWAS using a CMH test, resulting in many likely false positives. We were unable to identify the cause of these discrepancies (https://github.com/vgteam/vg/issues/4443). We should emphasise that vg is not designed for analysing poolseq data; the development of dedicated software for estimating SV allele frequencies from poolseq data, equivalent to those available for SNPs, is required in order to make full use of poolseq data in the pangenome era. Nevertheless, some of the SVs identified as significant from the CMH test are potentially involved in low ADB susceptibility; future studies using individually genotyped data could be used to confirm this.

Despite broadly accurate SNP allele frequency estimates from poolseq mapped to a pangenome, our reanalysis of data from Stocks et al.^42^ highlighted known challenges of analysing poolseq data^107^. The commonly used CMH test can assign very low *p*-values to heterogenous allele frequency shifts among pairs of pools^75^, and uncharacterised SVs can distort allele frequency estimations. A combination of these sources of error meant that despite mapping to a pangenome and using relatively stringent depth filtering, a likely rare, large/high copy number SV produced the lowest *p*-values in our dataset using this test (Supplementary Note 1). We find a subset of 220 SNPs with significant *p*-values from the CMH test that do not violate its assumptions of heterogeneity, or display unusual patterns of depth, and shift in frequency between unhealthy and healthy pools in a broadly consistent manner across most seed source zones (Fig. 5). We would note the inflation of *p*-values from the CMH test makes the threshold of inclusion in this group (passing Bonferroni correction with *ɑ* = 0.05) somewhat arbitrary. Nevertheless, these results indicate a shared genetic component to low susceptibility exists across a broad range of provenances. It should be noted that more densely sampled regions in the Stocks et al.^42^ dataset (e.g. Great Britain) may make SNPs consistently shifting in these regions more likely to be identified. Variation in the frequency of trees with low susceptibility exists between provenances^35,36^, suggesting there may be further geographically restricted genetic variation associated with low ADB susceptibility, although we are not able to accurately characterise this in our dataset. Higher coverage per pool, or individual-based sequencing, would allow more accurate characterisation of variants restricted to subsets of provenances.

In Stocks et al.^42^, the consistency of allele frequency shifts, and the potentially confounding effect of SVs, were not explicitly examined. Indeed, the maximum per-pool depth for sites considered was 37.5× the average coverage, rather than the more stringent 2.5× in the present study, increasing the potential for SVs to impact allele frequency estimates (see Supplementary Note 4). It is likely therefore that the 192 SNPs with *p*-values lower than 10^−13^ in Stocks et al.^42^ are similarly, or more severely, enriched for problematic sites, as those in our study. This may explain the lack of correlation of CMH *p*-values with estimated effect sizes in genomic prediction (GP) of ADB damage, and the fact that around two-thirds of their top 192 GWAS hits are ranked outside the top 2500 SNPs by GP effect size (Stocks et al.^42^). It may also explain a lack of shared genes between Doonan et al.^44^ and Stocks et al.^42^, although we should note that ADB health scores represent a complex phenotype underpinned by several mechanisms, which may reduce the chances of identifying similar underpinning regions across studies. Nevertheless, a GP model trained on the top 10,000 GWAS loci from Stocks et al.^42^ was able to predict tree health status with an accuracy of 67%^42^, despite likely including many loci violating the assumptions of the CMH test. This is consistent with our results, which suggest that despite containing false positives, SNPs with significant CMH test *p*-values after Bonferroni correction are nevertheless enriched with loci that display broadly consistent allele frequency shifts between healthy and unhealthy pools.

Whilst no GO terms were significantly enriched in the 211 genes near our candidate SNPs, 57 out of 206 of orthogroup clusters containing these genes also contained genes matching those showing gene expression differences between healthy vs. infected tissue, or low vs. high susceptibility genotypes^50,82–84^, an overlap greater than expected by chance (Supplementary Data 11 and Supplementary Fig. 23). The 211 genes within 10 kb of our candidate SNPs have a variety of potential roles in low ADB susceptibility. Several are homologues of *Arabidopsis* genes associated with changes in vernalisation, flowering or leaf senescence timing: VERNALISATION-INSENSITIVE 3-LIKE, FCS LIKE ZINC FINGER 6 and RETINOL DEHYDROGENASE^108–110^. Changes in phenology such as early leaf flushing and senescence have been implicated in low ADB susceptibility^111^, potentially associated with avoidance of pathogen infection. Genes associated with pathogen detection pathways, such as an NBS-LRR, a wall-associated kinase, five receptor-like kinases and a chitin receptor, some of which showed differential expression in response to ADB infection, were near significant SNPs. Genes involved in the regulation of hormone signalling were detected—including a homologue of *Arabidopsis* JASMONATE-INDUCED DIOXYGENASE 1, which regulates jasmonic acid homoeostasis in *Arabidopsis*^112^ and is differentially expressed in early infection by *H. fraxineus* in rachises and petioles^82,84^. Multiple homologues of cytochrome P450s were detected, some of which were differentially expressed in leaves and/or lesions^82,84^. Cytochrome P450 genes were also near the highest-ranked SNPs predicting low ADB damage in Doonan et al.^44^. Homologues of genes encoding aspartic peptidases and glucanases, which can degrade fungal cell walls^113,114^, were identified—a homologue of the aspartic peptidase detected was differentially expressed in infected petioles of a low susceptibility genotype, but not the high susceptibility genotype^83^. Genes producing antifungal compounds were also detected—such as a homologue of glutamate decarboxylase, differentially regulated in lesions, which produces GABA, a compound with antifungal properties^115,116^. A homologue of *Arabidopsis* DIRIGENT PROTEIN 1 was identified—this is involved in the biosynthesis of lignans^117^, compounds associated with low ash dieback susceptibility^111,118^. Pectate lyases and pectinase inhibitors may be involved in specific pathogen responses, such as cell wall remodelling, or preventing degradation by the fungus^119^, or more indirectly in mediating phenological changes^44^. Doonan et al. identified a pectinesterase inhibitor as being associated with autumn leaf yellowing, but not low ADB crown damage^44^. The pectate lyase was differentially expressed in developing lesions in high but not low susceptibility genotypes^84^, as well as between low susceptibility and high susceptibility tissue distant to the site of artificial inoculation^84^, and between leaves of high and low susceptibility plants^50^. This suggests pectin metabolism may be involved in both avoidance of and active resistance to ADB. Only two gene clusters overlapped with genes differentially expressed between healthy low vs. high susceptibility individuals^50^. It could be that genetic differences in low ADB susceptibility are more strongly associated with induced than constitutive expression difference in leaves, or that constitutive differences are mediated by trans-acting regulatory processes—several significant SNPs in this study are near transcription factors (Supplementary Data 11). The only gene in this study homologous to those differentially expressed in lesions between low and high susceptibility genotypes was a dispensable RNA polymerase associated with transcriptional silencing; knockouts of the *Arabidopsis* homologue to this gene are more vulnerable to fungal infection^120^. No genes overlap with those identified as candidates for fungal defence response in *Fraxinus* lineages that co-evolved with the pathogen^85^—supporting the suggestion of that study that mechanisms underlying low susceptibility in *F. excelsior* may differ from those in the natural hosts of *H. fraxineus*. This study indicates a variety of mechanisms underpin low ADB susceptibility, increasing the chances that the underlying genetic variants may be additive in their effects and suggesting natural selection or a breeding program may increase low ADB susceptibility.

Overall, the *F. excelsior* pangenome captures a diverse set of SVs from across the geographic range of European ash, including a large quantity of previously uncharacterised sequence and dispensable genes, allowing fuller investigation of the role of both SNPs and SVs in a breadth of ecological traits for this important tree species. After carefully accounting for spurious signals in our GWAS, we detect signals of a shared component of low susceptibility to ADB across UK provenances. Explicitly identifying variants with consistent effects across a range of seed sources may be useful in developing widely applicable genomic prediction models for low ADB susceptibility, and ultimately informing breeding programs to restore this threatened species.

## Methods

### Reference assembly

To construct a phased, chromosome-level assembly, we sampled a potted graft growing at Royal Botanic Gardens, Kew. The graft was created by Forest Research in 2017 using a rootstock and a scion from *F. excelsior* individuals of unknown provenance. Leaf tissue from this graft was flash-frozen in liquid nitrogen in May 2022 and sent to Cantata Bio, CA, USA (formerly Dovetail Genomics) on dry ice, where DNA extractions, library preparation, sequencing and genome assembly was performed as outlined below.

DNA was extracted by Cantata Bio using an in-house CTAB protocol and quantified using a Qubit 2.0 Fluorometer (Life Technologies, Carlsbad, CA, USA). The SMRTbell Express Template Prep Kit 2.0 (PacBio, Menlo Park, CA, USA), using the manufacturer-recommended protocol, was used to construct a PacBio SMRTbell library (~20 kb) for PacBio Sequel. The library was bound to polymerase using the Sequel II Binding Kit 2.0 (PacBio) and loaded onto the PacBio Sequel II). Sequencing was performed on PacBio Sequel II 8 M SMRT cells, resulting in 66 Gb data (approximately 79× coverage based on the prime 1C genome size estimate for *F. excelsior* of 840 Mb^54,121^). For the Omni-C libraries, chromatin was fixed in place with formaldehyde in the nucleus and then extracted. Fixed chromatin was digested with DNase I; chromatin ends were repaired and ligated to a biotinylated bridge adapter, followed by proximity ligation of adapter-containing ends. After proximity ligation, crosslinks were reversed and the DNA was purified. Purified DNA was treated to remove biotin that was not internal to ligated fragments. Sequencing libraries were generated using NEBNext Ultra enzymes and Illumina-compatible adapters. Biotin-containing fragments were isolated using streptavidin beads before PCR enrichment of each library. The library was sequenced on an Illumina HiSeqX platform to produce approximately 30× sequence coverage in 150 bp paired-end reads. The combined Illumina and PacBio data was used to produce an initial assembly with hifiasm v0.15.4-r347^122^ using default parameters. The de novo assembly and Dovetail OmniC library reads were used as input data for HiRise^123^ to produce a draft assembly. OmniC library reads were then aligned to the draft input assembly using BWA^124^, and reads with MQ < 50 removed. The mapping information was analysed using HiRise to produce a likelihood model for genomic distance between read pairs, and the model was used to identify and break putative misjoins, and to score and make joins.

After receiving the assemblies and raw data from Cantata Bio, the haploid genome assemblies were assessed using QUAST v5.2^125^ and BUSCO v5.5.4 using the eudicots_odb10 database^126^. To assess potential contaminants, scaffolds from the most contiguous of the two haploid genomes (BATG-1.0) were searched against the NCBI nt database (downloaded 21/02/23) using blast+ v2.11.0^127^ with settings -max_target_seqs 10 -max_hsps 1 -evalue 1e-25. To assess coverage, the PacBio data was mapped to the haploid genomes using minimap2 v2.5^128^. The combined data was analysed using blobtools v4.0.7^129^. To assess LTR completeness, LTRharvest from genome-tools v1.6.6^130^ and LTR_finder v1.07^131^ were run with default parameters, other than specifying a 85% similarity threshold, a length range 100–5000 bp, starting/ending motif TG/CA and a maximum distance of LTR starting positions of 20,000. LAI was calculated using LTR_retriever v3.0.4^56^. To estimate genome size using kmers, LVgs v1.00^132^ was run on the PacBio fastq files, with kmer sizes between 17 and 77 bp in increments of 10, and results analysed using Genomescope2 v2.1.0^55^_._

The PacBio data was used to de novo assemble a chloroplast genome for *F. excelsior* using Oatk v1.0^133^, using HMMER v.3.1b2 (hmmer.org) with parameter settings -c 30 -k 1001 and a chloroplast gene profile database provided by OatK (embryophyta_pltd). Annotation was performed using GeSeq v2.03^134^ using default settings.

### Long-read RNA extraction and sequencing

Long-read RNA-seq data from leaf and cambium tissue was generated to annotate the new genome assembly from the reference individual. Leaf material was collected from the same grafted tree as sampled for the reference assembly (see above) in May 2022 and flash-frozen in liquid nitrogen. For cambium tissue, twigs were sampled in January 2023, and the layer of tissue immediately below the bark was isolated using a fresh razor blade before flash-freezing in liquid nitrogen. Tissue was stored in a −80 freezer until RNA extraction.

For long-read RNA extraction, a modified version of the Qiagen RNeasy Plant Mini Kit standard protocol was used (Supplementary Method 1). In brief, 25–30 mg tissue was used with the standard kit protocol (including the optional DNase step) and eluted in 50 µl RNase-free water. Four hundred fifty microliters of buffer RLT was added to the RNA, and the RNeasy kit protocol was repeated (without the DNase step). RNA was quantified using a NanoDrop 2000 and a Promega QuantiFluor® RNA System Kit using a Quantus fluorometer. Samples were shipped to the Deepseq facility at the University of Nottingham, and fragment length was determined with an Agilent Tapestation using an RNA Screentape kit.

cDNA libraries were prepared using an Oxford Nanopore PCR-cDNA Sequencing Kit (SQK-PCS109), loaded onto R9.4.1 flowcells and sequenced on a Gridion X5 Mk1—this was performed once for the cambium library and twice for the leaf library. Runs were performed with high-accuracy basecalling (running on MinKNOW software version 22.12.5). All data reported relates to reads that passed a base calling quality filter (PHRED score >9).

### Sampling for pangenome construction

In sampling individuals for the ash pangenome, we aimed to (i) sample a broad range of European populations, with (ii) a particular focus on UK diversity by sampling individuals representing different Native Seed Zones (NSZ) in the UK^135^ (https://assets.publishing.service.gov.uk/government/uploads/system/uploads/attachment_data/file/797909/Regions_of_provenance_and_seed_zone_map.pdf). With this approach we aimed to capture a global set of variants from across the species range, whilst increasing our chances of capturing SVs segregating in the mainly UK populations for which short-read data is available from Stocks et al.^42^.

In total, 50 samples were used for long-read sequencing. To maximise the chances of identifying SVs segregating in the poolseq data from Stocks et al.^42^ one individual from each of the 13 NSZ used in the Forest Research (FR) mass screening field trial at North Burlingham, Norfolk, UK (Site 16)^42^ was sequenced, with the exception of NSZ-204 where two were sequenced, as individually sequenced data for this provenance were available from Stocks et al.^42^. The North Burlingham site was planted in 2013 with 2-year-old saplings that were grown for non-research purposes in commercial nurseries from seed sources from 15 different provenances. The site contained four complete replicate plots for each seed source, with 5000 trees per ha (a spacing of 1 m× 2 m). At the time of sampling, the site had high *H. fraxineus* inoculum pressure from naturally established infections, and ADB damage was severe throughout. In 2022 (when trees were 11 years old) individuals were sampled that appeared relatively healthy within each plot, and which scored between 3 and 5 on the damage scale of Pliura et al.^37^ (where 3 is a tree with moderate damage from ADB, 4 a tree with slight damage and 5 a tree with no visible signs of damage) to increase the chances of capturing alleles for low ADB susceptibility. Three wild trees of unknown age from seed zones in the UK not covered by the FR trial populations (NSZ-305, NSZ-406, NSZ-402) were also included to further capture UK diversity. To increase the likelihood of including more of the range-wide diversity of structural variation in *F. excelsior*, one individual from each of 29 mostly mainland European provenances was sampled from the Realising Ash’s Potential (RAP) trial (established in 2005), as well as two from provenances in the FRAXIGEN trial (established in 2004), both at Paradise Wood, Earth Trust, Oxfordshire. Finally, leaf material from the individual used for the linear reference genome was also included. Sample information is outlined in Supplementary Data 3, including approximate coordinates of origin where available. These coordinates are plotted in Fig. 1a on a map of Europe from rnaturalearth v1.2.0, overlaid with a shapefile of the *F. excelsior* native range from Caudullo et al.^136^. We note our sampling does not comprehensively cover the geographic range of *F. excelsior*—in particular, the far southwestern, southeastern European, West Asian, and far northern areas of the range are unrepresented in our samples (Fig. 1a).

Sampling for long-read DNA sequencing was performed in May 2022. For samples collected in the field, intact stems with leaves were excised from individual trees using secateurs or a pole pruner, wrapped in damp tissue paper and sealed in airtight plastic bags before returning to RBG Kew for storage at 4 °C. Leaf tissue was removed from the stems and frozen within 48 h of collection by immersing in dry ice, and then stored at −80 °C prior to DNA extraction.

### High molecular weight DNA extraction and RNA extraction from pangenome samples

For high molecular weight DNA extraction, a modified CTAB protocol was used^50^ (see Supplementary Method 2 for full protocol). In brief, 300–400 mg tissue was disrupted in liquid nitrogen and added to 8 ml Carlson buffer with 20 µl beta-mercaptoethanol and 40 µl Qiagen Proteinase K. This was incubated at 65 °C for 1 h, before adding 16 µl Qiagen RNase A and incubated for a further 15 min. One volume of SEVAG solution was added, the tube was inverted, and centrifuged for 10 min at 4 °C before the aqueous phase was removed. This step was then repeated. 0.7 volumes of isopropanol was added, and the tube was incubated at 4 °C for 15 min. The sample was centrifuged at 4 °C for 30 min to precipitate the DNA and resuspended in Qiagen Buffer AE before being redissolved in 5 ml Qiagen Buffer G2 for 15 min. A Qiagen Genomic Tip 100/G Kit was used to purify the DNA; the Isolation of Genomic DNA from Blood, Cultured Cells, Tissue, Yeast or Bacteria using Genomic-tips protocol from the kit handbook was used, with the modification of using 10 ml rather than 7.5 ml buffer QC. DNA was eluted in 100 µl buffer 1× TE and left to resuspend at room temperature and then at 4 °C for up to 3 and 4 days, respectively. DNA was quantified using a Quantus Flurometer with QuantiFlour dsDNA kit and a NanoDrop 2000. Integrity was measured using an Agilent Tapestation with a Genomic DNA kit, and the DNA was shipped to the University of York on dry ice to prevent fragmentation during transit, where library preparation was performed as described below.

Short fragment elimination was performed using a Circulomics SRE Standard Kit. Library preparation was performed using the Oxford Nanopore Technologies (ONT) SQKNBD114.24 (Protocol version NBE_9169_v114_revD_15Sep2022-promethion.pdf) library preparation kit, with the following modifications: (i) reaction volumes were proportionately scaled up to 60 μl total for end repair and A-tailing, and incubation times increased to 30 min each at 20 and 65 °C, (ii) reaction volumes were doubled for the barcode adapter ligation step, which was performed at room temperature for 1 h. Barcode-ligated samples were bead-cleaned separately before pooling at appropriate volumes prior to adapter ligation. Native barcodes were ligated and reads demultiplexed by barcode following the run. Libraries were loaded onto PromethION flowcells, runs commenced on a Gridion with live super accuracy base calling (running on MinKNOW software version 22.10.5) with barcode demultiplexing and adapter trimming. All data reported relates to reads that passed a base calling quality filter (PHRED score >10).

For 35/50 samples with remaining leaf tissue after DNA extraction, RNA from frozen material was extracted for short-read sequencing (outlined in Supplementary Data 3). For one additional sample (PG5), fresh leaf tissue was collected in May 2024 and placed into Thermo Fisher RNAlater, and then stored at 4 °C until RNA extraction in November 2025. The standard Qiagen RNeasy Plant Mini Kit Protocol was used, without the optional DNase step. Samples were sent to the Centre for Genomic Research (CGR), Liverpool, for library preparation and sequencing. Analysis using an Agilent Tapestation did not reveal any evidence of genomic DNA contamination. cDNA libraries were prepared using a New England Biolabs NEBNext polyA selection and Ultra II Directional RNA library preparation kit, and sequenced on half a lane on an Illumina NovaSeq X Plus using 25B chemistry, with paired-end 15-bp reads. Raw data were trimmed by CGR using Cutadapt^137^ v4.5 with option -O 3. Further trimming was performed using Trimmomatic^138^ with settings LEADING:7 TRAILING:7 ILLUMINACLIP:2:30:7 MINLEN:70, resulting in an average of 63 million read pairs per sample after trimming (Supplementary Data 3).

### Long-read DNA data analysis

The simplex ONT DNA sequence data generated was trimmed, firstly using Trimmomatic^138^ with settings LEADING:7 TRAILING:7, followed by Nanofilt v2.8.0^139^ with minimum average read quality of 7 and length 1000. Quality was inspected and summary statistics generated using Nanoplot v1.4.10^140^, FastQC v0.11.9^141^ and multiQC v1.14^142^.

Reads were mapped to BATG-1.0 using minimap2 v2.5^128^, with PCR duplicates and reads with mapping quality (MAPQ) < 20 removed using samtools v1.9^124^. Mean depth after filtering was 22.7× (Supplementary Data 3).

To inspect population structure of these samples, SNPs were called individually for all samples other than the reference genome individual using Clair3 v.1.0.5^143^ with model r941_prom_sup_g5014. Gvcfs were merged using Glnexus v1.4.1-0-g68e25e5n^144^. Genotypes with DP <8 or >60 were set to missing using bcftools v1.16^145^, and sites with >10% missing data or MAF < 0.05 were excluded. Plink2 v2.00a3.3LM^146^ was used to identify unlinked SNPs (using the setting –indep-pairwise 50 10 0.1) and calculate a PCA using default settings.

Read-based SV calling was performed using the long-read data generated for all 50 samples using Sniffles2 v2.0.7^62^ and cuteSV v2.0.3^63^, two tools that rank highly across several comparisons of read-based SV calling software^147–154^. Both tools were run with a minimum read support of 5, and cuteSV with the additional parameters –max_cluster_bias_INS 100 –max_cluster_bias_DEL 100 –diff_ratio_merging_INS 0.3 –diff_ratio_merging_DEL 0.3. Breakends/translocations (BND) or sites which were genotyped as homozygous reference were removed.

To complement the read-based SV calling, de novo assemblies were produced from the ONT read data. De novo assembly from trimmed reads was performed using Shasta v0.11.1^65^ with the Nanopore-May2022 config file, Flye v2.9.2-b1786^64^ with default settings, and nextDenovo v.2.5.0^66^ with an estimated genome size of 850 Mb. Assembly contiguity was assessed using QUAST. SVs were called using these assemblies by mapping them to BATG-1.0 using minimap2 in asm10 mode. SVs were called with svim-asm v.1.02^67^ using alignments with a minimum MAPQ of 20 and a minimum SV size of 50. Haplotype 2 of the linear reference genome was also mapped to BATG-1.0, and SVs called using the above method. SV calls between the two pseudochromosomal haploid genomes were compared to SV calls from the ONT data from the same individual using the various approaches, with SV sets merged using SURVIVOR v1.0.7^155^ using settings 200 2 1 1 1 50 for the intersection of sets and 200 1 1 1 1 50 for the union. A 200 bp merge distance yielded similar numbers of SVs called with Sniffles2 and cuteSV to values between 10 and 300 bp yielded similar numbers of SVs, with larger or smaller values resulting in fewer SVs. Assuming the SV calls between the two haploid linear reference genomes are a truth set, precision, recall, and F1 were calculated for the various SV calling methods from the lower coverage ONT data. Based on these comparisons (see Results), SVs for each individual were included if they were called in at least one of (i) cuteSV and Sniffles2 (ii) svim-asm from mapping the Shasta assembly to BATG-1.0.

These SVs were merged across all 50 individuals using SURVIVOR with settings 200 2 1 1 1 50. SVs that overlapped with Ns in the reference were removed. SVs that were called in more than 3/50 individuals were identified using bcftools v1.16 and used for downstream analysis. The overlap between these SVs and the repeat annotation was assessed using the bedtools v2.28.0^156^ window function with parameter -w 1. Consensus sequences for insertions and deletions were searched against the ncbi nt database using blast+ with parameters -max_hsps 1 -evalute 1e^−25^, with the resulting data loaded into blobtools. Based on analysis with blobtools, three sequences were identified as putative contaminants and the associated SVs were removed from the VCF. The resulting VCF, as well as the BATG-1.0 FASTA was used to construct a pangenome using the vg v1.64.0^68^ construct command with option -v -S -a.

### Linear reference and pangenome annotation

Repeats were identified using RepeatModeler v2.0.1^157^ in LTRStruct mode. The outputs of this were combined with the custom repeat library used previously to mask the BATG-0.5 assembly^53^, and used to mask the genome with RepeatMasker v4.1.2^158^ with the parameters -wublast -nolow. Telomere sequences were identified using Teloscope v0.0.9^159^ with default settings using the canonical plant telomeric repeat CCCTAAA. To annotate the linear reference genome, short-read RNA-seq data from leaf, root, cambium and flower tissue that had been used to annotate the BATG-0.5 assembly^53^, was downloaded from the European Nucleotide Archive (ENA), as well as RNA-seq data from Emerald Ash Borer-infested and control phloem tissue^160^, and ADB-infested and control petiole tissue^83^ (Supplementary Data 13). Together with the short-read leaf RNA-seq data for the BATG-1.0 individual generated as part of this study, these data were mapped to BATG-1.0 using STAR v2.7.10b with default parameters^161^. The long-read RNA-seq data generated in this study was trimmed firstly using Trimmomatic v0.39^138^ with settings LEADING:7 TRAILING:7, followed by Nanofilt v2.8.0^139^ with minimum average read quality of 7 and minimum length 100. The trimmed data was then mapped to the BATG-1.0 reference genome using minimap2 v2.5^128^. We acknowledge that this RNA-seq data does not provide comprehensive coverage of tissues/timepoints/conditions in the species, and that some genes may therefore be missed. Variability in the gene annotation process could lead to false positives during the identification of variable genes^16,162^, so we repeated the annotation process multiple times and combined the results. BRAKER3^60^ was run 10 times on the softmasked genome using the BAM files from the short-read data, and protein orthologue data from Viridiplantae from OrthoDB v11^163^. A version of BRAKER3 capable of using long-reads (Singularity build braker3_lr.sif docker://teambraker/braker3:devel) was also run 10 times using the BAM files from the long-read data and the same protein orthologue set as used with the short-read data. To combine the outputs of the runs, the amino acid sequences of the longest transcript per gene (minimum length 30 amino acids) for each run were clustered into orthogroups using Orthofinder v2.5.5^61^. Within each orthogroup, groups of sequences with non-overlapping positions within the BATG-1.0 assembly (likely representing recent duplicates) were separated using the bedtools intersect utility. For each of these groups, the gene model with the longest transcript from the set of 20 BRAKER3 runs was chosen. In some cases, OrthoFinder split genes with largely overlapping coding sequences into separate groups. In cases where two genes with a coding sequence on the same strand overlapped by >50% for one of the genes, only the gene producing the longest protein was retained (for further discussion on clustering see Supplementary Note 5). The features for the selected gene models were combined into a single bed file. The amino acid sequences from the final set of transcripts was used as the input for Orthofinder, along with protein sequences for BATG-0.5^53^, *Fraxinus pennsylvanica*^51^, *Olea europaea* subsp. *europaea*^164^, *Solanum lycopersicum*^165^, *Erythranthe guttata*^166^, *Sesamum indicum*^167^ and *Salvia miltiorrhiza*^168^ (Supplementary Data 2).

To annotate the pangenome, sequences and locations of structural variants were used to modify the sequence of the BATG-1.0 FASTA, which was then annotated. As most SVs were defined from read mapping to BATG-1.0 rather than from the individual de novo assemblies, this approach maximises the opportunity for novel genes/gene deletions to be explicitly linked to underlying SVs. The steps were as follows (see Supplementary Fig. 25 for an explanatory diagram): for each individual, a non-overlapping set of SVs detected in that individual was extracted from the combined VCF of SVs used to build the pangenome. Where SVs overlapped, insertions were retained in priority to other types of SVs, and longer SVs were retained in priority to shorter ones, as these may be more likely to capture coding sequences absent in BATG-1.0. This process resulted in 2394 deletions, 96 insertions and 21 inversions being left unrepresented in any of the samples (0.7% of SVs). Tandem duplicates (0.5% of SVs), which were not included in the graph pangenome, were also excluded. Based on these sets of SVs, sequence was then inserted, deleted, or inverted in the reference FASTA (the SV sequence in the combined VCF of SVs used to build the pangenome was used, so the same sequence was used to represent a given SV in different individuals) to produce a transformed FASTA for each individual in turn. The transformed FASTA was then annotated for each individual. Repeats were annotated and softmasked as described above. Long and short read RNA-seq data from the reference individual were mapped to each transformed FASTA using the same methods as for the reference sequence. For the 35 non-reference individuals where short-read RNA-seq data was generated as part of this study, leaf RNA-seq data from each individual was also mapped to the corresponding transformed FASTA and used in downstream annotation. A single run of the short-read BRAKER3 protocol and a single run of the long-read BRAKER3 protocol was performed as detailed above. For these two runs for each individual, the Orthofinder approach as outlined previously was used to consolidate the outputs of BRAKER3 into a single set of proteins. Orthofinder was then run again for each set of proteins across the 50 individuals, as well as the proteins from the consensus annotation of BATG-1.0. Non-overlapping groups of sequences were separated using bedtools as before. For each gene, bedtools intersect was used to identify SVs that overlapped the gene sequence. Novel genes not present in the BATG-1.0 genome should overlap with an SV, as should deletions of genes present in the reference genome. For each novel gene that overlapped a SV at least once (i.e. in at least one sample, including the reference individual sequenced with long reads), an F1 score was calculated to identify the strength of the association between the new gene and the SV:

1 $$F1=\frac{2\times {GeneSV}}{2\times {GeneSV}+{GeneNSV}+{NGeneSV}}$$

where *GeneSV* is the number of samples with the gene and the SV, *GeneNSV* is the number of samples with the gene but without the SV, and *NGeneSV* is the number of samples without the gene but with the SV. For novel genes overlapping more than one SV, the SV with the highest F1 score was retained as potentially causal for the novel gene. An equivalent calculation was performed for genes present in the BATG-1.0 genome but absent in at least one sample, where an SV overlapped with the annotation coordinates for the gene in the reference. To account for genes potentially being associated with multiple SVs, we also calculated an F1 score of gene presence/absence with all overlapping SVs—if this scored higher than any individual SV, this score was taken forward. Genes with an F1 score of 0.8 or above were retained in the final pangenome annotation as being dispensable; for those with an F1 score of <0.8, those absent in the BATG-1.0 genome were removed, and those present in the BATG-1.0 genome were classified as invariant. Genes that did not overlap an SV in any sample were classified as invariant if they were annotated in BATG-1.0 and were absent in some other samples, and discarded if they were absent in BATG-1.0 but present in some other samples. To assess the impact of OrthoFinder potentially oversplitting genes, in a separate analysis, orthogroups containing at least one pair of genes with a coding sequence on the same strand overlapping by more than 50% were merged, and SV/Gene F1 scores recalculated. The results for this were qualitatively similar to the unmerged dataset, so the latter was used subsequently—for more discussion, see Supplementary Note 5 and Supplementary Fig. 26.

The complete set of putatively dispensable genes (i.e. before removing those with an F1 score <0.8) was functionally annotated. Protein sequences were searched against Araport 11 representative gene peptides (downloaded 14/09/2022), the NCBI SR database (downloaded 20/08/24), and the UniProt/SwissProt database (downloaded 24/07/24) using DIAMOND v2.1.9.163^169^ in blastp mode. Interproscan v5.69.101.0^170^ with default settings was used to search for protein domains. GO terms were assigned using eggNOG-mapper v2.1.12^171^ by using DIAMOND blastp to search the eggNOG database. GO enrichment was performed using topGO v2.62.0^172^ using annot = annFun.gene2GO, nodeSize = 10 and the exact Fisher test with Bonferroni correction. OrthoFinder was run with the complete set of potential pangenome protein sequences using the same species and parameters described above. Ortholog clusters across assemblies were used to compare genes identified as associated with low ADB damage across studies. BATG-0.5 gene models were used for Stocks et al.^42^ and Kelly et al.^85^, and Fexc_ONT_UoY gene models for Franco Ortega et al.^50^. Chano et al.^83^ and Ferrari et al.^82^ mapped RNA-seq data to FRAX_001_PL and identified differentially expressed genes using an annotation of that genome that is not publicly available. Sahraei et al.^84^ mapped reads to an earlier version of the BATG-0.5 assembly (BATG-0.4v3), providing FASTA sequences of their differentially expressed genes (DEGs)—reciprocal best BLAST hits of these sequences and BATG-0.5 gene models was performed in Kelly et al.^85^, with 81.6% of the DEG sequences having a reciprocal best BLAST hit in the BATG-0.5 annotation; the corresponding BATG-0.5 sequences were used as proxies for the sequences in Sahraei et al.^84^. Supplementary Table 2 of Ferrari et al.^82^ identified best hits of the *F. excelsior* gene models to those of other assemblies—83.2% of these matched genes in the *O. europea, S. indicum, S. lycopersicum* and *E. guttatus* assemblies included in our analysis, including 90.8% of significant genes from Supplementary Tables 3 and 4 of that study. Chano et al.^83^ used the same annotation files as Ferrari et al.^82^, allowing homologues of 79.3% of their DEGs to be identified.

### SNP and SV calling from short-read data from individuals and pools

To test the impact of using the pangenome on read mapping accuracy, short-read data from 42 samples separately whole genome sequenced in Stocks et al.^42^ (this number selected to reflect the average number of individuals in a pool in that study, see generation of in silico pools below) was mapped using vg giraffe^68^ to either (i) the full pangenome or (ii) the BATG-1.0 genome converted into pangenome format using vg construct. The resulting gam files were converted to BAM files in the BATG-1.0 coordinate system using vg surject. Mapping statistics were calculated using samtools. For each mapping approach, SNPs were called using bcftools v1.19 call, and calls merged across individuals. Genotypes with DP <5 or >50 were set to missing, then sites with >10% missing individuals or a MAF of <0.05 were removed.

For SV calling from these individuals, vg pack was used with parameter -Q 5, and SVs were called using vg call with the VCF used to construct the pangenome as a reference. For SNP calling, BAMs were produced using vg surject. SNPs were then called using bcftools v1.19 call. SNPs and SVs were merged across individuals, and calls with DP <5 or >50 were set to missing; then sites with >10% missing individuals or a MAF of <0.05 were removed. To produce similar numbers of SNPs and SVs for linkage analysis, 1% of SNPs were randomly sampled using vcflib v1.0.3^173^ function vcfrandomsample. SNP and SV data were combined using bcftools v1.19 concat option. Pairwise linkage between markers was calculated using plink v1.9.-170906^174^, with parameters –ld-window-kb 1000 and –ld-window 99999. Linkage decay with distance between SVs alone, between SNPs and SVs, and between SNPs alone were analysed separately.

To identify the accuracy of SNP and SV calling from poolseq data using the pangenome, read files from the 42 individually sequenced samples (which each contained approximately the same number of reads, equating to approximately 22× 1C genome coverage) were combined, and 10% of read pairs randomly subsampled with seqkit v2.8.0^175^. This corresponds to approximately 2.2× coverage per individual, the average per-individual coverage of the poolseq data in Stocks et al.^42^. SNPs and SVs were called from this in silico pool using the approaches above, but with sites being set to missing if they had a DP < 50 or DP > 200. Allele frequencies for SNPs/SVs were calculated as the number of reads supporting the alternate allele divided by the total number of reads. For the merged individually called SNPs/SVs, the correlation between the alternate allele frequency in the individually called samples and the estimated allele frequency for the in silico pool was tested with a linear model in R v4.3.1^176^.

### GWAS for low susceptibility to ash dieback from poolseq data

A poolseq dataset from Stocks et al.^42^ was used to perform a GWAS for low susceptibility to ADB. In brief, this data was generated as follows: leaf samples were taken from four FR mass screening trial sites in England (including the North Burlingham site where individuals were sampled for constructing the pangenome), from 6 year old ash trees from a total of 12 different seed sources (10 from UK NSZ, one from Ireland [IRL-DON] and one from Germany [DEU]), as well as a group from a breeding seedling orchard (sourced in the UK) planted by the Future Trees Trust (FTT-SO). For each of the 13 sources, a pool of healthy trees (no visible damage) and a pool of unhealthy trees (main stem dead or partially dead)—42 trees per pool on average—were sequenced with the Illumina HiSeq X platform in 150 bp paired-end reads. Tree health was assessed based on a tree health score designed to assess the overall level of damage from ADB—it should be noted that low susceptibility to ADB is a complex trait, which may not be associated with long-term tree health, and may be underpinned by multiple underlying mechanisms. Two seed sources had a second pair of pools, produced as biological replicates, for a total of 30 pools (excluding one technical replicate). See Stocks et al.^42^ for full details. The trimmed, paired short read data from ENA (accession ERA1732352, 'Submitted Files') was used.

Here, these reads were mapped to the pangenome and read depths for the reference or alternate paths were extracted from the VCF for each pool to produce estimates of SV allele frequencies, as described in the previous section. The SV allele frequencies were then merged across pools. For SNPs, the output of vg giraffe was converted to linear reference coordinates using vg surject, with PCR duplicates and reads with MAPQ < 20 removed using samtools v1.9^177^. SNPs were called from the resulting BAM files using samtools mpileup with -d 8000 and the mpileup2sync function from Popoolation2^71^ v1.201 with –min-qual 20 to produce a.sync file. Sites were removed if there was a third most frequent allele with a total depth higher than 15. The frequencies of major alleles for (i) SVs and (ii) a random 1% of SNPs were used to perform PCA using the R prcomp function.

To identify SV and SNP allele frequency changes associated with ADB damage, Cochran–Mantel–Haenszel (CMH) tests were performed using the cmh-test.pl function from Popoolation2, with parameters min_count 15, min_coverage 40 and max_coverage 200. For sites with a significant result (Bonferroni correction with *ɑ* =  0.05), the Woolf test was used from the vcd R package^178^ to test for significant deviation from homogeneity across groups using the alternate/reference read counts for each pool. To quantify heterogeneity in allele frequency shifts, Cochran’s Q^73^ and *I*^*2*^ ^81^ were calculated. Cochran’s Q was calculated based on Eq. 2^73^

2 $$Q={\sum }_{i=1}^{k}\,{w}_{i}\,{({x}_{i}-{x}_{w})}^{2}$$

with ${x}_{i}$ being the estimated effect size from pool *i*, ${x}_{w}$ the weighted mean effect size estimate across all *k* pairs of pools, and ${w}_{i}$ being the inverse of the variance estimated for each pair. For the effect sizes, we used natural logarithm transformed of individual odds ratios:

3 $${Effect}\,{size}={{\mathrm{ln}}}\frac{{ad}}{{bc}}$$

where *a* = alt reads in healthy, *b* = ref reads in healthy, *c* = alt reads in unhealthy, *d* = ref reads in unhealthy) as recommended in Woolf ^80^ and performed in other studies^179^.

The variance in this measure was estimated using the following Eq. 4^80^:

4 $${Variance}=\frac{1}{a}+\frac{1}{b}+\frac{1}{c}+\frac{1}{d}$$

The natural logarithm transformed overall CMH test odds ratio is used as ${x}_{w}$. These values were used to calculate *Q*. *I*^*2*^ was calculated using the following Eq. 5:

5 $${I}^{2}=\frac{Q-d}{Q}$$

where *d* is the degrees of freedom; in this case, the number of pairs of pools minus one. For SNPs with *p* < 0.05 from the CMH test after Bonferroni correction, SNPs with a standard deviation of depth of >15, indicative of potentially uncaptured SVs that may interfere with allele frequency estimation (Supplementary Note 1 and Supplementary Figs. 27–29), were removed, along with SNPs that showed deviation from homogenous odds ratios (violating an assumption of the CMH test)—namely, SNPs with either an *I*^*2*^ value of <0.25, or a Woolf test *p*-value of <0.05. 220 of the SNPs classified as significant after Bonferroni correction remained after this filtering. Genes from the pangenome that overlapped these SNPs, or were within 10 kb, were identified using bedtools. For those with annotated GO terms, enrichment was performed as described above for dispensable vs. indispensable genes. These genes formed 206 orthogroups (gene clusters) in the Orthofinder run from the pangenome annotation (see linear reference and pangenome annotation section above)—genes with sequence matches identified from the expression studies described previously were identified. To calculate the number of shared clusters expected by chance, clusters containing at least one sequence match for DEGs from Sahraei et al.^84^, Ferrari et al.^82^, Chano et al.^83^, Franco-Ortega et al.^50^ and Kelly et al.^85^ were identified (3655 clusters in total). For random resampling, for each of these clusters, the genome from which the sequence match was identified was recorded (i.e. Fexc_ONT_UoY for Franco-Ortega et al.^50^; BATG-0.5 for Kelly et al.^85^ etc.; see linear reference and pangenome annotation section above)—where a cluster contained sequence matches originating from more than one genome, BATG-0.5 was preferentially chosen; if the sequence matches were identified from different genomes, one of those genomes was chosen at random. This resulted in a total of 1541 genes from BATG-0.5, 1790 from *O. europaea*, 148 from Fexc_ONT_UoY, 139 from *S. indicum*, 31 from *E. guttatus,* and 6 from *S. lycopersicum* to be randomly resampled. For each genome, the respective number of genes was chosen at random (e.g. 1541 genes from BATG-0.5), for a total of 3655 genes—the clusters that each gene belonged to were identified, and the number of these clusters that also contained at least one of the 211 candidate genes from this study was identified. This was repeated 10,000 times. A separate set of 10,000 × 3655 genes was generated to identify how many contained dispensable genes.

An alternative statistical test for GWAS, a quasibinomial generalized linear model (qGLM), was run using the poolFreqDiffTest_QGLM.py script from the PoolFreqDiff repository^75^, using parameters -mincnt 15 -minc 40 -maxc 200 -n 45. Different methods of accounting for the effect of provenance were investigated; see Supplementary Note 2 for further details. Fixation index (*F*_st_) and Fisher’s exact test were also calculated in sliding windows for pairs of pools—see Supplementary Note 3 for further details.

### Reporting summary

Further information on research design is available in the Nature Portfolio Reporting Summary linked to this article.

## Supplementary information

Supplementary Information

Peer Review File

Description of Additional Supplementary Files

Supplementary Data 1

Supplementary Data 2

Supplementary Data 3

Supplementary Data 4

Supplementary Data 5

Supplementary Data 6

Supplementary Data 7

Supplementary Data 8

Supplementary Data 9

Supplementary Data 10

Supplementary Data 11

Supplementary Data 12

Supplementary Data 13

Supplementary Data 14

Reporting Summary

## Source data

Source Data

## Data Availability

The sequencing data generated in this study have been deposited in the NCBI under BioProjects PRJNA906474 for long-read DNA sequence data, PRJNA942609 for long-read RNA sequencing data, and PRJNA1390666 for short-read RNA sequencing data. Annotation files for the BATG-1.0 genome and pangenome are available on Zenodo [https://doi.org/10.5281/zenodo.21649671]. The BATG-1.0 sequence is available at the Ash Tree Genomes database (https://ashgenome.org/uk-reference-genome/). Source data are provided with this paper.

## Code availability

Code needed to reproduce the analysis is available at GitHub [https://github.com/danielwood1992/Ash_pangenome] and Zenodo [https://doi.org/10.5281/zenodo.21683898].
