# Supplementary material for: European ash pangenome reveals widespread structural variation and genetic basis of low ash dieback susceptibility
Source: Nat Commun. 2026 Sep 17;17:9693. doi: 10.1038/s41467-026-77194-9 (PMC13586383; doi:10.1038/s41467-026-77194-9)
Supplement: Supplementary file 1 [file 41467_2026_77194_MOESM18_ESM.pdf]

nature portfolio

Corresponding author(s): Daniel Wood, Laura Kelly

Last updated by author(s): Jul 31, 2026

Reporting Summary

Nature Portfolio wishes to improve the reproducibility of the work that we publish. This form provides structure for consistency and transparency in reporting. For further information on Nature Portfolio policies, see our Editorial Policies and the Editorial Policy Checklist.

Statistics

For all statistical analyses, confirm that the following items are present in the figure legend, table legend, main text, or Methods section.

n/a	Confirmed	
☐	☒	The exact sample size (*n*) for each experimental group/condition, given as a discrete number and unit of measurement
☐	☒	A statement on whether measurements were taken from distinct samples or whether the same sample was measured repeatedly
☐	☒	The statistical test(s) used AND whether they are one- or two-sided<br>*Only common tests should be described solely by name; describe more complex techniques in the Methods section.*
☒	☐	A description of all covariates tested
☐	☒	A description of any assumptions or corrections, such as tests of normality and adjustment for multiple comparisons
☐	☒	A full description of the statistical parameters including central tendency (e.g. means) or other basic estimates (e.g. regression coefficient) AND variation (e.g. standard deviation) or associated estimates of uncertainty (e.g. confidence intervals)
☐	☒	For null hypothesis testing, the test statistic (e.g. *F*, *t*, *r*) with confidence intervals, effect sizes, degrees of freedom and *P* value noted<br>*Give P values as exact values whenever suitable.*
☒	☐	For Bayesian analysis, information on the choice of priors and Markov chain Monte Carlo settings
☒	☐	For hierarchical and complex designs, identification of the appropriate level for tests and full reporting of outcomes
☒	☐	Estimates of effect sizes (e.g. Cohen's *d*, Pearson's *r*), indicating how they were calculated

Our web collection on statistics for biologists contains articles on many of the points above.

Software and code

Policy information about availability of computer code

Data collection	minKNOW v22.12.5, minKNOW v22.10.5
Data analysis	hifiasm v0.15.4-r347, HiRise, QUAST v5.2, BUSCO v5.5.4, blast+ v2.11.0, minimap2 v2.5, blobtools v4.0.7, Oatk v1.0, HMMER v3.1b2, GeSeq v2.03, Trimmomatic v0.39, Nanoplot v1.4.10, FastQC v0.11.9, multiQC v1.14, samtools v1.9, Clair3 v1.0.5, bcftools v1.16, Plink2 v2.00a3.3LM, Sniffles v2.0.7, cuteSV v2.0.3, shasta v0.11.1, Flye v.29.2-b1786, nextDenOvo v2.5.0, svim-asm-v1.02, SURVIVOR v1.0.7, bedtools v2.28.0, vg v1.64.0 (patch vg.2c61112bb461e3a8e6b976baefe464798ef3f359), RepeatModeler v2.0.1, RepeatMAsker v4.1.2, Telescope v0.0.9, STAR v2.7.10b, Nanofilt v2.8.0, BRAKER3 v3.03, BRAKER3 v3.08, OrthoFinder v2.5.5, DIAMOND v2.19.163, Interproscan v5.69.101.0, eggNOG-mapper v2.1.12, topGO v2.52.0, bcftools v1.19, plink v1.9.-170906, seqkit v2.8.0, R v4.3.1, Popoolation2 v1201, vcd v1.4-11, ggplot2v3.5.2, tidyr v1.3.0. Genometools v1.6.6, LTR_finder v1.0.7, LTR_Retriever v3.0.4, LVgs v1.00, Genomescope v2.1.0, Cutadapt v4.5, glnexus v1.4.1-0-g68e25e5n, bwa v0.7.17, rnaturalearth v1.2.0, vcflib v1.0.3 Scripts using this software to run the analysis are being made available at https://github.com/danielwood1992/Ash_pangenome.

For manuscripts utilizing custom algorithms or software that are central to the research but not yet described in published literature, software must be made available to editors and reviewers. We strongly encourage code deposition in a community repository (e.g. GitHub). See the Nature Portfolio guidelines for submitting code & software for further information.

Data

Policy information about availability of data

All manuscripts must include a data availability statement. This statement should provide the following information, where applicable:

- Accession codes, unique identifiers, or web links for publicly available datasets
- A description of any restrictions on data availability
- For clinical datasets or third party data, please ensure that the statement adheres to our policy

Source Data are provided with this paper. The sequencing data generated in this study have been deposited in the NCBI under BioProjects PRJNA906474 (long-read DNA sequence data; https://www.ncbi.nlm.nih.gov/bioproject/?term=PRJNA906474), PRJNA942609 (long-read RNA sequencing data; https://www.ncbi.nlm.nih.gov/bioproject/?term=PRJNA942609) and PRJNA1390666 (short-read RNA sequencing data; https://www.ncbi.nlm.nih.gov/bioproject/?term=PRJNA1390666). Annotation files for the BATG-1.0 genome and pangenome are available on Zenodo https://doi.org/10.5281/zenodo.21649671.
The BATG-1.0 sequence is available at https://ashgenome.org/uk-reference-genome/.

Research involving human participants, their data, or biological material

Policy information about studies with human participants or human data. See also policy information about sex, gender (identity/presentation), and sexual orientation and race, ethnicity and racism.

Reporting on sex and gender	Non-human study.
Reporting on race, ethnicity, or other socially relevant groupings	Non-human study.
Population characteristics	Non-human study.
Recruitment	Non-human study.
Ethics oversight	Non-human study.

Note that full information on the approval of the study protocol must also be provided in the manuscript.

Field-specific reporting

Please select the one below that is the best fit for your research. If you are not sure, read the appropriate sections before making your selection.

☐ Life sciences ☐ Behavioural & social sciences ☒ Ecological, evolutionary & environmental sciences

For a reference copy of the document with all sections, see nature.com/documents/nr-reporting-summary-flat.pdf

Life sciences study design

All studies must disclose on these points even when the disclosure is negative.

Sample size	*Describe how sample size was determined, detailing any statistical methods used to predetermine sample size OR if no sample-size calculation was performed, describe how sample sizes were chosen and provide a rationale for why these sample sizes are sufficient.*
Data exclusions	*Describe any data exclusions. If no data were excluded from the analyses, state so OR if data were excluded, describe the exclusions and the rationale behind them, indicating whether exclusion criteria were pre-established.*
Replication	*Describe the measures taken to verify the reproducibility of the experimental findings. If all attempts at replication were successful, confirm this OR if there are any findings that were not replicated or cannot be reproduced, note this and describe why.*
Randomization	*Describe how samples/organisms/participants were allocated into experimental groups. If allocation was not random, describe how covariates were controlled OR if this is not relevant to your study, explain why.*
Blinding	*Describe whether the investigators were blinded to group allocation during data collection and/or analysis. If blinding was not possible, describe why OR explain why blinding was not relevant to your study.*

Behavioural & social sciences study design

All studies must disclose on these points even when the disclosure is negative.

Study description	*Briefly describe the study type including whether data are quantitative, qualitative, or mixed-methods (e.g. qualitative cross-sectional, quantitative experimental, mixed-methods case study).*

Research sample	*State the research sample (e.g. Harvard university undergraduates, villagers in rural India) and provide relevant demographic information (e.g. age, sex) and indicate whether the sample is representative. Provide a rationale for the study sample chosen. For studies involving existing datasets, please describe the dataset and source.*
Sampling strategy	*Describe the sampling procedure (e.g. random, snowball, stratified, convenience). Describe the statistical methods that were used to predetermine sample size OR if no sample-size calculation was performed, describe how sample sizes were chosen and provide a rationale for why these sample sizes are sufficient. For qualitative data, please indicate whether data saturation was considered, and what criteria were used to decide that no further sampling was needed.*
Data collection	*Provide details about the data collection procedure, including the instruments or devices used to record the data (e.g. pen and paper, computer, eye tracker, video or audio equipment) whether anyone was present besides the participant(s) and the researcher, and whether the researcher was blind to experimental condition and/or the study hypothesis during data collection.*
Timing	*Indicate the start and stop dates of data collection. If there is a gap between collection periods, state the dates for each sample cohort.*
Data exclusions	*If no data were excluded from the analyses, state so OR if data were excluded, provide the exact number of exclusions and the rationale behind them, indicating whether exclusion criteria were pre-established.*
Non-participation	*State how many participants dropped out/declined participation and the reason(s) given OR provide response rate OR state that no participants dropped out/declined participation.*
Randomization	*If participants were not allocated into experimental groups, state so OR describe how participants were allocated to groups, and if allocation was not random, describe how covariates were controlled.*

Ecological, evolutionary & environmental sciences study design

All studies must disclose on these points even when the disclosure is negative.

Study description	This study generated a pangenome for European ash (Fraxinus excelsior) - this involved sequencing the DNA of fifty samples of F. excelsior. For 36 of these individuals, RNA was extracted from the same leaf samples and short read cDNA sequenced. RNA to annotate the pangenome was extracted from two tissue types from one of the individuals, combined with RNA-seq data generated by Sollars et al. 2017 (https://doi.org/10.1038/nature20786). The pangenome was used as a reference to reanalyse short-read data generated in Stocks et al. 2019 (https://doi.org/10.1038/s41559-019-1036-6).

Research sample	All individuals sampled were European ash (Fraxinus excelsior). Sex phenotypes (expression of which can vary between years, and form a continuum) were not specifically recorded. For the chromosome-level reference assembly and one of the long-read sequencing samples, a potted graft at Kew was used. This was initially believed to have been a clone of the same individual as BATG-0.5, so was chosen to maximise compatibility with existing datasets from this genotype. Genotyping revealed that this individual is not a clone of BATG-0.5; the origin of the sample cannot be ascertained with certainty.<br><br>For the remaining 49 samples, one individual from each of the 13 NSZ used in the Forest Research (FR) mass screening field trial at North Burlingham, Norfolk, UK (Site 16) was sequenced, with the exception of NSZ-204 506 where two were sequenced, as individually sequenced data for this provenance were available from Stocks et al. 2019. The North Burlingham site was planted in 2013 with two-year-old saplings that were grown for non-research purposes in commercial nurseries from seed sources from 15 different provenances. The site contained four complete replicate plots for each seed source, with 5,000 trees per ha (a spacing of 1 m× 2 m). At the time of sampling, the site had high H. fraxineus inoculum pressure from naturally established infections, and ADB damage was severe throughout. In 2022 (when trees were eleven years old) individuals were sampled that appeared relatively healthy within each plot, and scored between 3 and 5 on the damage scale of Pliura et al. (we excluded the score of 0, which indicates trees that died soon after planting) to increase the chances of capturing alleles for low ADB susceptibility. Three wild trees of unknown age from seed zones in the UK not covered by the FR trial populations (NSZ-305, NSZ-518 406, NSZ-402) were also included to further capture UK diversity. To increase the likelihood of including more of the range-wide diversity of structural variation in F. excelsior, one individual from each of 29 mostly mainland European provenances was sampled from the Realising Ash's Potential (RAP) trial (established in 2005), as well as two from provenances in the FRAXIGEN trial (established in 2004), both at Paradise Wood, Earth Trust, Oxfordshire. Full details for each sample are available in Supplementary Table 3.<br><br>For RNA extraction for the pangenome: Leaf tissue was collected from the potted graft of an individual of unknown provenance growing at the Royal Botanic Gardens, Kew in May 2022 and flash frozen in liquid nitrogen. For cambium samples, twigs were sampled in January 2023 and the layer of tissue immediately below the bark isolated using a fresh razor blade before flash-freezing in liquid nitrogen. For short-read RNA sequencing, RNA was extracted from leaf tissue gathered for the long-read DNA sequencing was used, apart from a single sample (PG5) from the North Burlingham mass screening trial. This individual was revisited in May 2024, and leaf tissue stored in RNALater, and RNA extracted along with the other samples.<br><br>For short-read data from Stocks et al (2019): leaf samples were taken from four FR mass screening trial sites in England (including the North Burlingham site where individuals were sampled for constructing the pangenome), from six year old ash trees from a total of 12 different seed sources (10 from UK NSZ, one from Ireland [IRL-DON] and one from Germany [DEU]), as well as a group from a breeding seedling orchard planted by the Future Trees Trust (FTT-SO). Individuals were classified as healthy or unhealthy based on the scale of Pliura et al. (2017) - trees scoring 7 or above were considered healthy, and those with a score of 4-5 were considered as unhealthy. For each of the 13 sources, a pool of healthy trees and a pool of unhealthy trees (42 trees per pool on average) were sequenced. Two seed sources had a second pair of pools, produced as biological replicates, for a total of 30 pools (excluding one technical replicate). NSZ-204 had an additional 150 samples sequenced individually in this study, half of which were classified as healthy, and half as unhealthy.
Sampling strategy	For genomic DNA, leaf tissue was collected from each of the trees as described above. Leaves were stored in plastic bags until they could be flash frozen using dry ice (apart from the sample sent to Cantata Bio, that was flash frozen in liquid nitrogen). Sample size was not determined statistically. Most previously published pangenome studies at time of sampling included 25-50 individuals, and showed a plateau in the number of additional structural variants detected in the final sample, indicating these numbers were generally sufficient to capture most common segregating structural variants. We therefore selected fifty as the number of individuals from which to contruct the pangenome, based on the expectation that this would provide a reasonable probability of capturing the most common structural variants in Fraxinus excelsior. Controlled experiments were not performed in this study.
Data collection	All samples were collected by Daniel Wood, with help from others in some cases. Information from Forest Research (FR) was provided in order to correctly locate blocks of trees from particular provenances within the mass screening trial/Earth Trust sites. If multiple suitable trees were present in a block, the tree closest to the centre of the block was sampled to minimize the possibility of errors in determining the block boundary. Ash dieback disease scores were scored by Daniel Wood by visual inspection of the trees, scored on the scale of Pliura et al. (2011). Sample ID/names were recorded both in a notebook, and on the bags the samples were collected in. Laura Kelly returned to one of the FR research trials in May 2024 to collect leaf tissue from a single remaining sample, using co-ordinates and pictures from the original sampling to recover the original tree.
Timing and spatial scale	Samples for genomic DNA extraction were collected in May 2022. RNA-seq data samples were collected in May 2022 and January 2023. Sampling was performed entirely within the United Kingdom, but provenances of the sampled trees from the RAP trial spanned a substantial proportion of the native range of Fraxinus excelsior - sampling locations and the native range of F. excelsior are plotted in Figure 1. Tissue for RNA-seq of PG5 was collected in May 2024. RNA for short-read RNA-seq was extracted in November 2025.
Data exclusions	No samples were excluded from the analysis.
Reproducibility	All samples were used to construct the highest possible quality pangenome reference, so no repeated samples were taken to identify the reproducibility of constructing the pangenome itself. Examination of the number of unique structural variants added by the final sample in the analysis indicated that more samples would be required to fully capture all structural variants segregating range-wide in Fraxinus excelsior, so replication of this study may identify additional structural variants to those we captured.
Randomization	Samples used to construct the pangenome were not allocated into groups in this study - a pangenome combines information from all samples. Within the Stocks et al. (2019) study, pairs of healthy and unhealthy pools of individuals were used, with the same numbers of samples from the same proportion of mass-screening site locations used in the healthy and unhealthy pools; this controls for confounding factors relating to mass-screening site locations.

Blinding	Blinding was not relevant to this study - ash dieback damage status for individuals included in the pangenome was collected, but this did not impact the outcome of the study (for the mass screening trials, the healthiest individuals in a block were chosen for sequencing, but this did not depend on the health score identified).

Did the study involve field work? [x] Yes [ ] No

Field work, collection and transport

Field conditions	Samples for DNA sequencing were collected in May of 2022. The DNA sequence of long-lived individual trees is not determined by field conditions, so these were not recorded. Field conditions for collecting the RNA-seq data were also not recorded (beyond sampling dates) - these were intended to capture transcripts expressed in these tissues to aid in annotation in their totality, rather than comparing between the tissues, for which controlled conditions and replicates would be required. A single sample for short-read RNA-seq (individual PG5) was collected in May 2024 from one of the original trial sites.
Location	Samples from the graft used to generate BATG-1.0 were collected at the location of this plant at the Royal Botanic Gardens, Kew at 51.482, -0.288. The Forest Research mass screening was located on a privately owned site near North Burlingham in Norfolk, UK (within 2 miles of 52.637, 1.494) - note that this site was transferred from Forest Research's management and destroyed subsequent to the samples for this study being collected. The RAP trial at Earth Trust is located at 51.638, -1.200. Sites for the individually sampled wild trees are detailed in Supplementary Table 3.
Access & import/export	Sampling sites were accessed with permission of the landowners and land managers at each site. None were located in sites requiring specific permits. Sampling and most sequencing was performed within the United Kingdom. An import permit for the leaf sample sent to Cantata Bio in the United States of America was obtained by the company.
Disturbance	Before and after sampling, boots were manually cleaned with Propellar or equivalent to reduce the chances of spreading microorganisms to/from the sampling sites. Trees were accessed via established routes/paths. Damage to trees from taking leaf samples is minimal.

Reporting for specific materials, systems and methods

We require information from authors about some types of materials, experimental systems and methods used in many studies. Here, indicate whether each material, system or method listed is relevant to your study. If you are not sure if a list item applies to your research, read the appropriate section before selecting a response.

Materials & experimental systems

n/a	Involved in the study
[x]	[ ] Antibodies
[x]	[ ] Eukaryotic cell lines
[x]	[ ] Palaeontology and archaeology
[x]	[ ] Animals and other organisms
[x]	[ ] Clinical data
[x]	[ ] Dual use research of concern
[ ]	[x] Plants

Methods

n/a	Involved in the study
[x]	[ ] ChIP-seq
[x]	[ ] Flow cytometry
[x]	[ ] MRI-based neuroimaging

Antibodies

Antibodies used	*Describe all antibodies used in the study; as applicable, provide supplier name, catalog number, clone name, and lot number.*
Validation	*Describe the validation of each primary antibody for the species and application, noting any validation statements on the manufacturer's website, relevant citations, antibody profiles in online databases, or data provided in the manuscript.*

Eukaryotic cell lines

Policy information about cell lines and Sex and Gender in Research

Cell line source(s)	*State the source of each cell line used and the sex of all primary cell lines and cells derived from human participants or vertebrate models.*
Authentication	*Describe the authentication procedures for each cell line used OR declare that none of the cell lines used were authenticated.*
Mycoplasma contamination	*Confirm that all cell lines tested negative for mycoplasma contamination OR describe the results of the testing for mycoplasma contamination OR declare that the cell lines were not tested for mycoplasma contamination.*
Commonly misidentified lines (See ICLAC register)	*Name any commonly misidentified cell lines used in the study and provide a rationale for their use.*

Palaeontology and Archaeology

Specimen provenance	*Provide provenance information for specimens and describe permits that were obtained for the work (including the name of the issuing authority, the date of issue, and any identifying information). Permits should encompass collection and, where applicable, export.*
Specimen deposition	*Indicate where the specimens have been deposited to permit free access by other researchers.*
Dating methods	*If new dates are provided, describe how they were obtained (e.g. collection, storage, sample pretreatment and measurement), where they were obtained (i.e. lab name), the calibration program and the protocol for quality assurance OR state that no new dates are provided.*

☐ Tick this box to confirm that the raw and calibrated dates are available in the paper or in Supplementary Information.

Ethics oversight	*Identify the organization(s) that approved or provided guidance on the study protocol, OR state that no ethical approval or guidance was required and explain why not.*

Note that full information on the approval of the study protocol must also be provided in the manuscript.

Animals and other research organisms

Policy information about studies involving animals; ARRIVE guidelines recommended for reporting animal research, and Sex and Gender in Research

Laboratory animals	*For laboratory animals, report species, strain and age OR state that the study did not involve laboratory animals.*
Wild animals	*Provide details on animals observed in or captured in the field; report species and age where possible. Describe how animals were caught and transported and what happened to captive animals after the study (if killed, explain why and describe method; if released, say where and when) OR state that the study did not involve wild animals.*
Reporting on sex	*Indicate if findings apply to only one sex; describe whether sex was considered in study design, methods used for assigning sex. Provide data disaggregated for sex where this information has been collected in the source data as appropriate; provide overall numbers in this Reporting Summary. Please state if this information has not been collected. Report sex-based analyses where performed, justify reasons for lack of sex-based analysis.*
Field-collected samples	*For laboratory work with field-collected samples, describe all relevant parameters such as housing, maintenance, temperature, photoperiod and end-of-experiment protocol OR state that the study did not involve samples collected from the field.*
Ethics oversight	*Identify the organization(s) that approved or provided guidance on the study protocol, OR state that no ethical approval or guidance was required and explain why not.*

Note that full information on the approval of the study protocol must also be provided in the manuscript.

Clinical data

Policy information about clinical studies

All manuscripts should comply with the ICMJE guidelines for publication of clinical research and a completed CONSORT checklist must be included with all submissions.

Clinical trial registration	*Provide the trial registration number from ClinicalTrials.gov or an equivalent agency.*
Study protocol	*Note where the full trial protocol can be accessed OR if not available, explain why.*
Data collection	*Describe the settings and locales of data collection, noting the time periods of recruitment and data collection.*
Outcomes	*Describe how you pre-defined primary and secondary outcome measures and how you assessed these measures.*

Dual use research of concern

Policy information about dual use research of concern

Hazards

Could the accidental, deliberate or reckless misuse of agents or technologies generated in the work, or the application of information presented in the manuscript, pose a threat to:

No | Yes
- [x] No [ ] Yes — Public health
- [x] No [ ] Yes — National security
- [x] No [ ] Yes — Crops and/or livestock
- [x] No [ ] Yes — Ecosystems
- [x] No [ ] Yes — Any other significant area

Experiments of concern

Does the work involve any of these experiments of concern:

No | Yes
- [x] No [ ] Yes — Demonstrate how to render a vaccine ineffective
- [x] No [ ] Yes — Confer resistance to therapeutically useful antibiotics or antiviral agents
- [x] No [ ] Yes — Enhance the virulence of a pathogen or render a nonpathogen virulent
- [x] No [ ] Yes — Increase transmissibility of a pathogen
- [x] No [ ] Yes — Alter the host range of a pathogen
- [x] No [ ] Yes — Enable evasion of diagnostic/detection modalities
- [x] No [ ] Yes — Enable the weaponization of a biological agent or toxin
- [x] No [ ] Yes — Any other potentially harmful combination of experiments and agents

Plants

Seed stocks	No seed stocks were used in this study. Plant collection procedures, locations and dates are outlined in the field-specific reporting section above.
Novel plant genotypes	No novel plant genotypes were produced.
Authentication	No authentication procedures for seed stocks/genotypes were used in this study. Fraxinus excelsior has low levels of population structure across its range - we are not aware of markers that could reliably confirm the provenance of a particular genotype.

ChIP-seq

Data deposition

- [ ] Confirm that both raw and final processed data have been deposited in a public database such as GEO.
- [ ] Confirm that you have deposited or provided access to graph files (e.g. BED files) for the called peaks.

Data access links<br>*May remain private before publication.*	*For "Initial submission" or "Revised version" documents, provide reviewer access links. For your "Final submission" document, provide a link to the deposited data.*
Files in database submission	*Provide a list of all files available in the database submission.*
Genome browser session<br>(e.g. UCSC)	*Provide a link to an anonymized genome browser session for "Initial submission" and "Revised version" documents only, to enable peer review. Write "no longer applicable" for "Final submission" documents.*

Methodology

Replicates	*Describe the experimental replicates, specifying number, type and replicate agreement.*
Sequencing depth	*Describe the sequencing depth for each experiment, providing the total number of reads, uniquely mapped reads, length of reads and whether they were paired- or single-end.*
Antibodies	*Describe the antibodies used for the ChIP-seq experiments; as applicable, provide supplier name, catalog number, clone name, and lot number.*
Peak calling parameters	*Specify the command line program and parameters used for read mapping and peak calling, including the ChIP, control and index files used.*
Data quality	*Describe the methods used to ensure data quality in full detail, including how many peaks are at FDR 5% and above 5-fold enrichment.*

Software | *Describe the software used to collect and analyze the ChIP-seq data. For custom code that has been deposited into a community repository, provide accession details.*

Flow Cytometry

Plots

Confirm that:

☐ The axis labels state the marker and fluorochrome used (e.g. CD4-FITC).

☐ The axis scales are clearly visible. Include numbers along axes only for bottom left plot of group (a 'group' is an analysis of identical markers).

☐ All plots are contour plots with outliers or pseudocolor plots.

☐ A numerical value for number of cells or percentage (with statistics) is provided.

Methodology

Sample preparation | *Describe the sample preparation, detailing the biological source of the cells and any tissue processing steps used.*

Instrument | *Identify the instrument used for data collection, specifying make and model number.*

Software | *Describe the software used to collect and analyze the flow cytometry data. For custom code that has been deposited into a community repository, provide accession details.*

Cell population abundance | *Describe the abundance of the relevant cell populations within post-sort fractions, providing details on the purity of the samples and how it was determined.*

Gating strategy | *Describe the gating strategy used for all relevant experiments, specifying the preliminary FSC/SSC gates of the starting cell population, indicating where boundaries between "positive" and "negative" staining cell populations are defined.*

☐ Tick this box to confirm that a figure exemplifying the gating strategy is provided in the Supplementary Information.

Magnetic resonance imaging

Experimental design

Design type | *Indicate task or resting state; event-related or block design.*

Design specifications | *Specify the number of blocks, trials or experimental units per session and/or subject, and specify the length of each trial or block (if trials are blocked) and interval between trials.*

Behavioral performance measures | *State number and/or type of variables recorded (e.g. correct button press, response time) and what statistics were used to establish that the subjects were performing the task as expected (e.g. mean, range, and/or standard deviation across subjects).*

Acquisition

Imaging type(s) | *Specify: functional, structural, diffusion, perfusion.*

Field strength | *Specify in Tesla*

Sequence & imaging parameters | *Specify the pulse sequence type (gradient echo, spin echo, etc.), imaging type (EPI, spiral, etc.), field of view, matrix size, slice thickness, orientation and TE/TR/flip angle.*

Area of acquisition | *State whether a whole brain scan was used OR define the area of acquisition, describing how the region was determined.*

Diffusion MRI ☐ Used ☐ Not used

Preprocessing

Preprocessing software | *Provide detail on software version and revision number and on specific parameters (model/functions, brain extraction, segmentation, smoothing kernel size, etc.).*

Normalization | *If data were normalized/standardized, describe the approach(es): specify linear or non-linear and define image types used for transformation OR indicate that data were not normalized and explain rationale for lack of normalization.*

Normalization template | *Describe the template used for normalization/transformation, specifying subject space or group standardized space (e.g. original Talairach, MNI305, ICBM152) OR indicate that the data were not normalized.*

Noise and artifact removal | *Describe your procedure(s) for artifact and structured noise removal, specifying motion parameters, tissue signals and physiological signals (heart rate, respiration).*

Volume censoring | *Define your software and/or method and criteria for volume censoring, and state the extent of such censoring.*

Statistical modeling & inference

Model type and settings | *Specify type (mass univariate, multivariate, RSA, predictive, etc.) and describe essential details of the model at the first and second levels (e.g. fixed, random or mixed effects; drift or auto-correlation).*

Effect(s) tested | *Define precise effect in terms of the task or stimulus conditions instead of psychological concepts and indicate whether ANOVA or factorial designs were used.*

Specify type of analysis: ☐ Whole brain ☐ ROI-based ☐ Both

Statistic type for inference | *Specify voxel-wise or cluster-wise and report all relevant parameters for cluster-wise methods.*

(See Eklund et al. 2016)

Correction | *Describe the type of correction and how it is obtained for multiple comparisons (e.g. FWE, FDR, permutation or Monte Carlo).*

Models & analysis

n/a	Involved in the study
☐	☐ Functional and/or effective connectivity
☐	☐ Graph analysis
☐	☐ Multivariate modeling or predictive analysis

Functional and/or effective connectivity | *Report the measures of dependence used and the model details (e.g. Pearson correlation, partial correlation, mutual information).*

Graph analysis | *Report the dependent variable and connectivity measure, specifying weighted graph or binarized graph, subject- or group-level, and the global and/or node summaries used (e.g. clustering coefficient, efficiency, etc.).*

Multivariate modeling and predictive analysis | *Specify independent variables, features extraction and dimension reduction, model, training and evaluation metrics.*
