# Supplementary material for: European ash pangenome reveals widespread structural variation and genetic basis of low ash dieback susceptibility
Source: Nat Commun. 2026 Sep 17;17:9693. doi: 10.1038/s41467-026-77194-9 (PMC13586383; doi:10.1038/s41467-026-77194-9)
Supplement: Supplementary file 2 [file 41467_2026_77194_MOESM1_ESM.pdf]

European ash pangenome reveals widespread structural variation and genetic basis of low ash dieback susceptibility

Wood *et al.*

Supplementary Note 1. Rare SVs distort allele frequency estimates, leading to low CMH test *p*-values

We investigated whether SNPs with highly significant CMH test *p*-values may be strongly differentiated in subsets of pools, despite the limited evidence for SNPs associated with ADB low susceptibility that are widely shared across the seed sources. We first considered the 92 SNPs with a *p*-value of 1×10^{-13} (the significance threshold used for the CMH test in Stocks *et al.* [1] - 55/92 of these were in a single 1.3kb region on chromosome 15 (Supplementary Figure 27). The high density of CMH outlier SNPs, combined with raised depths in a subset of pools (Supplementary Figure 28), led us to suspect this region represented a structural variant (SV) not captured in our pangenome, but which may be strongly associated with low ADB susceptibility in a subset of provenances. However, mapping 692 individually sequenced samples to this region using bwa v0.7.17[2] (150 individually sequenced samples from Stocks *et al.* [1], displayed in Supplementary Figure 29B; 542 individuals from Metheringham *et al.* [3], not displayed) identified two samples - one from Stocks *et al.*, one from Metheringham *et al.* - with large increases in depth (29-33X that of the flanking region) compared to other samples (Supplementary Figure 29B), which was recapitulated when these two samples were mapped to the full pangenome (Supplementary Figure 29C, D). This suggests the presence in some individuals of a large or high copy number SV with high similarity to the 1.3kb region identified in our GWAS. A single individual with this large or high-copy SV could result in a pool with approximately the observed depth increase, giving a highly distorted estimate of SNP allele frequency. The standard deviation of depth, and to a lesser extent the depth of healthy pools relative to unhealthy pools in the top 92 SNPs often exceeded the genome-wide average of these statistics (Supplementary Figure 19) - this was less frequent in the 866 SNPs as a whole that are significant after Bonferroni correction, suggesting this issue is likely to be rare and disproportionately impacting the SNPs with the most extreme *p*-values from the CMH test (Supplementary Figure 19).

Supplementary Note 2. GWAS analysis

Wiberg *et al.* [4] identified a quasibinomial test as an alternative for detecting consistent shifts in allele frequencies. Using this test found no significant SNPs (either with Bonferroni correction with $\alpha = 0.05$, or $p < 10^{-13}$) associated with low susceptibility to ADB, with the lowest *p*-value being 4.4×10^{-7}. There was some evidence of *p*-value deflation (SV lambda = 0.58; SNP lambda = 0.62; Supplementary Figure 20), suggesting the test could be overly conservative. The top 1000

SNPs showed more consistent shifts than for CMH SNPs with $p < 10^{-13}$ (Figure 5), but also showed mean allele frequency shift magnitudes that were very similar to a randomly selected background set of SNPs (Supplementary Figure 20B-F). This suggests that the most consistent allele frequency shifts across the pools have a very small average allele frequency shift; in a large enough selection of SNPs, some of these sites would be expected to occur by chance. The qGLM test using the poolFreqDiffTest_QGLM.py script from the PoolFreqDiff repository[4] by default does not include an effect for pool stratification – including a fixed effect for provenance resulted in substantial *p*-value inflation (Supplementary Figure 21). Including fixed effects for the first two principal components of the PCA from poolseq SNP allele frequencies (Supplementary Figure 15C) resulted in more modest *p*-value inflation (Supplementary Figure 21B), identifying a single significant SNP with Bonferroni correction with $\alpha = 0.05$, but none with $p < 10^{-13}$. Using a binomial GLM with a random effect of provenance, or a binomial GLM and a random effect of provenance and observation (the latter allowing for more overdispersion) resulted in extreme *p*-value inflation (Supplementary Figure 21C and 21D).

Supplementary Note 3. Per pool differentiation estimates

To more explicitly test allele frequency shifts within subsets of provenances, we calculated i) Fisher's exact test using the fisher-test.pl function in Popoolation2, and ii) F_{st}, in 500bp/100bp sliding windows using the fst-sliding.pl function in Popoolation2, for each pair of healthy vs. unhealthy pools. We then looked at windows shared across multiple pairs of pools, either by multiple pairs sharing i) a 500bp/100bp sliding window containing a SNP passing a significance threshold for the Fisher's exact test SNPs ($p < 0.05$ after Bonferroni correction for the number of sliding windows), ii) a window in the top 1% of F_{st} values for each pair of pools. It should be noted that absolute F_{st} may lack power to detect small-effect SNPs diverging over very short timeframes, but regions in the top 1% of values may nevertheless be candidates for differentiated regions.

A total of 168,378 windows contained a significant Fisher's exact test SNP in at least one pair of pools - however, only 3,937 of these were shared across more than one pair. For both pairs of biological replicate pools from Stocks *et al.*[1], the overlap was very low - only 0.2% of all windows identified across the two pairs of biological replicates were identified as outliers in both pairs. Whilst 101,334 windows were found to have F_{st} values in the top 1% of values in more than one pool, only 5.1% and 4.9% of the outliers for replicates for NSZ-106 and NSZ-107 that were

common to more than one pool, were shared with their respective biological replicate. The clustering of biological replicates in a PCA of SNP allele frequency (Supplementary Figure 15) indicates that in aggregate, these data clearly contain biological signal - but high confidence allele frequency shifts at individual sites are difficult to distinguish from stochastic fluctuations in individual read counts inherent to poolseq data. We therefore do not believe it is possible to detect more geographically restricted genetic variation associated with low ADB susceptibility using the present dataset.

Supplementary Note 4. Effect of using the BATG-0.5 reference genome

To compare how the results of the SNP-based CMH test would be affected by using the pangenome, the GWAS was repeated using the BATG-0.5 reference genome, which was the first published reference assembly for *F. excelsior* and has been used for previous GWAS. Reads from the poolseq files were mapped to the assembly using bwa 0.7.17. Samtools v1.9[5] was used to remove PCR duplicates and reads with MAPQ < 20. From these bam files, the same approach as for the pangenome based analysis was used to create .sync files and perform CMH tests. To compare these results with those generated from the pangenome, a chain file between BATG-0.5 and BATG-1.0 was created using nf-lo v1.8.0[6] using settings –distance medium –aligner minimap2. Using the –annotation option, the positions of the significant SNPs from the BATG-0.5 GWAS were estimated in the new assembly. This resulted in 116 SNPs with $p < 10^{-13}$. A liftover of these 116 SNPs from BATG-0.5 identified 91 of the 92 sites with $p < 10^{-13}$ detected via the pangenome-based GWAS. Reads mapping to significant sites in the BATG-0.5 reference genome were mapped to the pangenome; these mapped to 82 of the 92 significant sites identified from the GWAS using the pangenome. Similarly, reads mapping to significant sites in the pangenome were mapped to BATG-0.5, covering 77 of the 116 significant sites. Only 17 SNPs identified as significant from mapping to the pangenome corresponded to the 192 SNPs in BATG-0.5 found as significant in Stocks *et al.*[1], as identified by the liftover, despite an equivalent position in BATG-1.0 being identified for all 192 sites from BATG-0.5. The above analysis indicates this is unlikely to be due to the different reference genomes used but is instead likely the result of different choices of parameters in the two studies. For example, in this study, sites were removed if a pool had a depth > 200, whereas in Stocks *et al.*[1] this value was 3,000. By contrast, if sites overlapped with a repeat sequence in Stocks *et al.*[1] they were removed, whereas in this study they were retained.

Supplementary Note 5. Pangenome annotation clustering

To avoid missing genes due to stochasticity in the BRAKER3 pipeline, we ran 10 runs of the pipeline using short read RNA-seq data, and 10 runs using the long read RNA-seq data. Across each run, genes were clustered using OrthoFinder[8], with each non-overlapping orthogroup cluster treated as a separate gene. 38,766 non-overlapping clusters were initially detected, with 35,524 containing exactly 20 sequences (i.e. annotated in every run), or 10 sequences (i.e. annotated in every long or short read run). This corresponded well with the number of genes in assemblies such as BATG-0.5[9]. However, 91 pairs of genes had the same start/end positions in the genome, with manual inspection identifying short deletions of particular exons likely being responsible, suggesting in some cases OrthoFinder was likely over-splitting genes in some cases. Any approach to clustering genes across different annotations has an inherent chance of clustering sequences together that should be separate genes, and also clustering sequences as two separate genes when they should be the same gene. To investigate the potential impact of this clustering, we identified cases where a coding sequence overlapped more than 50% with coding sequence from another gene on the same strand, and kept the longest protein coding gene. This resulted in a reduction of the number of genes from 38,766 (untrimmed BATG-1.0 gene set) to 37,983 (trimmed BATG-1.0 gene set). Whilst this potentially discards some real genes, we were concerned that downstream clustering of genes in the pangenome with oversplit genes may have led to oversplitting of pangenome genes as well, with potential downstream consequences on our estimation of the reliability of our dispensable gene assignments.

Running OrthoFinder on proteins sequences from the pangenome samples, along with both these gene sets, resulted in very similar numbers of genes - 48,324 non-overlapping Orthogroups when using the BATG-1.0 untrimmed gene set, and 48,356 when using the trimmed gene set. Further merging these gene sets, if any two genes on the same strand overlapped in their coding sequence by more than 50%, resulted in 45,508 genes when using the untrimmed BATG-1.0 sequences, and 45,114 genes when using the trimmed BATG-1.0 sequences. In some cases where the pangenome genes were merged, genes that did not overlap at all were merged, as they were connected by a series of overlapping intermediate genes – with 100 annotation files, the risk of erroneous gene fusions potentially increases.

We ran the analysis to detect the relationship between SVs and gene presence for each of the four combinations of analysis (untrimmed or trimmed BATG-1.0 gene set, merged or unmerged pangenome gene set) – the overall proportions of dispensable genes, and the generally low concordance of variable genes with SVs, was very similar for each combination of approaches (Supplementary Figure 26), although proportion of dispensable genes was slightly higher using the unmerged pangenome set (8.7% and 8.6% for the trimmed and untrimmed BATG-1.0 sequences, respectively) compared to the merged pangenome gene set (7.2% and 7.2% for the trimmed and untrimmed BATG-1.0 sequences, respectively). We judged that the trimmed BATG-1.0 and untrimmed pangenome datasets appeared to strike the best balance between avoiding undersplitting and oversplitting. Regardless of the exact approach of merging genes across annotation runs, the overall conclusion that many putatively dispensable genes are not reliably associated with sequence differences caused by SVs, remains the same.

Supplementary Method 1. RNA extraction protocol

High integrity RNA extraction from fresh/flash frozen *F. excelsior* leaves and cambium, modified from the Qiagen RNeasy Plant Mini Kit Protocol. Outputs; 10-20ug RNA, >100ng/µL, 260/280 and 260/230 >1.8, RIN > 7, ND/QT ratio < 2. <2 hours for 2 samples.

Materials

- 25-30mg fresh / flash frozen leaf tissue
- QIAGEN RNeasy Plant Mini Kit
- B-mercaptoethanol
- Qiagen RNase-free DNase Set
- Liquid Nitrogen
- Mortar and pestle

Method

1. Before starting, prepare buffers RLT and RPE as outlined in the Rneasy Plant Mini Kit quick start protocol. Note that each sample will require 2x the volume of buffers/reagents as outlined in the kit handbook. Prepare Dnase aliquots as outlined in the kit handbook.
2. Disrupt 25-30 mg tissue in liquid nitrogen and add to a 2 mL centrifuge tube. Do not allow the tissue to thaw.
3. Add 450 µL Buffer RLT and vortex.
4. Transfer the lysate to a QIAshredder spin column (lilac) placed in a 2 mL collection tube. Centrifuge for 2 min at full speed. Transfer the supernatant of the flow-through to a new microcentrifuge tube (not supplied) without disturbing the cell-debris pellet.
5. Add 0.5 volume of ethanol (96–100%) to the cleared lysate and mix immediately by pipetting.
6. Transfer the sample (usually 650 µl), with any precipitate, to an RNeasy Mini spin column (pink) in a 2 mL collection tube (supplied). Close the lid and centrifuge for 15 s at ≥8000 x g (≥10,000 rpm). Discard the flow-through.
7. Add 350 µL Buffer RW1 to the RNeasy spin column.
8. Close the lid and centrifuge for 15s at ≥8000 x g.
9. Add 10 µL DNase I stock solution to 70 µL Buffer RD. Mix by inversion.

10. Add the mixture directly to the RNeasy spin column, incubate at room temperature for 15mins.
11. Add 350 µL Buffer RW1 to the RNeasy spin column. Close the lid and centrifuge for 15s at ≥8000 x g. Discard the flow-through.
12. Add 500 µl Buffer RPE to the RNeasy spin column. Close the lid, and centrifuge for 15 s at ≥8000 x g. Discard the flow-through.
13. Add 500 µl Buffer RPE to the RNeasy spin column. Close the lid, and centrifuge for 2 min at ≥8000 x g.
14. Place the spin column in a new 2 mL tube. Centrifuge at 1min to dry the membrane.
15. Add 450 µL Buffer RLT to a new centrifuge tube and place the spin column in this tube.
16. Add 50 µL Rnase free water directly to the spin column membrane. Close the lid, and centrifuge for 1min at >8000g to elute the RNA.
17. Discard the spin column.
18. Using the flow through as the lysate, repeat the procedure without the Dnase step, using new spin/shredder columns (i.e. Steps 4-7, 11-14). Then transfer the spin column to a new empty 2 mL tube and repeat Step 16 to elute the RNA. Store at -80 °C.

Supplementary Method 2. DNA extraction protocol

High molecular weight gDNA extraction from fresh/flash frozen *F. excelsior* leaves. Modified from protocol outlined in Franco Ortega *et al*.[7]. Outputs; 8-35ug DNA, >100ng/µL, 260/280 and 260/230 >1.8, peak fragment size > 60kb, ND/QT ratio < 2. Including sample disruption and QC, this usually took 2 days for 12 samples.

Materials

- 300-400mg fresh / flash frozen leaf tissue (~3 "large" leaflets to 10 "small" leaflets)
- QIAGEN Genomic-tip 100/G and associated Qiagen buffers
- Carlson buffer, pre-warmed to 65° C:
    - 100 mM Tris-HCl, pH 9.5
    - 2% CTAB
    - 1.4 M NaCl
    - 1% PEG 8000
    - 20 mM EDTA

    - To ensure all CTAB is dissolved, stir the Carlson buffer overnight
- B-mercaptoethanol
- Chroloform
- Isopropanol
- Proteinase K (20 mg/mL)
- RNase A (100 mg/mL)
- Liquid nitrogen
- Mortar and pestle
- Vortex mixer
- 50 mL Falcon tubes
- Centrifuge capable of taking 50 mL tubes
- Water baths at 65° C, 50° C and 55° C

Method

Note: use wide-bore pipette tips throughout. Gently invert to mix; do not vortex after step 3.

1. Transfer 8 mL of Carlson buffer to a 50 mL Falcon tube. In a fume hood, add 20 µl ß-mercaptoethanol to the Carlson buffer, mix by vortexing and pre-warm to 65° C in a water bath. Immediately before use, add 100 µl proteinase K.
2. Pre-cool the mortar and pestle with liquid nitrogen until both are at -80° C. This keeps the sample as a fine powder during grinding and will prevent re-activation of intracellular DNases. Pour ~30 mL of liquid nitrogen into the mortar and add 300-400 mg leaves. When the liquid nitrogen has evaporated, grind the tissue for approximately 30 seconds, to a flour-like consistency. Keep the sample at the bottom of the mortar as much as possible. Add another ~30 mL of liquid nitrogen and repeat grinding for approximately 30 seconds. Perform 2 - 4 cycles of grinding in total. Note: making sure the sample does not thaw, you can place foil over the mortar containing the sample and store at -80 - then you can do the downstream steps simultaneously (I did up to 12).
3. Transfer the frozen powdered tissue to the tube with the pre-warmed Carlson buffer and vortex the tube for 5 sec. Immediately transfer the tube to a 65° C water bath and incubate for 1 hour, mixing the sample by inversion at regular intervals (every 10 mins or so).
4. Add 40 µl of RNase A (100mg/mL), mix by inversion and incubate for a further 15 minutes.

5. Let the sample cool to room temperature, then add 1 volume of chloroform. Mix the sample by inversion.
6. Centrifuge the sample at 5500 g for 10 mins at 4° C.
7. Carefully transfer the top (aqueous) phase to a new 50 mL Falcon tube, without disturbing the interphase.
8. Repeat the chloroform wash steps 5-7 above.
9. Add 0.7 volumes of isopropanol to the top phase and mix thoroughly by inverting the tube 10 times. Place the tube at 4°C for 15 mins.
10. Centrifuge the sample at 5500 g for 30 mins at 4° C. Carefully discard the supernatant, without disturbing the pellet.
11. (Optional) If not proceeding to g-tip clean up that day, the pellet can be resuspended in an appropriate volume low TE (250 μL Qiagen Buffer AE) and stored at 4*C overnight. Alternatively, proceed to g-tip clean up as below.
12. Carefully dissolve DNA pellet at 5 mL of G2 buffer. Place the sample in a 50° C water bath for 15 mins, mixing occasionally until the pellet dissolves.
13. Equilibrate a QIAGEN Genomic-tip 100/G column with 4 mL of Buffer QBT.
14. Apply your fully dissolved DNA in G2 buffer to the equilibrated QIAGEN Genomic-tip 100/G column. Allow the DNA to enter the resin by gravity flow.
15. Wash the QIAGEN Genomic-tip 100/G with 10 mL of Buffer QC. Wait until all the buffer flows through the resin and repeat the wash with another 10 mL of Buffer QC. (Note; this is more than in the standard protocol, so a Genomic Buffer Kit will not have enough QC for 25 samples).
16. Place the QIAGEN Genomic-tip 100/G over a clean 50 mL Falcon tube and elute the genomic DNA with 5 mL of Buffer QF, pre-warmed to 55°C. Allow the eluate to cool down room temperature.
17. Precipitate the DNA by adding 0.7 volumes (3.5 mL) of room temperature isopropanol to the eluted DNA and mix by inverting the tube several times. Incubate at room temperature for 15 min.
18. Centrifuge at 5500 g for 30 mins at 4° C and carefully remove the supernatant. The pellet will likely be highly fragmented, the supernatant will need to very carefully pipetted off.
19. Wash the centrifuged DNA pellet with 4 mL of cold 70% ethanol. Invert the tube several times to disturb the pellet, and centrifuge at 5500 g for 10 min at 4°C.

20. Carefully remove the supernatant without disturbing the pellet. The pellet will likely be highly fragmented, the supernatant will need to very carefully pipetted off.
21. Air dry the pellet for 10 min and resuspend the DNA in 100 µL of 1x TE buffer pH 8.
22. For high concentrations/quantities of DNA (>100ng/µL), the DNA will take a long time to resuspend. Leaving for 3 days at room temperature and 4 days at 4°C (highly variable readings on a spectrophotometer from a single sample suggests it has not fully resuspended).

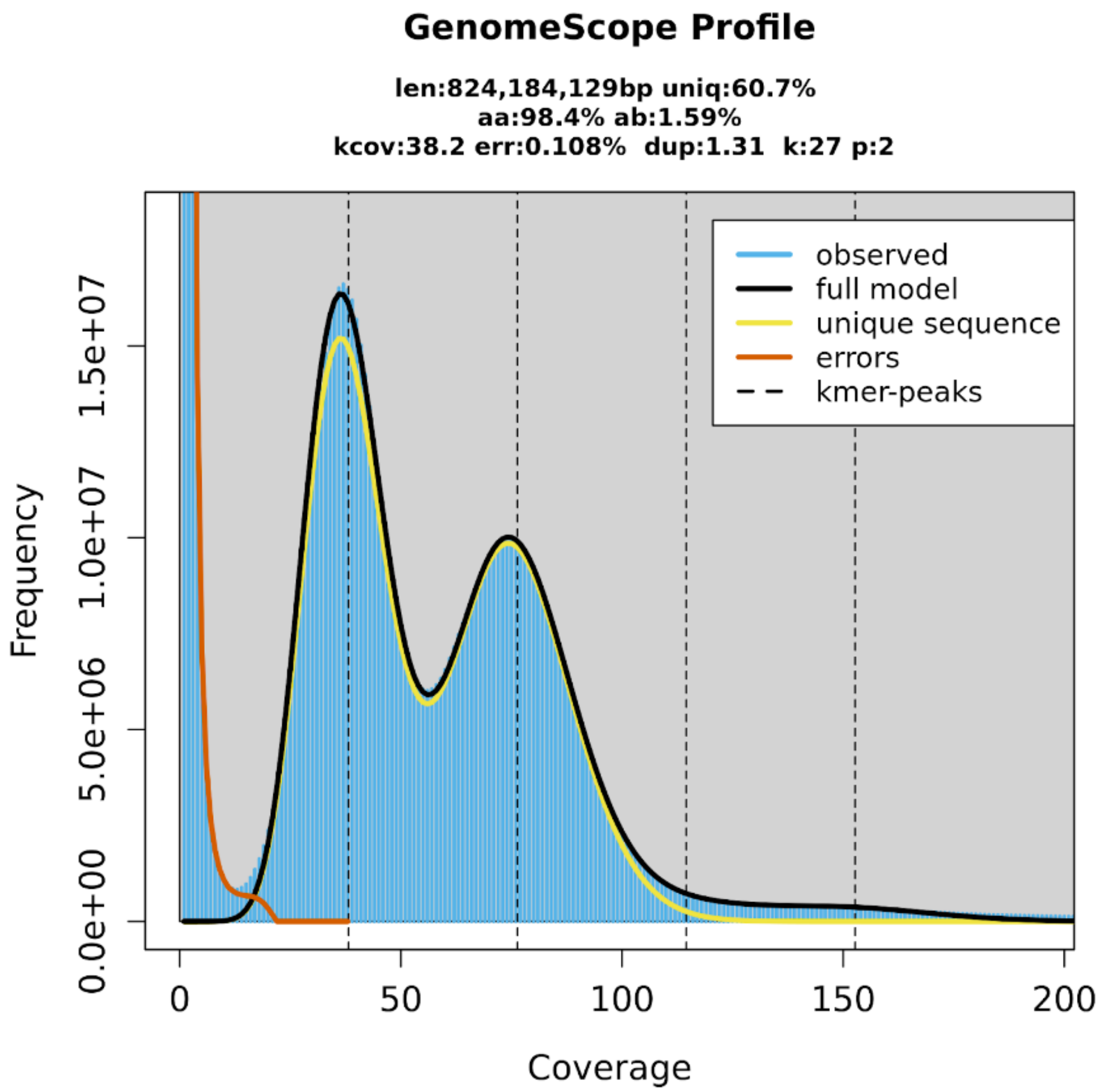

Supplementary Figure 1. Kmer coverage profile for BATG-1.0 PacBio HiFi reads. Frequencies and coverage of 27bp kmers from PacBio HiFi data for the BATG-1.0 individual using Genomescope[10]. Plot produced internally by Genomescope from fastq files (PRJNA906474) – see Methods for details to recreate plot.

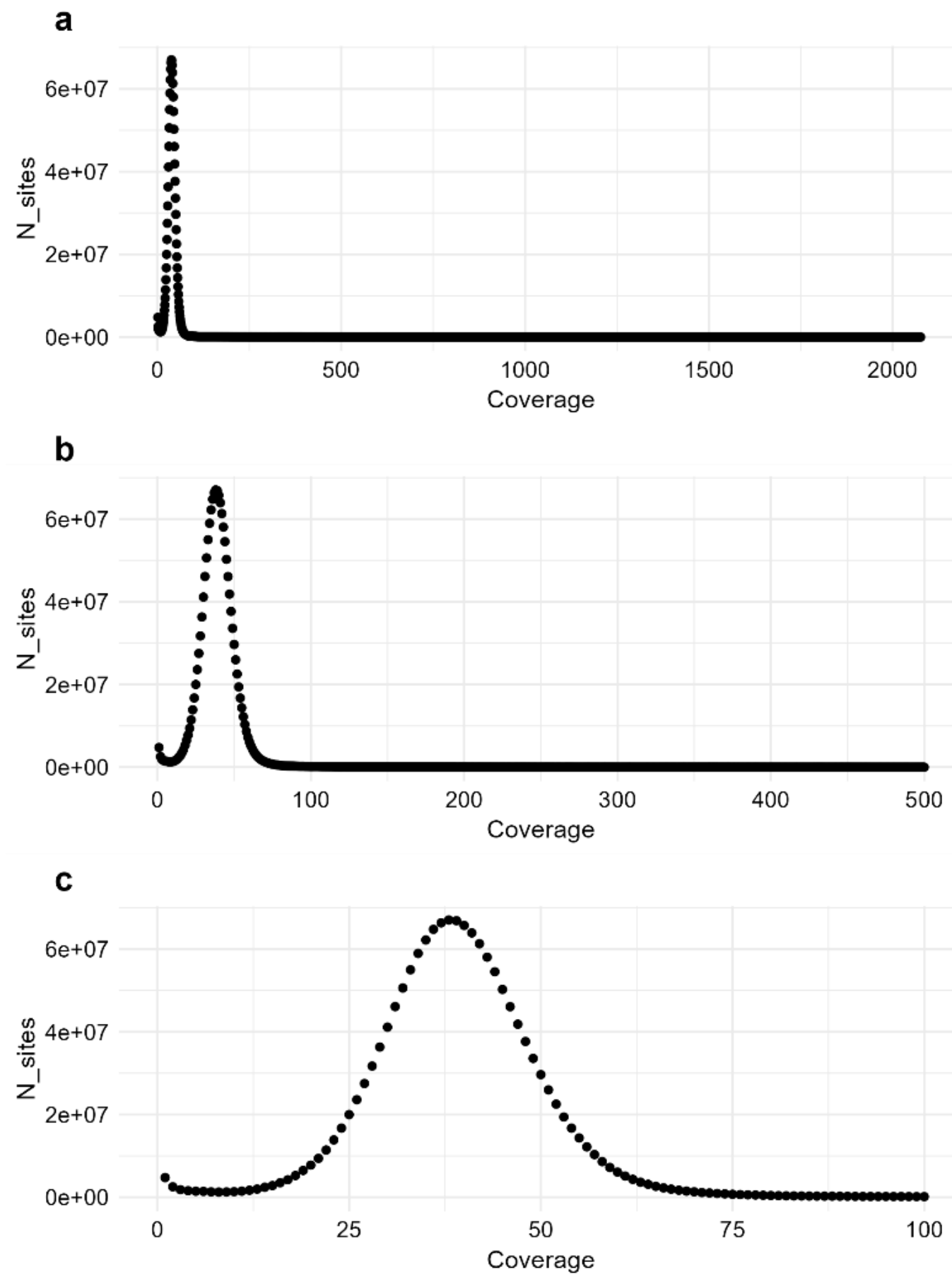

Supplementary Figure 2. Coverage profiles of phased linear reference genome. Coverage profiles of PacBio long-reads used to construct the phased assemblies, mapped to a concatenated fasta file of both haplotypes, with per-site coverage on the X axis, and the number of sites displaying this coverage on the Y axis. For legibility, the same data are displayed with X axis cutoffs of A) 2086 (maximum per-site coverage value was 2076), B) 500 and C) 100. Source data are provided as a Source Data file.

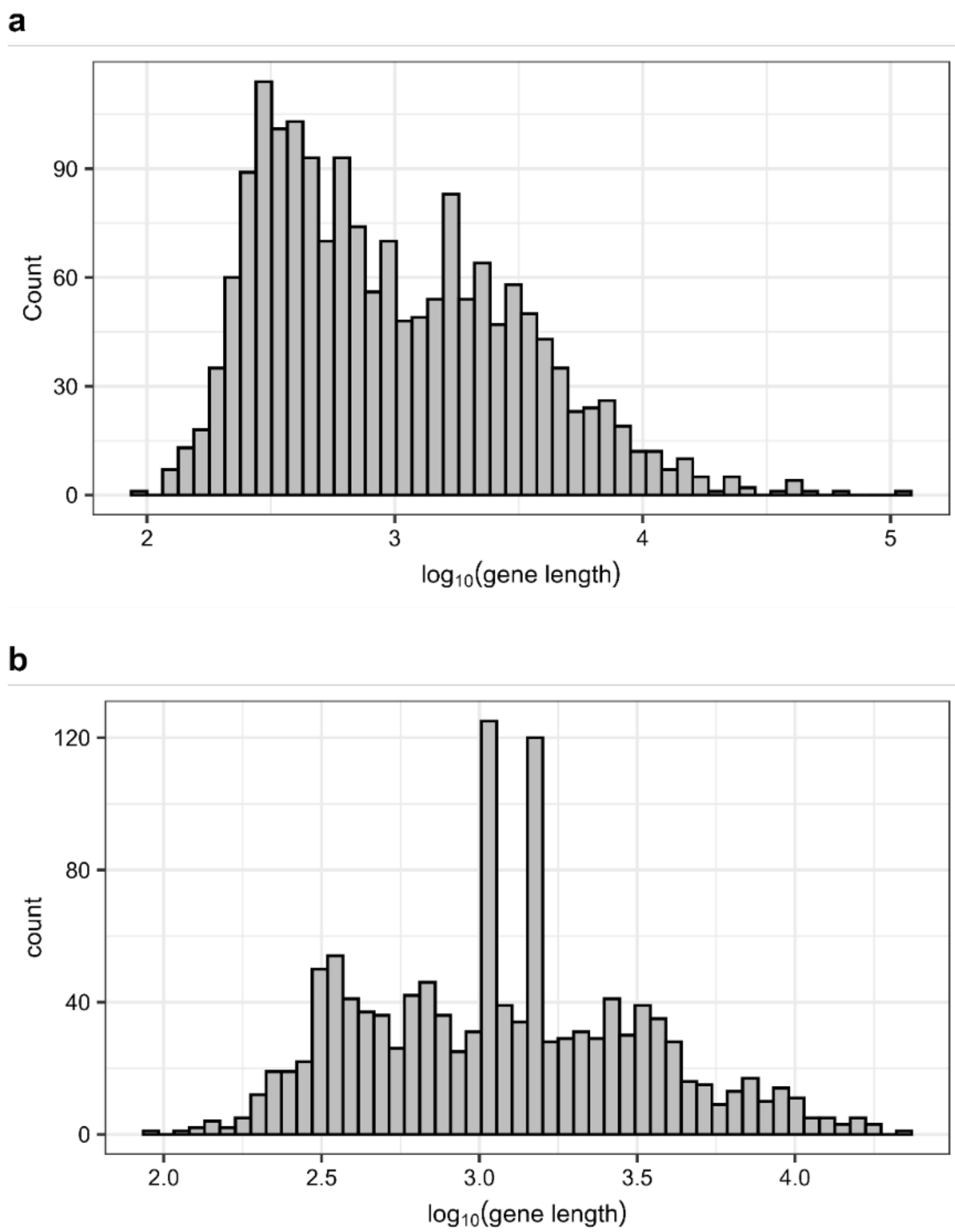

Supplementary Figure 3. Length distributions for BATG-1.0 genes identified exclusively with one RNA-seq data type. Histograms of $\log_{10}$ transformed gene length for genes in BATG-1.0 identified exclusively by short-read RNA seq data or long-read RNA seq data. A) Lengths for genes identified in all short-read BRAKER3 runs, but no long-read BRAKER3 runs, and B) genes identified in all long-read BRAKER3 runs, but no short-read BRAKER3 runs. Source data are provided as a Source Data file.

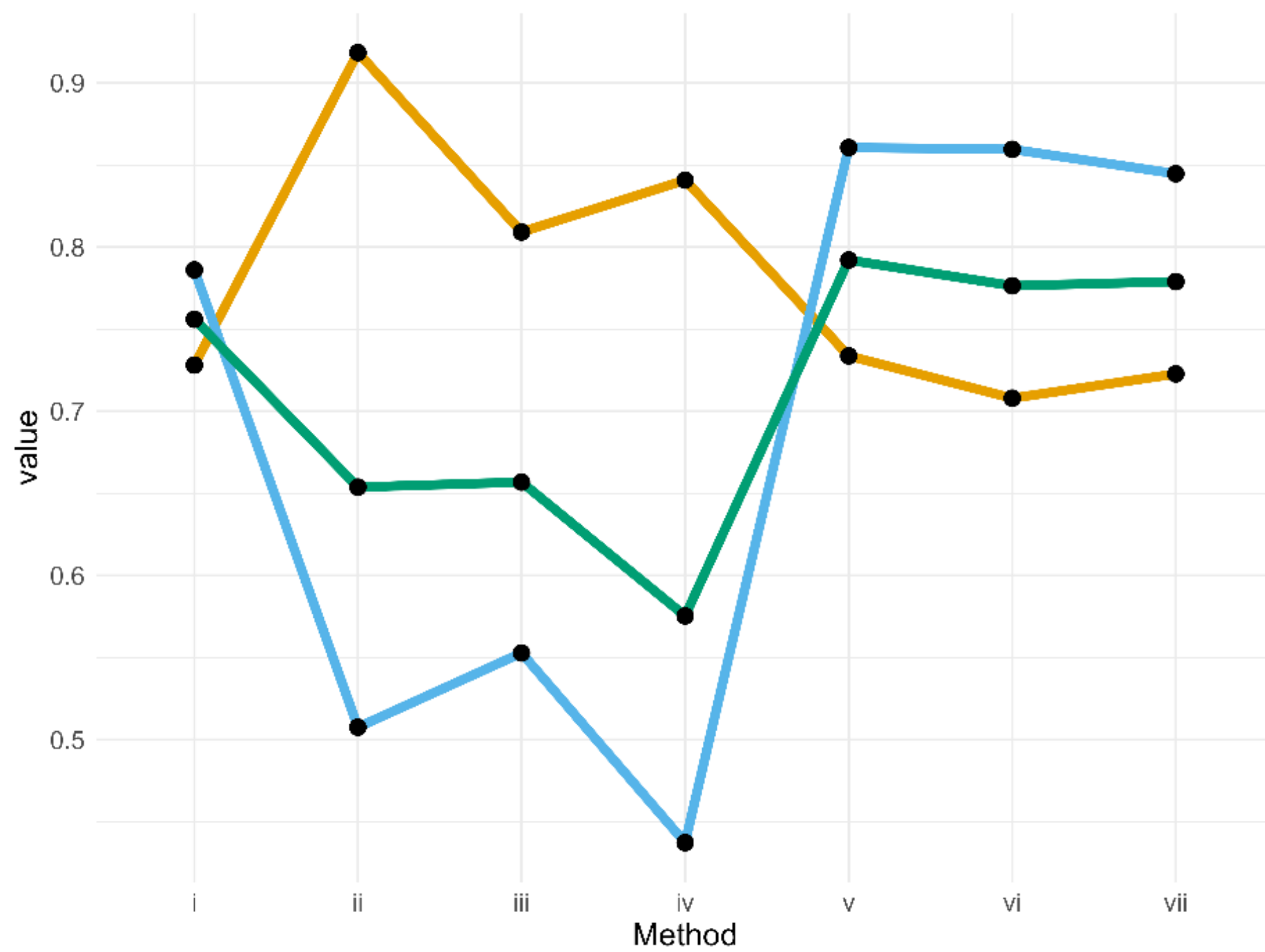

Supplementary Figure 4. Precision, recall and F1 scores for different structural variant calling methods. Values of the precision (orange), recall (blue) and F1 score (green) of structural variant (SV) calling for different methods. Values were based on comparing SVs called using the long-read sequencing data from the reference individual, mapped to BATG-1.0 either as individual reads or assembled contigs, with the putative truth set comprising variants called by mapping the second haplotype of the reference genome to BATG-1.0 and calling SVs with svim-asm. The methods considered were: i) SVs called by both cuteSV and Sniffles2; ii) SVs called by svim-asm with a mapping of the *de novo* assembly produced by shasta; iii) SVs called by svim-asm with a mapping the *de novo* assembly produced by Flye; iv) SVs called by svim-asm with a mapping of *de novo* assembly produced by nextDeNovo; v) SVs called by methods i and/or ii; vi) SVs called by methods i and/or iii; vii) SVs called by methods i and/or iv. Source data are provided as a Source Data file.

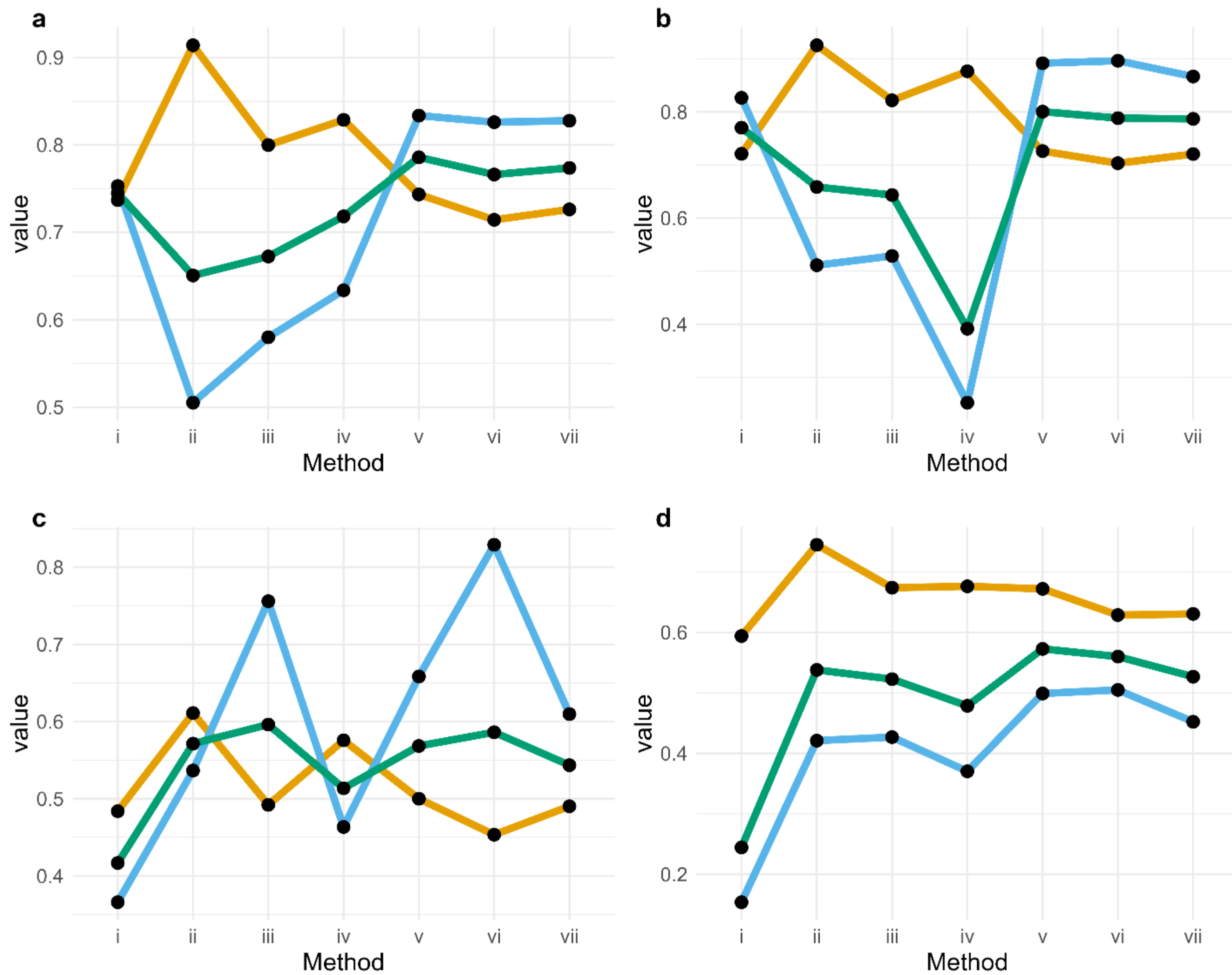

Supplementary Figure 5. Class specific precision/recall/F1 of structural variants in BATG-1.0. Values of the precision (orange), recall (blue) and F1 score (green) of structural variant (SV) calling for different methods. Values were based on comparing SVs called using the long-read sequencing data from the reference individual, mapped to BATG-1.0 either as individual reads or assembled contigs, with the "truth" set comprising variants called by mapping the second haplotype of the reference genome to BATG-1.0 and calling SVs with svim-asm. Each panel denotes these values for A) Insertions, B) Deletions, C) Inversions and D) Tandem Duplications. The methods considered were: i) SVs called by both cuteSV and Sniffles2; ii) SVs called by svim-asm with a mapping of the *de novo* assembly produced by shasta; iii) SVs called by svim-asm with a mapping the *de novo* assembly produced by Flye; iv) SVs called by svim-asm with a mapping of *de novo* assembly produced by nextDeNovo; v) SVs called by methods i and/or ii; vi) SVs called by methods i and/or iii; vii) SVs called by methods i and/or iv. Source data are provided as a Source Data file.

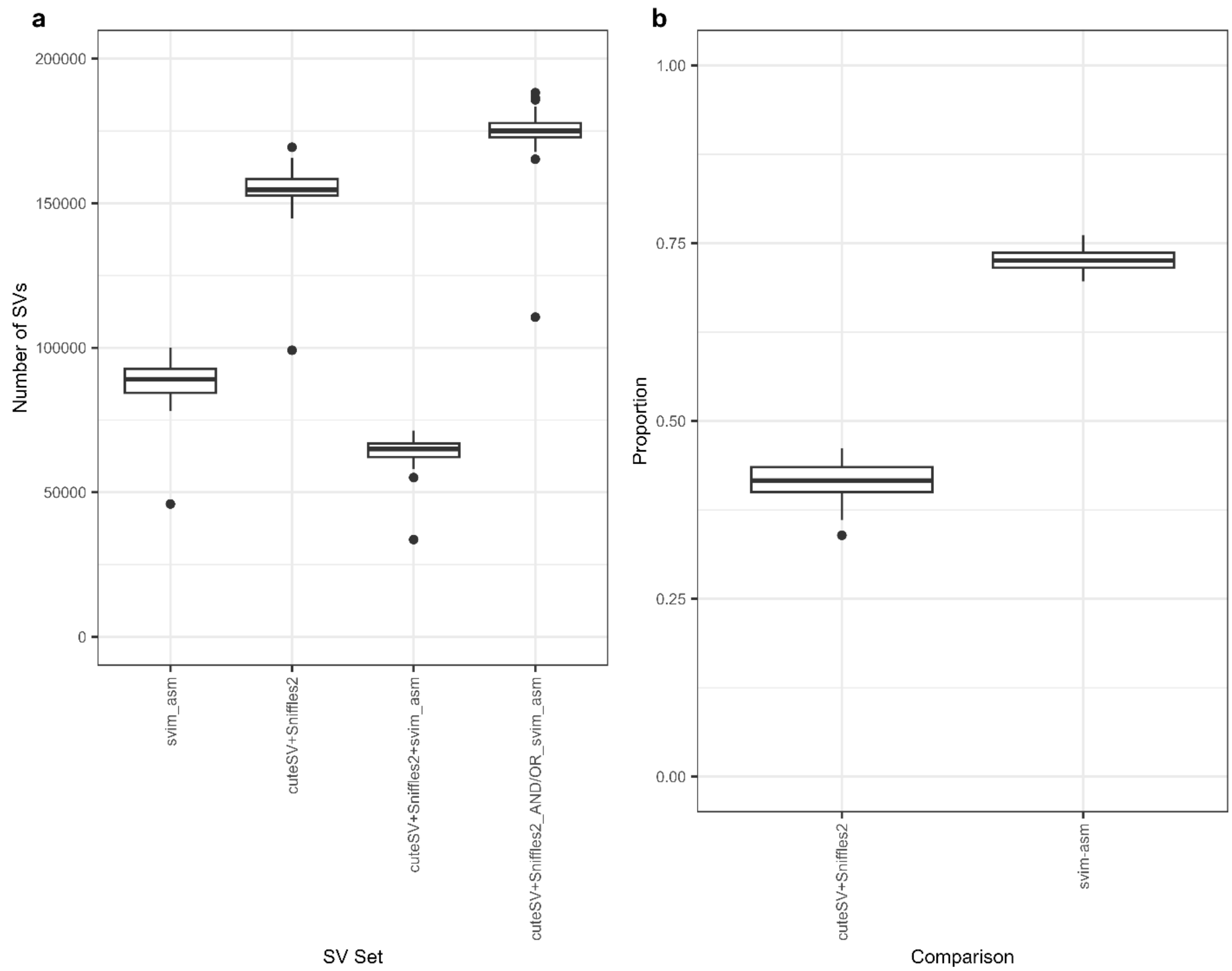

Supplementary Figure 6. Structural variant caller concordance across different individuals. A: Boxplots showing, for each of the methods outlined on the X axis, the total number of structural variants (SVs) called per sample. Methods are either i) SV called by svim-asm, ii) SVs called by both cuteSV[11] and Sniffles2[12], iii) SVs called by cuteSV, Sniffles2 and svim-asm[13], iv) SVs called by either cuteSV and Sniffles2, and/or SVs called by svim-asm. B: Boxplots showing, for each sample, the proportion of total SVs called per sample called by i) cuteSV and Sniffles2, and ii) svim-asm, for the set of SVs called by either/both of these methods. Source data are provided as a Source Data file.

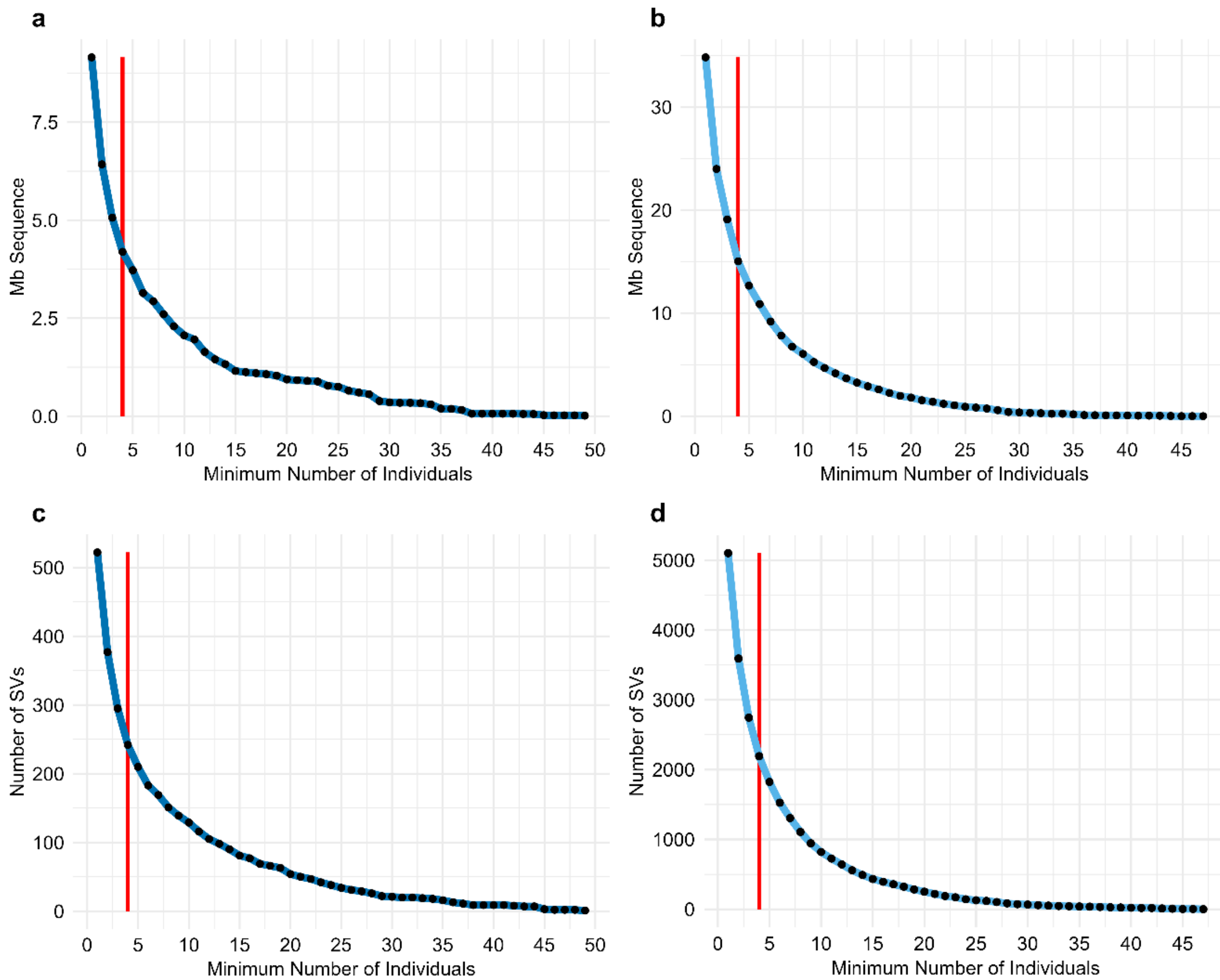

Supplementary Figure 7. Inversion and tandem duplicate frequencies. The same data as Figures 2A and 2B are plotted for inversions and tandem duplicates separately to aid visualisation. The total quantity of sequence (A, B) and total number of structural variants (SVs; C, D) of different SV classes, at different minimum numbers of individuals called from long-read data. Data for inversions are plotted in panels A and C, data for tandem duplications in panels B and D. A vertical red line at n = 4 indicates the cutoff for SVs being included in the pangenome. Source data are provided as a Source Data file.

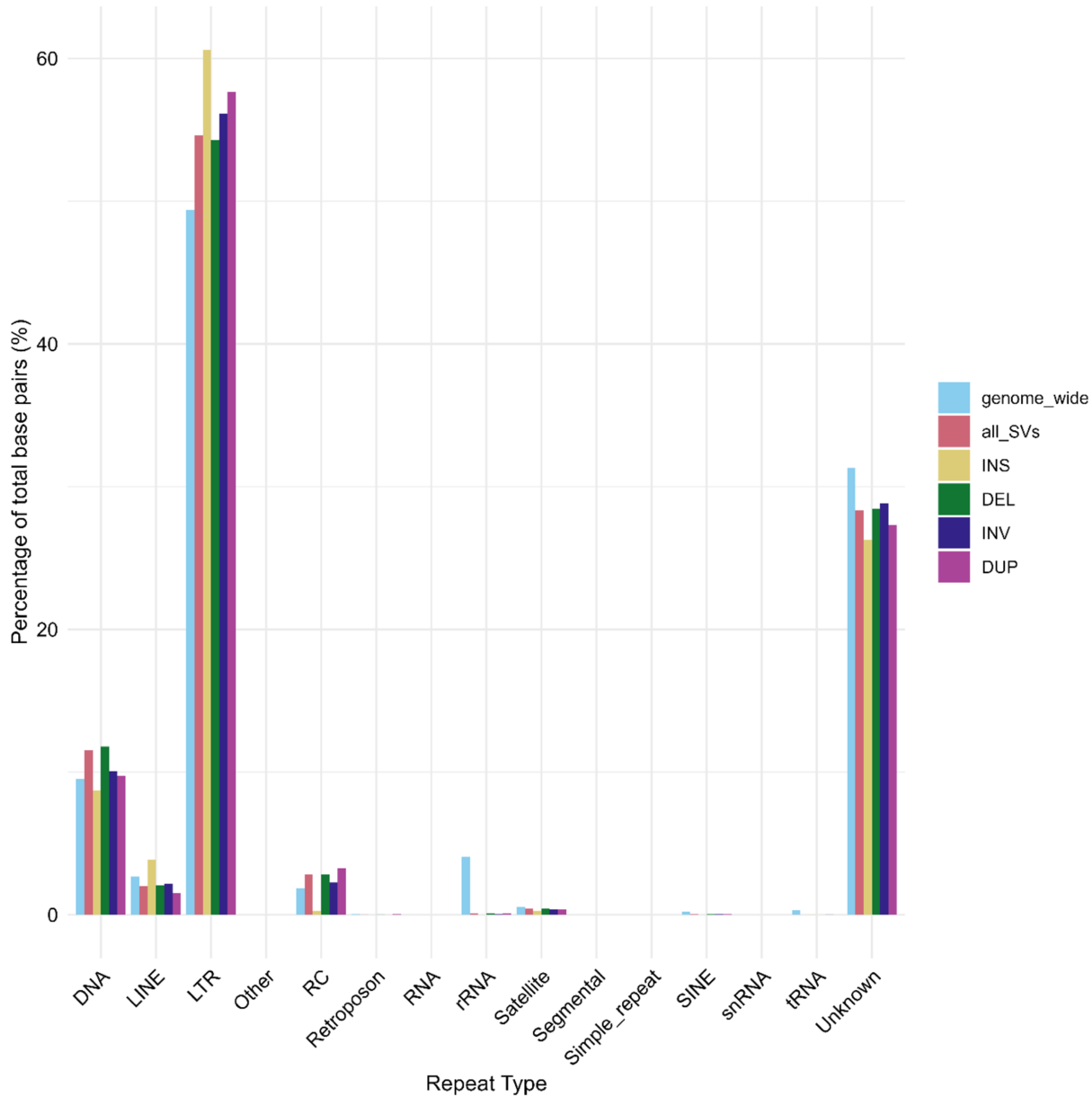

Supplementary Figure 8. Classes of repeats overlapping structural variants. For i) the genome as a whole, ii) all classes of structural variants, iii) insertions (INS), iv) deletions (DEL), v) inversions (INV) and tandem duplications (DUP), the percentage of base pairs overlapping with various classes of repetitive element are plotted, grouped by repetitive element. These are i) DNA transposons, ii) long interspersed nuclear elements (LINEs), iii), long terminal repeat (LTR) transposons, iv) miscellaneous classes of element ("Other"), v) rolling-circles (RC), vi) retrotransposons, vii) RNAs, viii) rRNAs, ix) Satellites, x) segemental duplications, xi) simple repeats, xii) short interspersed nuclear elements (SINEs), xiii) tRNAs, and xiv) unclassified repeats ("Unknown"). Source data are provided as a Source Data file.

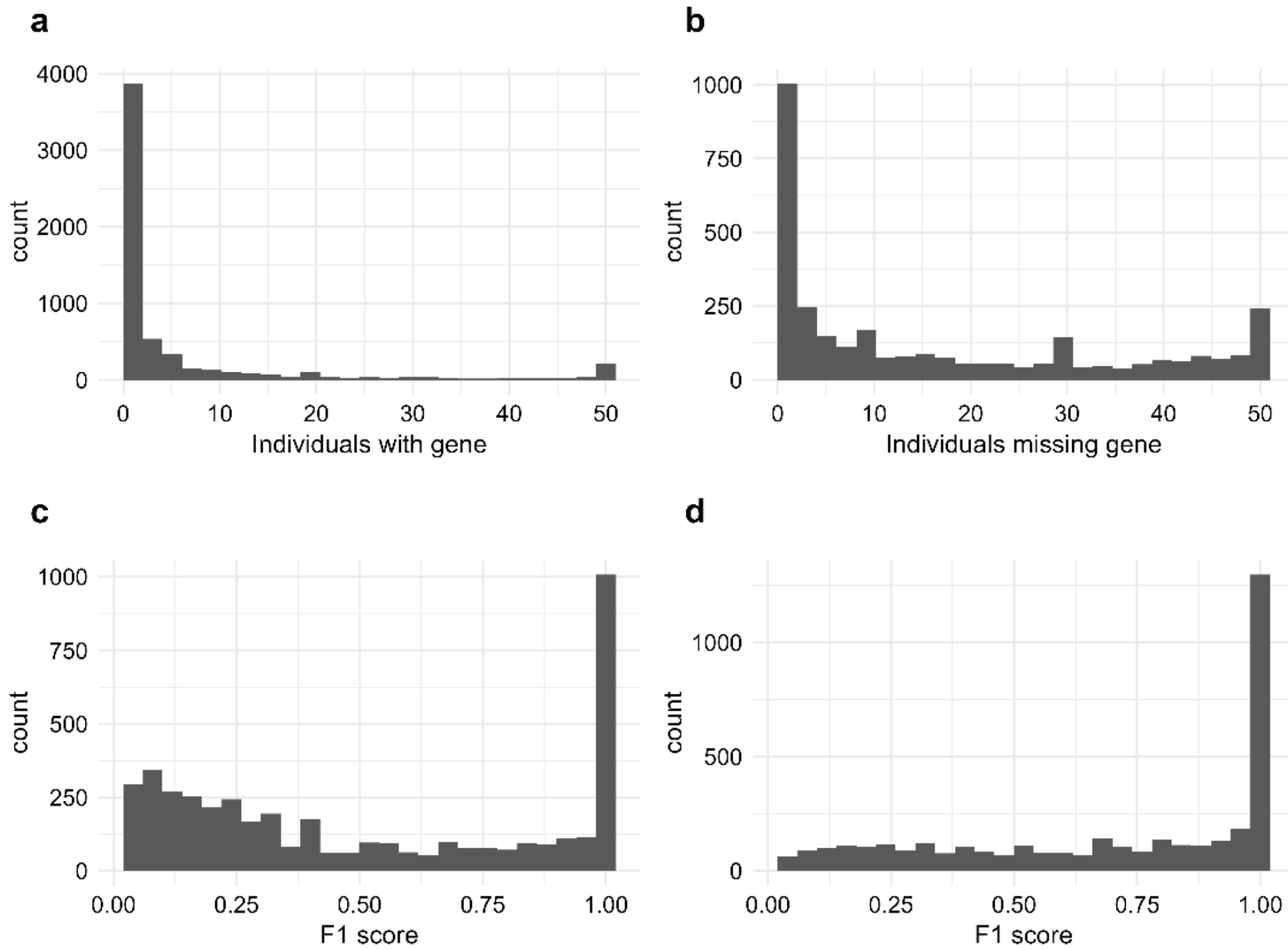

Supplementary Figure 9. F1 scores for putatively variable genes association with structural variants. Histograms showing A) for genes absent from the linear reference annotation but present in at least one other individual, but not overlapping with a structural variant (SV), the number of individuals where this gene was present; B) for genes present in the linear reference annotation but absent from at least one other individual, but not overlapping with a SV, the number of individuals where the gene was absent, C) for genes absent from the linear reference annotation but present in at least one individual with a SV overlapping the gene location, the best F1 score of the SVs overlapping the gene, D) for genes present in the linear reference individual but absent from at least one individual where an SV overlaps with the gene sequence, the best F1 score of the SVs overlapping the gene. Source data are provided as a Source Data file.

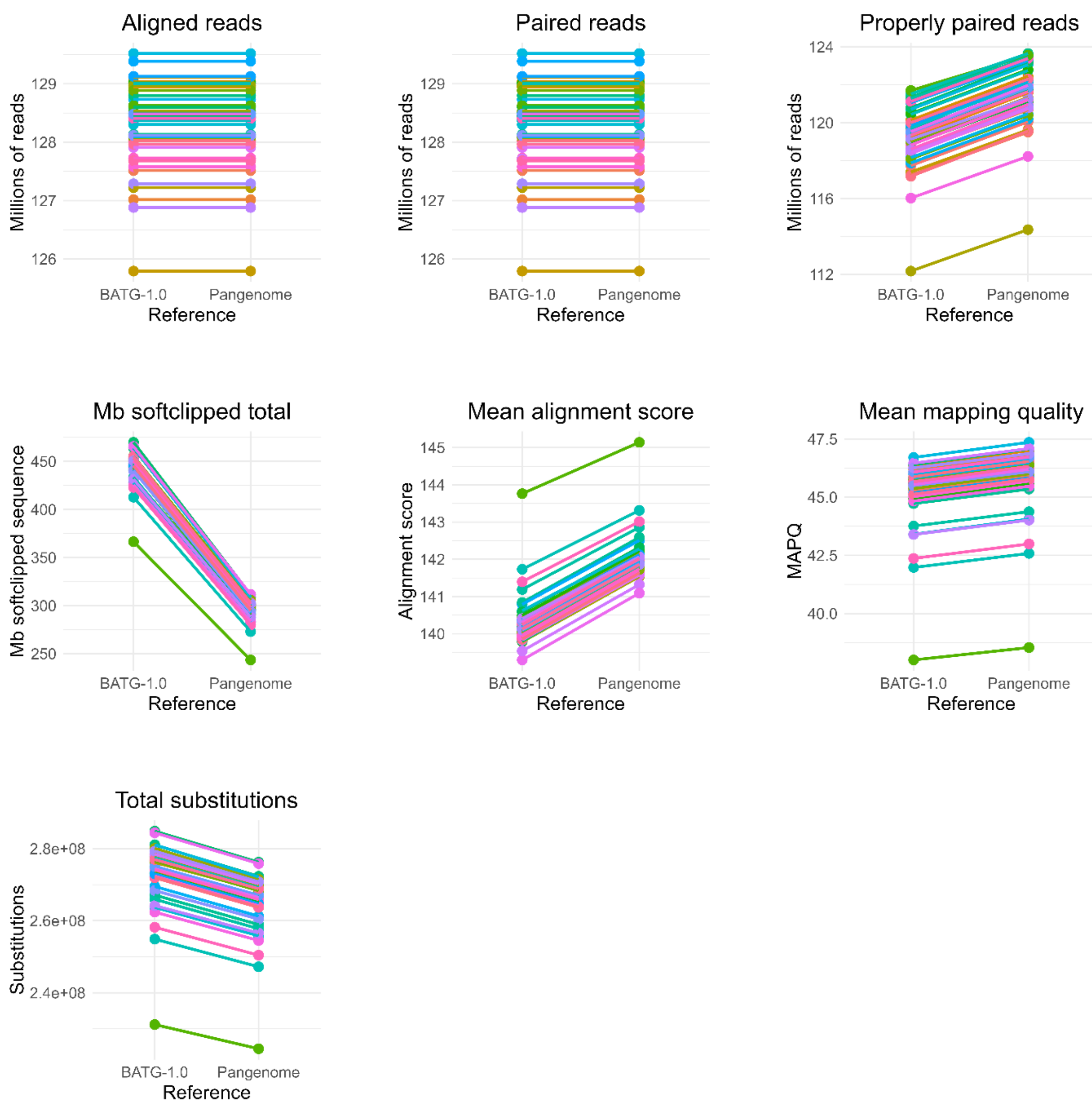

Supplementary Figure 10. Read mapping statistics for the linear reference vs. the pangenome. Read mapping statistics for short reads from 42 samples sequenced in Stocks *et al.* [1], mapped using giraffe either to the linear reference genome in pangenome format (BATG-1.0), or to the full pangenome (Pangenome). Each individual is represented by a different colour - lines connect values for each individual in the reference-only pangenome to the full pangenome in the individual panels. The panels outline i) the total number of aligned reads, ii) the total number of paired reads, iii) the total number of properly paired reads, iv) the number of megabases of softclipped sequences, v) the mean alignment score, vi) the mean mapping quality and vii) the total number of substitutions, as estimated by the vg stats function. Source data are provided as a Source Data file.

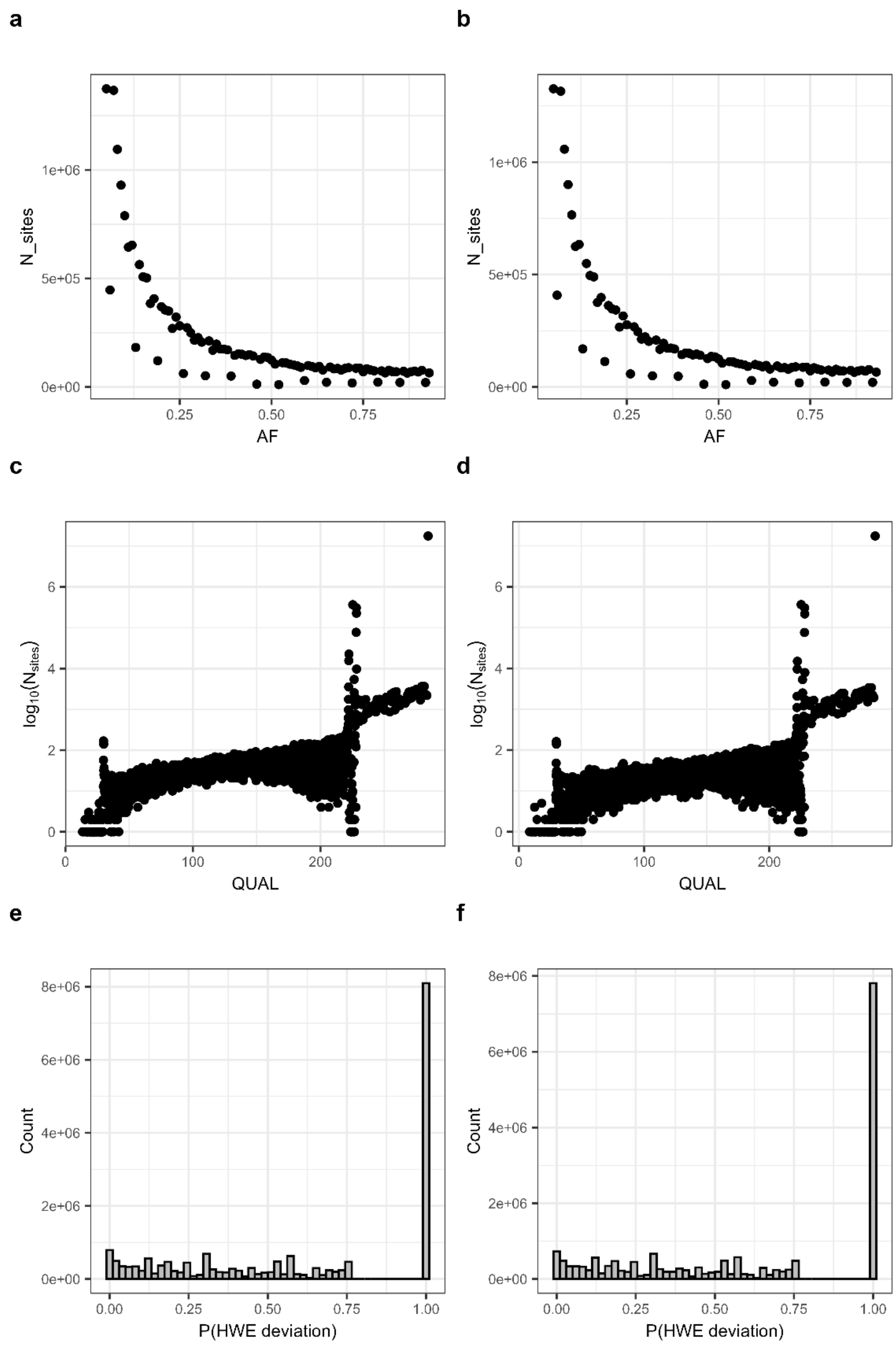

Supplementary Figure 11. Single nucleotide polymorphism calls from linear reference genome vs. pangenome. For single nucleotide polymorphisms (SNPs) called using linear reference genome (column 1: panels A, C, E) and the full pangenome (column 2, panels B, D, F), i) the frequency of SNPs with varying levels of allele frequency (AF; panels A + B), ii) the $\log_{10}$(frequency) of QUAL scores (panels C and D), and iii) the frequency of sites with a given *p*-value from the Hardy-Weinburg equilibrium (HWE) test from bcftools (a two sided chi-squared test; panels E and F). Source data are provided as a Source Data file.

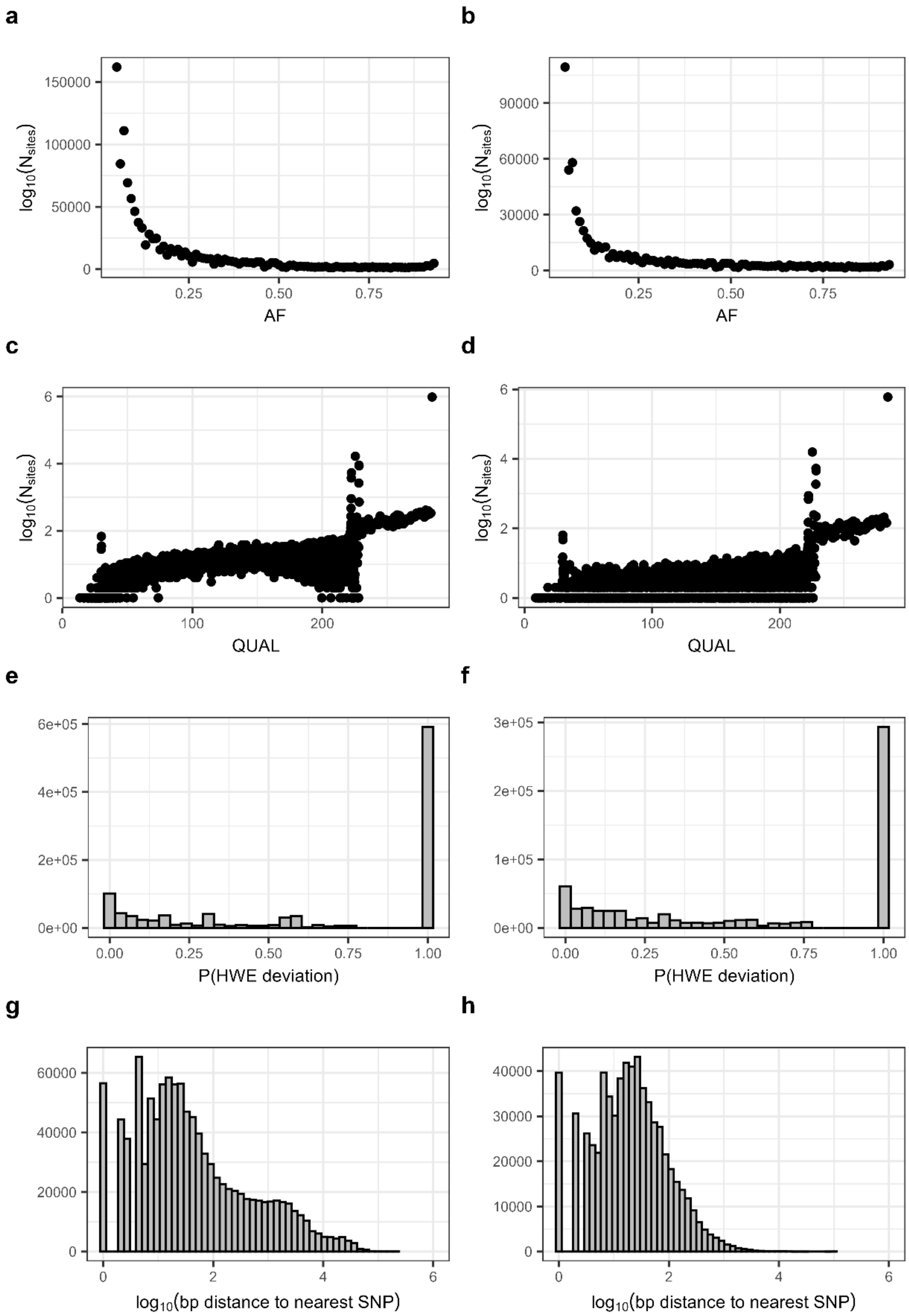

Supplementary Figure 12. Single nucleotide polymorphisms called exclusively from linear reference genome vs. exclusively from pangenome. For single nucleotide polymorphisms (SNPs) called exclusively when using linear reference genome (column 1, panels A, C, E and G), and called exclusively when using the pangenome (column 2, panels B, D, F and H), i) the frequency of SNPs with varying levels of allele frequency (AF; panels A and B), ii) the $\log_{10}$(frequency) of QUAL scores (panels C and D), and iii) the frequency of sites with a given *p*-value from the Hardy-Weinburg equilibrium (HWE) test in bcftools (a two-sided chi-squared test; panels E and F), iv) the $\log_{10}$(basepair distance) of each SNP, to the closest SNP in the full alternate callset (panels G and H). Source data are provided as a Source Data file.

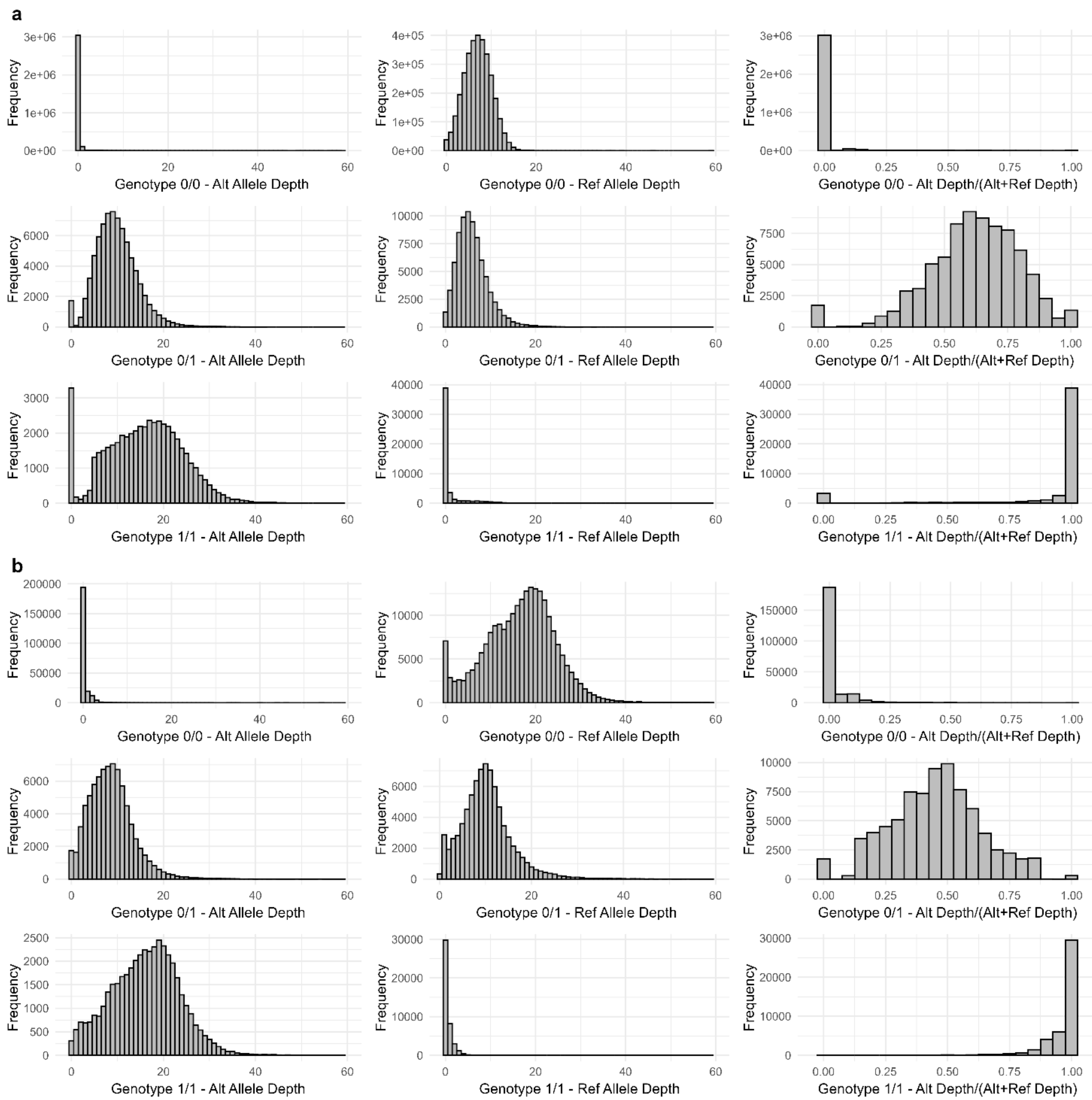

Supplementary Figure 13. Allele depths for single nucleotide polymorphism and structural variant calls from data for an example individual. Histograms outlining allelic depths for A) single nucleotide polymorphisms (SNPs) and B) structural variants (SVs) called from an example individual - Individual 164 from Stocks *et al.*[1]. The panels indicate - for homozygous reference calls (top row), heterozygote calls (middle row) and homozygous alternate calls (bottom row) - the alternate allele depth (first column), reference allele depth (second column) and the alternate allele depth as a proportion of the total allelic depth (last column). Source data are provided as a Source Data file.

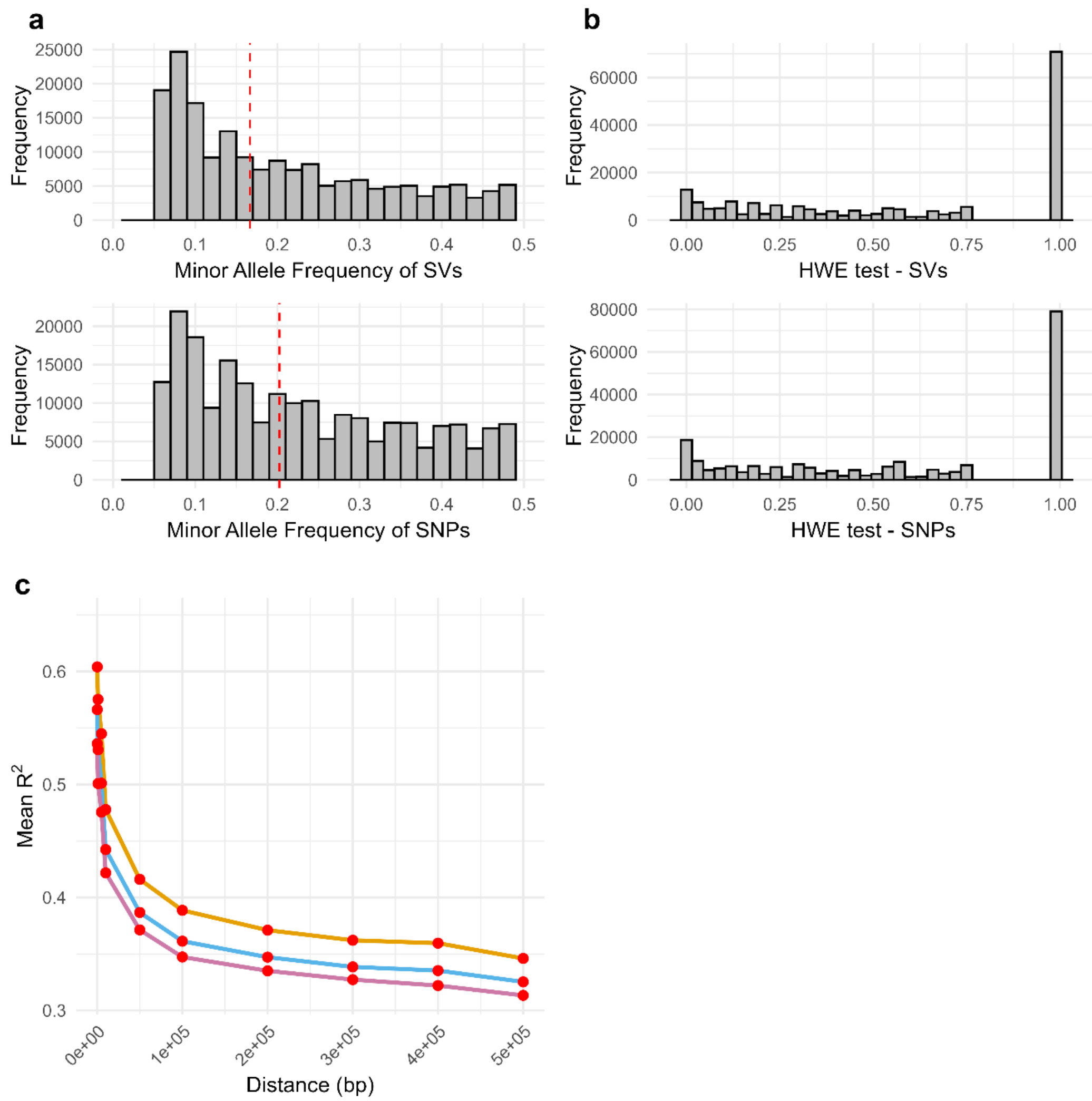

Supplementary Figure 14. Statistics of SNP and SV calls combined across individuals. A) Histograms of minor allele frequencies for SNPs (top panel) and SVs (bottom panel) called from a set of individually sequenced samples from Stocks *et al.*[1]. Red vertical dotted line indicates the median value of minor allele frequency for both datasets. B) For the same dataset, *p*-values from the Hardy-Weinburg equilibrium (HWE) test (two-sided chi-squared test) calculated by bcftools. C) Estimates of linkage disequilibrium decay by distance between SNPs (orange line), SVs (purple line) and between SNPs and SVs (blue line). Pairwise linkage disequilibrium was estimated using plink2[14], with mean R^2 values in predefined bins (red points) estimated in R. Source data are provided as a Source Data file.

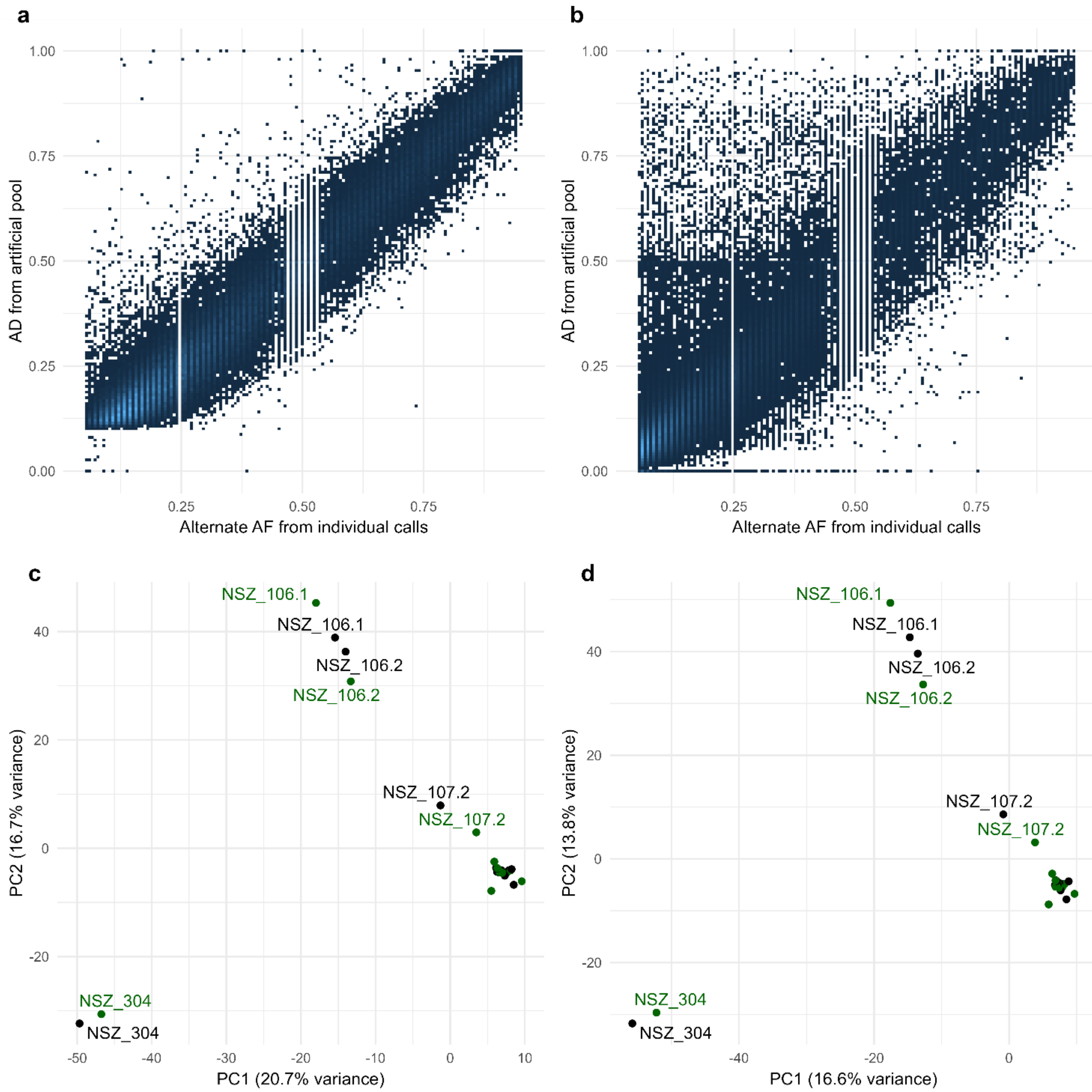

Supplementary Figure 15. Structural variant and single nucleotide polymorphism calling from poolseq data. A), B) Heatmaps of alternate allele frequencies for single nucleotide polymorphisms (SNPs) (A) and structural variants (SVs; B) estimated from individual calls (X axis) from a subset of individually sequenced ash trees from Stocks *et al.* [1] vs. an allele frequency estimate from *in silico* pools generated by randomly downsampling reads for these individuals, and measuring the allelic depth (AD; Y axis). Colour scale represents the number of sites in a square. C), D) Plots of the first two principal components (PCs) from principal components analysis from allele frequencies estimated for SNPs (C) and SVs (D) from the poolseq data from Stocks *et al.*[1]; axis labels represent the percentage of total variance explained by the PCs. Point labels indicate pool provenances from Stocks *et al.* [1] for a subset of pools (i.e. those outside the main dense cluster). Suffixes .1 and .2 represent biological replicate pools. Colours correspond to pool health scores - healthy (green) and unhealthy (black). Source data are provided as a Source Data file.

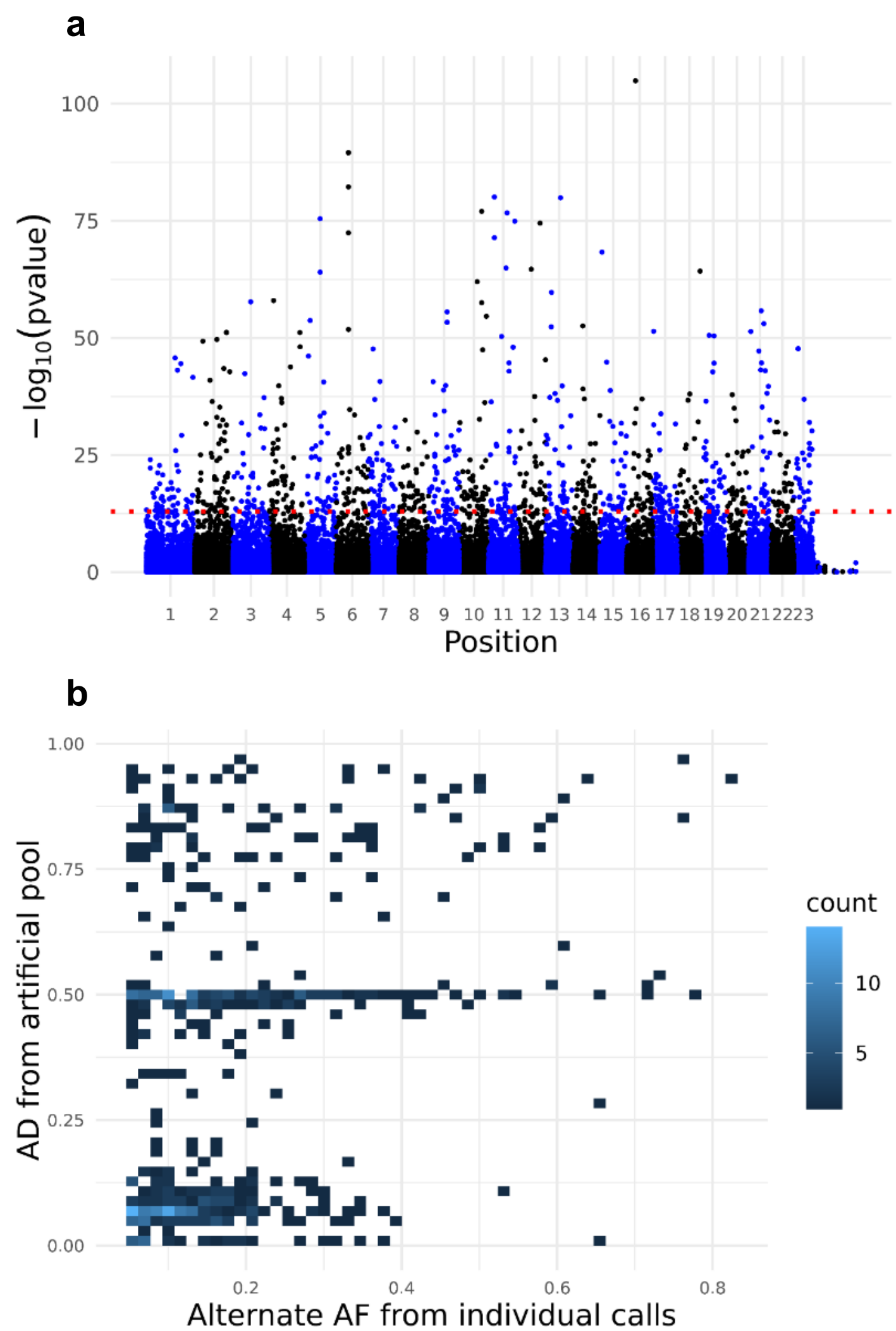

Supplementary Figure 16. Genome-wide association using structural variant allele frequencies estimated from poolseq data. A) Manhattan plot for structural variants (SVs) called from poolseq data from Stocks *et al.* [1], identifying association with ash dieback disease damage. Y axis values represent the unadjusted $-\log_{10}$ *p*-values of a two-sided Cochran-Mantel Haenszel (CMH) test (red horizontal line corresponds to $p = 1 \times 10^{-13}$). Sites are ordered by position along scaffolds in the BATG-1.0 genome assembly, with alternating black and blue colours representing the different scaffolds. Scaffolds are displayed in order of size, with the largest on the left and smallest on the right. B) For SVs identified as significant in the CMH test, heatmap of alternate allele frequencies estimated from individual calls (X axis) from a subset of individually sequenced ash trees from Stocks *et al*. vs. an allele frequency estimate from *in silico* pools generated by randomly down sampling reads for these individuals and measuring the allelic depth (AD; Y axis). Colour scale represents the number of sites in a square. Source data are provided as a Source Data file.

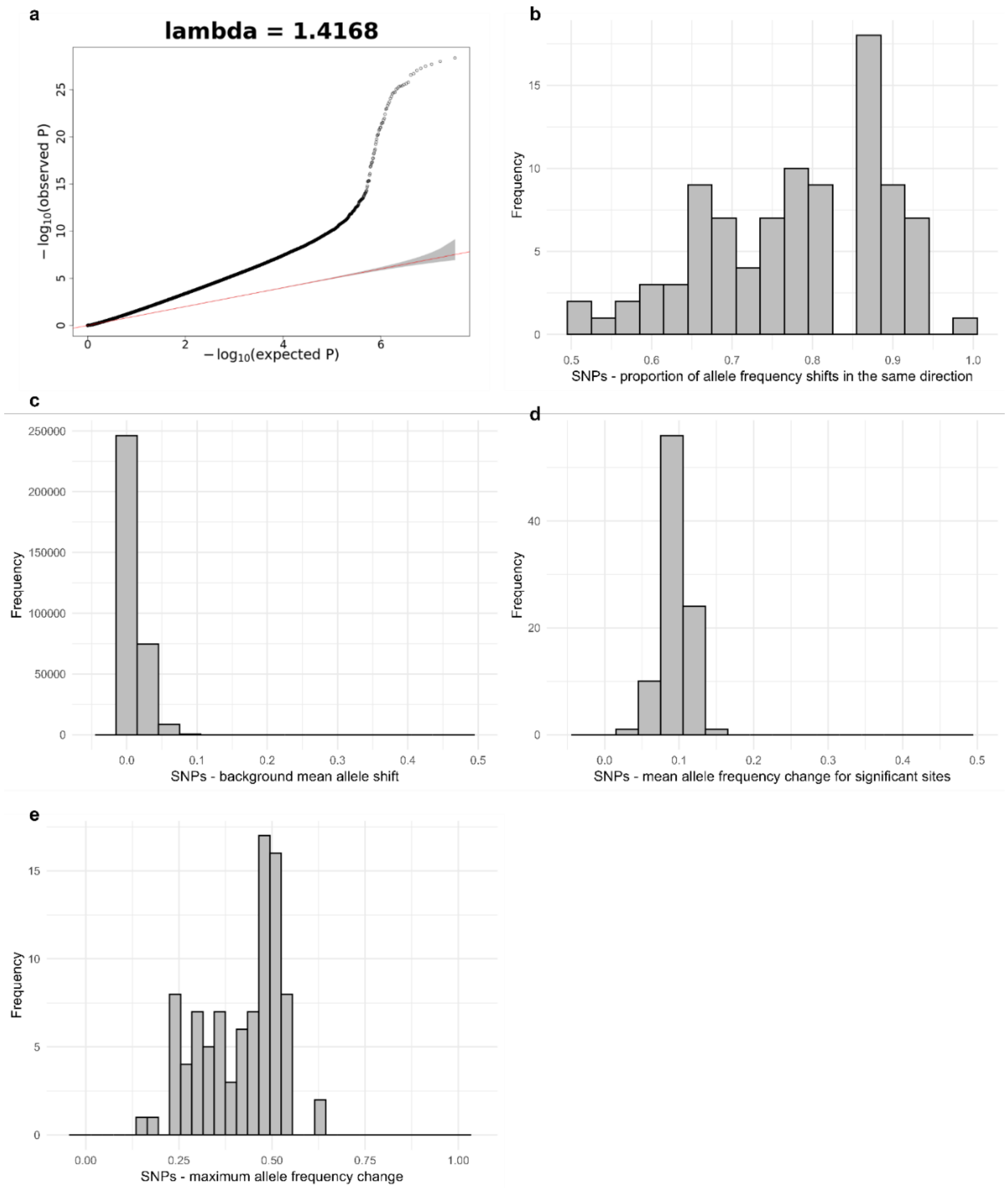

Supplementary Figure 17. Cochran-Mantel-Haenszel test statistics for single nucleotide polymorphisms. A) quantile-quantile plots for -$\log_{10}$(*p*-values) from two-sided Cochran-Mantel Hahn (CMH) tests from single nucleotide polymorphism (SNP) allele frequencies, as implemented in the GWASTools[15] package. Genomic inflation factor (lambda) is indicated at the top of each plot. B) Histogram indicating the proportion of allele frequency shifts occurring in the most frequent direction between healthy and unhealthy pools, excluding pairs of pools where the allele frequency shift is 0. C) For a random 1% of SNPs considered in the analysis, histogram of the mean absolute value of allele frequency shift between unhealthy and healthy pools. D + E) For SNPs identified as significant using the CMH test, histogram of the D) mean and E) maximum absolute value of allele frequency shift between unhealthy and healthy pools. Source data are provided as a Source Data file.

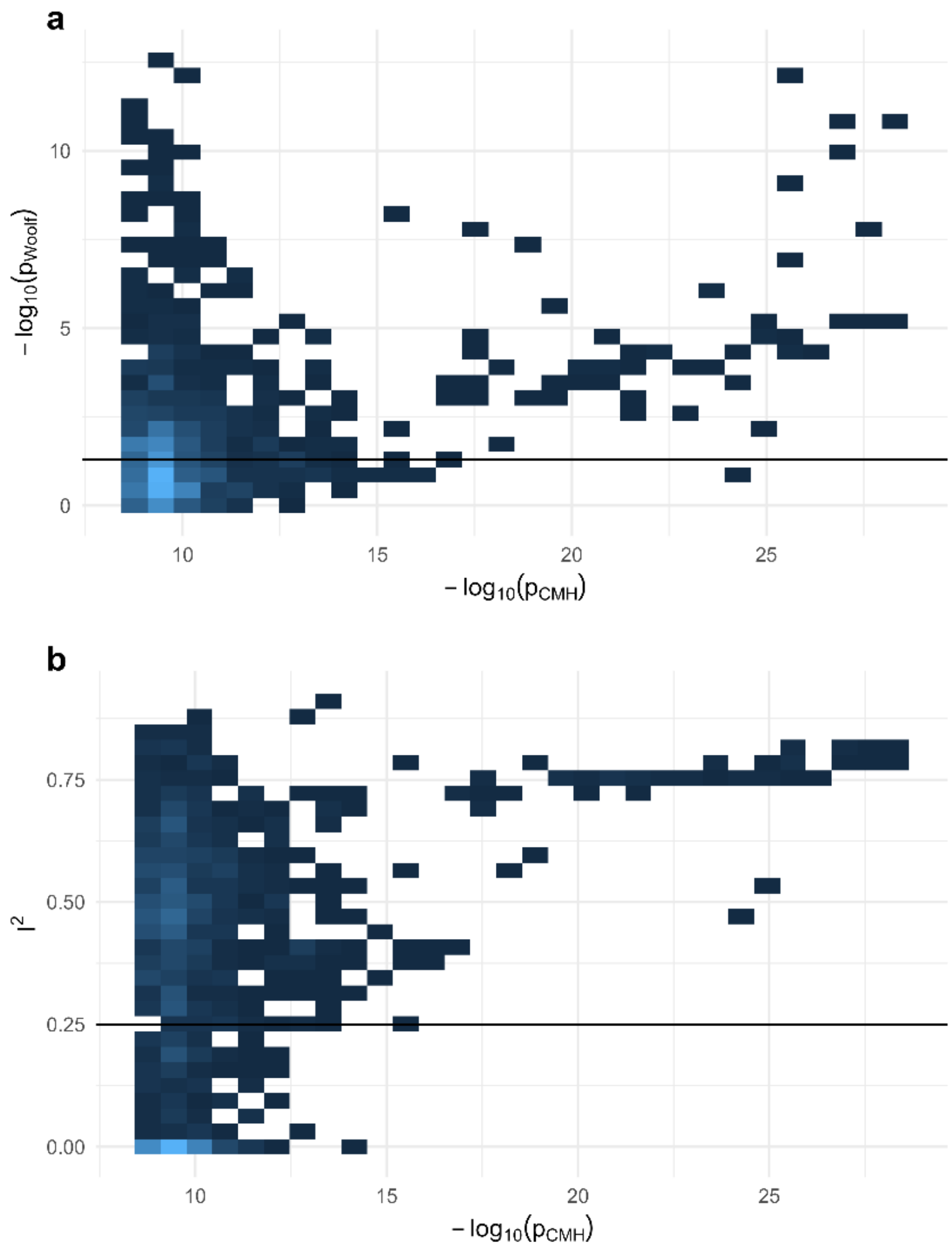

Supplementary Figure 18. Heterogeneity tests for single nucleotide polymorphisms with significant *p*-values from Cochran-Mantel Haenszel test. For the 866 single nucleotide polymorphisms (SNPs) with significant *p*-values from the Cochran-Mantel-Haenszel (CMH) test) after Bonferroni correction (α = 0.05) , $-\log_{10}$(*p*-values) from the CMH test are plotted on a heatmap against A) $-\log_{10}$(*p*-values) from a Woolf test, and B) values of the I^2 statistic. Horizontal lines represent thresholds for significant heterogeneity; $p < 0.05$ for the Woolf test, and $I^2 > 0.25$. Source data are provided as a Source Data file.

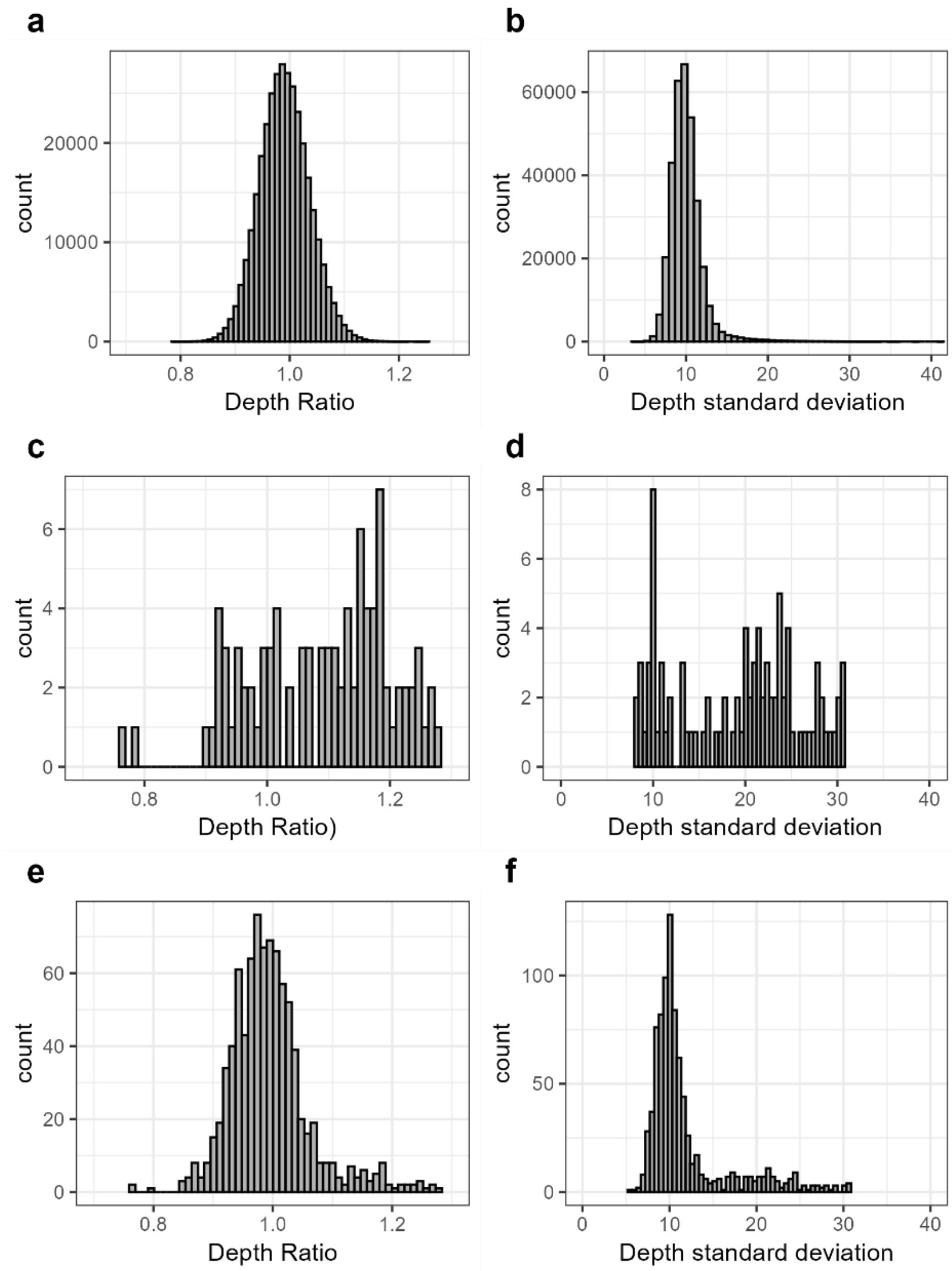

Supplementary Figure 19. Depth statistics for single nucleotide polymorphisms with significant *p*-values from Cochran-Mantel-Haenszel tests. Histograms showing, for different sets of single nucleotide polymorphism (SNP) sites called from the Stocks *et al.* [1] poolseq data mapped to the pangenome, i) the ratio of the mean depth of healthy pools vs. unhealthy pools (panels A, C, E), and ii) the standard deviation of read depths across all the pools. Panels indicate sets of SNPs - i) a random 1% of total SNPs (A, B), ii) 92 SNPs with a *p*-value $< 10^{-13}$ (C, D) and iii) 866 SNPs with a *p*-value passing Bonferroni correction with $\alpha = 0.05$ (E,F). Source data are provided as a Source Data file.

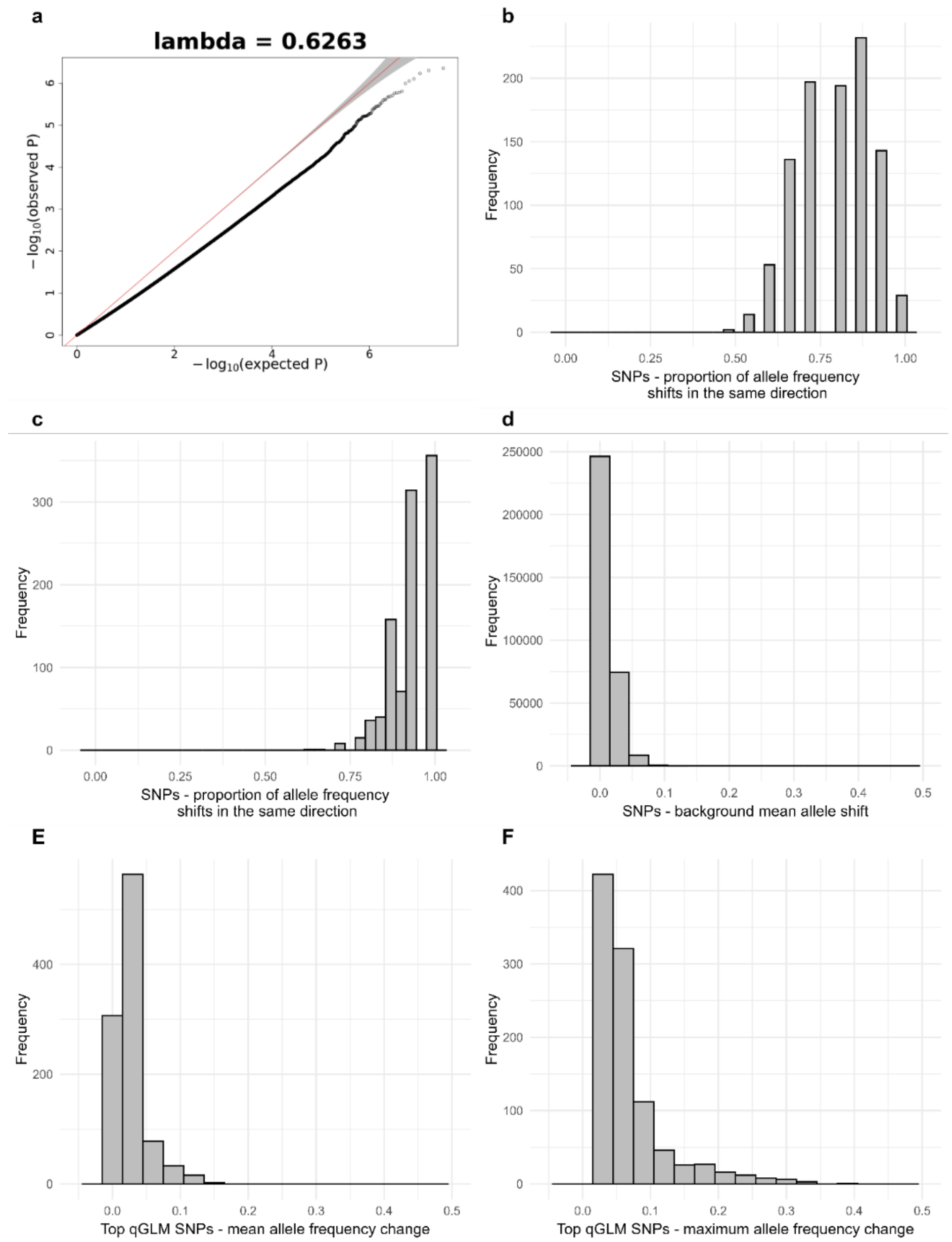

Supplementary Figure 20. Quasi-binomial generalized linear model test statistics for single nucleotide polymorphisms. A) quantile-quantile plots for -$\log_{10}$(*p*-values) from quasibinomial generalized-linear model (qGLM) tests from single nucleotide polymorphism (SNP) allele frequencies. Genomic inflation factor (lambda) is indicated at the top of each plot. B) Histogram indicating the proportion of allele frequency shifts occurring in the most frequent direction between healthy and unhealthy pools, including pairs of pools where the allele frequency shift is 0. C) Histogram indicating the proportion of allele frequency shifts occurring in the most frequent direction between healthy and unhealthy pools, excluding pairs of pools where the allele frequency shift is 0 D) For a random 1% of SNPs considered in the analysis, histogram of the mean absolute value of allele frequency shift between unhealthy and healthy pools. E + F) For the 1,000 SNPs with the smallest *p*-values (none of which were significant), histograms of the E) mean and F) maximum absolute value of allele frequency shift between unhealthy and healthy pools. Source data are provided as a Source Data file.

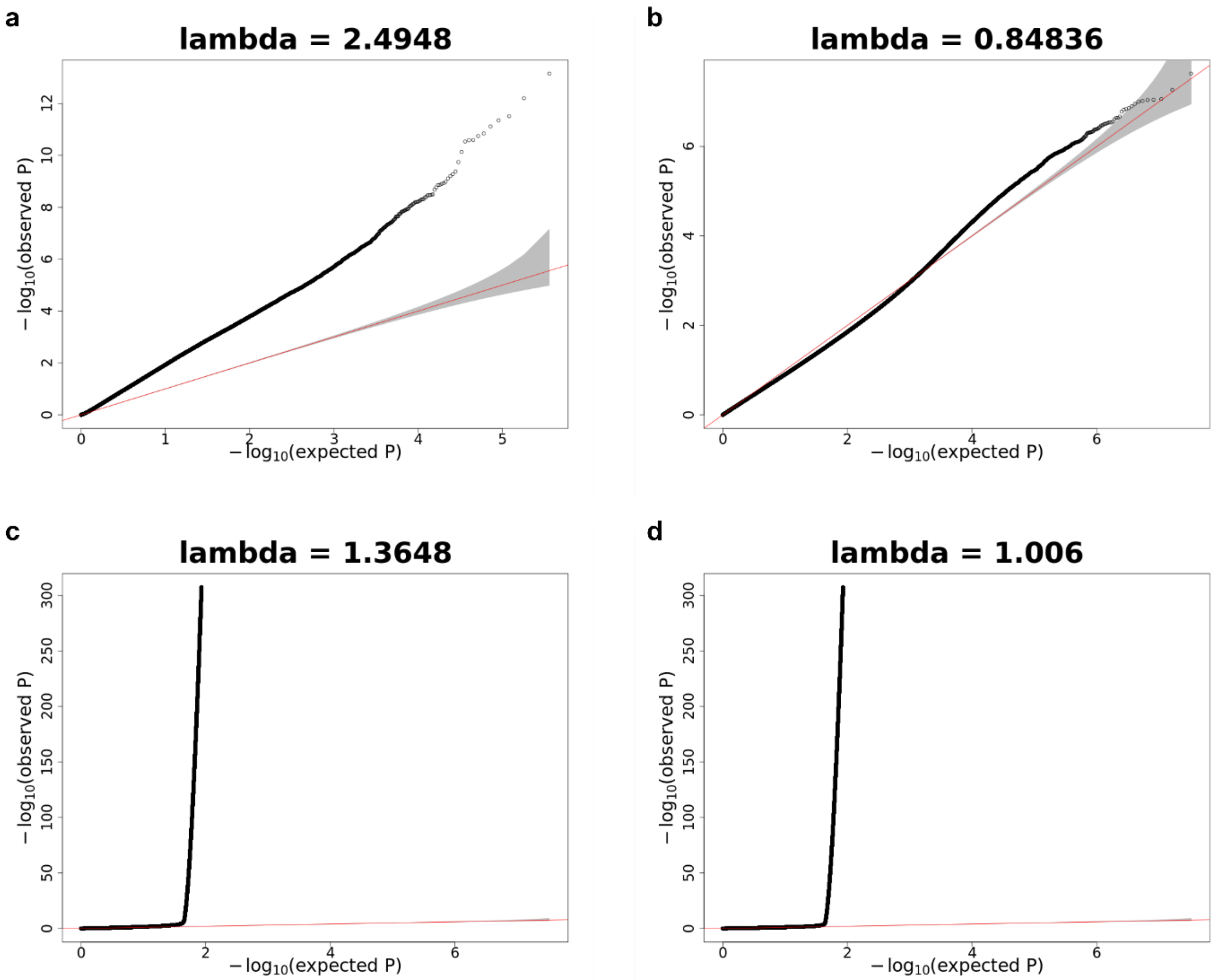

Supplementary Figure 21. Quantile-quantile plots for quasibinomial generalized linear model tests. Quantile-quantile plots for -log$_{10}$(*p*-values) from generalized-linear model (GLM) tests from single nucleotide polymorphism (SNP) allele frequencies. Genomic inflation factor (lambda) is indicated at the top of each plot. Panels indicate A) using seed source as a fixed effect in a quasibinomial GLM, B) using the first two principal components from principal component analysis of SNPs from the poolseq data as a fixed effect in a quasibinomial GLM, C) Using seed source as a random effect in a binomial GLM, and D) Using seed source and individual pool identity as random effects in a binomial GLM. Source data are provided as a Source Data file.

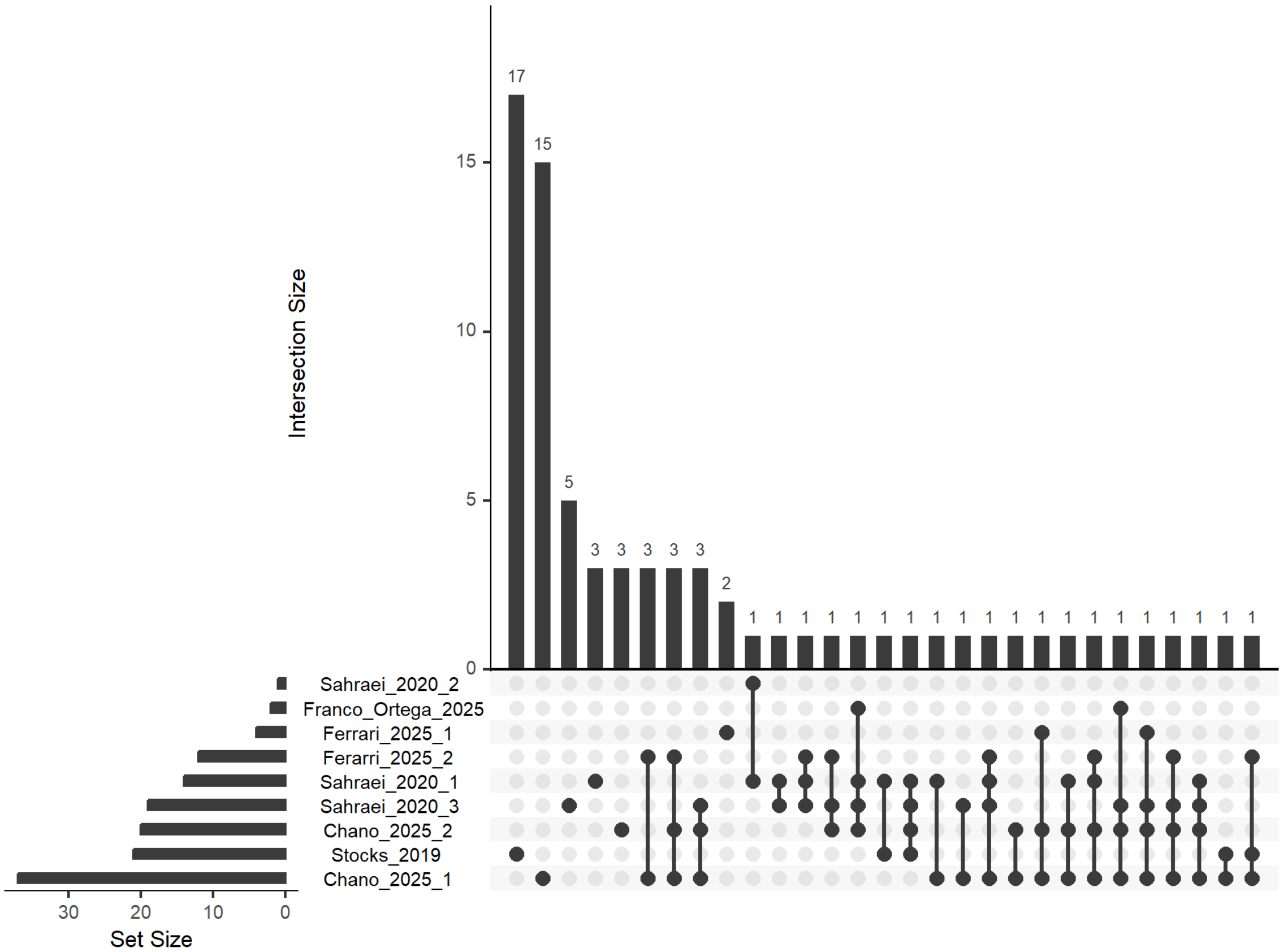

Supplementary Figure 22. Overlapping gene clusters across datasets. Upset plot for subsets of genes from genomic/transcriptomic studies identifying genes associated with low ash dieback susceptibility, or the response to ash dieback infection, that are in the same 206 gene clusters containing the 211 candidate genes identified in this study. Set size panel corresponds to the total number of genes in each subset (i.e. the total overlap between genes identified in that study, and the current study). In the intersection size panel, single dots correspond to genes unique to that subset that intersect with genes in this study, lines connecting dots represent genes found in multiple subsets that intersect with genes in this study. Subsets are as follows: Overlap with genes identified from Chano *et al.*[16]; differentially expressed genes (DEGs) between infected and uninfected petiole tissue in i) a low susceptibility genotype (Chano_2025_1) and ii) a high susceptibility genotype (Chano_2025_2); Overlap with genes identified from Ferrari *et al.*[17]; DEGs between infected and uninfected petiole tissue in i) a low susceptibility genotype (Ferrari_2025_1) and ii) a high susceptibility genotype (Ferrari_2025_2); Overlap with DEGs from Franco-Ortega *et al.*[7] between leaf tissue in low susceptibility and high susceptibility genotypes (Franco_Ortega_2025); Overlap with DEGs from Sahraei *et al.*[18]; i) expressed in distant uninfected bark tissue following artificial inoculation between low and high susceptibility genotypes (Sahraei_2020_1), ii) between lesions in low vs. high susceptibility genotypes (Sahraei_2020_2), and iii) between infected lesions following artificial inoculation, and distant uninfected bark tissue (Sahraei_2020_3); Genes within 5 kilobases either up or downstream of a SNP identified in Stocks *et al.*[1]. Source data are provided as a Source Data file.

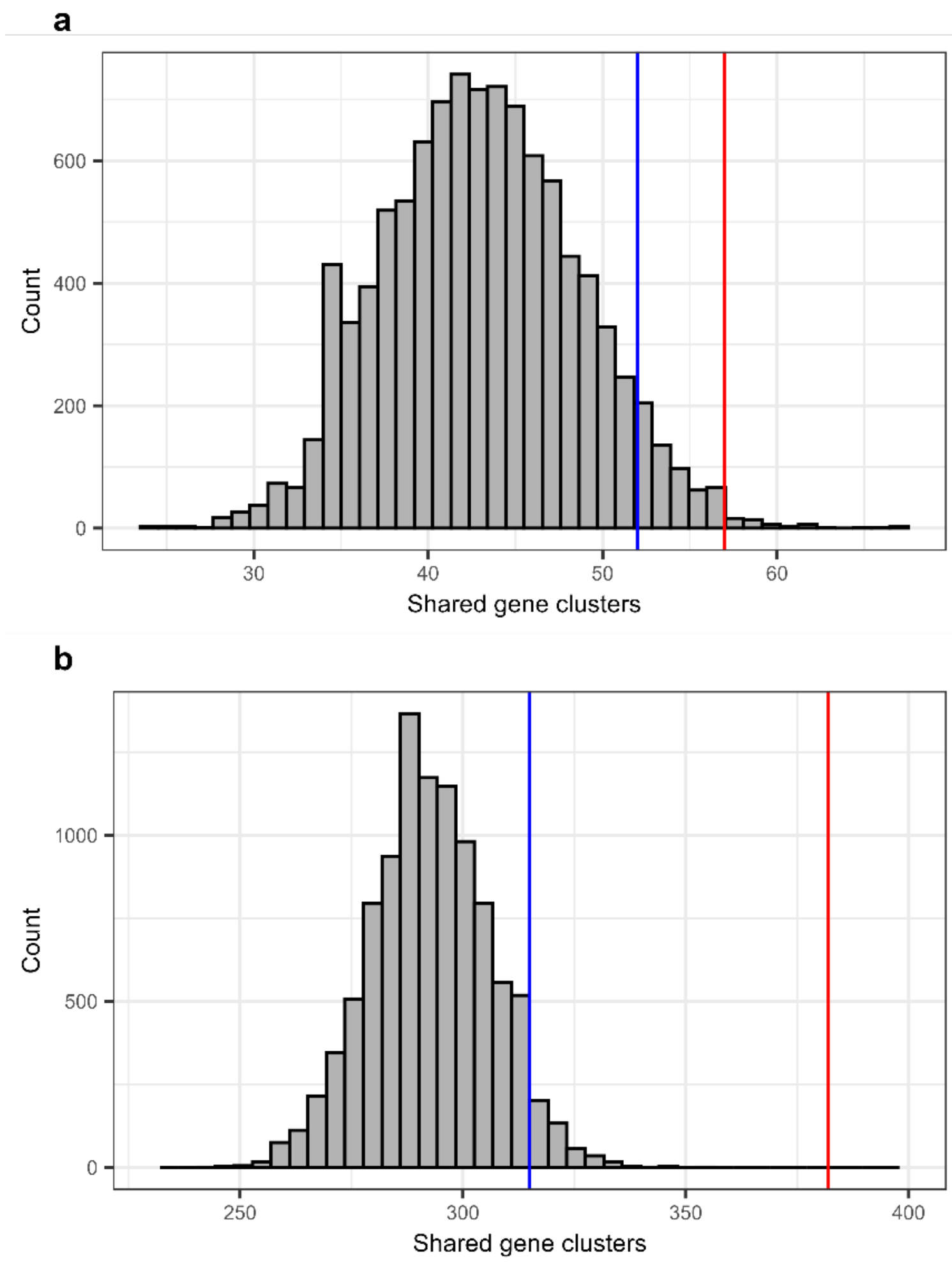

Supplementary Figure 23. Observed vs. expected overlaps in gene clusters. A total of 3,655 gene clusters (Orthogroups) from the OrthoFinder[8] run in this study contained sequence matches to genes associated with ash dieback from a number of studies (see Methods for details). A) 10,000 sets of 3,655 gene clusters were randomly selected, and how many of the 206 clusters containing 211 genes within 10 kilobases of the 220 candidate single nucleotide polymorphisms (SNPs) identified in this study that overlap with these was counted, displayed in a histogram. Blue line represents 95th percentile, red line represents how many of the 206 clusters overlapped with the non-random 3,655 genes (n=57). B) 10,000 sets of 3,655 genes were randomly selected, and the frequency with which these occurred in clusters containing dispensable genes plotted in a histogram. Blue line represents 95th percentile, red line represents how many of the non-random 3,655 gene clusters contained dispensable genes (n=382). Source data are provided as a Source Data file.

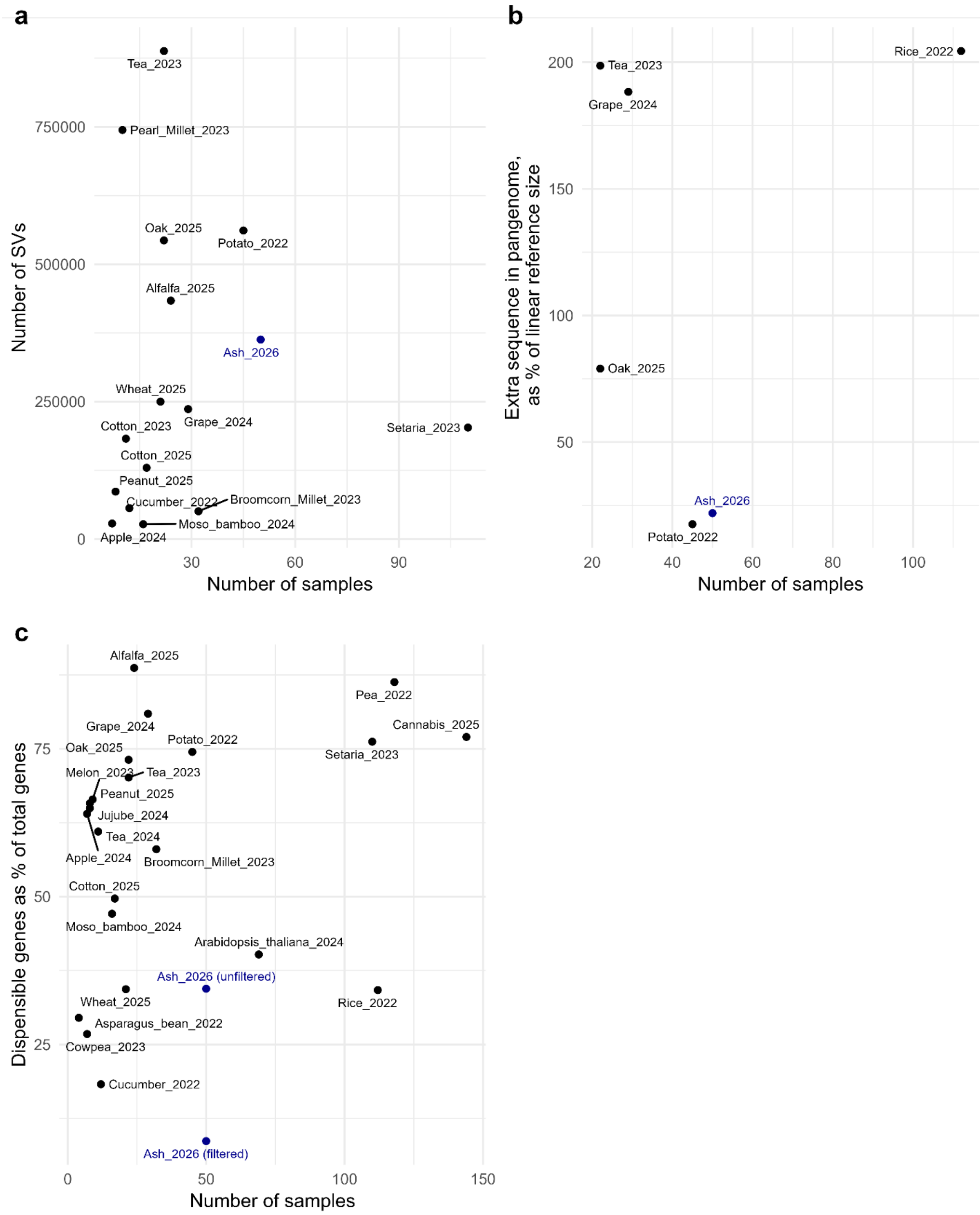

Supplementary Figure 24. Comparison of key statistics with other recent pangenome studies. Results from the present study compared with a selection of plant pangenome studies published since 2022; pangenome studies using primarily short-read data, or incorporating multiple species, were excluded. Labels correspond to study organism and publication date, detailed in Supplementary Data File 12. Number of samples included in the pangenome is on the X axis. Y axis numbers correspond to total number of SVs discovered (A), additional sequence included in the pangenome, as a percentage of the reference genome size of that species (B), or the number dispensable genes as a proportion of the total number of genes in the pangenome. Results from the present study are in blue; in panel C, Ash_2026 (unfiltered) refers to the proportion of dispensable genes prior to filtering, Ash_2026 (filtered) refers to the proportion after filtering for consistent association with SVs - see Results for further details. Source data are provided as a Source Data file.

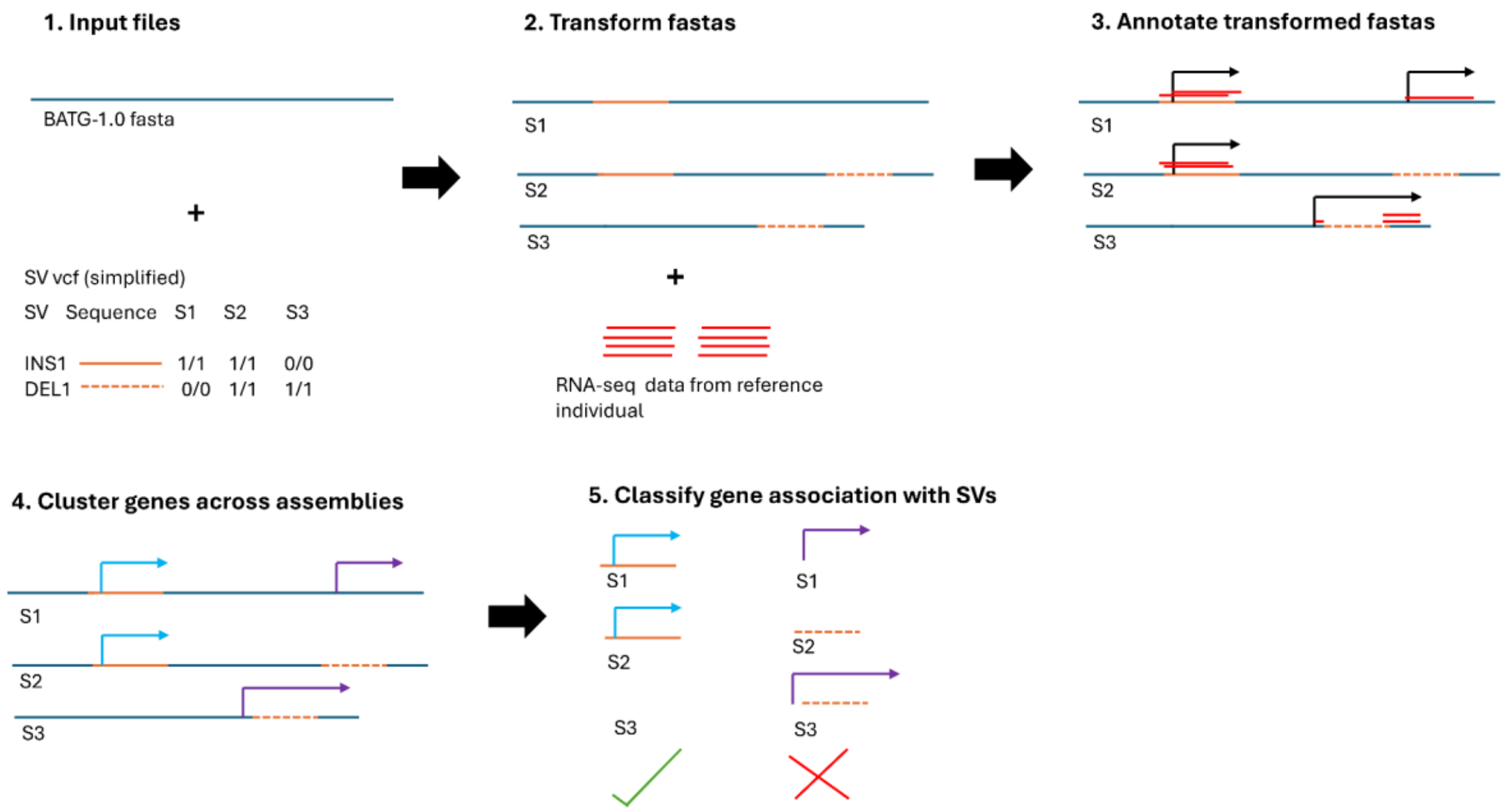

Supplementary Figure 25. Explanatory diagram for the pangenome annotation. Explanatory diagram for annotation of the pangenome - see Methods section Linear reference and pangenome annotation for full description. For the pangenome annotation, the BATG-1.0 linear reference was transformed using the vcf file of SV calls from each of the 50 ONT samples (1 - S1, S2 and S3 correspond to 3 example samples). For each sample, calls from the vcf used to transform the BATG-1.0 fasta; where an insertion was called, the consensus insertion sequence was inserted at the appropriate position, for deletions the relevant sequence from the BATG-1.0 fasta was deleted, for inversions the sequence was inverted. This resulted in a transformed fasta for each sample (2). Using the RNA-seq data from the reference individual, each of the transformed fasta files was annotated (3). Based on sequence homology and position in the genome, genes were clustered across each of the transformed fasta files (4). For each gene cluster, the strength of association of genes with each SV was assessed (5): genes that were consistently associated with an SV were kept as variable genes, whereas those not showing a consistent pattern with an overlapping SV were not. Source data are provided as a Source Data file.

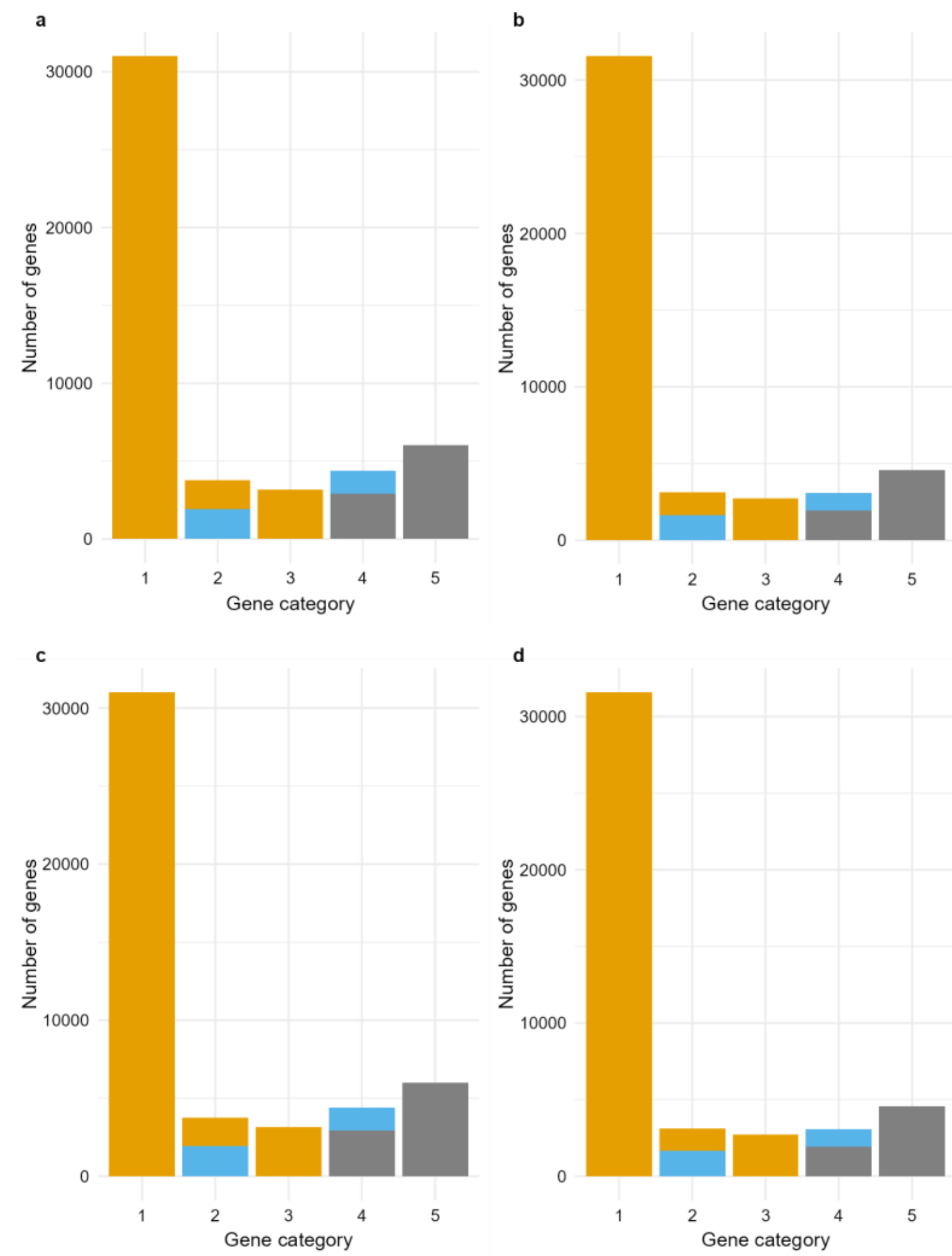

Supplementary Figure 26. The impact of different annotation merging approaches on genes classified as dispensable or indispensable. Barplots of the number of genes in each category in the pangenome, where numbers correspond to genes present in the BATG-1.0 reference (1, 2 and 3) and in every other sample (1), or absent in some samples and overlapping with a structural variant (SV; 2), or (3) absent in some samples, but none of these overlap with an SV, or genes that are absent from the BATG-1.0 reference (4,5) and either overlap with an SV (4) or never overlap with an SV (5). Bar colours are based on reclassification of genes in each category as invariant (orange), dispensable (blue), or discarded from the analysis (grey) according to how consistently they overlap with SVs based on an F1 score. Genes in category 2 with an F1 score > 0.8 are classified as dispensable (blue); those with a score below that threshold are categorised as invariant. Genes in category 3 are never associated with an SV so are all categorised as invariant. Genes in category 4 with an F1 score > 0.8 are classified as dispensable, those with a score <= 0.8 are discarded from the analysis. Genes in category 5 are never associated with an SV so are discarded from the analysis. In an approach where genes absent in some individuals are treated as dispensable, all the genes in categories 2-5 would be treated as dispensable genes. Panels correspond to analysis performed where i) genes in BATG-1.0 that overlapped by >50% in a coding sequence on the same strand had the shortest gene removed (A and B), or not (C and D), and ii) Orthogroups producing during the annotation of the pangenome grouped together if any two members had a >50% of a coding sequence overlapping on the same strand (B and D), or not (A and C). Source data are provided as a Source Data file.

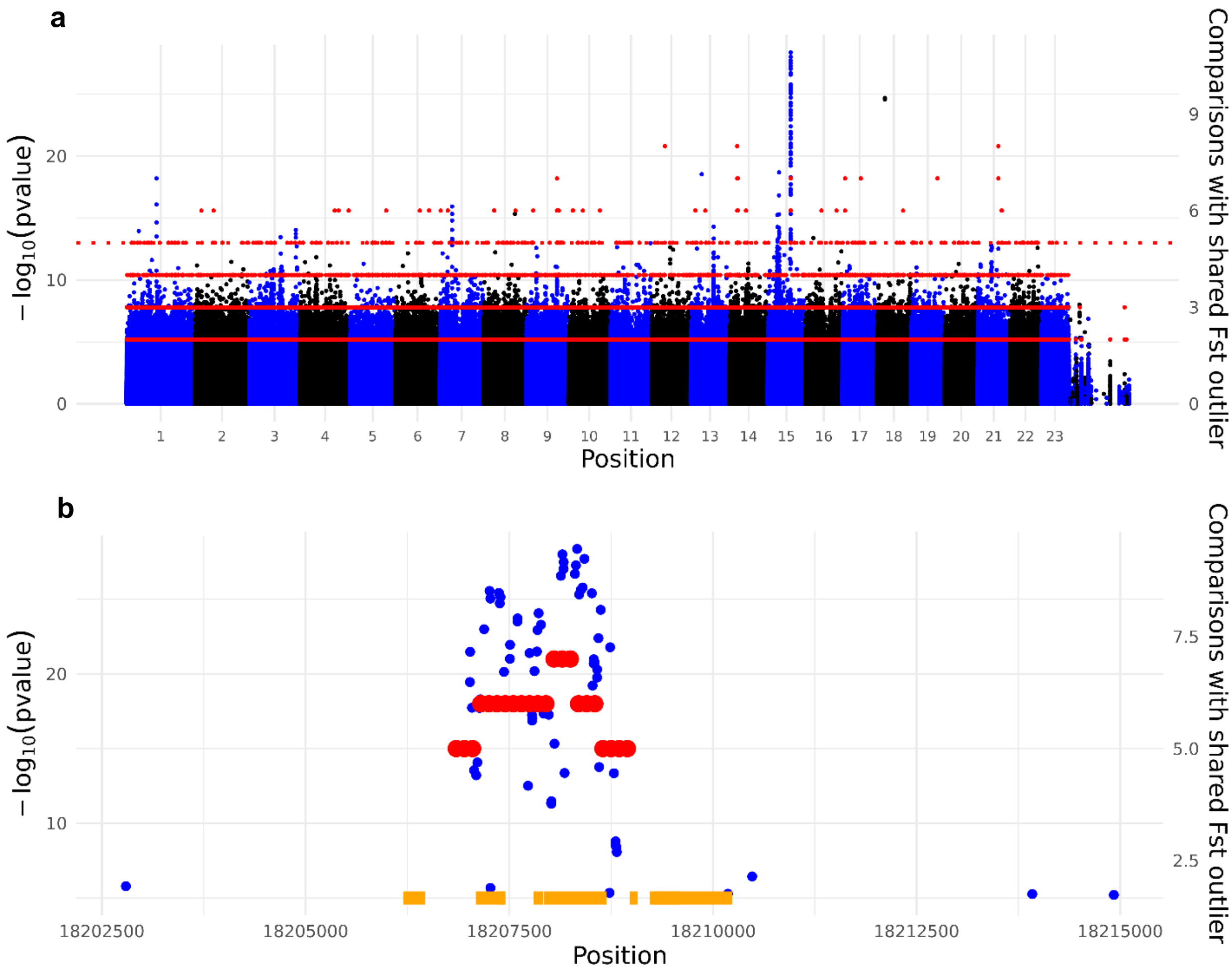

Supplementary Figure 27. Putative outlier region on chromosome 15. A) Association of single nucleotide polymorphisms (SNPs) called from poolseq data from Stocks *et al.*[1], with ash dieback disease damage. Sites are ordered by position along scaffolds in the BATG-1.0 genome assembly. Labels for the 23 largest scaffolds are shown. Black and blue coloured points represent the -$\log_{10}$ *p*-values of a Cochran-Mantel Haenszel (CMH) test, with alternating colours representing the different scaffolds and values indicated by the left hand Y axis. Red points indicate the number of 500bp sliding windows sharing an F_{st} window in the top 1% of values for each chromosome, shared by a minimum number of comparisons as indicated on the right hand Y axis (i.e. 5 corresponds to 5 comparisons having an Fst value in the top 1%; axes are scaled so that 5 on the right hand axis corresponds to -$\log_{10}$ (p) = 13 on the left hand axis). B) Figure A, zoomed in on the region on chromosome 15 representing a cluster of 55/92 CMH test values < 1 x 10^{-13}. Orange bars represent annotated repeats overlapping the region. Source data are provided as a Source Data file.

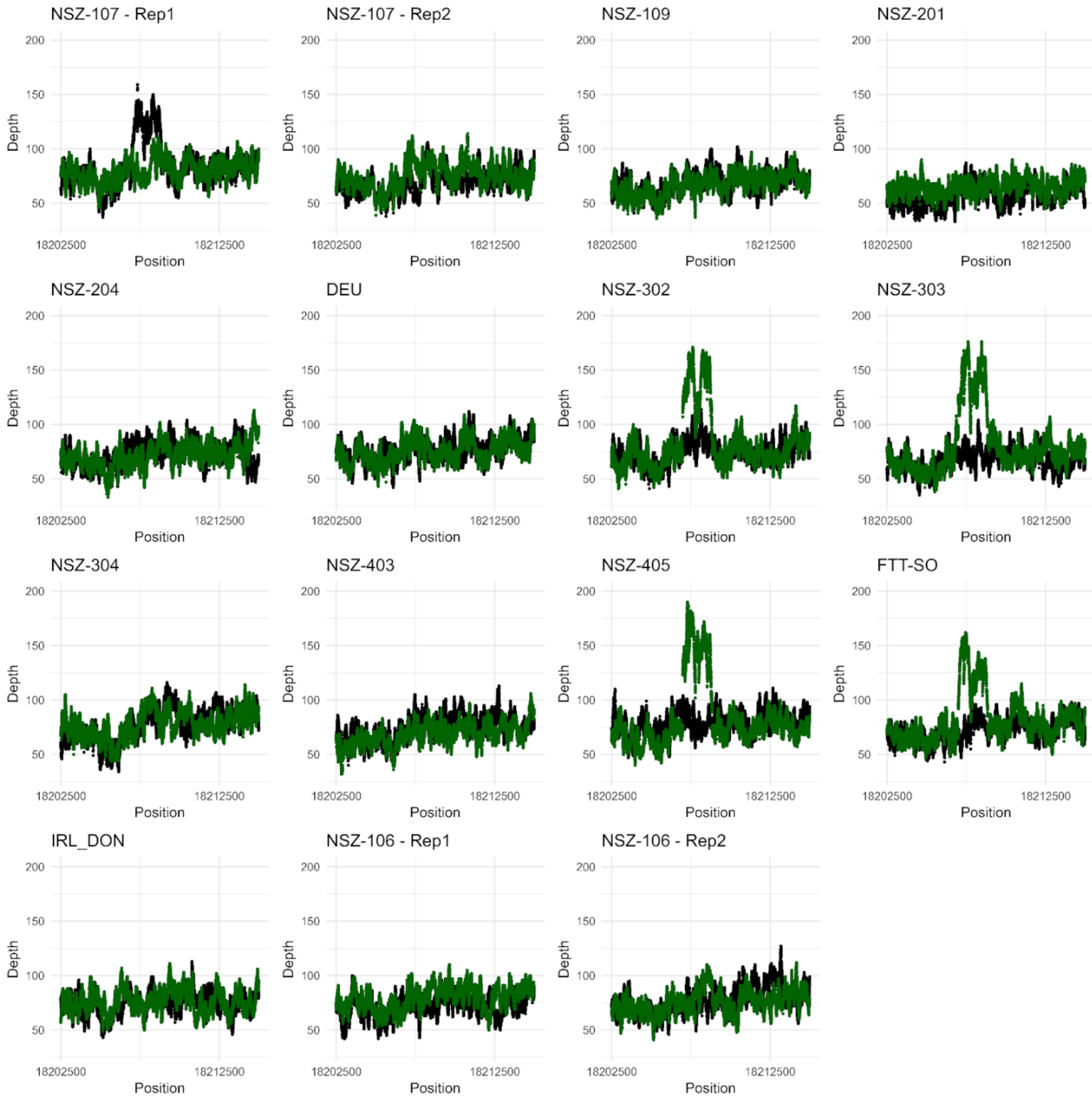

Supplementary Figure 28. Read depths across chromosome 15 outlier region. Total read depths of reads for each of the 15 pairs of pools, surrounding the region with overlapping significant single nucleotide polymorphisms (SNPs) with significant Cochran-Mantel Haenszel test *p*-values and fixation index (F_{st}) outlier windows on Chromosome 15. Green points represent depths in the healthy pools; black the unhealthy pools. Biological replicates from National Seed Zone (NSZ)-107 and NSZ-106 are plotted separately. See Stocks *et al.*[1] for explanation of pool names. Source data are provided as a Source Data file.

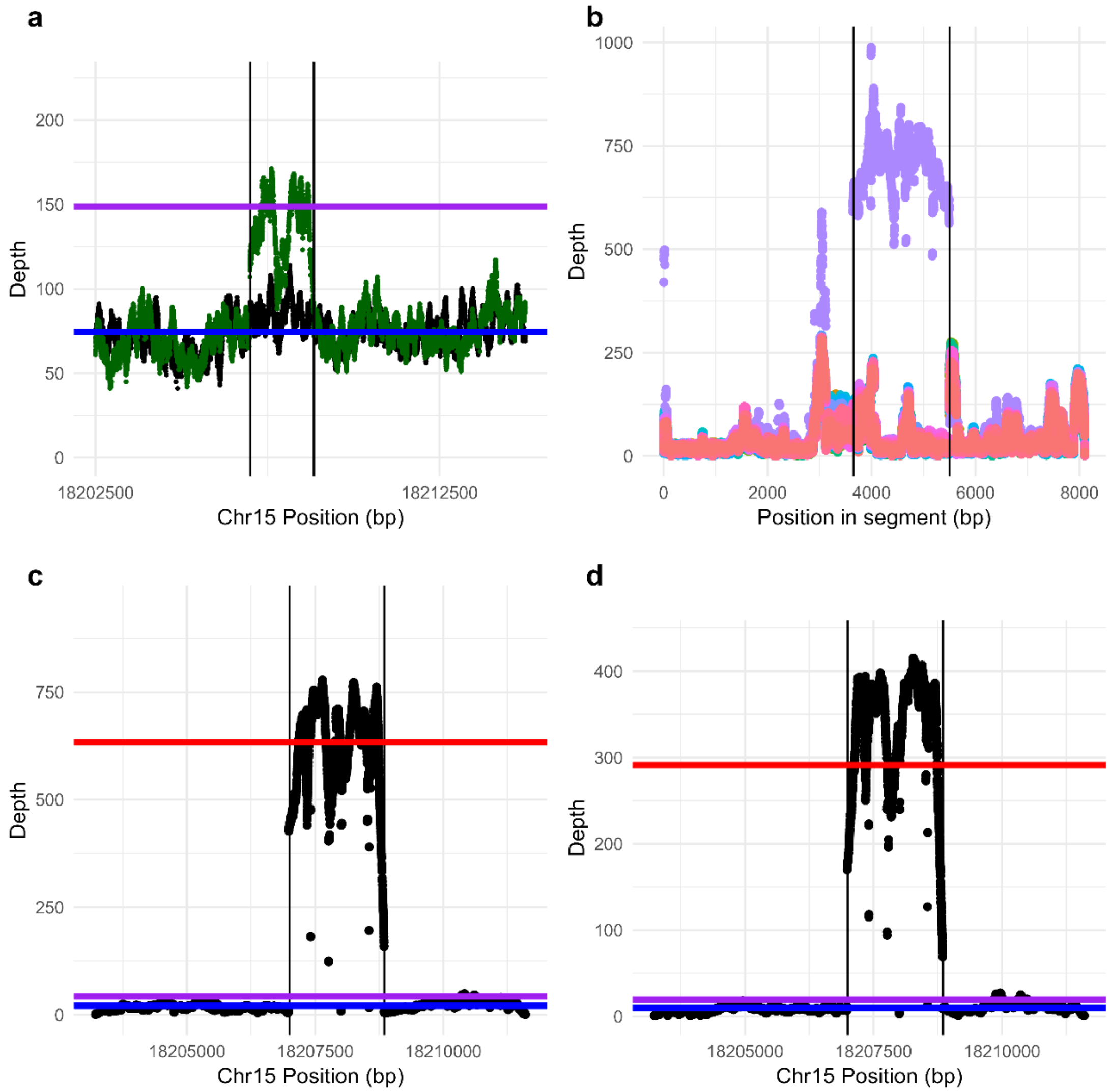

Supplementary Figure 29. Read depths across chromosome 15 outlier region from individually sequenced samples. Read depth from reads mapped to the pangenome in the region of the chromosome 15 outlier region for National Seed Zone (NSZ)-302 poolseq data from healthy pool (green) and unhealthy pool (black). B) Read depths from reads from 150 individually sequenced samples from Stocks *et al.*[1] mapped to a segment corresponding to the high depth region in A, and 3kb upstream and downstream of this. Depth for each individual is presented in a different colour. Panels C and D represent read depths from Individual 58 from Stocks *et al.* [1] (C), and Individual S178C from Metheringham *et al.*[3] (D). Vertical lines represent the boundary of the outlier region. Horizontal lines in panels A, C and D represent multiple of the median depth of the flanking region outside the outlier region, for each panel (+/- 3kb) . blue = 1X, purple = 2X, red = 30X. Source data are provided as a Source Data file.

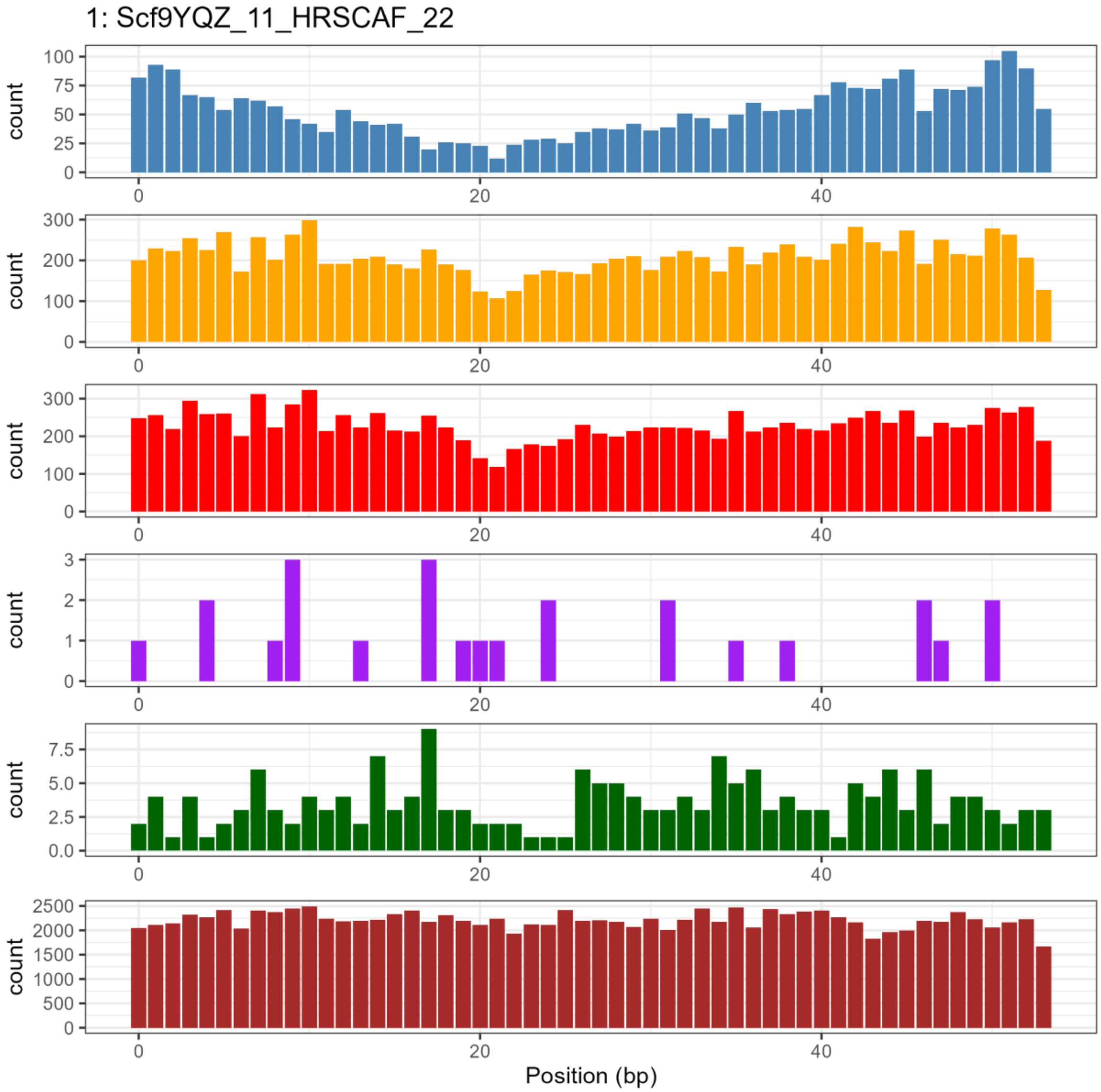

Supplementary Figure 30. Chromosome 1 feature plots. Features of the largest pseudochromosomal scaffold (Scf9YQZ_11_HRSCAF_22) of BATG-1.0. Each panel represents a feature plotted by density per 100kb (Y axis), along the chromosome (x axis). Panels are, from top to bottom, i) genes (blue), ii) insertions (orange), iii) deletions (red), iv) inversions (purple), v) tandem duplications (green) and vi) repeats (brown). Source data are provided as a Source Data file.

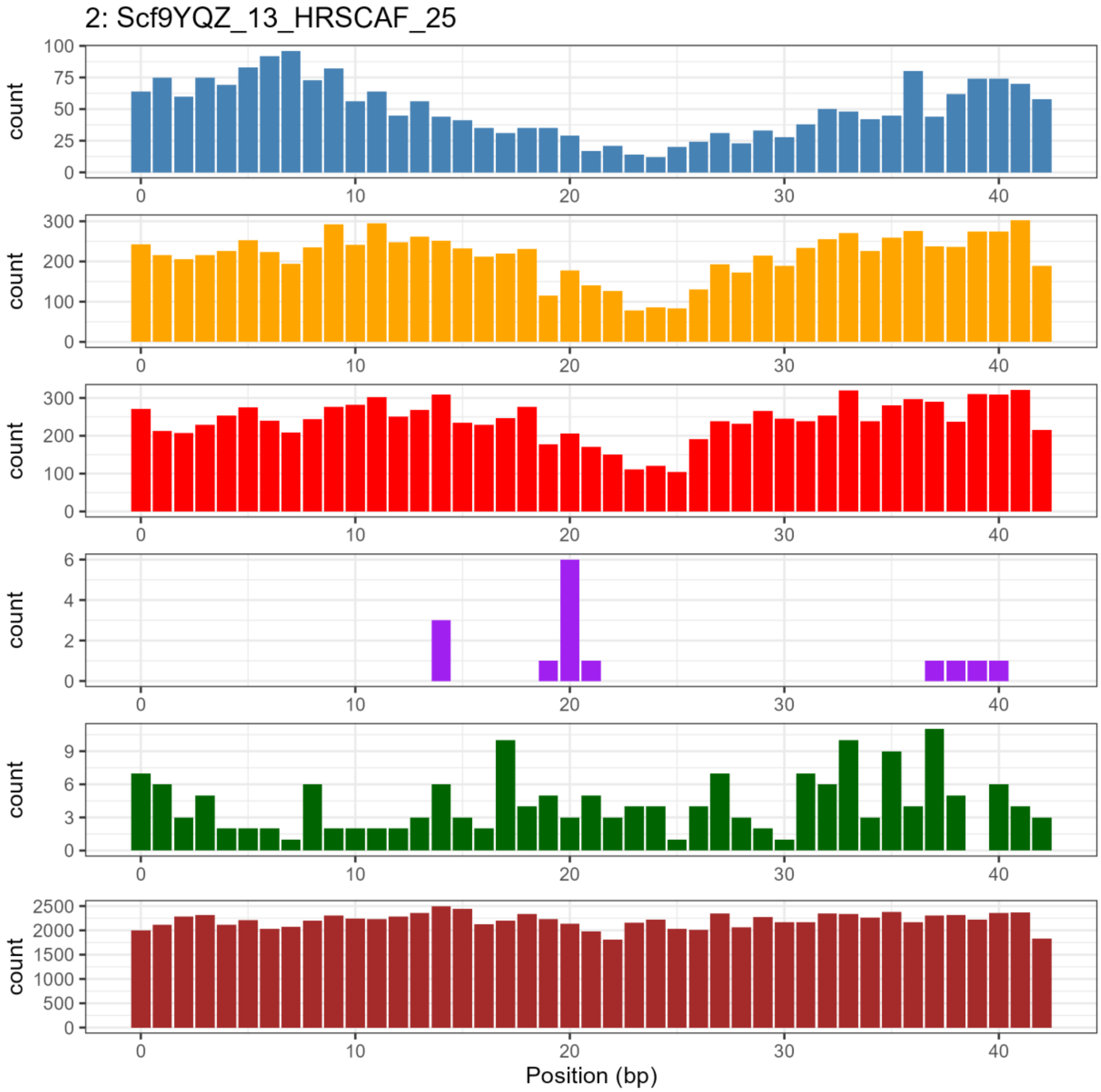

Supplementary Figure 31. Chromosome 2 feature plots. Features of the second-largest pseudochromosomal scaffold (Scf9YQZ_13_HRSCAF_25) of BATG-1.0. Each panel represents a feature plotted by density per 100kb (Y axis), along the chromosome (x axis). Panels are, from top to bottom, i) genes (blue), ii) insertions (orange), iii) deletions (red), iv) inversions (purple), v) tandem duplications (green) and vi) repeats (brown). Source data are provided as a Source Data file.

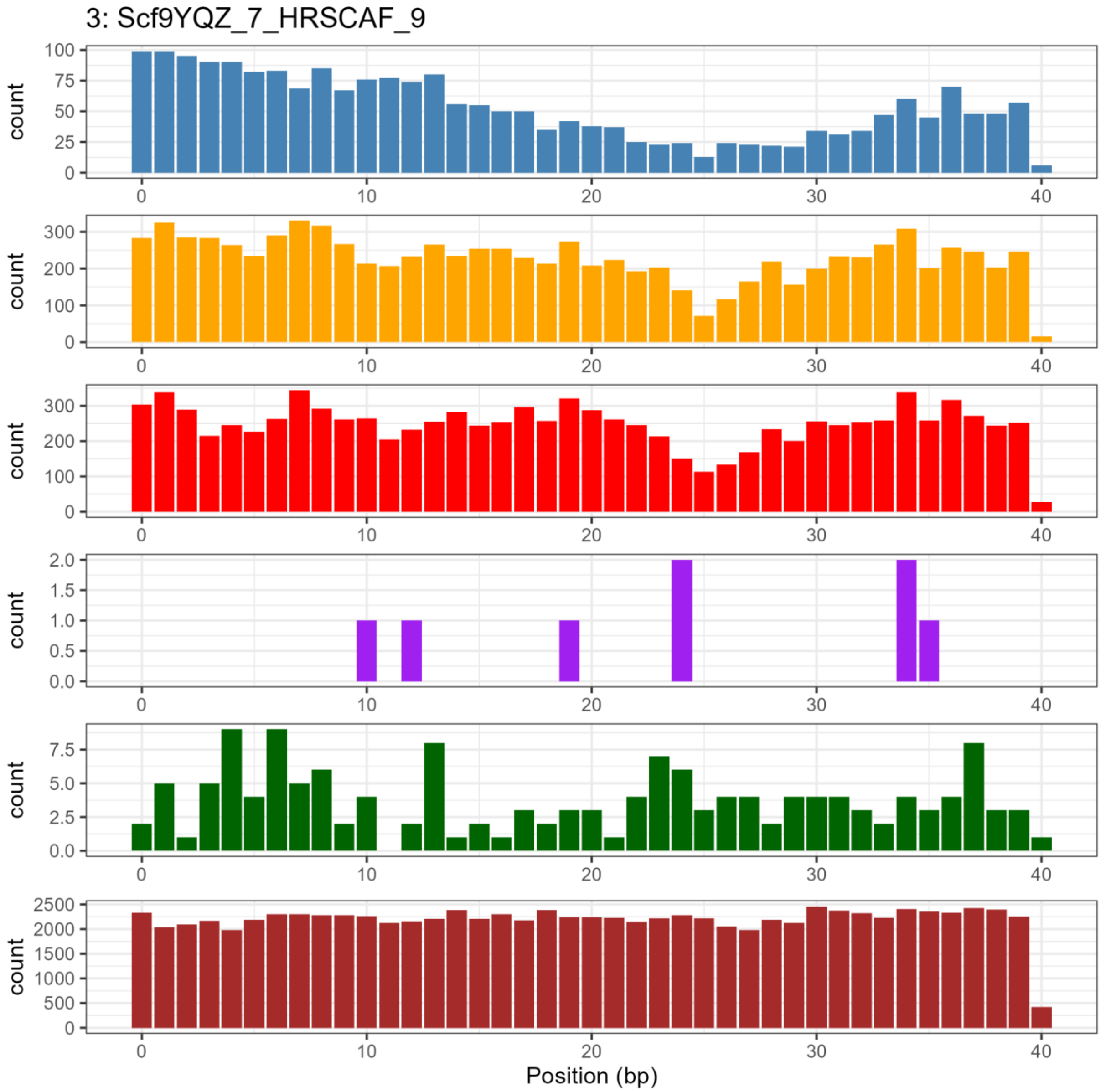

Supplementary Figure 32. Chromosome 3 feature plots. Features of the third-largest pseudochromosomal scaffold (Scf9YQZ_7_HRSCAF_9) of BATG-1.0. Each panel represents a feature plotted by density per 100kb (Y axis), along the chromosome (x axis). Panels are, from top to bottom, i) genes (blue), ii) insertions (orange), iii) deletions (red), iv) inversions (purple), v) tandem duplications (green) and vi) repeats (brown). Source data are provided as a Source Data file.

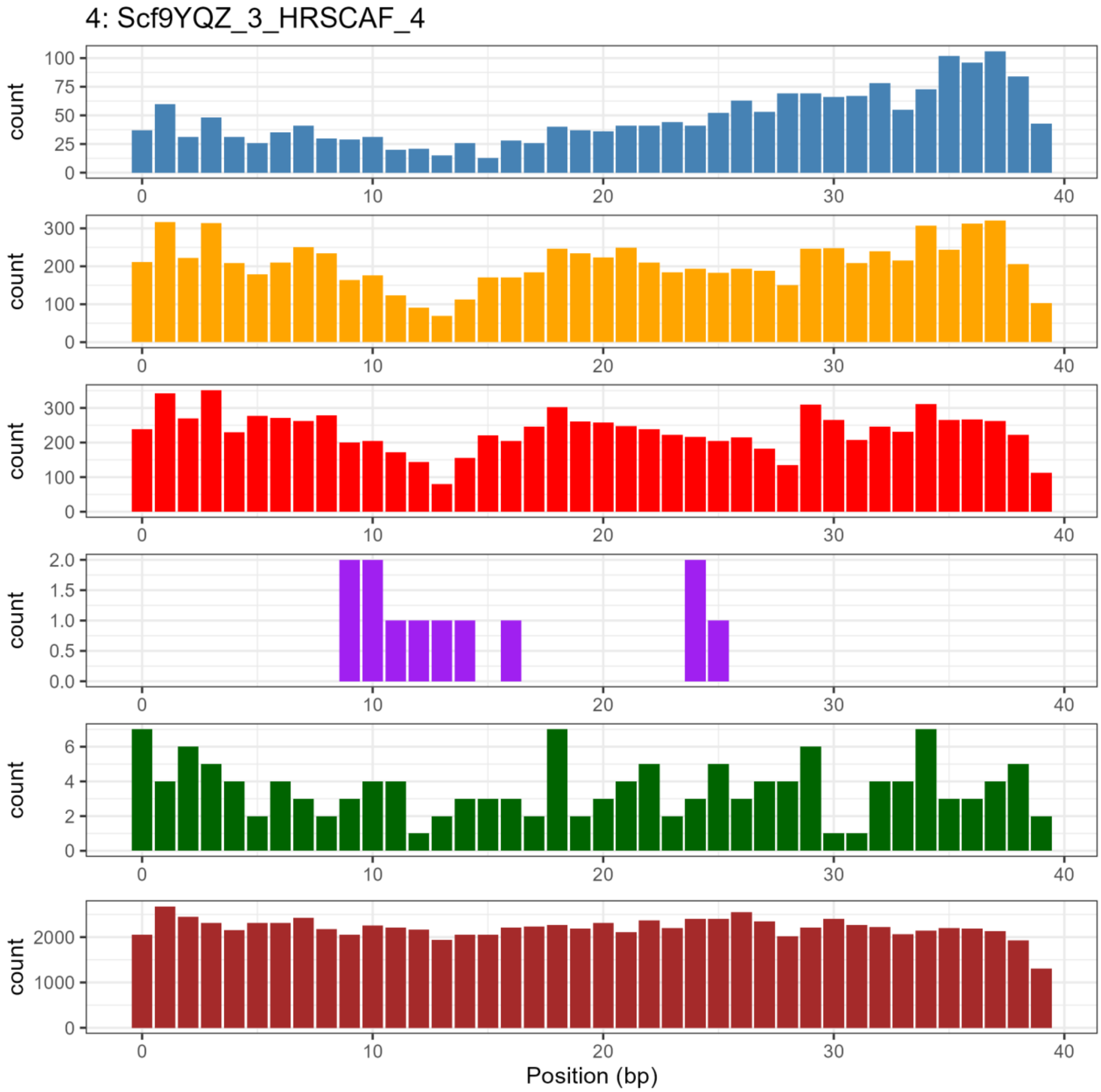

Supplementary Figure 33. Chromosome 4 feature plots. Features of the fourth-largest pseudochromosomal scaffold (Scf9YQZ_3_HRSCAF_4) of BATG-1.0. Each panel represents a feature plotted by density per 100kb (Y axis), along the chromosome (x axis). Panels are, from top to bottom, i) genes (blue), ii) insertions (orange), iii) deletions (red), iv) inversions (purple), v) tandem duplications (green) and vi) repeats (brown). Source data are provided as a Source Data file.

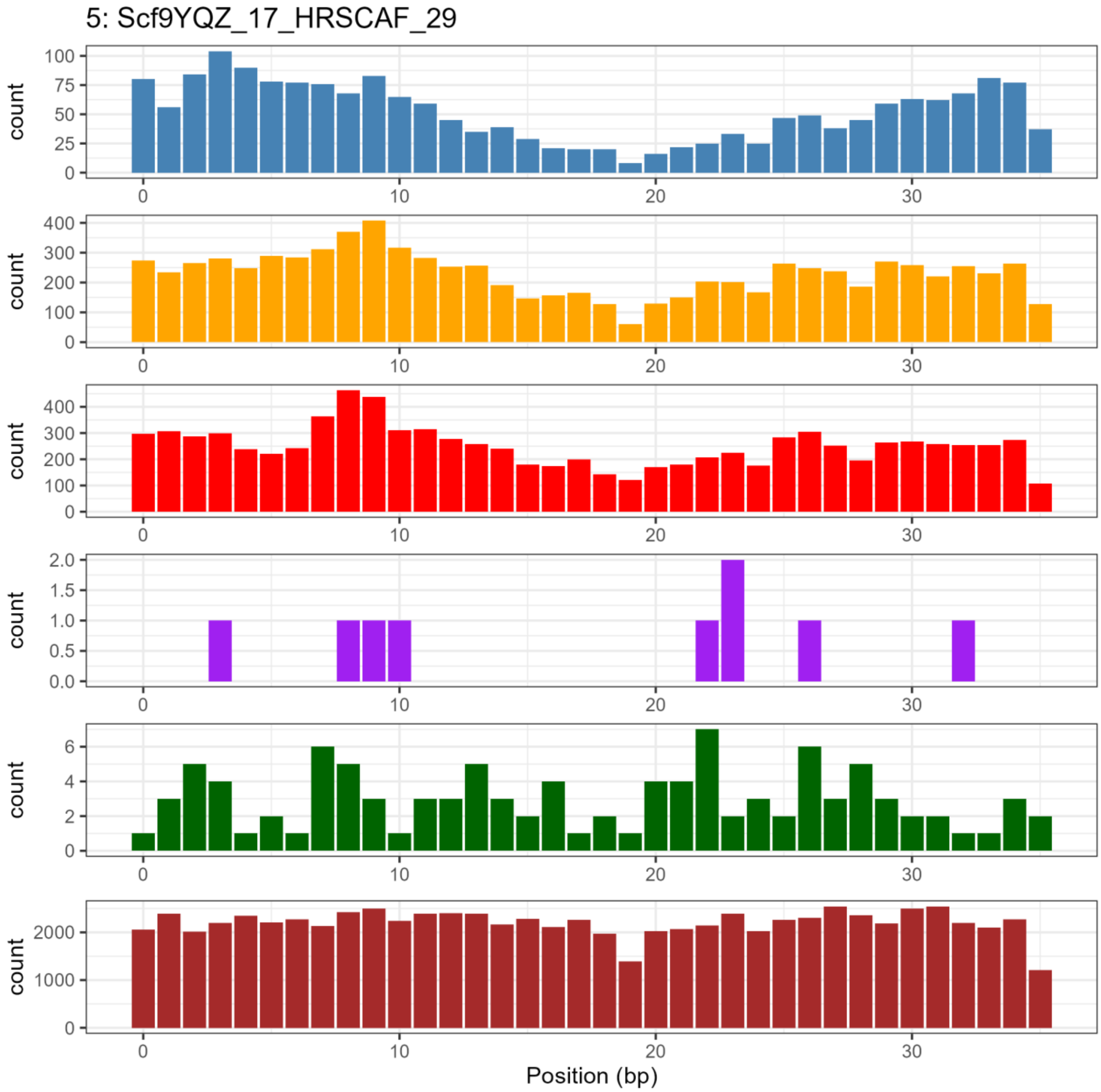

Supplementary Figure 34. Chromosome 5 feature plots. Features of the fifth-largest pseudochromosomal scaffold (Scf9YQZ_17_HRSCAF_29) of BATG-1.0. Each panel represents a feature plotted by density per 100kb (Y axis), along the chromosome (x axis). Panels are, from top to bottom, i) genes (blue), ii) insertions (orange), iii) deletions (red), iv) inversions (purple), v) tandem duplications (green) and vi) repeats (brown). Source data are provided as a Source Data file.

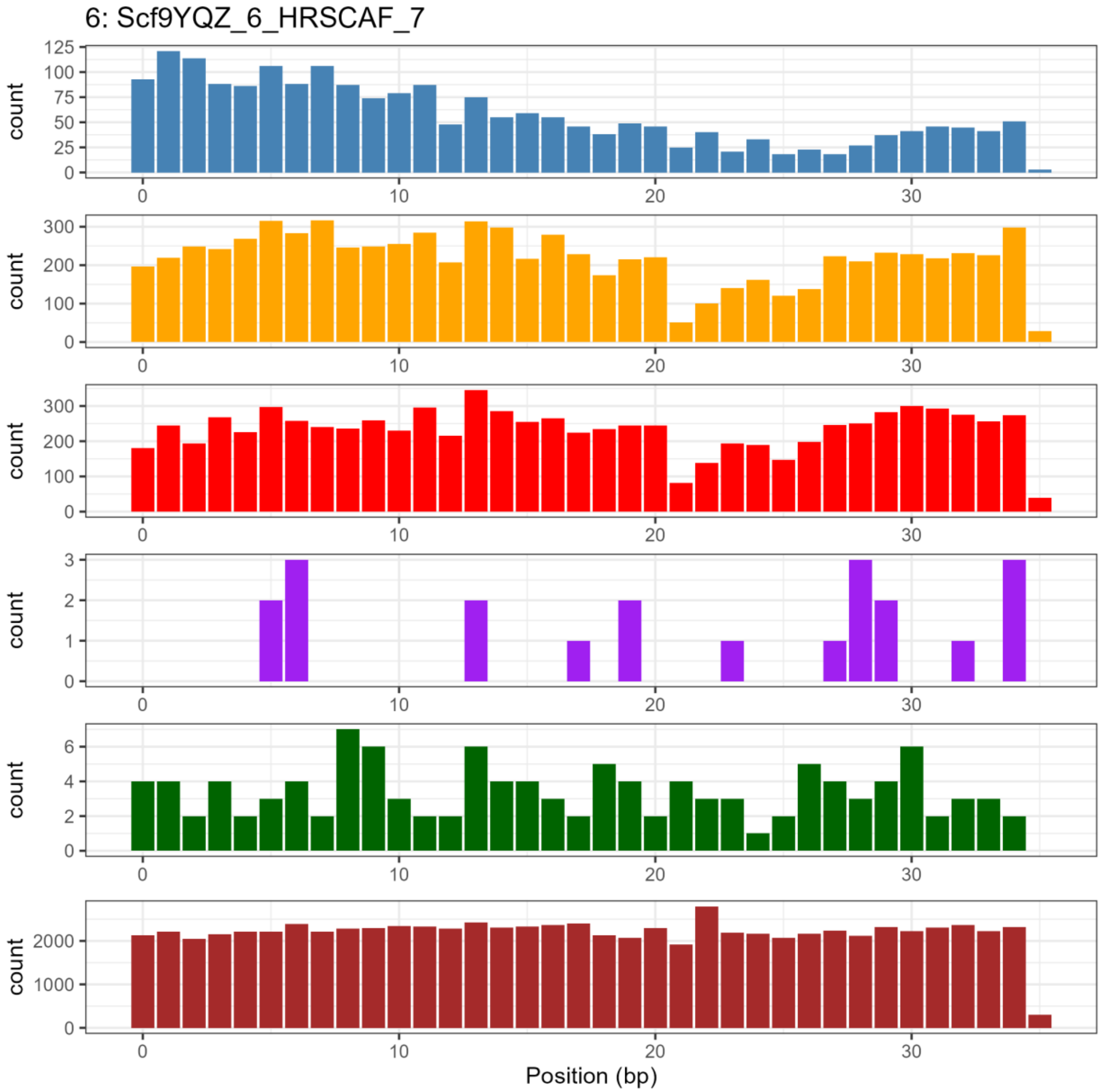

Supplementary Figure 35. Chromosome 6 feature plots. Features of the sixth-largest pseudochromosomal scaffold (Scf9YQZ_6_HRSCAF_7) of BATG-1.0. Each panel represents a feature plotted by density per 100kb (Y axis), along the chromosome (x axis). Panels are, from top to bottom, i) genes (blue), ii) insertions (orange), iii) deletions (red), iv) inversions (purple), v) tandem duplications (green) and vi) repeats (brown). Source data are provided as a Source Data file.

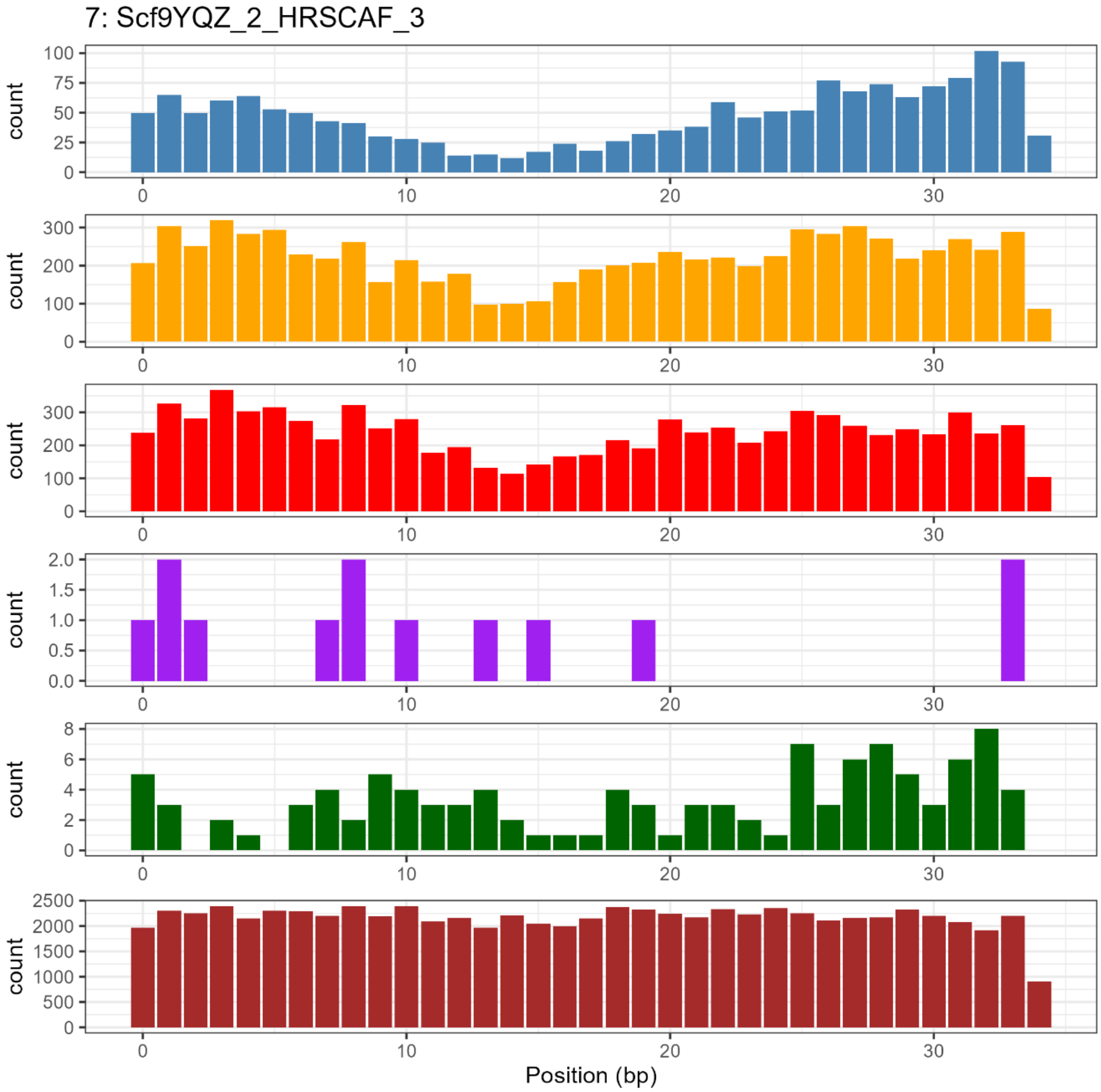

Supplementary Figure 36. Chromosome 7 feature plots. Features of the seventh-largest pseudochromosomal scaffold (Scf9YQZ_2_HRSCAF_3) of BATG-1.0. Each panel represents a feature plotted by density per 100kb (Y axis), along the chromosome (x axis). Panels are, from top to bottom, i) genes (blue), ii) insertions (orange), iii) deletions (red), iv) inversions (purple), v) tandem duplications (green) and vi) repeats (brown). Source data are provided as a Source Data file.

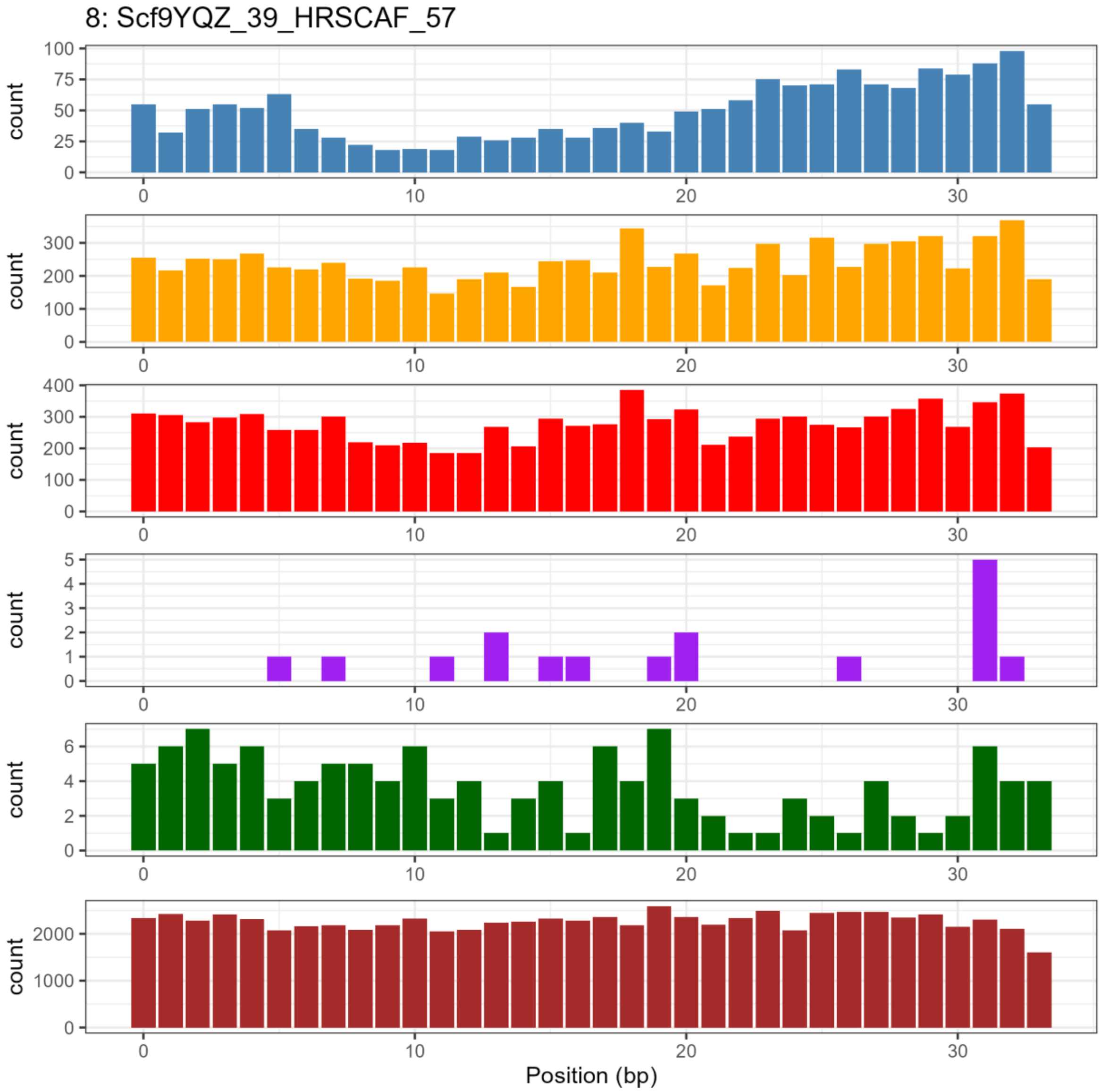

Supplementary Figure 37. Chromosome 8 feature plots. Features of the eighth-largest pseudochromosomal scaffold (Scf9YQZ_39_HRSCAF_57) of BATG-1.0. Each panel represents a feature plotted by density per 100kb (Y axis), along the chromosome (x axis). Panels are, from top to bottom, i) genes (blue), ii) insertions (orange), iii) deletions (red), iv) inversions (purple), v) tandem duplications (green) and vi) repeats (brown). Source data are provided as a Source Data file.

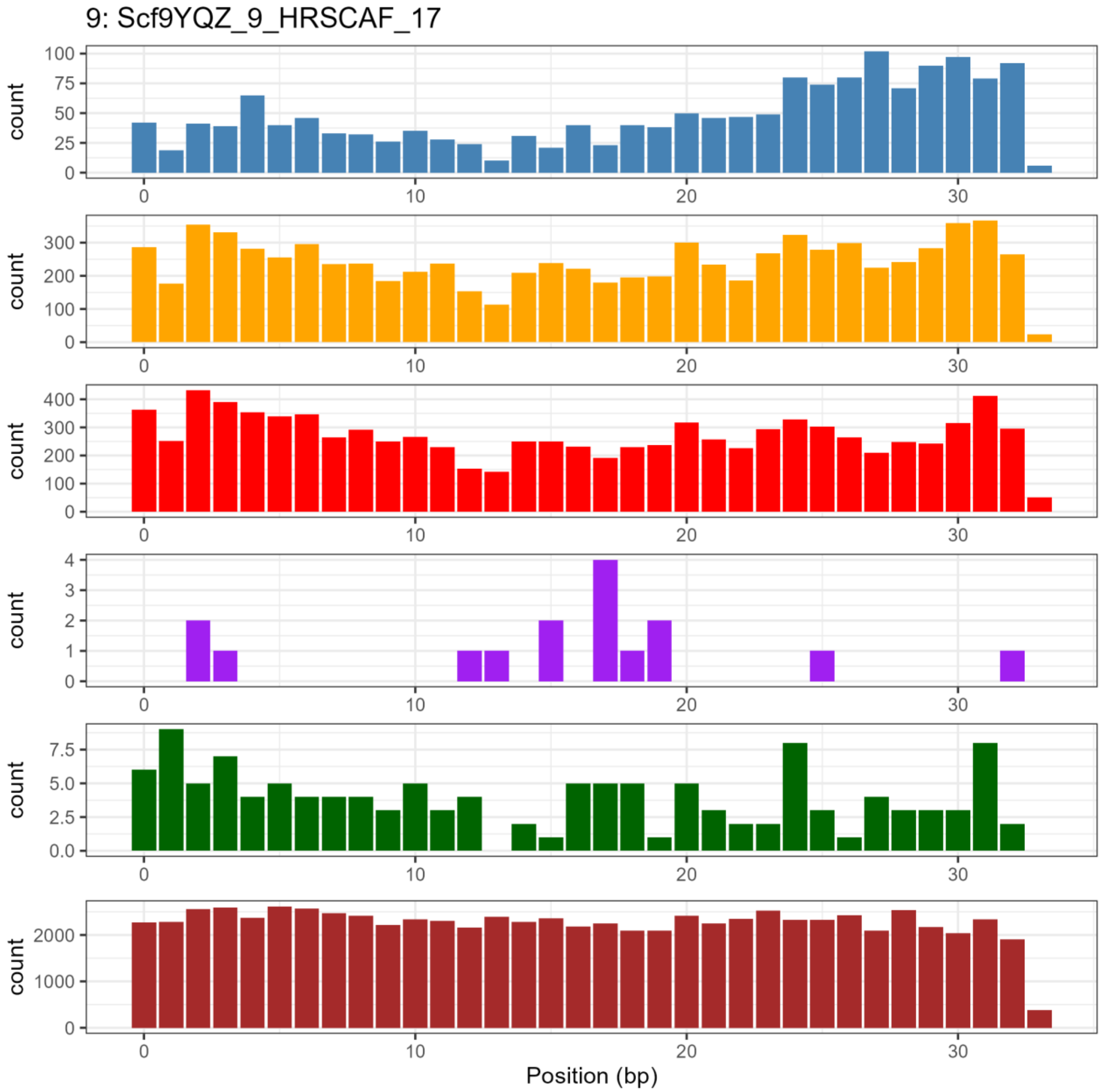

Supplementary Figure 38. Chromosome 9 feature plots. Features of the ninth-largest pseudochromosomal scaffold (Scf9YQZ_9_HRSCAF_17) of BATG-1.0. Each panel represents a feature plotted by density per 100kb (Y axis), along the chromosome (x axis). Panels are, from top to bottom, i) genes (blue), ii) insertions (orange), iii) deletions (red), iv) inversions (purple), v) tandem duplications (green) and vi) repeats (brown). Source data are provided as a Source Data file.

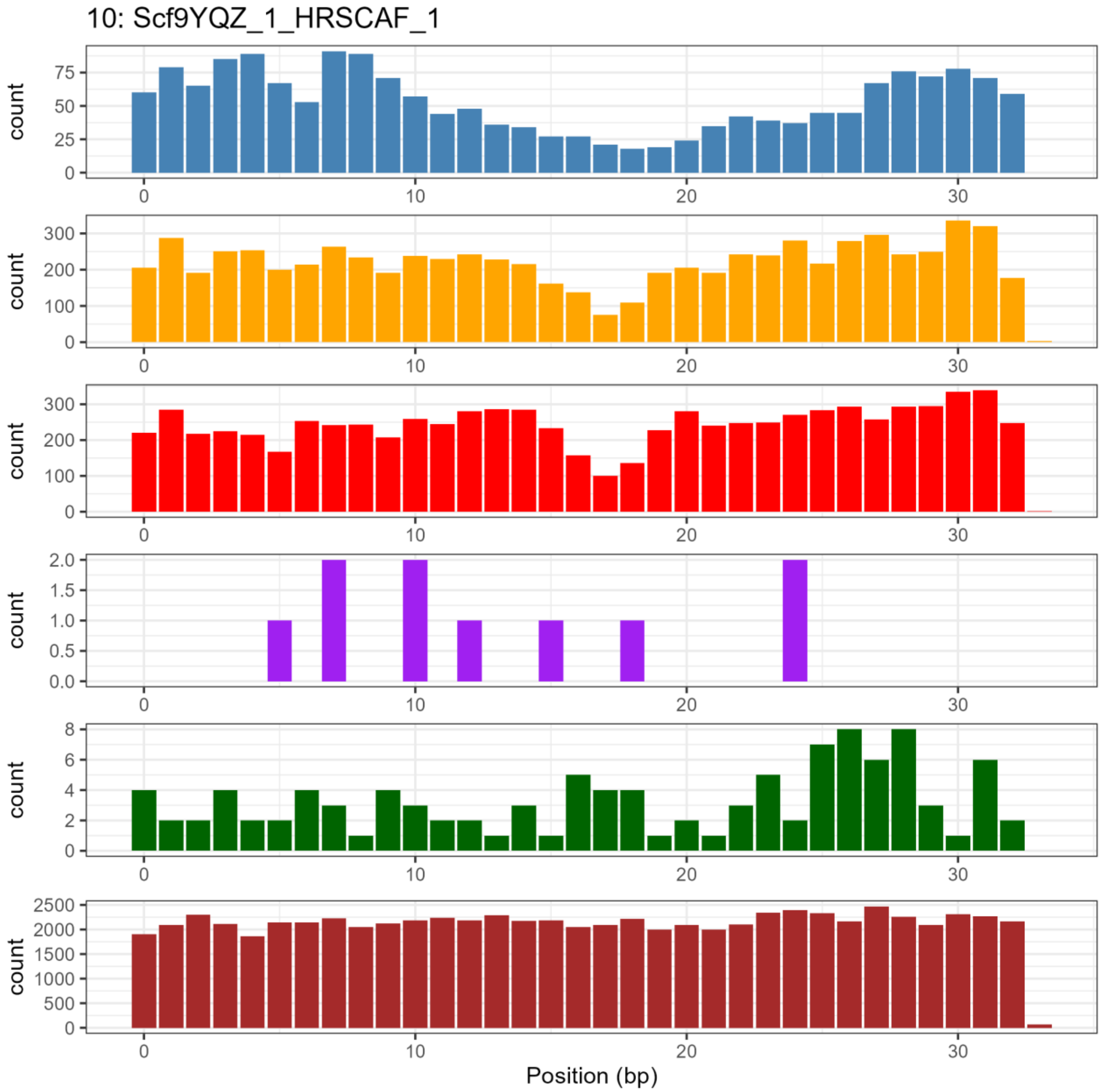

Supplementary Figure 39. Chromosome 10 feature plots. Features of the tenth-largest pseudochromosomal scaffold (Scf9YQZ_1_HRSCAF_1) of BATG-1.0. Each panel represents a feature plotted by density per 100kb (Y axis), along the chromosome (x axis). Panels are, from top to bottom, i) genes (blue), ii) insertions (orange), iii) deletions (red), iv) inversions (purple), v) tandem duplications (green) and vi) repeats (brown). Source data are provided as a Source Data file.

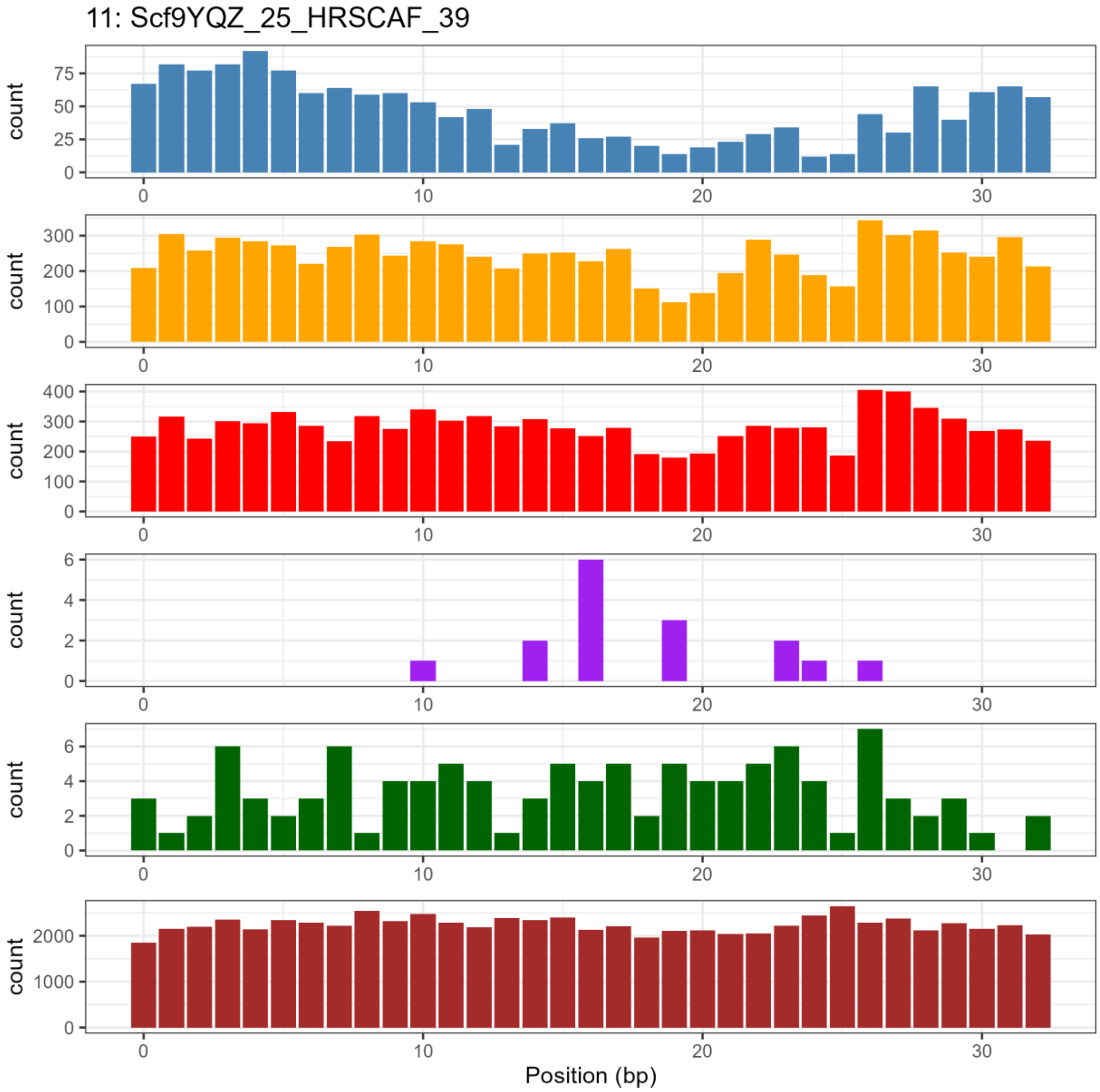

Supplementary Figure 40. Chromosome 11 feature plots. Features of the eleventh-largest pseudochromosomal scaffold (Scf9YQZ_25_HRSCAF_39) of BATG-1.0. Each panel represents a feature plotted by density per 100kb (Y axis), along the chromosome (x axis). Panels are, from top to bottom, i) genes (blue), ii) insertions (orange), iii) deletions (red), iv) inversions (purple), v) tandem duplications (green) and vi) repeats (brown). Source data are provided as a Source Data file.

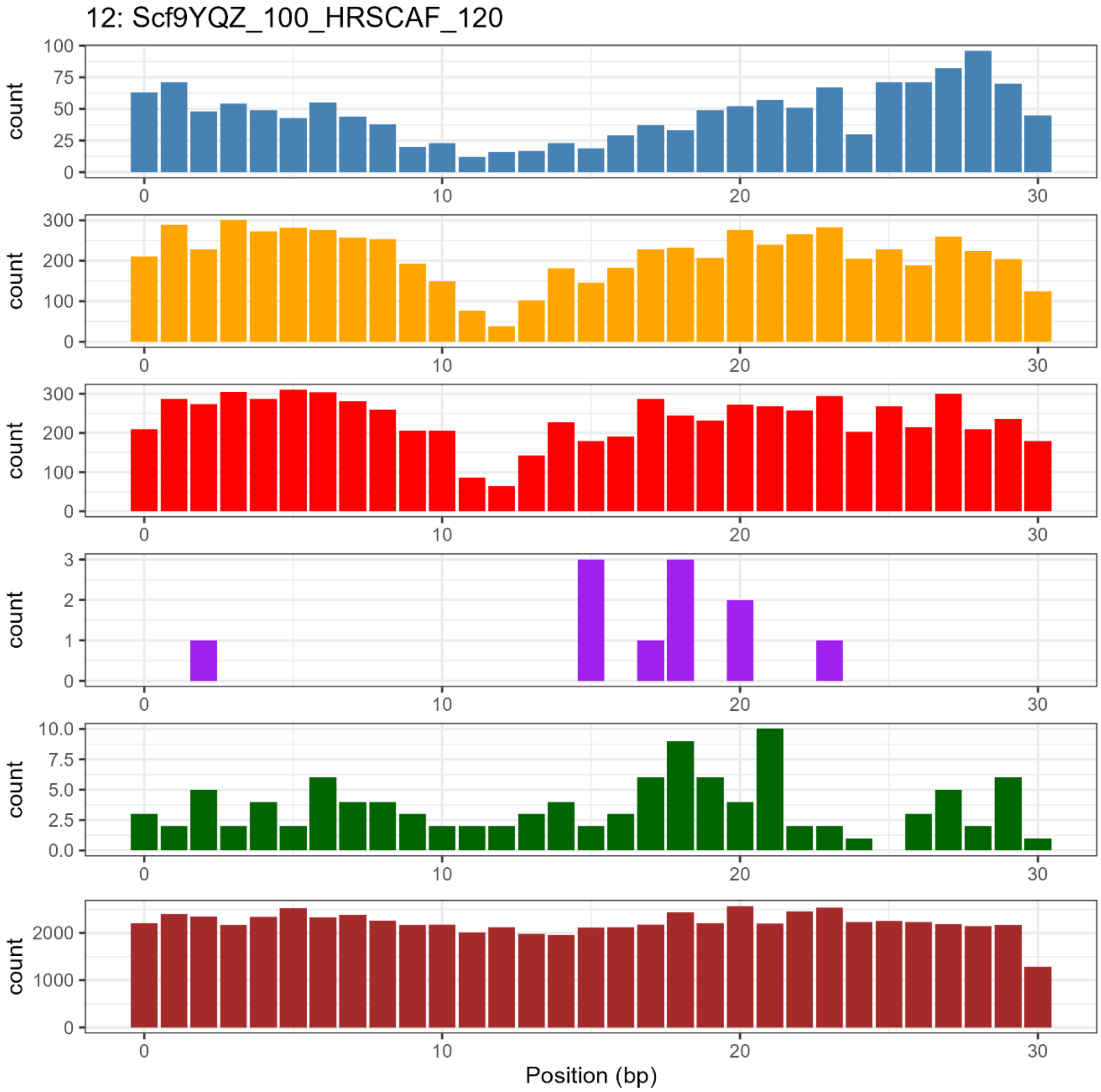

Supplementary Figure 41. Chromosome 12 feature plots. Features of the twelfth-largest pseudochromosomal scaffold (Scf9YQZ_100_HRSCAF_120) of BATG-1.0. Each panel represents a feature plotted by density per 100kb (Y axis), along the chromosome (x axis). Panels are, from top to bottom, i) genes (blue), ii) insertions (orange), iii) deletions (red), iv) inversions (purple), v) tandem duplications (green) and vi) repeats (brown). Source data are provided as a Source Data file.

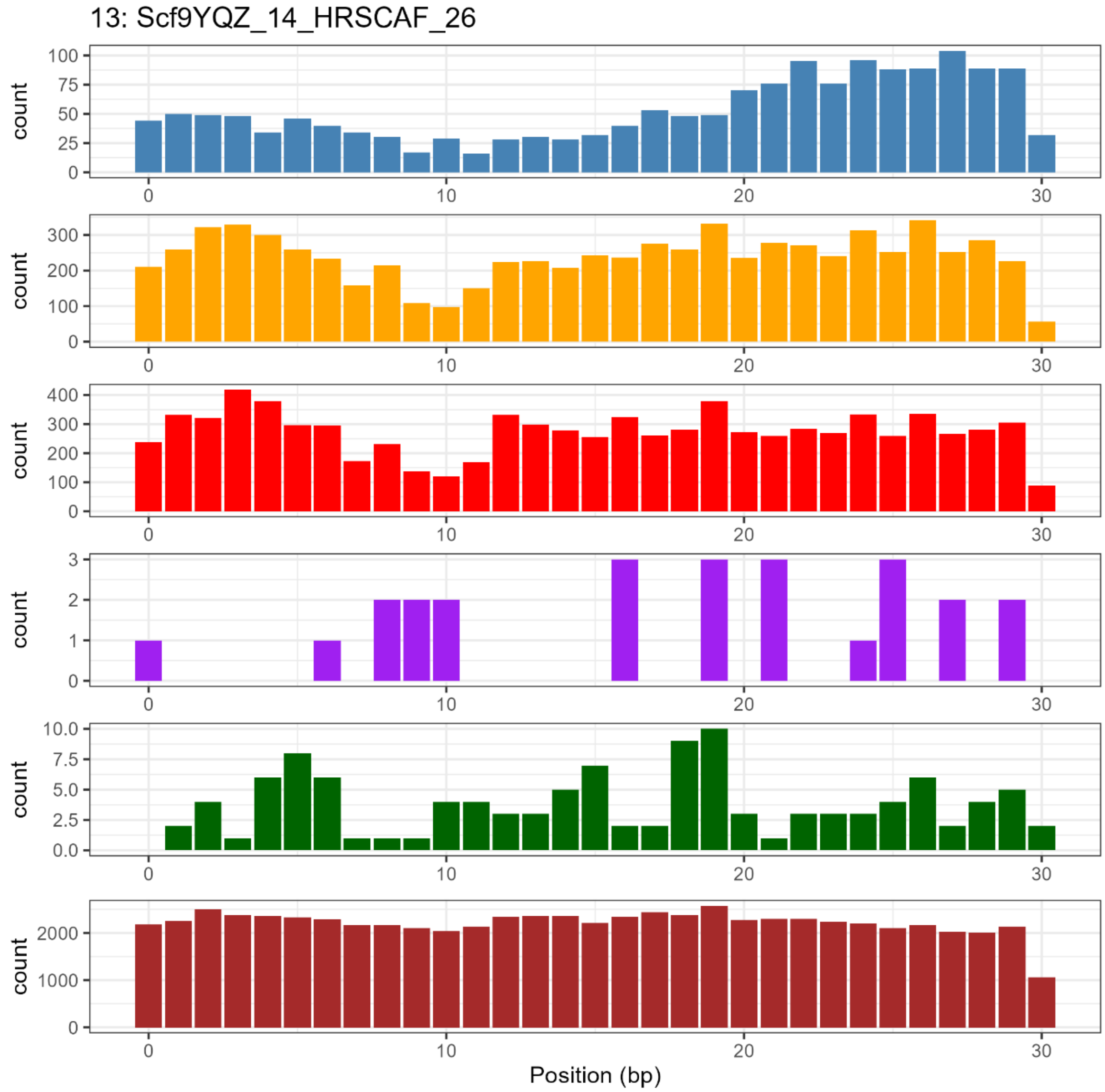

Supplementary Figure 42. Chromosome 13 feature plots. Features of the thirteenth-largest pseudochromosomal scaffold (Scf9YQZ_14_HRSCAF_26) of BATG-1.0. Each panel represents a feature plotted by density per 100kb (Y axis), along the chromosome (x axis). Panels are, from top to bottom, i) genes (blue), ii) insertions (orange), iii) deletions (red), iv) inversions (purple), v) tandem duplications (green) and vi) repeats (brown). Source data are provided as a Source Data file.

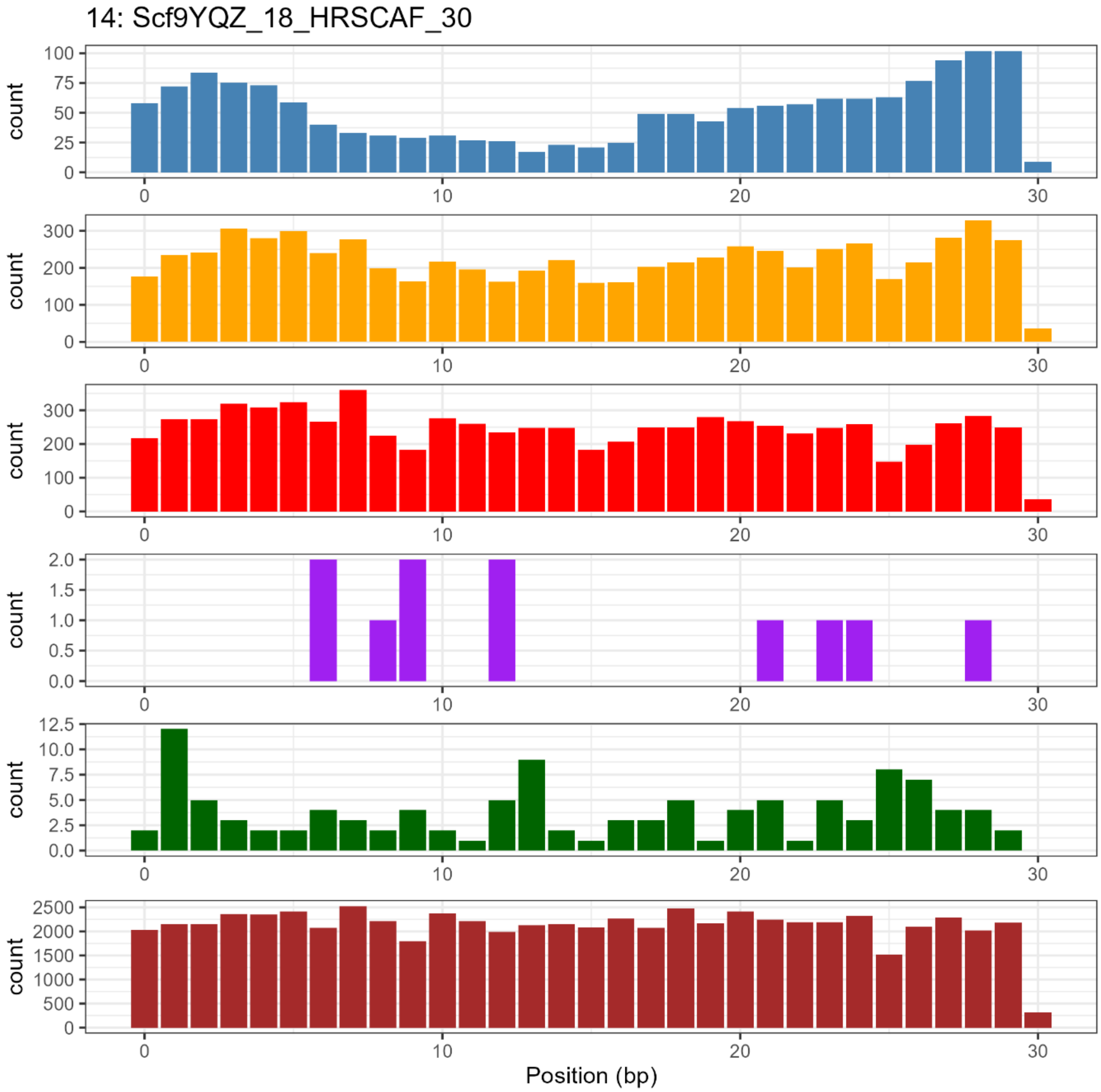

Supplementary Figure 43. Chromosome 14 feature plots. Features of the fourteenth-largest pseudochromosomal scaffold (Scf9YQZ_18_HRSCAF_30) of BATG-1.0. Each panel represents a feature plotted by density per 100kb (Y axis), along the chromosome (x axis). Panels are, from top to bottom, i) genes (blue), ii) insertions (orange), iii) deletions (red), iv) inversions (purple), v) tandem duplications (green) and vi) repeats (brown). Source data are provided as a Source Data file.

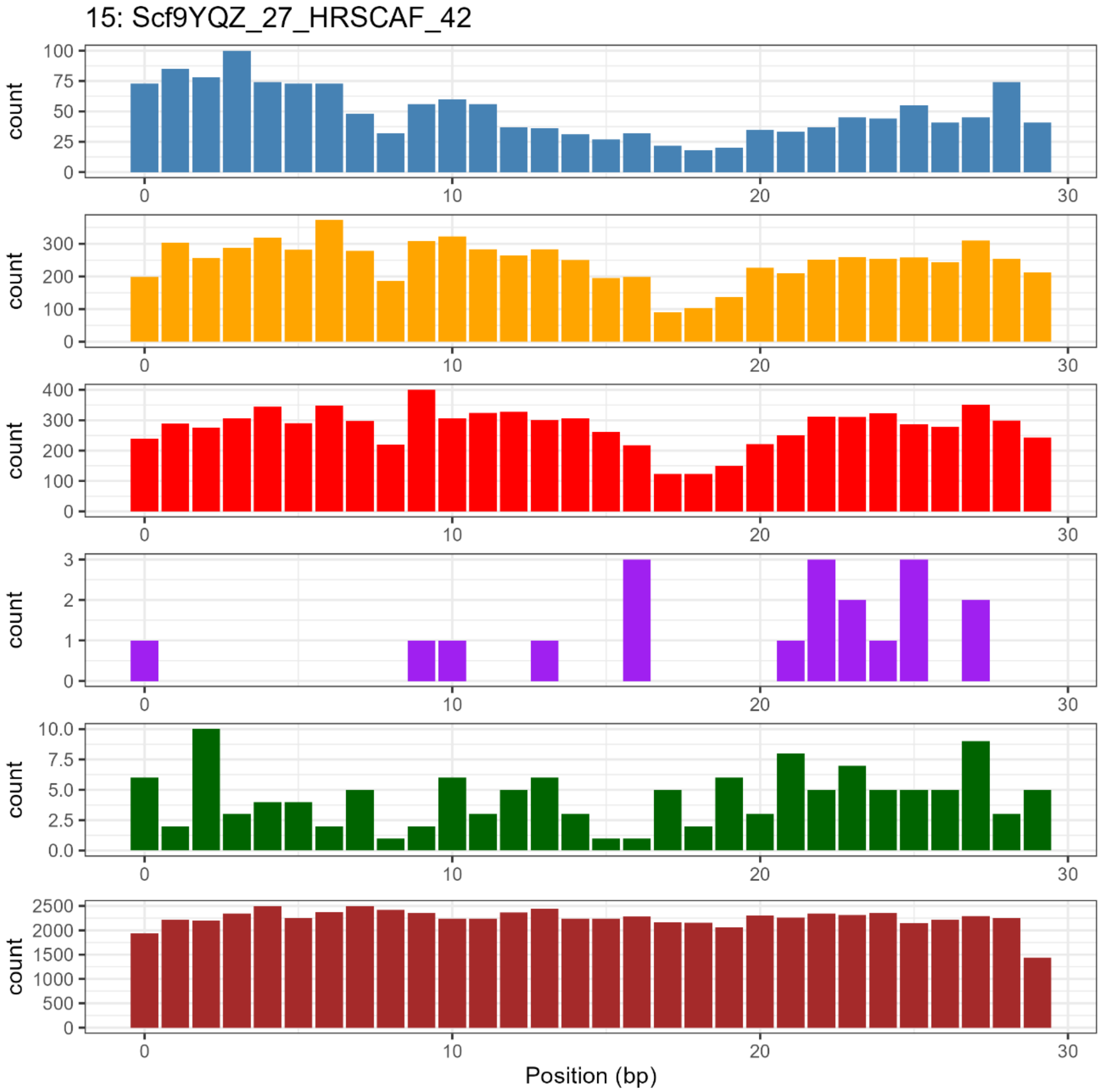

Supplementary Figure 44. Chromosome 15 feature plots. Features of the fifteenth-largest pseudochromosomal scaffold (Scf9YQZ_27_HRSCAF_42) of BATG-1.0. Each panel represents a feature plotted by density per 100kb (Y axis), along the chromosome (x axis). Panels are, from top to bottom, i) genes (blue), ii) insertions (orange), iii) deletions (red), iv) inversions (purple), v) tandem duplications (green) and vi) repeats (brown). Source data are provided as a Source Data file.

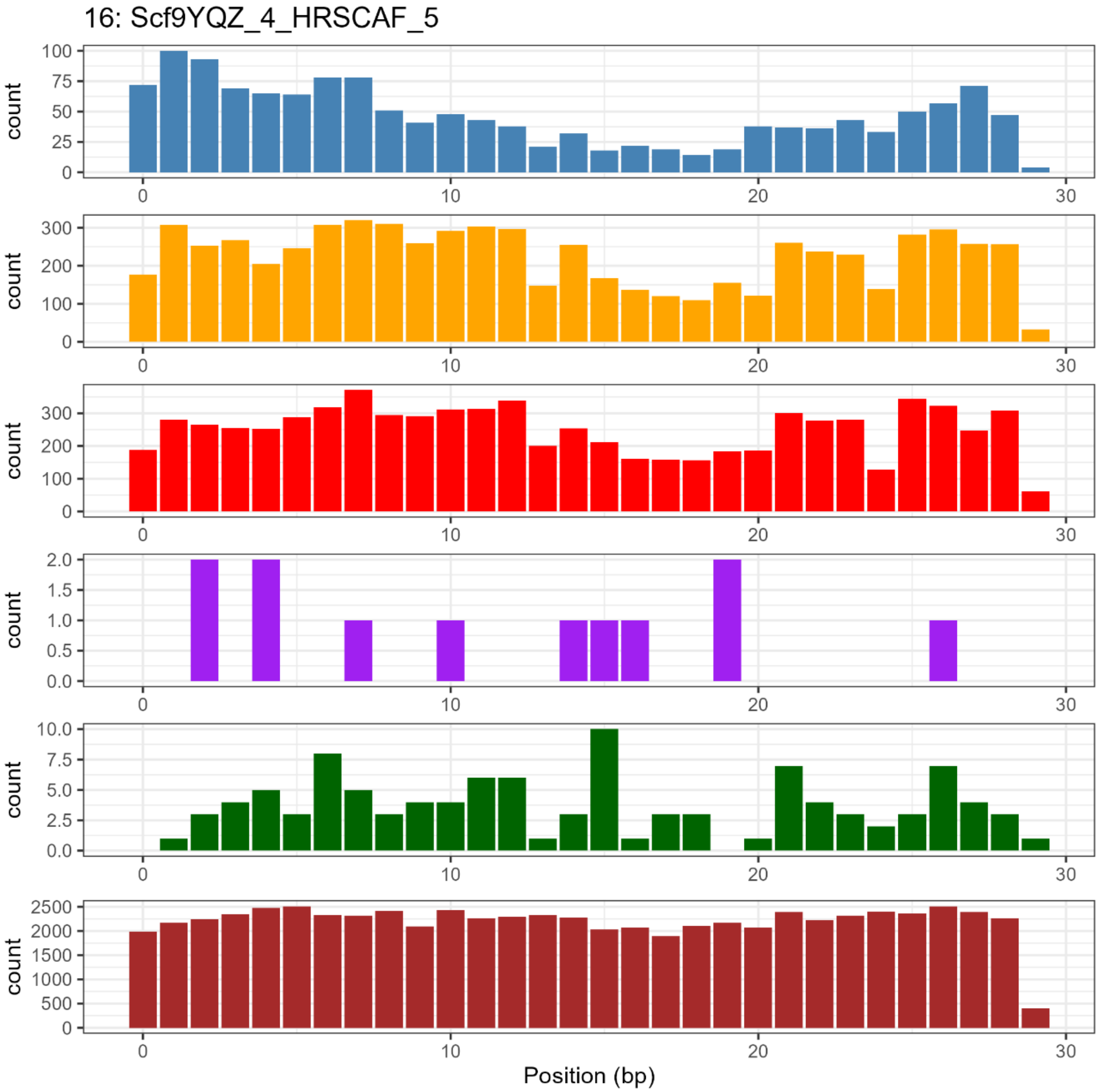

Supplementary Figure 45. Chromosome 16 feature plots. Features of the sixteenth-largest pseudochromosomal scaffold (Scf9YQZ_4_HRSCAF_5) of BATG-1.0. Each panel represents a feature plotted by density per 100kb (Y axis), along the chromosome (x axis). Panels are, from top to bottom, i) genes (blue), ii) insertions (orange), iii) deletions (red), iv) inversions (purple), v) tandem duplications (green) and vi) repeats (brown). Source data are provided as a Source Data file.

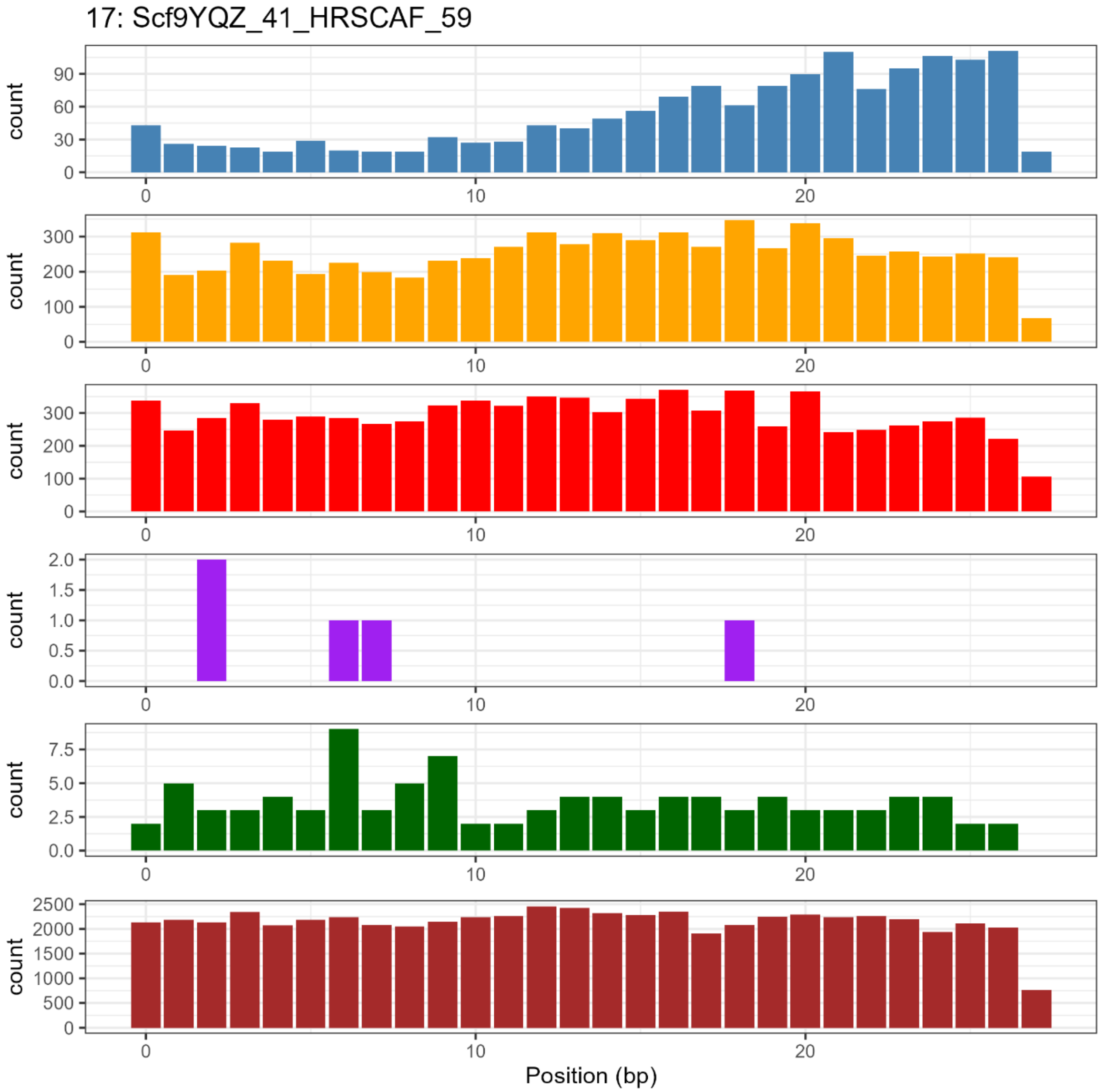

Supplementary Figure 46. Chromosome 17 feature plots. Features of the seventeenth-largest pseudochromosomal scaffold (Scf9YQZ_41_HRSCAF_59) of BATG-1.0. Each panel represents a feature plotted by density per 100kb (Y axis), along the chromosome (x axis). Panels are, from top to bottom, i) genes (blue), ii) insertions (orange), iii) deletions (red), iv) inversions (purple), v) tandem duplications (green) and vi) repeats (brown). Source data are provided as a Source Data file.

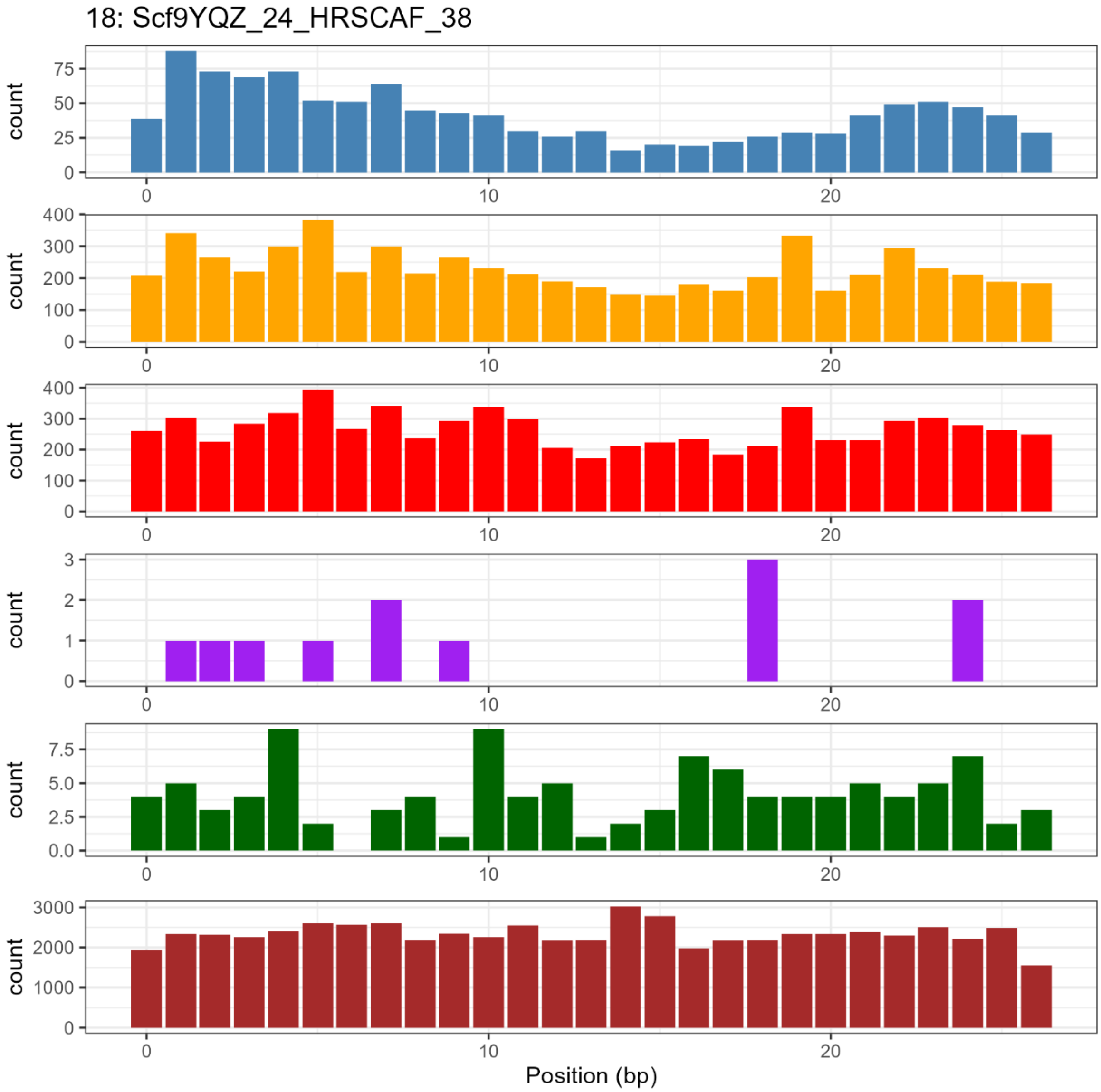

Supplementary Figure 47. Chromosome 18 feature plots. Features of the eighteenth-largest pseudochromosomal scaffold (Scf9YQZ_24_HRSCAF_38) of BATG-1.0. Each panel represents a feature plotted by density per 100kb (Y axis), along the chromosome (x axis). Panels are, from top to bottom, i) genes (blue), ii) insertions (orange), iii) deletions (red), iv) inversions (purple), v) tandem duplications (green) and vi) repeats (brown). Source data are provided as a Source Data file.

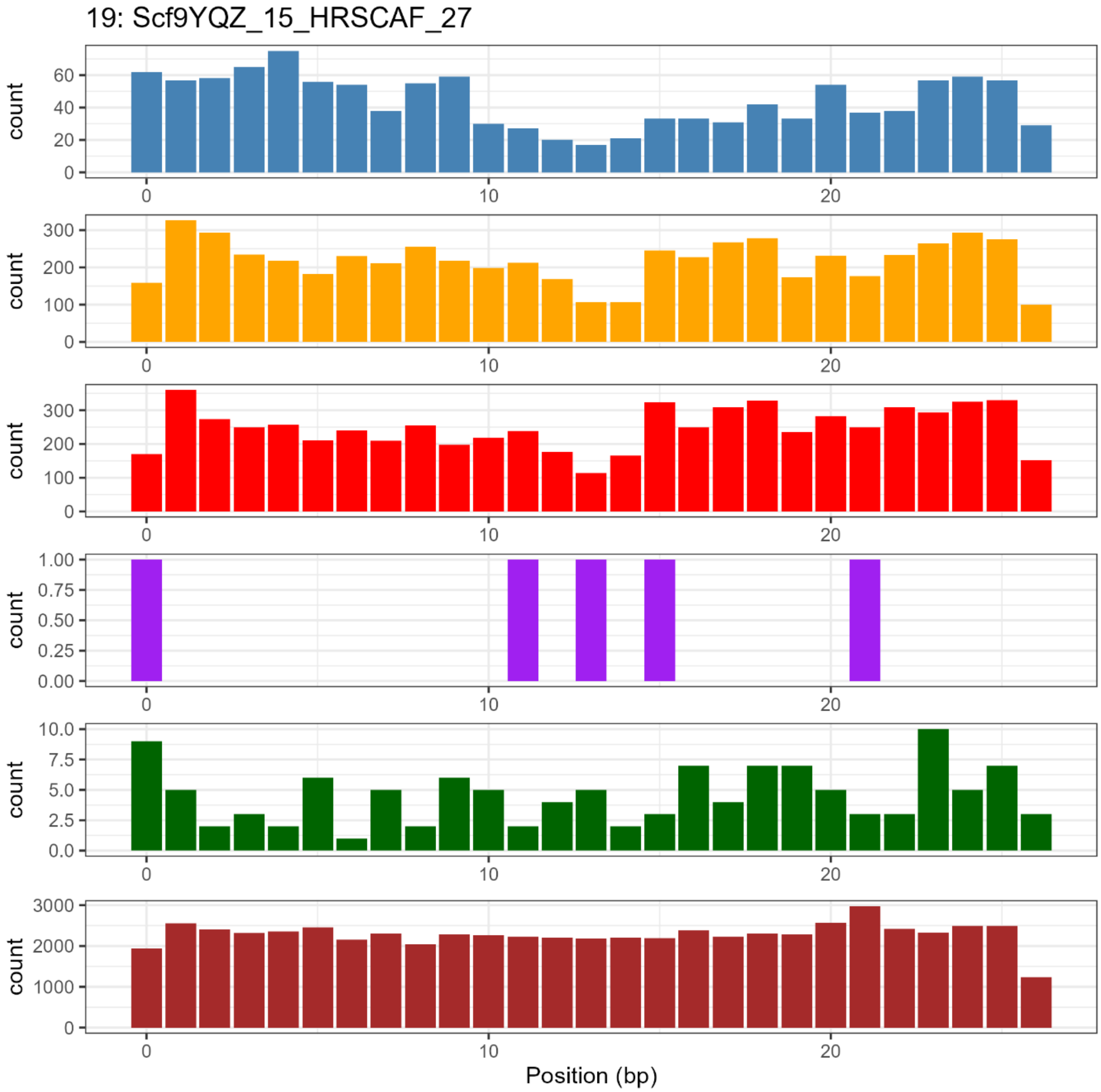

Supplementary Figure 48. Chromosome 19 feature plots. Features of the nineteenth-largest pseudochromosomal scaffold (Scf9YQZ_15_HRSCAF_27) of BATG-1.0. Each panel represents a feature plotted by density per 100kb (Y axis), along the chromosome (x axis). Panels are, from top to bottom, i) genes (blue), ii) insertions (orange), iii) deletions (red), iv) inversions (purple), v) tandem duplications (green) and vi) repeats (brown). Source data are provided as a Source Data file.

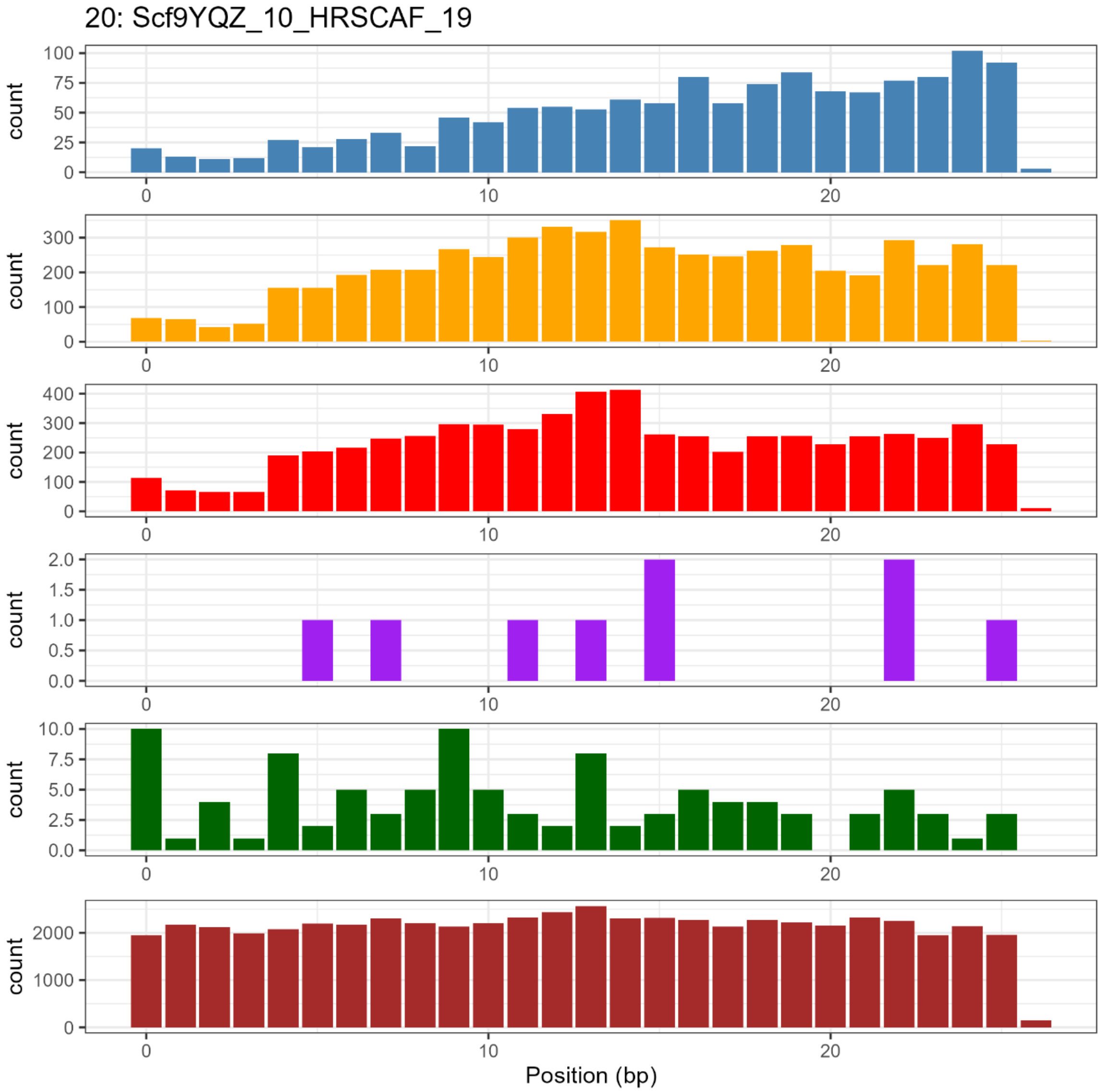

Supplementary Figure 49. Chromosome 20 feature plots. Features of the twentieth-largest pseudochromosomal scaffold (Scf9YQZ_10_HRSCAF_19) of BATG-1.0. Each panel represents a feature plotted by density per 100kb (Y axis), along the chromosome (x axis). Panels are, from top to bottom, i) genes (blue), ii) insertions (orange), iii) deletions (red), iv) inversions (purple), v) tandem duplications (green) and vi) repeats (brown). Source data are provided as a Source Data file.

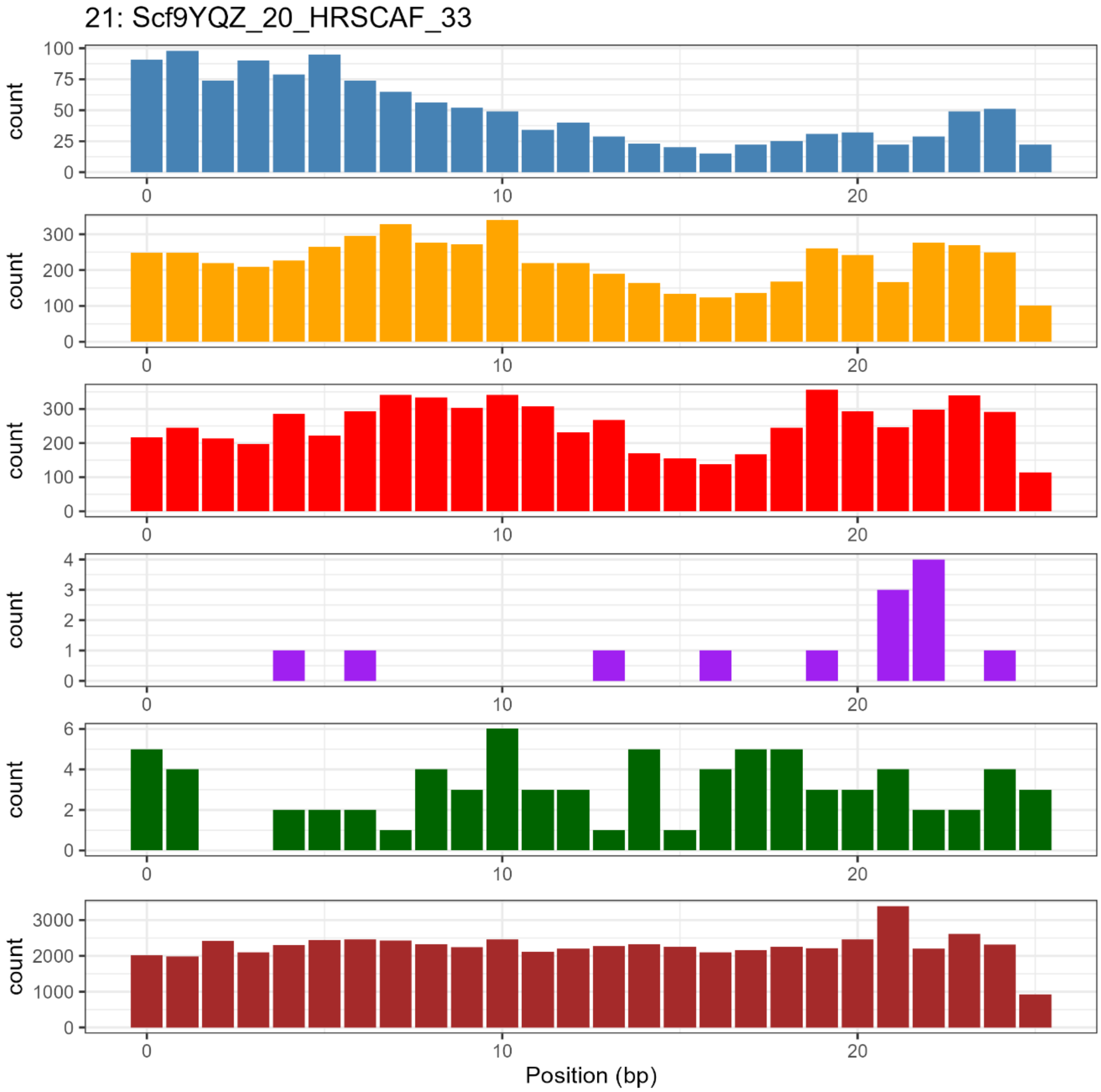

Supplementary Figure 50. Chromosome 21 feature plots. Features of the twenty-first-largest pseudochromosomal scaffold (Scf9YQZ_20_HRSCAF_33) of BATG-1.0. Each panel represents a feature plotted by density per 100kb (Y axis), along the chromosome (x axis). Panels are, from top to bottom, i) genes (blue), ii) insertions (orange), iii) deletions (red), iv) inversions (purple), v) tandem duplications (green) and vi) repeats (brown). Source data are provided as a Source Data file.

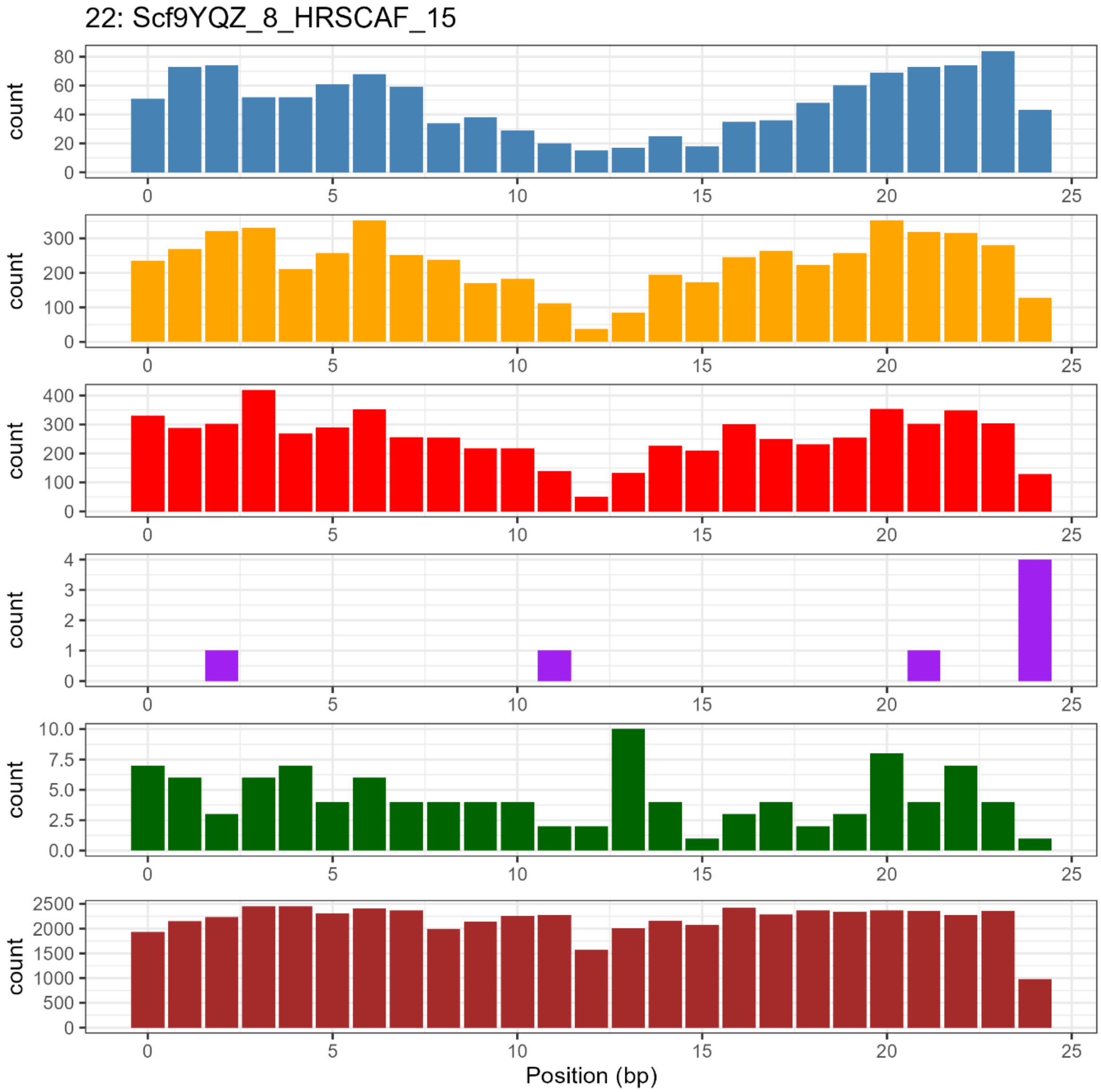

Supplementary Figure 51. Chromosome 22 feature plots. Features of the twenty-second-largest pseudochromosomal scaffold (Scf9YQZ_8_HRSCAF_15) of BATG-1.0. Each panel represents a feature plotted by density per 100kb (Y axis), along the chromosome (x axis). Panels are, from top to bottom, i) genes (blue), ii) insertions (orange), iii) deletions (red), iv) inversions (purple), v) tandem duplications (green) and vi) repeats (brown). Source data are provided as a Source Data file.

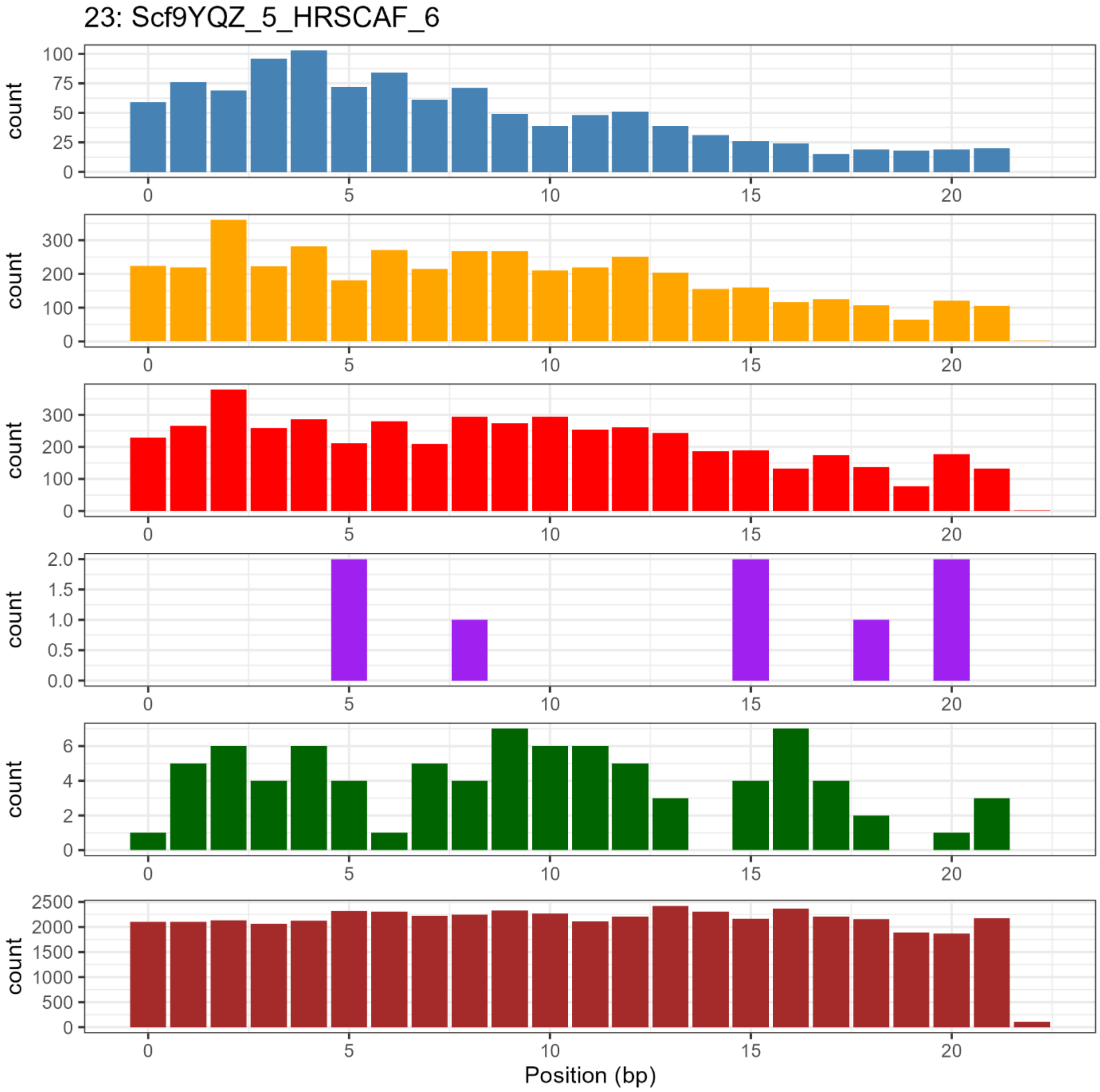

Supplementary Figure 52. Chromosome 23 feature plots. Features of the twenty-second-largest pseudochromosomal scaffold (Scf9YQZ_5_HRSCAF_6) of BATG-1.0. Each panel represents a feature plotted by density per 100kb (Y axis), along the chromosome (x axis). Panels are, from top to bottom, i) genes (blue), ii) insertions (orange), iii) deletions (red), iv) inversions (purple), v) tandem duplications (green) and vi) repeats (brown). Source data are provided as a Source Data file.

Supplementary Table 1. LTR Assembly Index per scaffold. For BATG-1.0 and haplotype 2, LTR Assembly Index (LAI) score per scaffold for top 23 scaffolds by size.

**Assembly**	**Chromosome number**	**Scaffold name**	**LAI**
BATG-1.0	1	Scf9YQZ_11_HRSCAF_22	21.24
BATG-1.0	2	Scf9YQZ_13_HRSCAF_25	19.21
BATG-1.0	3	Scf9YQZ_7_HRSCAF_29	21.05
BATG-1.0	4	Scf9YQZ_3_HRSCAF_24	20.22
BATG-1.0	5	Scf9YQZ_17_HRSCAF_229	22.67
BATG-1.0	6	Scf9YQZ_6_HRSCAF_27	20.19
BATG-1.0	7	Scf9YQZ_2_HRSCAF_23	21.06
BATG-1.0	8	Scf9YQZ_39_HRSCAF_257	19.56
BATG-1.0	9	Scf9YQZ_9_HRSCAF_217	19.7
BATG-1.0	10	Scf9YQZ_1_HRSCAF_21	20.21
BATG-1.0	11	Scf9YQZ_25_HRSCAF_239	18.63
BATG-1.0	12	Scf9YQZ_100_HRSCAF_2120	19.82
BATG-1.0	13	Scf9YQZ_14_HRSCAF_226	20.61
BATG-1.0	14	Scf9YQZ_18_HRSCAF_230	20.13
BATG-1.0	15	Scf9YQZ_27_HRSCAF_242	20.94
BATG-1.0	16	Scf9YQZ_4_HRSCAF_25	22.61
BATG-1.0	17	Scf9YQZ_41_HRSCAF_259	23.21
BATG-1.0	18	Scf9YQZ_24_HRSCAF_238	21.05
BATG-1.0	19	Scf9YQZ_15_HRSCAF_227	21.1
BATG-1.0	20	Scf9YQZ_10_HRSCAF_219	22.02
BATG-1.0	21	Scf9YQZ_20_HRSCAF_233	20.48
BATG-1.0	22	Scf9YQZ_8_HRSCAF_215	20.13
BATG-1.0	23	Scf9YQZ_5_HRSCAF_26	21.24
haplotype 2	1	ScfDVGaF_16_HRSCAF_28	21.25
haplotype 2	2	ScfDVGaF_12_HRSCAF_22	18.92
haplotype 2	3	ScfDVGaF_20_HRSCAF_34	20.45
haplotype 2	4	ScfDVGaF_144_HRSCAF_167	21.15
haplotype 2	5	ScfDVGaF_11_HRSCAF_18	22.49
haplotype 2	6	ScfDVGaF_7_HRSCAF_10	20.39
haplotype 2	7	ScfDVGaF_28_HRSCAF_47	19.72
haplotype 2	8	ScfDVGaF_21_HRSCAF_36	20.42
haplotype 2	9	ScfDVGaF_23_HRSCAF_38	20.45
haplotype 2	10	ScfDVGaF_17_HRSCAF_29	22.25
haplotype 2	11	ScfDVGaF_14_HRSCAF_25	18.85
haplotype 2	12	ScfDVGaF_8_HRSCAF_11	20.14
haplotype 2	13	ScfDVGaF_6_HRSCAF_9	19.42
haplotype 2	14	ScfDVGaF_39_HRSCAF_59	20.42
haplotype 2	15	ScfDVGaF_24_HRSCAF_39	18.25
haplotype 2	16	ScfDVGaF_2_HRSCAF_3	23.74
haplotype 2	17	ScfDVGaF_1_HRSCAF_2	21.38
haplotype 2	18	ScfDVGaF_4_HRSCAF_7	22.21
haplotype 2	19	ScfDVGaF_3_HRSCAF_5	21.75
haplotype 2	20	ScfDVGaF_9_HRSCAF_15	18.58
haplotype 2	21	ScfDVGaF_10_HRSCAF_16	22
haplotype 2	22	ScfDVGaF_33_HRSCAF_53	18.23
haplotype 2	23	ScfDVGaF_5_HRSCAF_8	21.74

Supplementary Table 2. Single nucleotide polymorphism calling statistics using linear reference genome vs. pangenome

	**Linear reference**	**Pangenome**	**Linear reference only**	**Pangenome only**
Total SNPs called	18,955,565	18,540,348	1,057,326	642,020
Transition/Transversion ratio	2.2	2.18	2.5	2.13
Mean proportion missing	1.32%	1.26%	3.30%	3.90%
Mean AF	0.269	0.273	0.187	0.231
% sites with QUAL < 284.5 (maximum)	6.44%	6.15%	8.40%	6.52%
% sites with p (HWE) < 1	57.30%	57.80%	44.10%	54.30%

Table outlining single nucleotide polymorphism (SNP) calling statistics for reads from 42 short-read sequenced samples, called i) using a the BATG-1.0 linear reference genome, ii) using the pangenome, iii) called only using the BATG-1.0 linear reference genome, and iv) called only using the pangenome. Statistics include a) the total SNPs called, b) the transition/transversion ratio, c) the mean proportion missing, d) the mean allele frequency of SNPs called, e) the percentage of sites with the SNP quality score being less than the maximum value (284.5), and e) the percentage of sites with a *p*-value from the bcftools Hardy Weinberg Equilibrium test (two-sided chi-squared test) < 1.

Supplementary references

1. Stocks, J. J. et al. Genomic basis of European ash tree resistance to ash dieback fungus. *Nat. Ecol. Evol*. **3**, 1686–1696 (2019).
2. Li, H. & Durbin, R. Fast and accurate short read alignment with Burrows-Wheeler transform. *Bioinformatics* **25**, 1754–1760 (2009).
3. Metheringham, C. L. et al. Rapid polygenic adaptation in a wild population of ash trees under a novel fungal epidemic. *Science* **388**, 1422–1425 (2025).
4. Wiberg, R. A. W., Gaggiotti, O. E., Morrissey, M. B. & Ritchie, M. G. Identifying consistent allele frequency differences in studies of stratified populations. *Methods Ecol. Evol*. **8**, 1899–1909 (2017).
5. Li, H. et al. The Sequence Alignment/Map format and SAMtools. *Bioinformatics* **25**, 2078–2079 (2009).
6. Talenti, A. & Prendergast, J. nf-LO: A Scalable, Containerized Workflow for Genome-to-Genome Lift Over. *Genome Biol. Evol*. **13**, evab183 (2021).
7. Franco Ortega, S. et al. Fraxinus excelsior updated long-read genome reveals the importance of MADS-box genes in tolerance mechanisms against ash dieback. *G3* **15**, 2024.12.20.629733 (2025).
8. Emms, D. M. & Kelly, S. OrthoFinder: phylogenetic orthology inference for comparative genomics. *Genome Biol.* **20**, 238 (2019).
9. Sollars, E. S. A. et al. Genome sequence and genetic diversity of European ash trees. *Nature* **541**, 212–216 (2017).
10. Ranallo-Benavidez, T. R., Jaron, K. S. & Schatz, M. C. GenomeScope 2.0 and Smudgeplot for reference-free profiling of polyploid genomes. *Nat. Commun*. **11**, 1432 (2020).
11. Smolka, M. et al. Detection of mosaic and population-level structural variants with Sniffles2. *Nat. Biotechnol*. **42**, 1571–1580 (2024).
12. Jiang, T. et al. Long-read-based human genomic structural variation detection with cuteSV. *Genome Biol.* **21**, 189 (2020).
13. Heller, D. & Vingron, M. SVIM-asm: structural variant detection from haploid and diploid genome assemblies. *Bioinformatics* **36**, 5519–5521 (2021).
14. Chang, C. C. et al. Second-generation PLINK: rising to the challenge of larger and richer datasets. *Gigascience* **4**, 7 (2015).
15. Gogarten, S. M. et al. GWASTools: an R/Bioconductor package for quality control and analysis of genome-wide association studies. *Bioinformatics* **28**, 3329–3331 (2012).
16. Chano, V. et al. Transcriptional time-course analysis during ash dieback infection revealed different responses in tolerant and susceptible Fraxinus excelsior genotypes. *BMC Plant Biol.* **25**, 107 (2025).
17. Ferrari, R. C. et al. Transcriptional profiling of Fraxinus excelsior leaves during the early infection phase of ash dieback. *J. Plant Dis. Prot*. **132**, 48 (2025).
18. Sahraei, S. E., Cleary, M., Stenlid, J., Brandström Durling, M. & Elfstrand, M. Transcriptional responses in developing lesions of European common ash (*Fraxinus excelsior*) reveal genes responding to infection by Hymenoscyphus fraxineus. *BMC Plant Biol*. **20**, 455 (2020).
19. Alexa A, R. J. topGO: Enrichment Analysis for Gene Ontology. http://bioconductor.org/packages/topGO/ (2024).
