# Supplementary material for: European ash pangenome reveals widespread structural variation and genetic basis of low ash dieback susceptibility
Source: Nat Commun. 2026 Sep 17;17:9693. doi: 10.1038/s41467-026-77194-9 (PMC13586383; doi:10.1038/s41467-026-77194-9)
Supplement: Supplementary file 3 [file 41467_2026_77194_MOESM2_ESM.pdf]

nature portfolio

Peer Review File

European ash pangenome reveals widespread structural variation and genetic basis of low ash dieback susceptibility

Corresponding Author: Dr Daniel Wood

This file contains all reviewer reports in order by version, followed by all author rebuttals in order by version.

Attachments originally included by the reviewers as part of their assessment can be found at the end of this file.

Version 0:

Reviewer comments:

Reviewer #1

(Remarks to the Author)
Wood et al. assemble a phased, chromosome-scale Fraxinus excelsior reference (BATG-1.0), build a 50-sample graph pangenome, and re-analyse pool-seq data for loci underlying low susceptibility to ash dieback (ADB). The resource is valuable and timely; the assembly is highly contiguous with telomeric repeats anchoring most major scaffolds, and the pangenome captures extensive SV diversity (≈363k SVs; ≈174 Mb novel sequence). However, several conclusions—especially the size/composition of the "dispensable gene" set and a reported chr15 candidate insertion—rely on assumptions or incomplete validation and would benefit from additional analyses and clarifications.

Major comments
1) Assembly quality and repeat completeness need repeat-aware validation
The assembly is large and contiguous (hap1: 793 Mb, N50 = 32.9 Mb) with many scaffolds showing terminal telomeric motifs, but the size is ~84–90 Mb below the prior 1C estimate (~877 Mb). Repeat content is reported at ~66% (mostly LTRs). Please substantiate repeat completeness:
• Compute LAI (with LTR_retriever) and report intact LTR counts/ages per scaffold; compare with Fraxinus/Oleaceae references.
• Provide a k-mer–based genome size estimate (e.g., GenomeScope from Illumina/HiFi k-mers) to triangulate with flow-cytometry.
• Discuss and, if possible, demonstrate repeat collapse (e.g., coverage profiles showing modal ~2× in collapsed regions) as a likely source of the ~100 Mb shortfall; also note inter-individual genome-size variation.
2) SV calling/merging: precision vs. recall, class coverage, and merging parameters
Your internal benchmark (haplotype-vs-haplotype "truth") yields recall 84.4% and precision 72.2% for the chosen union strategy; you then restrict to SVs seen in ≥3 individuals. This helps, but remains an indirect false positive control. Please:
• Report class-specific precision/recall (INS/DEL/INV/TANDUP) against the haplotype truth set; add a second precision check by spot-validating 30–50 SVs (local re-assembly or PCR) in two non-reference samples.
• Justify SURVIVOR's 200 bp merge distance and show a sensitivity sweep (100/150/300 bp) to avoid over-merging distinct TE-proximal events.
• State up front that BND/translocations were excluded (reasonable for reliability), and—if feasible—add a conservative BND subset with assembly support.
• Within individuals you generate a non-overlapping SV set by prioritizing insertions and longer events; please justify biologically and quantify how often shorter, potentially compound events were dropped.
3) Pangenome annotation & the "dispensable gene" set—assumptions that limit inference
You annotate each transformed genome using RNA-seq from the reference individual's leaf/cambium and require a consistent SV overlap (called "F1" ≥0.8) to retain genes as dispensable. This is an elegant way to curb annotation artifacts, but it risks false negatives for genes expressed in other tissues/states and for PAV arising via dispersed duplications or non-local regulatory changes. Please:
• Incorporate additional F. excelsior RNA-seq (tissues/seasons/stress) if available, or explicitly acknowledge the limitation.
• Add a read-depth DNA presence/absence check for gene bodies (Illumina/ONT) to rescue genes with weak RNA evidence.
• Consider liftover from updated F. excelsior annotations to complement BRAKER3.

• Rename the custom “F1” (it is not the standard F1) to SV–gene association score, justify the 0.8 cutoff, and provide a threshold sensitivity analysis (e.g., 0.7/0.9) for dispensable-gene counts and GO enrichments.
• Temper functional language: “133 with GO terms associated with low susceptibility” are annotations, not functional evidence; report enrichment with appropriate backgrounds and FDR/q-values, or frame as hypotheses.
Note on consistency: The Results state 7.8% dispensable (3,065/39,420), but the Discussion states 6.6%. Please reconcile.
4) Chromosome-level context of SVs is under-presented
You note that 99.9% of SVs map to the 23 pseudochromosomes and 69% overlap repeats, but a genome-wide view is missing. Please add per-chromosome SV density (e.g., 100 kb windows; INS vs DEL), alongside repeat and gene density, and test enrichment in peri/centromeric regions. This will also help interpret the flat INV/TANDUP trends in Fig. 2B/C (likely low counts + class-specific sensitivity + the ≥3 filter).
5) Graph mapping: quantify biological impact, not just mapping metrics
Mapping to the pangenome reduces soft-clipping by ~33% and modestly improves MQ/AS (~1–1.5%). Please show whether these changes alter callsets (SNP/SV): concordance vs linear mapping, Ti/Tv, HWE, etc., to place mapping improvements in biological context.
6) Pool-seq GWAS: clarify design, apply multiple-testing control, temper claims, and strengthen chr15
Pool-seq SV allele frequencies are noisy (your in-silico validation shows discrepancies), and SV CMH hits are enriched for such discrepancies. CMH SNP hits are largely provenance-specific; qGLM yields none. This fits a highly polygenic trait with strong heterogeneity. Please:
• Provide plain-language pool-seq and phenotype summaries (definition of “healthy/unhealthy”; compact recap of Stocks et al. design) in Methods; and apply genome-wide multiple-testing control (FDR/Bonferroni) with QQ/λGC for the CMH SNP analysis (currently a fixed $p < 1\times10^{-13}$ threshold).
• Use a meta-analysis across provenances (fixed/random effects; Cochran’s Q, I^2) to formalize heterogeneity; report the fraction of hits with consistent direction.
• Temper language in Abstract/Results to emphasize provenance-specific architectures rather than “strong associations.”
• chr15 signal: The tight SNP cluster + depth spikes are intriguing, but no insertion is in your SV catalog. Please rephrase as a candidate insertion-like signal and add targeted validation: (i) local short-read assemblies of depth-spike pools to recover insert/breakpoints; (ii) targeted ONT or amplicon validation in individuals; (iii) screen for TE hallmarks and potential regulatory motifs; (iv) reconcile the NSZ-107 replicate with higher depth in the unhealthy pool.
7) BUSCO: reconcile genome vs. protein assessments
Assembly BUSCO completeness is ≈98.2–98.3% (genome mode), whereas the protein set shows 94.4% completeness. Please present a small table clarifying genome vs protein BUSCO and discuss why the protein set is lower (conservative consensus across 20 BRAKER runs; tissue bias).

Figure-specific / presentation comments
1. Figure 2 (SV properties).
○ Panel A: Clarify that colors represent different random merge-order replicates (not SV classes).
○ Panels B/C: Add a sentence explaining the flat INV/TANDUP curves at higher support cutoffs (rarity, class-specific sensitivity, ≥3 filter) and consider adding per-class discovery curves vs. individuals to show whether INS/DEL discovery has plateaued.
2. Figure 3 (dispensable logic).
○ Rename “F1” in the panel/legend to SV–gene association score (threshold 0.8); clarify in the caption that categories 3 and 5 (no SV overlap) are treated as invariant or discarded, respectively.
3. Extended Data Figure 1 (SV benchmarking).
○ The methods are independent; lines connecting points imply a trajectory. Please plot points without connecting lines or facet by method.
4. Genome-wide SV map.
○ Add a circos/per-chromosome SV density (INS vs DEL) with repeat/gene density to support textual statements and aid biological interpretation.
5. Extended Data numbering.
○ In Fig. 5D you direct readers to ED Fig. 10 for depth plots, but ED Fig. 10 is the pangenome annotation diagram. Please fix the cross-reference.
Minor comments (clarity, methods gaps, consistency)
• Define pool-seq, CMH, qGLM, and Fst sliding window on first use; in Fig. 5 caption, note why CMH and Fst are complementary.
• SNP calling (ONT): State per-sample coverage; justify DP filters (DP<8 or >60 set to missing) used for PCA.
• Graph construction: Report basic graph stats (nodes/edges, added sequence per chromosome) and deposit vg indexes alongside VCF/FASTA.
• Nomenclature/typos: Standardize “pseudochromosomal” (a few instances read “pseudochromosmal”) and use consistent labels (“BATG-1.0” for hap1; “haplotype 2” for hap2).
• Long-read trimming: You apply Trimmomatic to ONT reads before NanoFilt; this is unusual—please justify or simplify.
• SV thresholds: Explicitly state treatment of partially overlapping SVs within individuals vs across individuals; justify n ≥ 3 and 200 bp merge distance, and add sensitivity.
• Data/code availability: Replace NCBI accession “XXXXX” with the actual accession; ensure code repo contains pinned environments/containers.

Reviewer #2

(Remarks to the Author)
The study by Wood and coauthors reports the assembly of a pangenome for common ash, with the aim of enhancing the

genomic resources available for studying this species, particularly in the context of ash dieback (ADB). This is a highly relevant and timely area of research. The work represents an impressive bioinformatic effort, and the analytical pipelines appear to be both thorough and robust. However, we see four drawbacks that, in our opinion, substantially limit the validity of the results:

1) The lack of RNA-seq data for pangenome annotation for any individual other than the linear reference individual and its mother. This data seems very sensitive for a reliable assessment of gene models for individuals across the range and especially dispensable genes not found in the reference individual. Given the strong focus of the work on identifying reliable dispensable genes (see statement already in the abstract at lines 28-30, we “demonstrated that failing to explicitly link SVs…. Can lead to substantial overestimation of dispensable genes”), this lack limits its results and conclusions. Note that gene prediction artefacts could lead to a lack of overlap between dispensable genes and SVs, as suggested also at lines 370-372. Since other RNAseq data is publicly available from other studies it could be a valid option to use it to improve or confirm annotations (even if the individual trees don´t match with the assemblies)?

2) We don´t think that the Fst outlier scan approach is the correct analytical approach for ADB susceptibility as not enough time passed since the advent of ADB to justify this approach and we expect limited power, especially given that no study until now suggested the existence of very large-effect loci controlling the response to ADB.

3) It is not clear to us how the same poolseq data from Stocks et al 2019 (SNPs section) which delivered consistent GWAS top candidates using the older reference, does not show consistent results in association outliers across provenances when using the pangenome. Overall, the (inconsistent) results of GWAS using the pangenome is not discussed enough in comparison to the results from Stocks et al 2019. Maybe for this reason, we are not convinced that using the pangenome instead of the linear reference can be defined a substantial improvement.

4) The authors explain the lack of genomic regions consistently associated with ADB susceptibility across provenances and pools in the GWAS analyses as a consequence of different genetic backgrounds harbouring different sets of loci involved in susceptibility (lines 34-35, ….). Even if this may be a valid explanation in line with results from other studies (Chano et al 2025, and a preprint from Szukala et al 2024), an additional potential explanation could lie in the way susceptibility was assessed based on the crown damage using a binary assessment (healthy vs susceptible), which is probably affected by a series of variables and may not capture well the quantitative response of the ash trees to the infection. We think that this possibility should be at least discussed also in light of the lack of overlap of top associated loci from GWAS analyses across Europe (Stock et al. 2019; Doonan et al. 2025), which used a similar assessment approach.

Minor comments:
Introduction:

1. Lines 48 “the majority of genes” and line 65 “a small fraction of trees” – please be more specific about the amount.

Results:

1. Line 98: the smaller size of this assembly compared to previously reported values (almost a 10% difference to 1C = 877 Mb; and a smaller difference to FRAX_001_PL total ungapped length ca 806.3 Mb) even if not unusual probably indicates that the assembly still collapses a good portion of repetitive regions?
2. Lines 130-134; the fact that short reads RNA-seq leads to the discovery of more genes and in general the differences in annotation between short and long reads could be discussed more. Could they be explained by the higher depth of short-reads, which allows for better discovery of genes with low expression? Do short non-coding RNAs detected by short reads but not long reads? Is the BREAKER pipeline more sensitive to short reads – are long reads collapsed into fewer, longer models? I think these questions highlight the importance of the RNAseq source used for the annotation (see also my first comment).
3. Lines 169-173: lack of a plateau in discovered SVs by addition of new individuals. Can you exclude that a technical bias, instead of a biological explanation, lies behind this observation? Can it be that each individual assembly may come with assembly-specific errors (collapsed repeats, misjoins,…) that appear as new SVs?

Discussion

1. Lines 329-334 - Improvements of the chromosome-level assembly are reported and highlighted mainly in relation to the BATG-5.0 assembly, but I believe it would be more meaningful to report improvements in relation to pseudochromosomal-level assemblies such as FRAX_001_PL.

M & M

1. Lines 466-467: fits better in Results section.
2. Lines 726-727: 2.2X coverage per individual is very low for GWAS even within the context of poolseq, with much lower resolution and more confounding than individual-level GWAS. Even if this is the same data published in Stocks et al 2019, maybe this low level of resolution should be considered when discussing the GWAS results.
3. Line 521: as far as I can see in S Table 3 provenances are from UK, Ireland, Lithuania, France, Denmark, Italy, Czech Republic, Poland, Belgium, Germany, Russia; please report in the text that variation from SE Europe and SW Europe is fully missing.
4. Line 568 Since long-reads were used for both genome assembly and RNAseq, pleace specify in the title which long reads.

We hope that our remarks help you to improve the manuscript!

Heino Konrad & Aglaia Szukala
Austrian Research Centre for Forests (BFW), heino.konrad@bfw.gv.at, aglaia.szukala@bfw.gv.at

Aglaia Szukala co-reviewed this manuscript. This is part of the Nature Communications initiative to facilitate training in peer review and to provide appropriate recognition for Early Career Researchers who co-review manuscripts.

Reviewer #3

(Remarks to the Author)
I co-reviewed this manuscript with one of the reviewers who provided the listed reports. This is part of the Nature Communications initiative to facilitate training in peer review and to provide appropriate recognition for Early Career Researchers who co-review manuscripts.

Reviewer #4

(Remarks to the Author)
Thank you for the opportunity to review this manuscript. The authors present a chromosome-scale phased genome assembly and a pangenome for European ash (Fraxinus excelsior), constructed from 50 individuals across its distribution range. The primary aims are to characterize structural variation (SV) and to leverage this genomic resource to investigate the genetic basis of low susceptibility to the devastating ash dieback (ADB) disease.
The generation of the BATG-1.0 assembly and a 50-sample pangenome represents a significant and valuable genomic resource for the community. The methodological approach is generally sound and comprehensive, particularly the multi-tool SV calling pipeline and the rigorous strategy for linking dispensable genes to underlying SVs, which avoids overestimation, a common pitfall in pangenome studies.
However, while the data resource is impressive, the study falls short of providing the mechanistic insight and biological validation expected for a journal of Nature Communications' caliber. The central claims regarding the role of SVs in ADB susceptibility remain largely speculative, supported by correlative evidence from re-analyzed existing datasets rather than novel functional insights. The pool-seq GWAS for SVs is convincingly shown to be unreliable, and the SNP-based analysis reveals a highly polygenic and population-specific architecture without pinpointing clear, validated causal factors.

Major Concerns
1. The entire study is computational. The key conclusion, that SVs and dispensable genes contribute to ADB resistance, lacks any experimental support. The validation of key candidate SVs or candidate dispensable genes in resistant vs. susceptible genotypes is crucial to move from correlation to causation.
2. The results in Extended Data Figures 6 and 7 clearly demonstrate that SV allele frequency estimation from pool-seq data is error-prone, leading to a high false positive rate in the CMH test. This fundamentally undermines the entire SV-based GWAS section. The conclusion that no consistent SV associations were found is based on a flawed method.
3. The main finding is that resistance is polygenic and population-specific, which was already known or suspected from previous work (e.g., Stocks et al. 2019, Meger et al. 2024). The pangenome, while a better reference, does not yield transformative new biological insights into the mechanism of resistance in this manuscript.
4. The sampling is heavily skewed towards UK provenances. This limits the generalizability of conclusions about "diverse genetic backgrounds" across Europe. Population structure was not adequately controlled for in the GWAS, potentially inflating false positives.
5. The strong signal on chromosome 15 is intriguing but poorly characterized. The failure to find the causative SV in the pangenome is noted, but no deeper investigation (e.g., targeted assembly of the region in key individuals) is attempted. The content of the region (mostly repetitive) offers no functional clues.

Specific Comments
6. The approach used for genome assembly (PacBio plus ONT) is sound, but the assembly could benefit from additional computational validation. The assembly metrics (N50, L90, BUSCO) are excellent. Consider a more rigorous assessment for assembly completeness.
7. The high percentage of repeats (65.6%) is plausible for ash but could be commented on in relation to assembly completeness. Was this high repeat content well characterized, and does it hinder the identification of novel genes?
8. The annotation protocol using multiple BRAKER3 runs is rigorous but complex. How were conflicts between the short-read and long-read-based annotations ultimately resolved?
9. The number of annotated genes (38,276) is lower than in some other F. excelsior assemblies. Please discuss potential reasons (biological vs. methodological).
10. The pangenome approach is appropriate for SV discovery. The use of Sniffles and cuteSV provides a solid framework for SV calling, but did these methods capture all variations? Was there an assessment for any missed SVs?
11. The saturation curve (Fig. 2B) clearly shows that SV discovery has not plateaued, suggesting more diversity remains to be captured.
12. Why were SVs called in fewer than 3 individuals excluded? While this reduces false positives, it may eliminate rare, functionally important variants.
13. It is noted that 69% of SVs overlap with repeats. Was there any analysis of the type of repeats (LTR, LINE, etc.) associated with SVs?
14. The distribution of SVs across chromosomes (e.g., centromeric vs. telomeric regions) is not shown or discussed.
15. The number of dispensable genes (3,065) identified is notable, but the reasoning for calling a gene dispensable should be carefully assessed. What criteria were used to determine if a gene is dispensable, and how reliable is this categorization?
16. The drastic reduction in dispensable genes (from 34% to 7.8%) after filtering is a crucial result that should be emphasized more.
17. How were genes that overlapped with multiple SVs handled? Was the longest SV or the one with the highest F1 score chosen?
18. The GO term analysis is somewhat weak. The lack of clear enrichment in the high-confidence dispensable gene set is disappointing but perhaps honest. This could be discussed more.
19. For the 133 defense-related dispensable genes, a more detailed analysis is needed (e.g., what protein families do they

belong to? Are any NBS-LRR genes?).
20. The study identifies 362,965 SVs, a large number. What proportion of these are actually relevant for disease resistance versus other ecological traits?
21. The study links SNPs and SVs with low ADB susceptibility. However, GWAS results for low susceptibility to ADB are somewhat inconclusive. Does the paper provide sufficient justification for the statistical approaches used (e.g., CMH test, qGLM)?
22. The quasibinomial GLM test was a good additional analysis to try, and its negative result is important to include.
23. The authors claim to identify loci associated with low disease susceptibility. Were these loci replicated across different European populations, or are they specific to the UK or specific provenances?
24. The GWAS analysis identifies many significant SNPs, but the authors acknowledge potential false positives. What measures are in place to reduce these false positives, and how can future studies improve the reliability of GWAS findings in this context?
25. Patterns of linkage decay between SNPs, SVs, and their potential implications for disease resistance are briefly mentioned. Can the authors provide further exploration of how this could guide future breeding programs?
26. While SV genotyping from poolseq data is promising, the authors mention discrepancies. A deeper discussion of these discrepancies and their impact on the overall results is required.
27. The authors suggest combining variants from different genetic backgrounds for improved resistance. Could this lead to increased heterosis or other unintended consequences? More exploration of the ecological impacts would strengthen the study.
28. The suggestion that crossing populations could improve resistance is logical but remains speculative without empirical data.
29. The inconsistency in the NSZ-107 replicate is concerning and should be discussed as a potential issue with sample pooling or phenotyping.
30. The failure to find the insertion in the pangenome highlights a limitation of the current sampling strategy.

Version 1:

Reviewer comments:

Reviewer #1

(Remarks to the Author)
The authors addressed all of my comments. I don't have further comments.

Reviewer #2

(Remarks to the Author)
The authors did an impressive job in addressing the reviewers comments. We find the answers to our comments and corresponding modifications of their work are overall well done. Among others, they generated quite a lot of new RNA-Seq data for improving the gene models annotation; we believe these changes indeed strengthen the reliability of results and conclusions of the manuscript. The authors also have altered their interpretation of the data in respect to provenance-specific architectures and the limitations of their poolseq data set; they also provide suggestions as to why SNPs previously reported to be associated to ADB tolerance were not detected as candidates in this study. Overall we believe that the current version of the manuscript is fit for publication and provides an important addition in the search for the molecular basis of ADB tolerance.

Reviewer #3

(Remarks to the Author)
I co-reviewed this manuscript with one of the reviewers who provided the listed reports. This is part of the Nature Communications initiative to facilitate training in peer review and to provide appropriate recognition for Early Career Researchers who co-review manuscripts.

Reviewer #4

(Remarks to the Author)
I would like to acknowledge the substantial effort the authors have invested in revising this manuscript. The revised version shows clear improvements in several important respects, including more rigorous analyses, additional validation steps, and a more cautious and balanced interpretation of previously highlighted results. These revisions have meaningfully enhanced the technical robustness and transparency of the study. Moreover, the genomic resources generated here, particularly the chromosome-scale assembly and the pangenome dataset, represent a valuable contribution to the field and will likely be of broad interest to the tree genomics and plant-pathogen interaction communities.
However, despite these improvements, I remain concerned that the manuscript, in its current form, does not yet provide the level of biological insight or conceptual advance for publication in Nature Communications. In particular, the central objective of establishing a clear link between genomic variation and ash dieback susceptibility remain only partially achieved.
1. While the study is technically strong and the authors have taken commendable steps to ensure analytical rigor, there remains a notable gap between the quality of the genomic resource and the strength of the biological conclusions. The main

findings rely largely on correlative analyses, and although the authors appropriately acknowledge the associated limitations, the manuscript does not yet provide sufficiently strong evidence to support a clear biological or mechanistic interpretation of disease susceptibility.
2. The revised manuscript convincingly highlights important limitations in estimating SV allele frequencies from pool-seq data, which in itself constitutes a valuable methodological contribution. However, this also substantially weaken the SV-based association analyses as a foundation for biological inference. Consequently, the role of structural variation in the trait of interest remains unclear. The manuscript therefore appears to have moved away from what initially a central component of the study, without fully established a coherent alternative framework.
3. The SNP-based analyses appear more robust, and the identification of variants showing consistent allele frequency shifts is encouraging. Nevertheless, these results remain challenging to interpret biological context. While the findings suggest a highly polygenic architecture, the study does not yet identify clearly interpretable loci, pathways, or mechanisms that would advance understanding of ash dieback resistance. Further development of this aspect, such as deeper functional annotation, integration with prior studies, or additional validation, would be required to enhance the impact of these findings.
4. The pangenome resource is of high quality and has been constructed with appropriate methodological care. However, its contribution to biological insight remains somewhat limited. At present, the pangenome analysis primarily refines estimates (e.g., of dispensable gene content) and improves methodological rigor, but does not yet clearly demonstrate how it leads to new conceptual understanding of the biological system under investigation.
In summary, I appreciate the substantial improvements made by the authors and recognize the value of the genomic resources presented. Nevertheless, additional work would be required to more convincingly establish the biological significance of the findings and to achieve the level of conceptual or mechanistic advance expected for this journal.

Version 2:

Reviewer comments:

Reviewer #4

(Remarks to the Author)
The authors have further improved the manuscript in response to the previous round of review. In particular, the revised manuscript now more clearly explains how structural variation, gene presence-absence variation, and SNP-based association signals relate to differences in ash dieback susceptibility. I also appreciate the clearer statement of the intended level of biological interpretation and the strengthened discussion of polygenic architecture.
However, while these changes improve clarity and help place the results in better context, I still view the manuscript as a collection of well-executed but relatively separate genomic analyses that are not yet fully integrated into a single, clear model of disease susceptibility. The relationships between structural variation, dispensable gene dynamics, and SNP-based signals are better described, but remain largely interpretative rather than forming a unified mechanistic or predictive framework for ash dieback resistance.
I agree that no additional experimental validation is needed, and I recognize that the study is not intended to be mechanistic. My main remaining concern therefore relates to the level of conceptual integration and whether the manuscript goes beyond a largely descriptive summary of genomic signals. Further strengthening of the conceptual synthesis would improve the manuscript, although I acknowledge the progress made in this revision.

Open Access This Peer Review File is licensed under a Creative Commons Attribution 4.0 International License, which permits use, sharing, adaptation, distribution and reproduction in any medium or format, as long as you give appropriate credit to the original author(s) and the source, provide a link to the Creative Commons license, and indicate if changes were made.
In cases where reviewers are anonymous, credit should be given to 'Anonymous Referee' and the source.
The images or other third party material in this Peer Review File are included in the article's Creative Commons license, unless indicated otherwise in a credit line to the material. If material is not included in the article's Creative Commons license and your intended use is not permitted by statutory regulation or exceeds the permitted use, you will need to obtain permission directly from the copyright holder.
To view a copy of this license, visit https://creativecommons.org/licenses/by/4.0/

REVIEWER COMMENTS

We would like to thank all reviewers for their constructive comments - engaging with them has, we believe, resulted in substantial improvements to the manuscript.

Note 1: each specific reviewer comment has been given an identifier (e.g. ***R1.1, R1.2*** *for the first and second comments from reviewer 1, etc.) for ease of navigation.* **Text of reviewer comments are coloured in green.** *Our responses are in italics. Some of these may be split into multiple parts (if quotes from the manuscript are inserted); the responses begin with numbers if so.* ***Bold text*** *refers to locations in the revised text.* Non-italicised text *is quoted from the revised manuscript.*

Note 2: The original version of this manuscript stated that the individual used to produce the BATG-1.0 reference assembly was produced in 2017 by grafting scion material from the original tree used to produce the first ash genome assembly, BATG-0.5. However, we have since produced short-read sequencing data for the graft to compare to data available for BATG-0.5 and we now no longer believe this graft is from the same individual. 10.4 million heterozygous SNPs, and 4.3 million homozygous alternate SNPs were called mapping the BAG-0.5 sequencing data to BATG-1.0; this compares to 9.7 million heterozygous SNPs and only ~3,200 homozygous alternate SNPs from the BATG-1.0 plant mapped to BATG-1.0. A second ramet was available, which also only showed ~3,000 homozygous alternate SNPs, suggesting material from the same plant was grafted onto the two separate rootstocks, but this was not the same as the original individual used to produce BATG-0.5. This suggests an error/mislabeling in the original grafting process (or which tree was sequenced to produce BATG-0.5) took place. Unfortunately, as the grafting was performed 9 years ago, and the original tree has died, it has not proven possible to identify the true source of BATG-1.0. It is most likely that it represents a tree from the same FRAXIGEN trial at Paradise Wood, Earth Trust, Oxfordshire, UK that was the source of the material sequenced for BATG0.5, but we cannot be certain. We have amended the manuscript, referring now to BATG-1.0 as a tree of unknown provenance. Other than making the specific comparison between the assembly quality of BATG-0.5 and BATG-1.0 less pertinent (which as reviewer 2 pointed out, is a less relevant comparison than with newer pseudochromosomal assemblies from different

individuals anyway), we do not believe this affects any of the conclusions of the paper. All material used to generate RNA-seq and DNA sequence data for the BATG-1.0 assembly was taken from the same individual grafted tree.

Reviewer #1 (Remarks to the Author):

Wood et al. assemble a phased, chromosome-scale Fraxinus excelsior reference (BATG-1.0), build a 50-sample graph pangenome, and re-analyse pool-seq data for loci underlying low susceptibility to ash dieback (ADB). The resource is valuable and timely; the assembly is highly contiguous with telomeric repeats anchoring most major scaffolds, and the pangenome captures extensive SV diversity (≈363k SVs; ≈174 Mb novel sequence). However, several conclusions—especially the size/composition of the "dispensable gene" set and a reported chr15 candidate insertion—rely on assumptions or incomplete validation and would benefit from additional analyses and clarifications.

Major comments
1) Assembly quality and repeat completeness need repeat-aware validation
The assembly is large and contiguous (hap1: 793 Mb, N50 = 32.9 Mb) with many scaffolds showing terminal telomeric motifs, but the size is ~84–90 Mb below the prior 1C estimate (~877 Mb). Repeat content is reported at ~66% (mostly LTRs). Please substantiate repeat completeness:

R1.1
• Compute LAI (with LTR_retriever) and report intact LTR counts/ages per scaffold; compare with Fraxinus/Oleaceae references.

LAI and intact LTR counts have been added to the main text. For comparisons with other recent F. excelsior assemblies, and LAI per chromosome, see Supplementary Table 1. We believe a wider comparison across Fraxinus/the Oleaceae is beyond the scope of the current paper, and our assembly/annotation methods differ from other studies, making it challenging to disentangle biological and methodological causes of differences. However, Supplementary Table S7 in Liu et al. *(2024) reports this information across the Fraxinus genus using a single assembly approach.*

Results: Lines 136-140
"The LTR Assembly Index (LAI) was 14.85 and 14.62 for BATG-1.0 and haplotype 2 respectively, with 5.35-5.44 intact LTRs/Mb (comparable to other recent *Fraxinus* assemblies),

with the top 23 scaffolds in each having average LAIs of 20.74 and 20.62 respectively (Supplementary Table 1) - a score of >10 indicates a reference standard genome[56]

Methods: Lines 581-585

"To assess LTR completeness, LTRharvest from genome-tools v1.6.6 [113] and LTR_finder v1.07[114] were run with default parameters, other than specifying a 85% similarity threshold, length range 100-5000bp, starting/ending motif TG/CA and maximum distance of LTR starting positions of 20,000. LAI was calculated using LTR_retreiver v3.0.4[56]"

R1.2

• Provide a k-mer–based genome size estimate (e.g., GenomeScope from Illumina/HiFi k-mers) to triangulate with flow-cytometry.

• Discuss and, if possible, demonstrate repeat collapse (e.g., coverage profiles showing modal ~2× in collapsed regions) as a likely source of the ~100 Mb shortfall; also note inter-individual genome-size variation.

Coverage of reads mapping to the concatenated fastas of BATG-1.0 and haplotype 2 are displayed in Supplementary Figure 2 (see below). The average coverage to each individual assembly was 77X: a peak of 38X when mapping to both assemblies is approximately as expected (98.3% of reads mapped). There is little evidence of 2x~ modal peaks, suggesting if there are collapsed regions these are likely to be highly repetitive and highly collapsed (e.g. the ribosomal DNA, centromere sequence). We should note that the flow cytometry estimate of 877Mb from Sollars et al. differs substantially from the prime 840Mb estimate from Siljak-Yakovlev et al. 2014 in the plant C-values database, and both of these are from different individuals to the current individual (see Note 2 above). A k-mer genome size estimate from the PacBio input data using GenomeScope gives an estimate of between 823-825Mb. We have added further discussion of this to the text.

Results: Lines 122-136

"The total assembly sizes of 787Mb and 793Mb are smaller than the 1C genome sizes of 877Mb and 840Mb estimated by flow cytometry in Sollars et al.[53] (2017) and Siljak-Yakovlev et al. (2014)[54] respectively - different estimates could result from methodological variation or inter-individual genome size differences. Analysing 27bp kmers from the PacBio reads using GenomeScope[55] gave an estimated genome size range of 823-825.4Mb for this individual (Supplementary Figure 1) - other kmer lengths tested (see Methods) gave very similar size estimates. The coverage of read mapping to both phased assemblies did not show clear peaks of higher depth beyond the main homozygous peak, which would be indicative of collapsed sequences (Supplementary Figure 2). This suggests the remaining difference between

estimated genome sizes and assembly length may be due to highly repetitive regions such as centromeres and ribosomal DNA that are challenging to assemble. The assembly sizes are similar to the size of unpublished pseudochromosomal assemblies of *F. excelsior* that do not use *F. pennsylvanica* for scaffolding (i.e. FRAX_001_PL and daFraExce3.hap1.1; see Supplementary Table 1)."

Methods: Lines 585-587

"To estimate genome size using kmers, LVgs v1.00[115] was run on the PacBio fastq files, with kmer sizes between 17 and 77bp in increments of 10, and results analysed using Genomescope2 v2.1.0[55]"

Supplementary Information: Lines 303-318

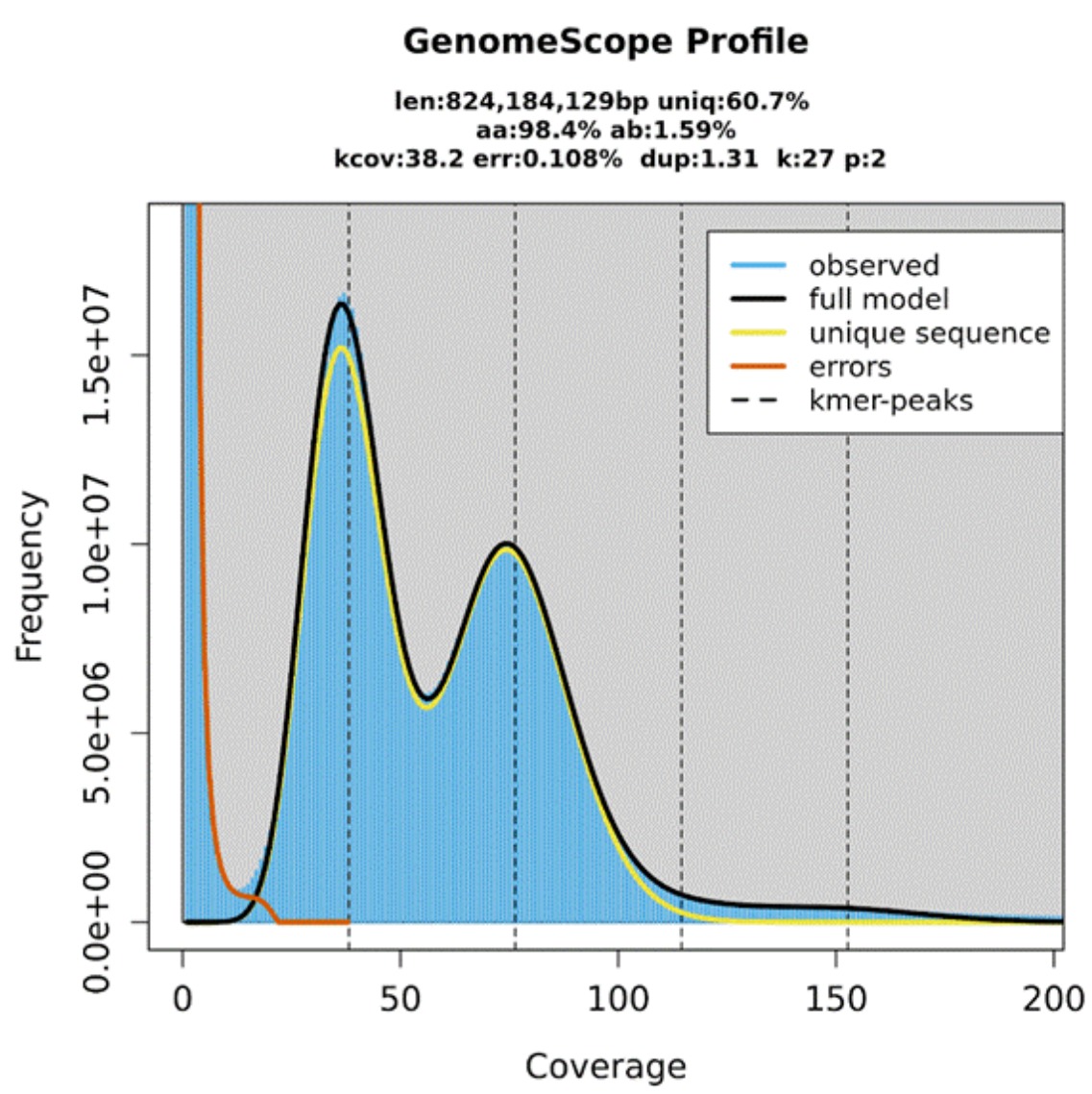

"Supplementary Figure 1: kmer coverage profile for BATG-1.0 PacBio HiFi reads

Frequencies and coverage of 27bp kmers from PacBio HiFi data for the BATG-1.0 individual using Genomescope.

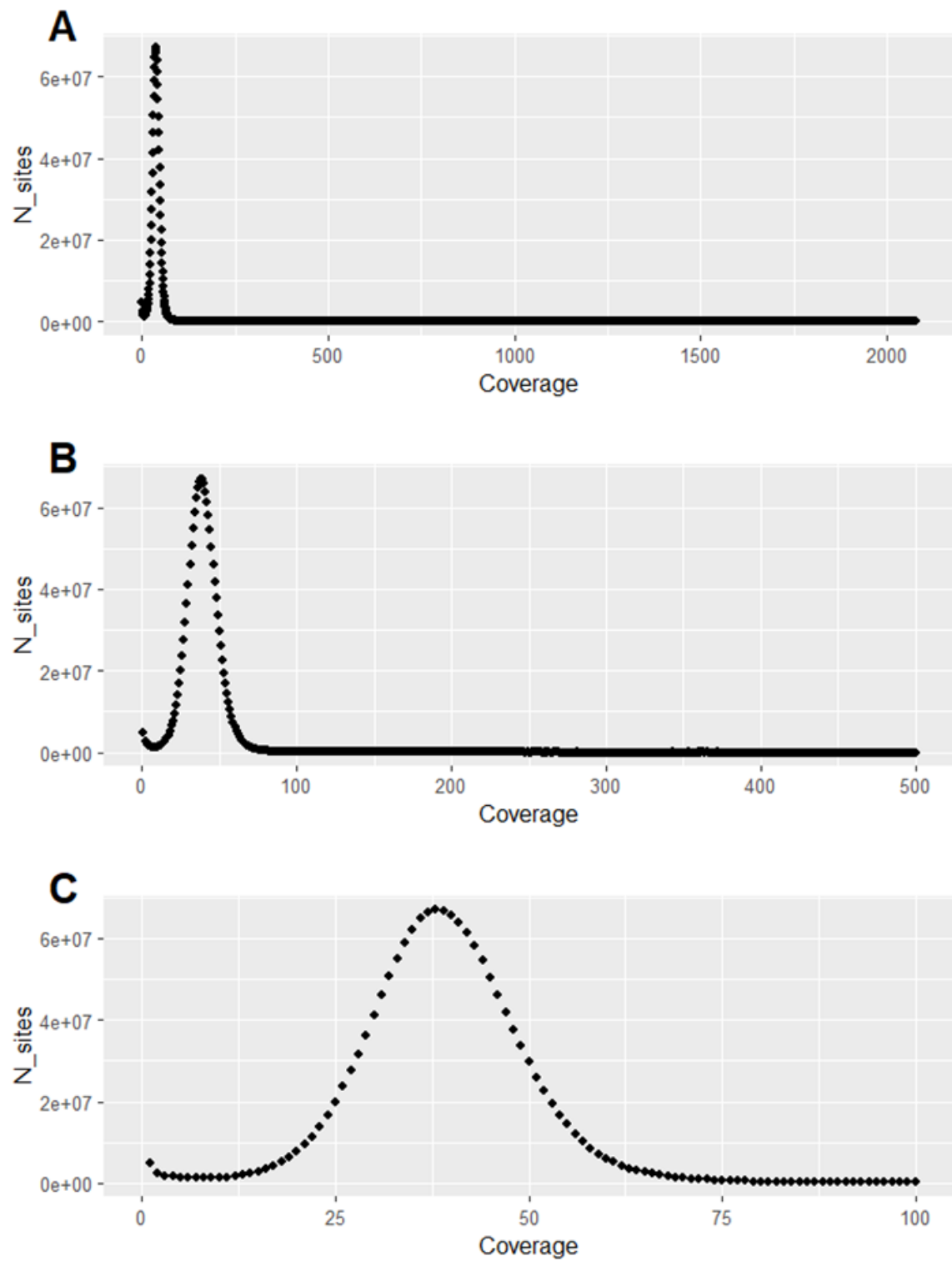

Supplementary Figure 2 – coverage profiles of phased linear reference genome

Coverage profiles of PacBio long-reads used to construct the phased assemblies, mapped to a concatenated fasta file of both haplotypes, with per-site coverage on the X axis, and the number of sites displaying this coverage on the Y axis. For legibility, the same data are displayed with X axis cutoffs of A) 2086 (maximum per-site coverage value was 2076), B) 500 and C) 100."

2) SV calling/merging: precision vs. recall, class coverage, and merging parameters
Your internal benchmark (haplotype-vs-haplotype "truth") yields recall 84.4% and precision 72.2% for the chosen union strategy; you then restrict to SVs seen in ≥3 individuals. This helps, but remains an indirect false positive control. Please:

R1.3

• Report class-specific precision/recall (INS/DEL/INV/TANDUP) against the haplotype truth set; add a second precision check by spot-validating 30–50 SVs (local re-assembly or PCR) in two non-reference samples.

1 .We have reported class specific precision/recall checks as requested, see below. Shasta AND/OR (Sniffles2 and cuteSV) gave the highest F1 for deletions, insertions and tandem duplications. For inversions, using svim-asm + Flye alone gave a slightly higher F1 score than

Shasta AND/OR (Sniffles2 and cuteSV). The overall precision/recall for inversions and tandem duplications were generally lower than insertions and deletions. This has been added to the text.

Results: Lines 199-203

"For insertions, deletions and tandem duplications, this method also gave the highest F1; for inversions, using svim-asm from a Flye *de novo* assembly gave a higher F1, although the overall precision/recall for inversion and tandem repeats was low (Supplementary Figure 4)."

Supplementary Material: Lines 330-347

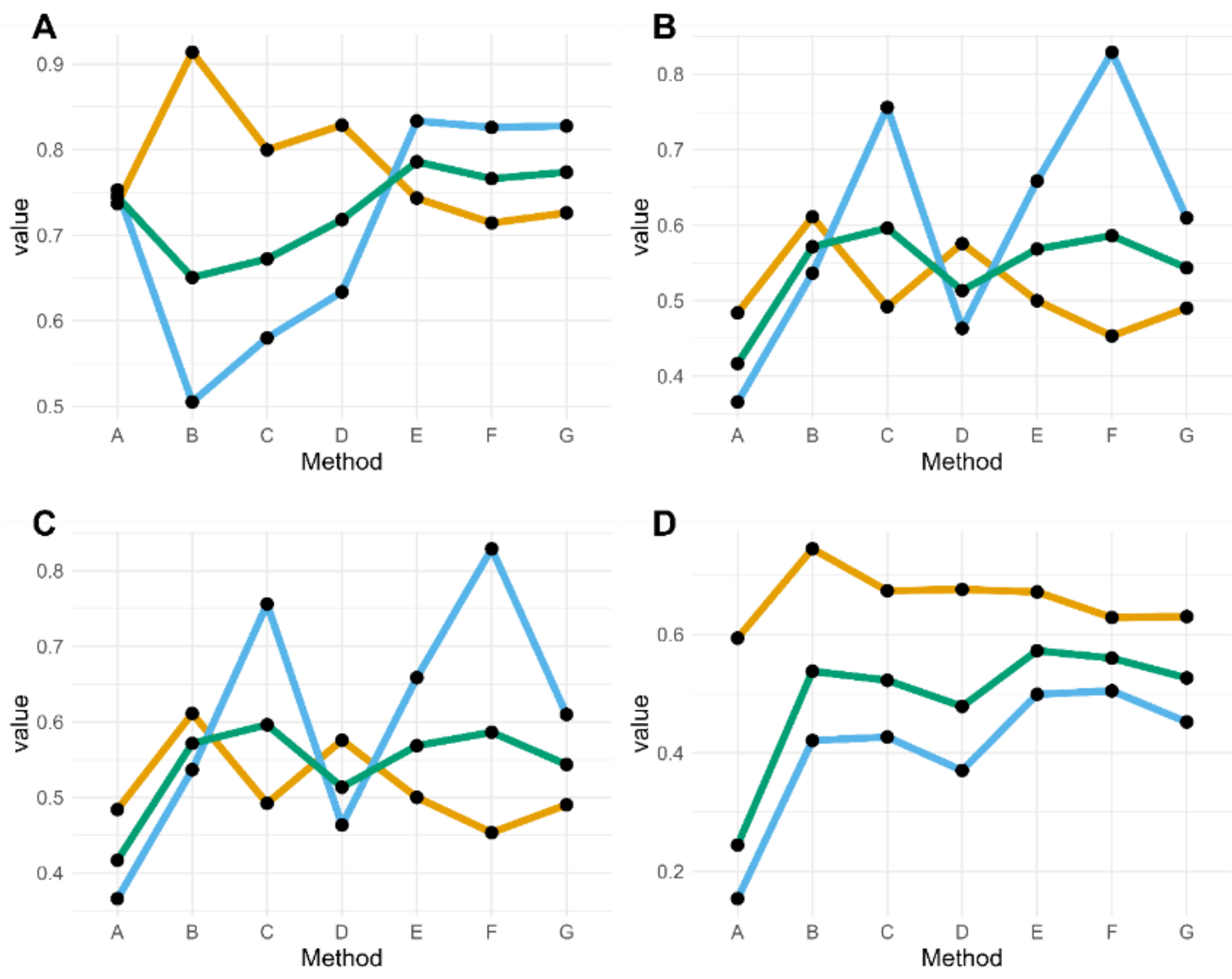

"Supplementary Figure 4 – Class specific precision/recall/F1 of structural variants in BATG-1.0

Values of the precision (orange), recall (blue) and F1 score (green) of structural variant (SV) calling for different methods. Values were based on comparing SVs called using the long-read sequencing data from the reference individual, mapped to BATG-1.0 either as individual reads or assembled contigs, with the "truth" set comprising variants called by mapping the second haplotype of the reference genome to BATG-1.0 and calling SVs with svim-asm. The methods considered were: A) SVs called by both cuteSV and Sniffles2; B) SVs called by svim-asm with a mapping of the *de novo* assembly produced by shasta; C) SVs called by svim-asm with a mapping the *de novo* assembly produced by Flye; D) SVs called by svim-asm with a mapping of *de novo* assembly produced by nextDeNovo; E) SVs called by methods 1 and/or 2; F) SVs called by methods 1 and/or 3; G) SVs called by methods 1 and/or 4. Each panel denotes these values for A) Insertions, B) Deletions, C) Inversions and D) Tandem Duplications."

2. For a second precision check, we calculated for each individual the number of SVs called by i) svim-asm, ii) cuteSV and Sniffles2, iii) svim_asm and cuteSV and Sniffles2, as well as the set called by cuteSV and Sniffles2, and/or svim-asm (as used in the final pangenome set). Calling using svim-asm uses global de novo assemblies mapped to the BATG-1.0 reference genome, which we believe is equivalent to de-novo assembling locally mapped reads. Similarly to the within-BATG-1.0 checks, we find cuteSV+Sniffles2 call a large number of SVs not called by svim-asm, with a smaller proportion of SVs being unique to svim-asm. A median of 41.6% of SVs called by cuteSV + Sniffles2 are also called by svim-asm, and a median of 72.5% of svim-asm SVs are also called by cuteSV + Sniffles2. We have added these points to the text.

Results: Lines 204-207

"Within each sample, a median of 41.6% of SVs called with cuteSV and Sniffles2 were also called with svim-asm, and a median of 72.6% of SV called with svim-asm were also called with cuteSV and Sniffles2 (Supplementary Figure 5)."

Supplementary Information: Lines 352-363

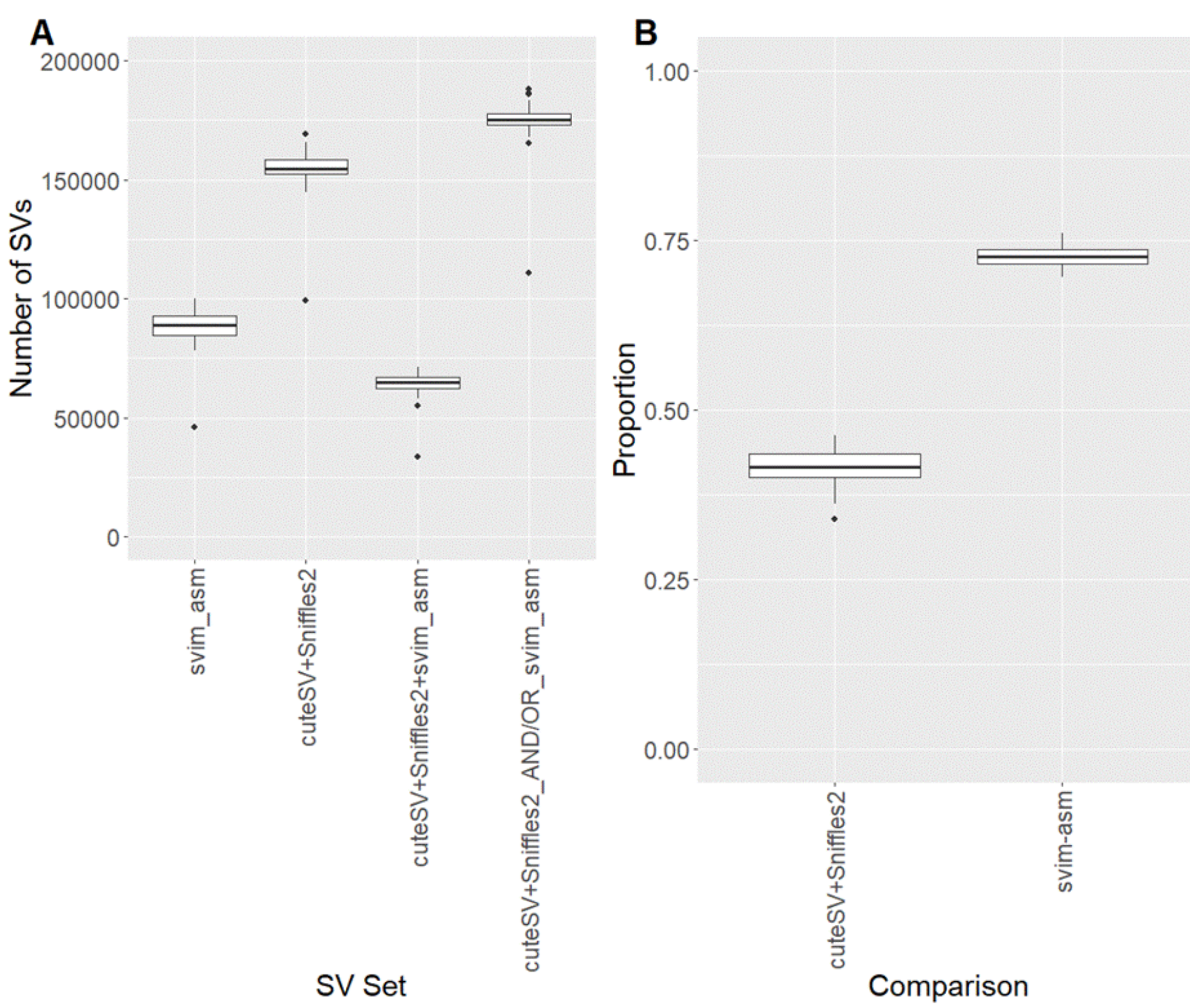

“Supplementary Figure 5 – structural variant caller concordance across different individuals

A: Boxplots showing, for each of the methods outlined on the X axis, the total number of structural variants (SVs) called per sample. Methods are either i) SV called by svim-asm, ii) SVs called by both cuteSV and Sniffles2, iii) SVs called by cuteSV, Sniffles2 and svim-asm, iv) SVs called by either cuteSV and Sniffles2, and/or SVs called by svim-asm. B: Boxplots showing, for each sample, the proportion of total SVs called per sample called by i) cuteSV and Sniffles2, and ii) svim-asm, for the set of SVs called by either/both of these methods.”

R1.4

• Justify SURVIVOR’s 200 bp merge distance and show a sensitivity sweep (100/150/300 bp) to avoid over-merging distinct TE-proximal events.

The threshold for the SURVIVOR merge distance had been based on maximising the number of SVs recovered from merging the cuteSV and Sniffles2 calls based on an earlier version of the pipeline, observing the number of SVs called per sample. We reasoned this would represent a balance between requiring very stringent agreement in breakpoints (reducing the number of SVs reported) and overmerging SVs that should be separate (which would also reduce the number of SVs). Repeating this analysis with the range of merge distance thresholds (plotted below) indicates a marginal impact of SURVIVOR merge distance on total SV numbers between 10bp and 300bp. The SURVIVOR merge distance with the maximum average number of SVs was 100bp; a 200bp merge distance represented a decrease in the number of SVs by 0.5%, suggesting a marginal over-merging of SVs may be taking place, but this is unlikely to significantly impact the results of the study. We have added text justifying our threshold to the text:

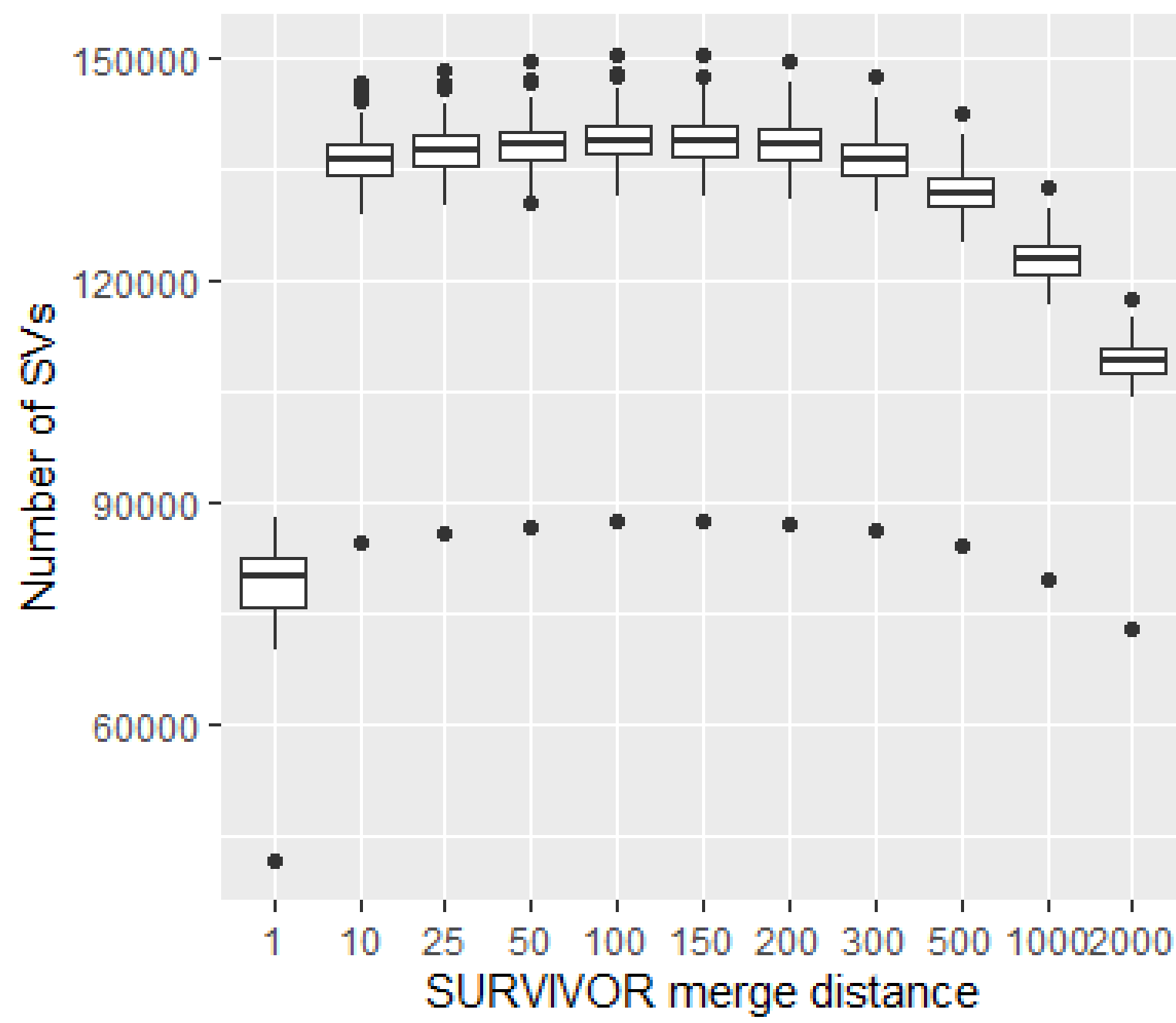

Methods: Lines 741-743

A 200bp merge distance yielded similar numbers of SVs called with Sniffles2 and cuteSV to values between 10 and 300bp yielded similar numbers of SVs, with larger or smaller values resulting in fewer SVs

R1.5

• State up front that BND/translocations were excluded (reasonable for reliability), and—if feasible—add a conservative BND subset with assembly support.

Breakends (BNDs) were excluded from the read-based SV calls, as they do not unambiguously describe a SV. The reviewer is correct that assemblies may resolve some BNDs. Our SV calling methods include SVs based on assembly alignment to the reference genome. Therefore, SVs called as BND from read-based approaches that are supported by our assemblies should already be included in our SV sets. BNDs representing SVs too large to be captured by the assembly based approaches will be excluded - we have updated the text to clarify this.

Methods: Lines 727-728

"Breakends/translocations (BND) or sites which were genotyped as homozygous reference were removed. "

R1.6

• Within individuals you generate a non-overlapping SV set by prioritizing insertions and longer events; please justify biologically and quantify how often shorter, potentially compound events were dropped.

We are not sure which SV set the reviewer is referring to. For producing the SV call sets for the pangenome, SVs were merged within individuals (between SV callers), then across individuals, using SURVIVOR - SVs that passed detection thresholds were all included. Insertions and longer events were not prioritised here.

For the pangenome annotation however, we did generate a non-overlapping set of SVs per individual, as we needed to produce a haploid sequence to annotate. Here we did prioritize insertions and longer events, as we were particularly interested in capturing novel genes not present in the reference. We reasoned that insertions, and longer events, would be more likely to capture novel coding sequence - but it is possible that shorter events or deletions that were excluded also result in novel genes. In most cases, an SV dropped in one sample was still included in a different sample - but the approach did lead to a small number of SVs (2,501; 0.7% of SVs) being unrepresented in any sample. It is possible that a small proportion of novel genes captured by our SVs may have been missed. It should also be noted that as tandem duplicates (0.5% of all SVs) were not able to be incorporated into the graph pangenome, they were also excluded from the annotation process - this was absent in the methods section. We have added these points to the text.

Methods: Lines 809-814

"Where SVs overlapped, insertions were retained in priority to other types of SVs, and longer SVs were retained in priority to shorter ones, as these may be more likely to capture coding sequences absent in BATG-1.0 This process resulted in 2,394 deletions, 96 insertions and 21 inversions being left unrepresented in any of the samples (0.7% of SVs). Tandem duplicates (0.5% of SVs), which were not included in the graph pangenome, were also excluded.

3) Pangenome annotation & the "dispensable gene" set—assumptions that limit inference You annotate each transformed genome using RNA-seq from the reference individual's leaf/cambium and require a consistent SV overlap (called "F1" ≥0.8) to retain genes as dispensable. This is an elegant way to curb annotation artifacts, but it risks false

negatives for genes expressed in other tissues/states and for PAV arising via dispersed duplications or non-local regulatory changes. Please:

R1.7

• Incorporate additional F. excelsior RNA-seq (tissues/seasons/stress) if available, or explicitly acknowledge the limitation.

*1. To address this and other reviewer comments (**R2.1**), we have expanded the tissues, stressors and genotypes of RNA-seq data included in the study. Previously, the BATG-1.0 and the individuals in the pangenome were annotated with short-read RNA-seq data from the leaf, cambium, root and flower of the parent of the BATG-0.5 individual, leaf RNA-seq data from the BATG-0.5 individual, and long-read RNA-seq data from leaf and cambium of the BATG-1.0 individual. To this, we have further added i) short-read leaf data from the BATG-1.0 individual, ii) short-read RNA-seq data from phloem tissue, both from a control individual, and an individual infected with Emerald Ash Borer [from* Doonan et al. *(2023)], and iii) short-read RNA-seq data for petiole tissue from a control individual, and petiole tissue from an individual infected with ash dieback [from* Chano et al. *(2025)*]. *Details of this data is outlined in Supplementary Table 8.*

We also generated a new short-read RNA-seq dataset for leaf tissue for the majority of individuals (36/50) present in the pangenome (it was not possible to generate RNA-seq data from leaf tissue for the remaining individuals due to a lack of suitable plant material; samples included are outlined in Supplementary Table 3). For each of these 36 individuals, RNA-seq data from leaf tissue from that individual was mapped to the corresponding transformed fasta during pangenome annotation, in addition to the expanded RNA-seq datasets described above.

Whilst this should capture a larger proportion of transcripts, false negatives will still remain as we cannot sample every tissue/season/stress for every individual. We have acknowledged this limitation in the text.

Methods: Lines 765-772

"To annotate the linear reference genome, short-read RNA-seq data from leaf, root, cambium and flower tissue that had been used to annotate the BATG-0.5 assembly[53], was downloaded

from the European Nucleotide Archive (ENA), as well as RNA seq data from Emerald Ash Borer infested and control phloem tissue[143], and ADB infested and control petiole tissue[144] (Supplementary Table 8).Together with the short-read leaf RNA-seq data for the BATG-1.0 individual generated as part of this study, these data were mapped to BATG-1.0 using STAR v2.7.10b with default parameters[145]"

Methods Lines 776-779
"We acknowledge that this RNA-seq data does not provide comprehensive coverage of tissues/timepoints/conditions in the species, and that some genes may therefore be missed."

Methods Lines 821-824
"For the 35 non-reference individuals where short-read RNA-seq data was generated as part of this study, leaf RNA-seq data from each individual was also mapped to the corresponding transformed fasta and used in downstream annotation."

2. This additional data did not lead to a substantial change in the number of genes annotated in the reference genome or pangenome, or any qualitative change in our conclusions relating to dispensable genes. However, this may be partially explained by some additional changes we have made to the reference genome and pangenome annotation pipelines, as re-running the analysis drew our attention to some overlooked issues that we were concerned may have affected the results (but ultimately resulted in very little overall change to the results).

Firstly, during the repeated annotation of the reference genome, we noticed a small number of cases where OrthoFinder classified genes with identical start/end sites into different Orthogroups, despite the protein sequence only differing by a small deletion, likely due to failure to annotate a subset of exons. We were concerned that this may inflate the number of genes, and may have impacted our assessment of how often genes are dispensable. We therefore examined overlap between genes - if more than 50% of a gene coding sequence overlapped with another on the same strand, only the gene with the longest total protein coding sequence was retained.

For annotating the pangenome, where OrthoFinder was also used to cluster genes across assemblies, we also tried clustering sequences by coding sequence overlap. When we did this, we identified cases where large groups of non-overlapping genes were clustered into the same "gene", due to overlapping intermediates - with 100 separate BRAKER runs being

clustered, we reasoned this was increasingly possible compared to the reference annotation. We were concerned that this undersplitting could also have an impact on our downstream conclusions. We therefore decided to retain the orthogroups produced by OrthoFinder without clustering by overlapping CDS sequences, even if these in some cases may oversplit similar genes (i.e. the approach displayed in panel A in the figure below).

We discuss this issue at length in Supplementary Note 7 (Supplementary Material Lines 255-300), and present the results from all four combinations of approaches (trimming BATG-1.0 CDS overlapping genes or not, clustering pangenome orthologs by CDS overlaps or not) - in Supplementary Figure 17 (below). The overall proportion of dispensable genes varied from 7.2%-8.7%. Despite our initial concerns, this issue does not appear to have a significant impact on our overall conclusions.

Supplementary Material: Lines 557-581

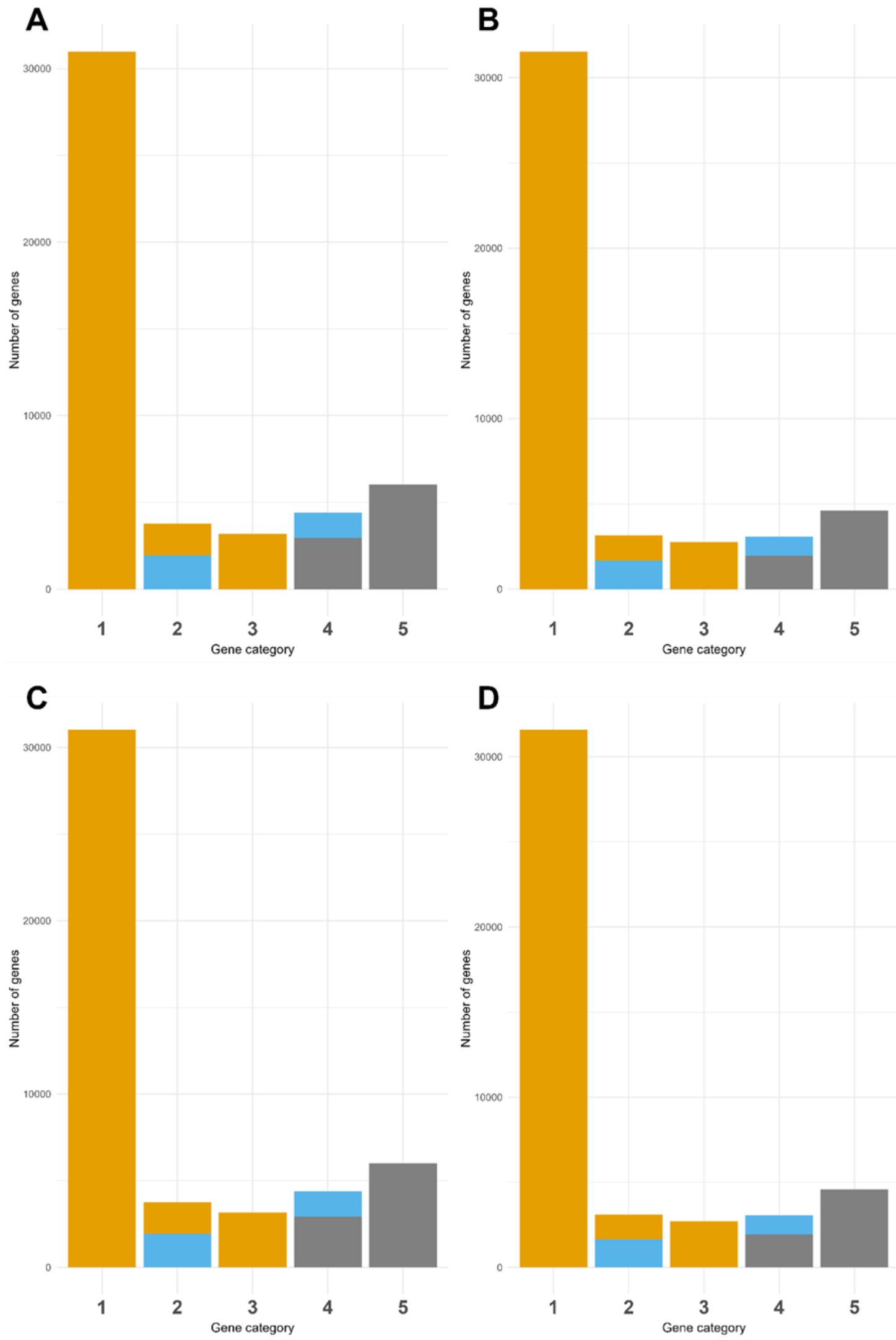

"Supplementary Figure 17 – The impact of different annotation merging approaches on genes classified as dispensable or indispensable

Barplots of the number of genes in each category in the pangenome, where numbers correspond to genes present in the BATG-1.0 reference (1, 2 and 3) and in every other sample (1), or absent in some samples and overlapping with an SV (2), or (3) absent in some samples, but none of these overlap with an SV, or genes that are absent from the BATG-1.0 reference (4,5) and either overlap with an SV (4) or never overlap with an SV (5). Bar colours are based on reclassification of genes in each category as invariant (orange), dispensable (blue), or discarded from the analysis (grey) according to how consistently they overlap with SVs based on an F1 score. Genes in category 2 with an F1 score > 0.8 are classified as dispensable (blue); those with a score below that threshold are categorised as invariant. Genes in category 3 are never associated with an SV so are all categorised as invariant. Genes in category 4 with an F1 score > 0.8 are classified as dispensable, those with a score <= 0.8 are discarded from the

analysis. Genes in category 5 are never associated with an SV so are discarded from the analysis. In an approach where genes absent in some individuals are treated as dispensable, all the genes in categories 2-5 would be treated as dispensable genes. Panels correspond to analysis performed where i) genes in BATG-1.0 that overlapped by >50% in a coding sequence on the same strand had the shortest gene removed (A and B), or not (C and D), and ii) Orthogroups producing during the annotation of the pangenome grouped together if any two members had a >50% of a coding sequence overlapping on the same strand (B and D), or not (A and C)."

3. Secondly, our original approach considered how reliable the relationship was between the presence and absence of particular genes, and particular SVs. However, we became concerned that some SVs may be redundant in terms of affecting gene presence/absence (e.g. different deletions overlapping the same gene), which may have resulted in an artificially low association between gene presence and absence, as different combinations of SVs are present in different samples. We therefore added an additional test; how reliably the presence of any overlapping SV corresponded to gene presence/absence, in additional the relationship between each particular SV and a gene. If the F1 score calculated from this was higher than any individual SV, this was the score reported. This resulted in a very small increase in the number of genes displaying a consistent association with SVs (having an F1 score of > 0.8).

Despite these numerous changes to the annotation pipeline, the patterns of indispensable vs. dispensable genes, and the high frequency of putatively dispensable genes failing to be consistently associated with one/any SV, was overall very similar to our original results. We nevertheless believe that our new annotations are more accurate than those in the original version of the manuscript. The methods have been updated to reflect the changes described above.

Methods - Lines 788-796

"Within each orthogroup, groups of sequences with non-overlapping positions within the BATG-1.0 assembly (likely representing recent duplicates) were separated using the bedtools intersect utility. For each of these groups, the gene model with the longest transcript from the set of 20 BRAKER3 runs was chosen. In some cases, OrthoFinder split genes with largely overlapping coding sequences into separate groups. In cases where two genes with a coding sequence on the same strand overlapped by >50% for one of the genes, only the gene producing the longest protein was retained (for further discussion on clustering see Supplementary Note 7)."

Methods - Lines 844-846

“To account for genes potentially being associated with multiple SVs, we also calculated an F1 score of gene presence/absence with all overlapping SVs - if this scored higher than any individual SV, this score was taken forward.”

R1.8

• Add a read-depth DNA presence/absence check for gene bodies (Illumina/ONT) to rescue genes with weak RNA evidence.

Our substantially improved RNA-seq dataset should reduce the chances of genes being missed due to reduced annotation evidence. Whilst some are likely to be missed, RNA-seq evidence provides important validation that a gene-like sequence is a gene, so we believe that requiring sufficient RNA-seq evidence for annotation is appropriate.

Our annotation approach for the pangenome reduces the likelihood of variable genes erroneously being characterised as missing in some individuals (i.e. as variable) despite gene bodies still being present. The BATG-1.0 sequence is transformed by SVs called in each individual, so the only sequence differences between individual assemblies will be the SVs. For a gene present in some of the individuals to be considered truly missing in others, we require that a DNA sequence change (i.e. an SV) is consistently associated with the difference in the presence of a gene. Even if weak RNA-seq evidence failed to annotate a gene in an individual, its absence would not be classified as truly missing unless an overlapping SV was also present in this individual and in most of the other individuals where the gene was not annotated, as well as being absent in most individuals where the gene was annotated. Therefore our approach implicitly already performs a DNA presence/absence check, in the form of checking association of putative gene presence/absence with DNA sequence differences (i.e. the SVs) between the assemblies.

In studies where separate de-novo assemblies are being annotated, we agree that checking for evidence of a gene body that is putatively absent in that individual (i.e. failing to be annotated) would be an important step. We have added text making this point.

Discussion: Lines 454-458

“We should also note that we aimed to identify genes specifically within our characterised SVs; in studies where *de novo* assemblies are explicitly incorporated (e.g. all-against-all pangenomes

and comparative genomics more broadly) specific testing of DNA sequence differences underlying presence/absence of genes should be performed, as has been suggested elsewhere[99]"

R1.9

• Consider liftover from updated F. excelsior annotations to complement BRAKER3.

We believe that additional F. excelsior annotations are unlikely to capture additional genes in BATG-1.0. Published annotations in Franco Ortega et al. *(2025) and* Liu et al. *(2024) use older versions of BRAKER, and a reduced set of short-read RNA data to annotate their F. excelsior genomes compared to the present study. Additional genes identified by these studies are more likely to result from technical differences between pipelines rather than providing additional biological information.*

R1.10

• Rename the custom "F1" (it is not the standard F1) to SV–gene association score, justify the 0.8 cutoff, and provide a threshold sensitivity analysis (e.g., 0.7/0.9) for dispensable-gene counts and GO enrichments.

1. For our score, we denote N_Gene_SV (number of samples with the gene and the SV) = True Positives (TP), N_Gene_NSV (the number of samples with the gene but without the SV) as false negatives (FN), and N_NGene_SV (number of samples without the gene but with the SV) as false positives (FP).

The formula we quoted in the original manuscript for F1 score is TP / (TP + 0.5×FP + 0.5×FN). The standard F1 is the harmonic mean of precision and recall which can be denoted as F1 = 2×TP / (2×TP + FP + FN) (Christen et al. 2024)*, which is mathematically identical to the previous formula (multiplying numerator and denominator by 2).*

For clarity, we have multiplied the numerator and denominator by 2 in the text to more closely resemble the standard presentation of F1. As far as we can tell, this score corresponds to the standard F1. We are not opposed to changing the name in principle, if the reviewer could expand upon their reasoning.

Methods: Lines 836-837
“an F1 score was calculated to identify the strength of the association between the new gene and the SV: 2 × N_Gene_SV / (2 × N_Gene_SV + N_Gene_NSV + N_NGene_SV)”

2. The choice of 0.8 was somewhat arbitrary - a substantial proportion of genes have an F1 score of 1, but we felt it was appropriate to allow a small degree of inconsistency given that genes sometimes fail to be annotated in repeated BRAKER runs on the same sequence, and overall we believe our annotation approach is fairly conservative. The numbers of gene counts for different values of F1 are detailed in Extended Data Figure 2, panels C and D, which we believe illustrates the sensitivity of the choice of F1 for the number of genes that would be classified as dispensable.

We acknowledge that excluding genes as dispensable based on their F1 score will affect GO enrichment. As set out in the manuscript, the full set of putatively dispensable genes (F1 cutoff = 0) has a number of enriched GO terms that are not enriched in the high confidence set of dispensable genes (F1 cutoff = 0.8) However, the overall observed/expected numbers of genes with enriched GO terms identified from the full set of putative dispensable genes are similar in the reduced set of genes (Figure 4B), indicating that genes with high F1 score have little difference in gene ontology terms compared with the full set of putatively dispensable genes. We therefore do not think characterising the exact F1 threshold that would lead to particular GO terms becoming classified as significant would be particularly informative.

R1.11

• Temper functional language: “133 with GO terms associated with low susceptibility” are annotations, not functional evidence; report enrichment with appropriate backgrounds and FDR/q-values, or frame as hypotheses.

We agree with the reviewer - this is an error in the original text. The genes were annotated with the “defense response” GO term as part of the functional annotation, rather than explicitly associated with ADB resistance by this study. We have tempered the language to reflect this.

Abstract: Lines 34-36

"identifying 3,412 high-confidence dispensable genes, including 141 annotated with gene ontology terms associated with defence response."

R1.12

Note on consistency: The Results state 7.8% dispensable (3,065/39,420), but the Discussion states 6.6%. Please reconcile.

We have updated both points with the updated fraction of dispensable genes (8.7%).

Results: Lines 250-252

"This brought the total number of genes in the pangenome to 39,419, of which 3,412 were dispensable (8.7%% of the total)."

Discussion: Line 431

"We identified 8.7% of genes in the pangenome as dispensable"

R1.13

4) Chromosome-level context of SVs is under-presented
You note that 99.9% of SVs map to the 23 pseudochromosomes and 69% overlap repeats, but a genome-wide view is missing. Please add per-chromosome SV density (e.g., 100 kb windows; INS vs DEL), alongside repeat and gene density, and test enrichment in peri/centromeric regions. This will also help interpret the flat INV/TANDUP trends in Fig. 2B/C (likely low counts + class-specific sensitivity + the ≥3 filter).

*A circular plot with gene density, insertions, deletions, and inversions is added as Figure 2D: not included in this plot were tandem duplications (that are not included in the pangenome), and repeats (that display a uniform density throughout the genome) for reasons of legibility. Individual chromosome plots with all these features (including tandem duplications and repeats) per 100kb are available in Supplementary File 1. Centromere locations have been challenging to reliably locate in ash, and are the focus of ongoing work, so these have not been displayed. We have clarified the point about flat INV/TANDUP trends in the reviewer's specific comment, below (**R1.21**).*

R1.14

5) Graph mapping: quantify biological impact, not just mapping metrics
Mapping to the pangenome reduces soft-clipping by ~33% and modestly improves MQ/AS (~1–1.5%). Please show whether these changes alter callsets (SNP/SV): concordance vs linear mapping, Ti/Tv, HWE, etc., to place mapping improvements in biological context.

We have added further analysis on the impact of mapping to a pangenome on SNP calling as the reviewer suggests. SVs called using short-read data only using only the reference genome are not considered here - we cannot genotype our previously discovered SVs using only the reference sequence. We do not find substantial difference in the properties of called SNPs between reference-only and pangenome SNP calling, after depth and missingness filtering that a realistic analysis would perform (Supplementary Figures 8,9; Supplementary Table 6). It should be noted that a truly comprehensive dissection of the differences in SNP calling between pangenome vs. reference approaches would be interesting, but would require higher depth samples, more species, and a wider variety of SNP calling approaches, which is beyond the scope of this paper.

Results Lines 294-305

"Mapped reads in each case were used to call SNPs - overall numbers of SNPs and quality metrics were similar using both approaches; only 5.6% and 3.4% of SNPs called in the reference-based and pangenome approaches respectively were not called with the other approach. The quality of SNPs that were not detected with both approaches appeared similar to the general background set of SNPs (Supplementary Figures 8,9; Supplementary Table 6). The median distance of unique SNPs from one approach to SNPs from the other approach was <30bp for both callsets, although the reference-based callset included some SNPs >1kb from the nearest SNP in the pangenome-based callsets (Supplementary Figure 9). This indicates that while the improved overall mapping to the pangenome results in some SNP-calling differences, the overall impact on SNP-based analysis is likely to be minimal compared to that associated with using a high-quality linear reference genome."

Discussion: Lines 468-469

"However, there was not a clear improvement in SNP calling compared to using the BATG-1.0 linear reference genome."

Methods: Lines 878-880

"For each mapping approach, SNPs were called using bcftools v1.19 call and calls merged across individuals. Genotypes with DP < 5 or >50 were set to missing, then sites with >10% missing individuals or a MAF of <0.05 were removed."

Supplementary Material: Lines 395-421

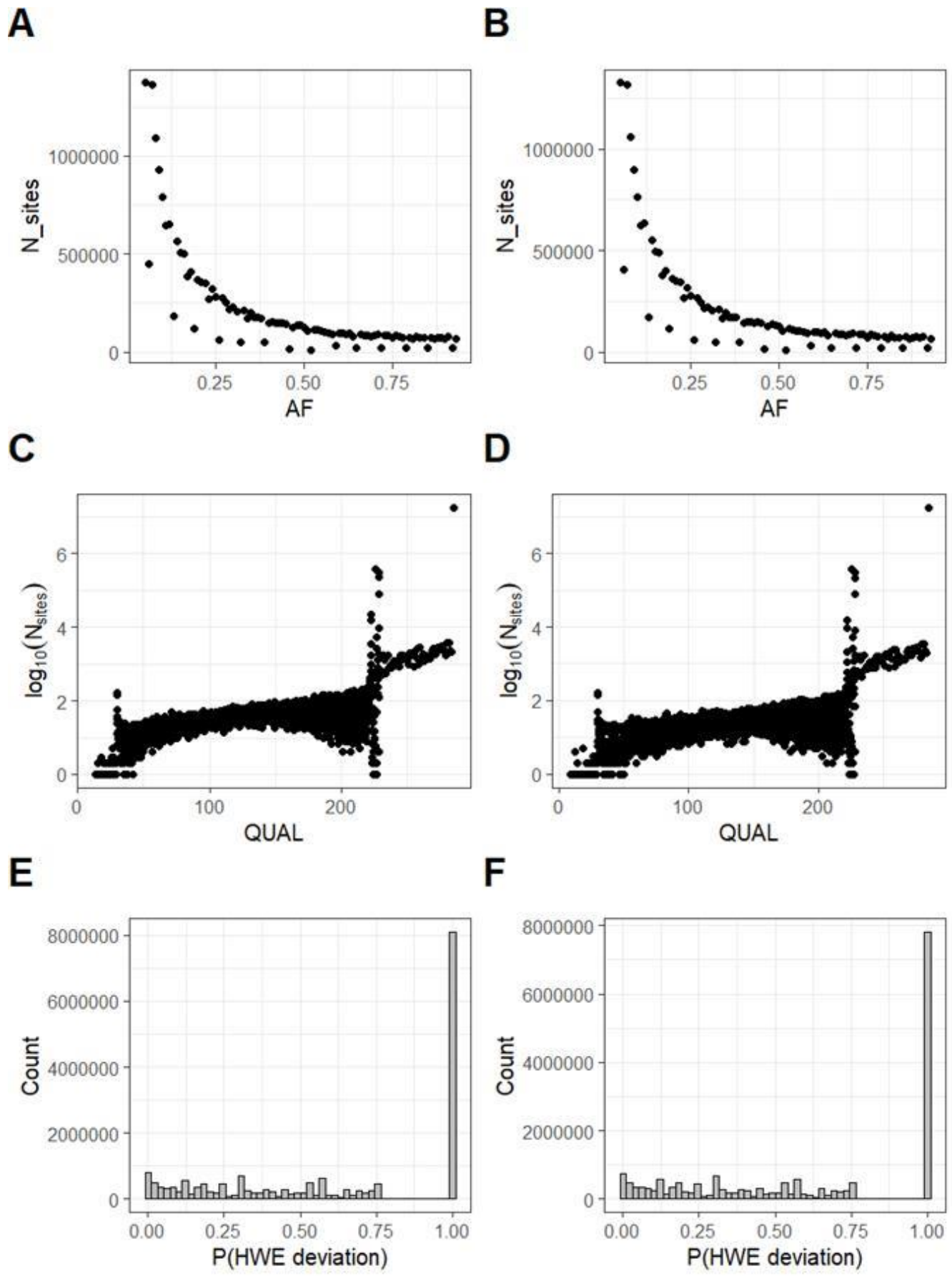

Supplementary Figure 8 – SNP calls from linear reference genome vs. pangenome
For SNPs called using linear reference genome (column 1: panels A, C, E) and the full pangenome (column 2, panels B, D, F), i) the frequency of SNPs with varying levels of allele frequency (AF; panels A + B), ii) the $\log_{10}$(frequency) of QUAL scores (panels C and D), and iii) the frequency of sites with a given probability of deviating from Hardy-Weinburg equilibrium (HWE; panels E and F).

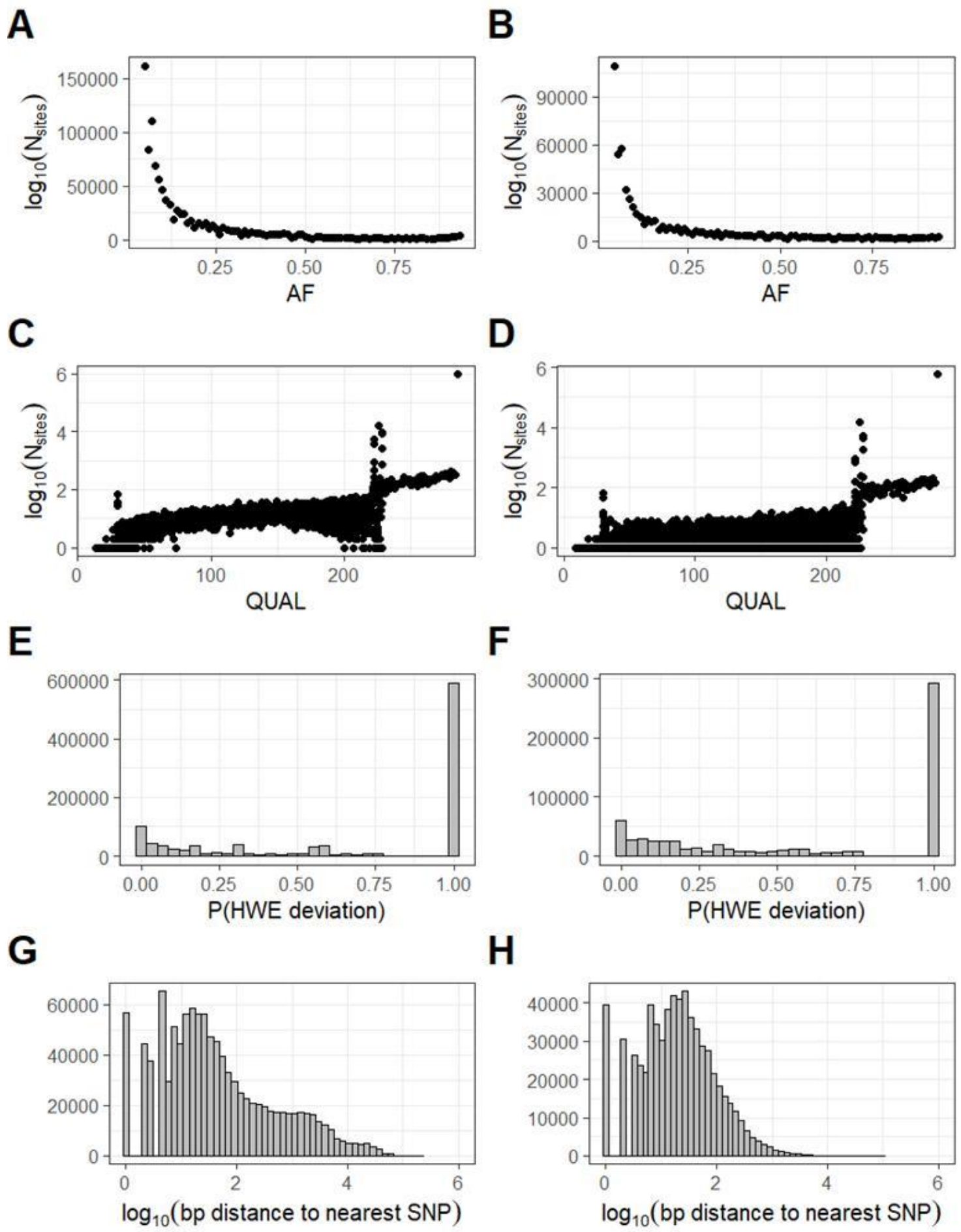

Supplementary Figure 9 – SNPs called exclusively from linear reference genome vs. exclusively from pangenome

For SNPs called exclusively when using linear reference genome (column 1, panels A, C, E and G), and called exclusively when using the pangenome (column 2, panels B, D, F and H), i) the frequency of SNPs with varying levels of allele frequency (AF; panels A and B), ii) the $\log_{10}$(frequency) of QUAL scores (panels C and D), and iii) the frequency of sites with a given probability of deviating from Hardy-Weinburg equilibrium (HWE; panels E and F). The fourth panels indicate the $\log_{10}$(basepair distance) of each SNP, to the closest SNP in the alternate callset (panels G and H)."

6) Pool-seq GWAS: clarify design, apply multiple-testing control, temper claims, and strengthen chr15
Pool-seq SV allele frequencies are noisy (your in-silico validation shows discrepancies), and SV CMH hits are enriched for such discrepancies. CMH SNP hits are largely provenance-specific; qGLM yields none. This fits a highly polygenic trait with strong heterogeneity. Please:

R1.15
• Provide plain-language pool-seq and phenotype summaries (definition of "healthy/unhealthy"; compact recap of Stocks et al. design) in Methods; and apply genome-wide multiple-testing control (FDR/Bonferroni) with QQ/λGC for the CMH SNP analysis (currently a fixed $p < 1\times10^{-13}$ threshold).

1. A plain language summary of poolseq has been added as requested:

Introduction: Lines 94-96
"Reanalysis of poolseq data (i.e. sequence data from a mixture of approximately equal amounts of DNA from multiple individuals) from over 1,200 individuals[42]"

2. A recap of Stocks et al. methods, and definition of healthy/unhealthy has been added to the Results section:

Results: Lines 906-921
"A poolseq dataset from Stocks et al.[42] was used to perform a GWAS for low susceptibility to ADB. In brief, this data was generated as follows: leaf samples were taken from four FR mass screening trial sites in England (including the North Burlingham site where individuals were sampled for constructing the pangenome), from six year old ash trees from a total of 12 different seed sources (10 from UK NSZ, one from Ireland [IRL-DON] and one from Germany [DEU]), as well as a group from a breeding seedling orchard (sourced in the UK) planted by the Future Trees Trust (FTT-SO). For each of the 13 sources, a pool of healthy trees (no visible damage) and a pool of unhealthy trees (main stem dead, or partially dead) - 42 trees per pool on average - were sequenced with the Illumina HiSeq X platform in 150bp paired-end reads. Tree health was assessed based on a tree health score designed to assess the overall level of damage from ADB - it should be noted that low susceptibility to ADB is a complex trait, which may not be associated with long-term tree health, and may be underpinned by multiple underlying mechanisms. Two seed sources had a second pair of pools, produced as biological replicates, for a total of 30 pools (excluding one technical replicate). See Stocks et al.[42] for full details."

3. λ and a QQ plot for the CMH SNP analysis were in the SI in the original manuscript; they are displayed in Supplementary Figure 10A of the revised version. SNPs passing FDR/Bonferonni correction are now reported as requested:

Lines 354-356
"866 SNPs were identified as significant after Bonferroni correction with α = 0.05 (uncorrected p-value threshold = 1.52×10^{-9}), and 404,503 with 5% false discovery rate using the Benjamini-Hochberg procedure."

R1.16
• Use a meta-analysis across provenances (fixed/random effects; Cochran's Q, I^2) to formalize heterogeneity; report the fraction of hits with consistent direction.

The extent to which SNPs shift in consistent directions is plotted in Figure 5 in the revised manuscript, and are discussed throughout the Results (Lines 351-392) and Discussion (Lines 485-529). We calculated Cochran's Q and I^2, using a threshold of $I^2 < 0.25$, to indicate low heterogeneity (Higgins et al. 2003). The fraction of hits with consistent direction are reported. Overall, the I^2 value gave similar results to the Woolf test, as previously reported: for the 866 SNPs with significant CMH test p-values after Bonferroni correction, the I^2 and p(Woolf) are plotted below. The typical threshold of 0.25 for I^2, and 0.05 for p(Woolf test) - plotted as horizontal and vertical lines respectively - in some cases result in differences between SNPs being classified as being heterogenous, but there appears to be a clear mathematical relationship between the two metrics, suggesting they broadly capture similar information. To be conservative, we only considered SNPs as being unlikely to be affected by heterogeneity as a confounding factor, if they had both $I^2 < 0.25$ and p(Woof) < 0.05.

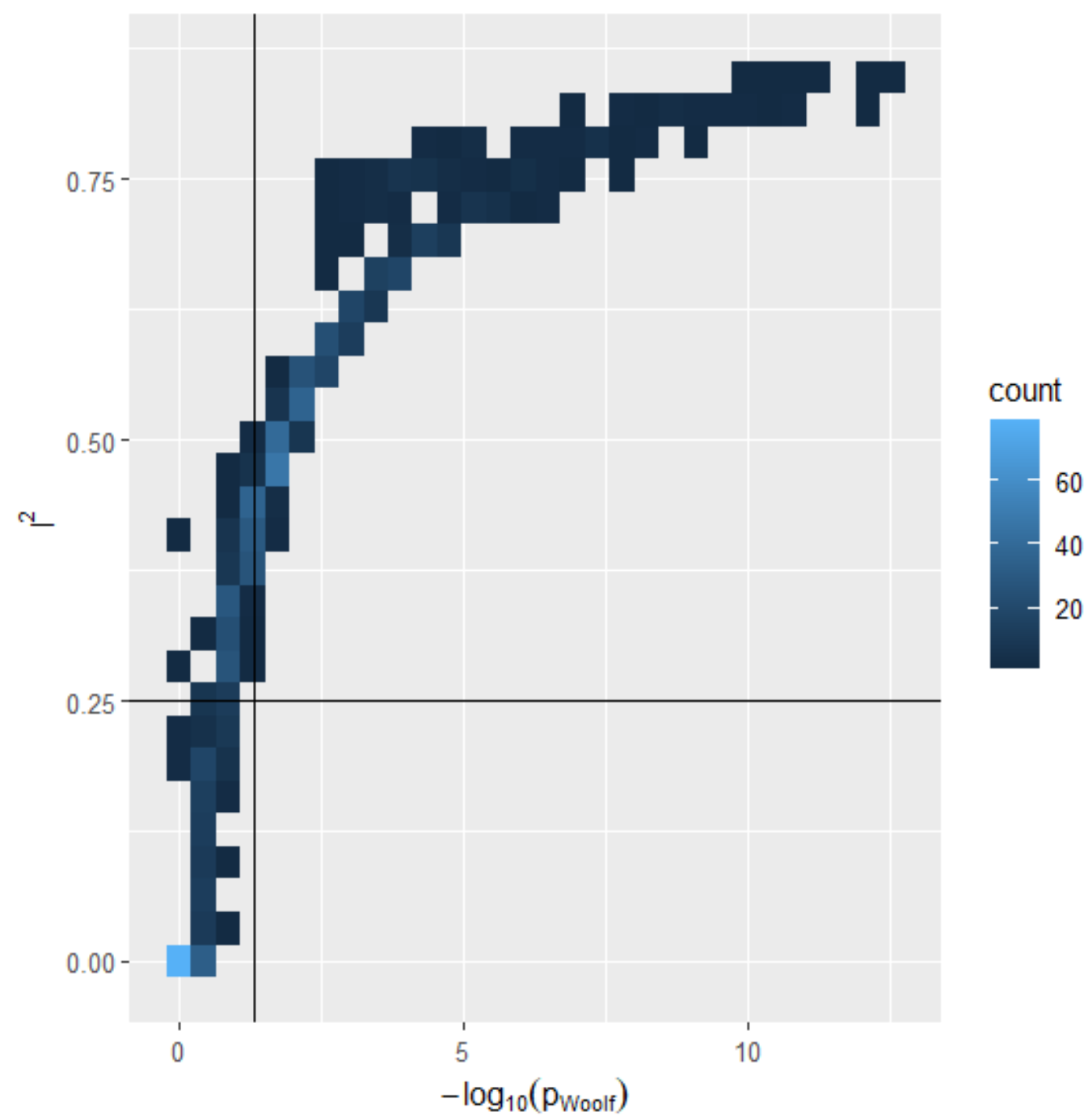

Results: Lines 360-363

"Deviation from homogeneity, as measured either by a significant Woolf test[80] p-value ($p < 0.05$: 53.4% of significant SNPs), or by an I^2 statistic > 0.25 (73.2% of SNPs)[81], was common, and was particularly pronounced for SNPs with the lowest CMH test p-values (Supplementary Figure 11)"

Methods: Lines 939-951

"To quantify heterogeneity in allele frequency shifts, Cochran's Q[73] and I^2 [81] were calculated. Cochran's Q was calculated as $Q = \sum_{i=1}^{k} w_i (x_i - x_w)^2$ from Cochran (1954)[73], with x_i being the estimated effect size from pool i, x_w the weighted mean effect size estimate across all k pairs of pools, and w_i being the inverse of the variance estimated for each pair. For the effect sizes, we used natural logarithm transformed of individual odds ratios [i.e. ln(ad/bc)], where a = alt reads in healthy, b = ref reads in healthy, c = alt reads in unhealthy, d = ref reads in unhealthy) as recommended in Woolf (1955)[80] and performed in other studies[163]. The variance in this measure was estimated as 1/a + 1/b + 1/c + 1/d, as specified in Woolf (1955)[80]. The natural logarithm transformed overall CMH test odds ratio is used as x_w. These values were used to calculate Q. I^2 was calculated from these as $I^2 = (Q - df) / Q$, where df is the degrees of freedom; in this case, the number of pairs of pools minus one."

R1.17

• Temper language in Abstract/Results to emphasize provenance-specific architectures rather than "strong associations."

*We are no longer convinced that our analysis supports provenance-specific architectures - we have substantially altered the Results and Discussion to reflect this. We now believe large allele frequency shifts across subsets of provenances on chromosome 15 are likely the result of rare SVs confounding allele frequency estimation (see reply to comment **R1.18** below). Measures looking at strength of allele frequency shifts between individual pairs of pools (F_{ST}, Fisher's Exact Test) generally detected few overlapping windows of differentiation, even between biological replicates, suggesting these metrics are unable to reliably identify sites undergoing shifts in particular subsets of provenances (this is discussed in Supplementary Note 3). If there are provenance-specific architectures, effect sizes are likely so small that we are unable to detect them using this dataset - only by leveraging information across the pools (using the CMH test) were we able to identify 220 SNPs that behaved broadly consistently across most provenances. This does not exclude the possibility that provenance-specific genetic architectures are important in conferring low ADB susceptibility. We have added text outlining this:*

Results: Lines 378-384

"We attempted to explicitly assess heterogeneity in allele frequency changes across pairs of pools using the Fixation index (F_{ST}) or Fisher's exact test, and considering sites where multiple pairs of pools showed a strongly supported allele frequency shift. However, this proved ineffective for identifying sites shared by subsets of pools (with minimal overlap found even between biological replicates from Stocks et al.[42]), likely due to the inherent noise in individual allele frequency estimates from poolseq data (see Supplementary Note 3)."

Discussion: Lines 505-510

"Variation in the frequency of trees with low susceptibility exists between provenances[35,36], suggesting there may be further geographically restricted genetic variation associated with low ADB susceptibility, although we are not able to accurately characterise this in our dataset."

Supplementary Note 3: Lines 60-84

"To more explicitly test allele frequency shifts within subsets of provenances, we calculated i) Fisher's exact test using the fisher-test.pl function in Popoolation2, and ii) F_{st}, in 500bp/100bp sliding windows using the fst-sliding.pl function in Popoolation2, for each pair of healthy vs. unhealthy pools. We then looked at windows shared across multiple pairs of pools, either by multiple pairs sharing i) a 500bp/100bp sliding window containing a SNP passing a significance

threshold for the Fisher's exact test SNPs ($p < 0.05$ after Bonferroni correction for the number of sliding windows), ii) a window in the top 1% of F_{st} value for each pair of pools. It should be noted that absolute F_{st} may lack power to detect small-effect SNPs diverging over very short timeframes, but regions in the top 1% of values may nevertheless be candidates for differentiated regions.

168,378 windows contained a significant Fisher's exact test SNP in at least one pair of pools - however, only 3,937 of these were shared across more than one pair. For both pairs of biological replicate pools from Stocks et al. 2019, the overlap was very low - only 0.2% of all windows identified across the two pairs of biological replicates were identified as outliers in both pairs. Whilst 101,334 windows were found to have Fst values in the top 1% of values in more than one pool, only 5.1% and 4.9% of the outliers for replicates for NSZ-106 and NSZ-107 that were common to more than one pool, were shared with their respective biological replicate. The clustering of biological replicates in a PCA of SNP allele frequency (Extended Data Figure 6) indicates that in aggregate, these clearly data contain biological signal - but high confidence allele frequency shifts at individual sites are difficult to distinguish from stochastic fluctuations in individual read counts inherent to poolseq data. We therefore do not believe it is possible to detect more geographically restricted genetic variation associated with low ADB susceptibility using the present dataset."

R1.18

• chr15 signal: The tight SNP cluster + depth spikes are intriguing, but no insertion is in your SV catalog. Please rephrase as a candidate insertion-like signal and add targeted validation: (i) local short-read assemblies of depth-spike pools to recover insert/breakpoints; (ii) targeted ONT or amplicon validation in individuals; (iii) screen for TE hallmarks and potential regulatory motifs; (iv) reconcile the NSZ-107 replicate with higher depth in the unhealthy pool.

1. Initially, we attempted local de-novo assembly of reads mapping to this region (R) from pools as the reviewer suggests - this did not identify a single contig that clearly corresponded to all the depth-spike pools. It is possible that more of the pools contain a copy of the insertion-like signal than those with a clear depth-spike, which would make identifying a unique contig challenging. We reasoned it would be easier to resolve this putative SV by considering individually sequenced short-read samples, where available.

We checked whether the 150 individually sequenced samples from Stocks et al., and 542 individually sequenced samples from Metheringham et al. (2025), contained a depth-spike for this region. Due to computational limitations, these reads were mapped to the focal region (R), plus three kilobases upstream/downstream. This will result in some reads erroneously

mapping to this region. However, for 2/692 samples (Individual 58 from Stocks et al. 2019, individual S178C from Metheringham et al. 2025), there were a clear and very large depth spikes in this region (Supplementary Figure 16B, see below). This pattern was recapitulated when sequences for these two samples were mapped to the entire pangenome (Supplementary Figure 16C, 16D). The breakpoints are very similar to the depth-spike region from the pools, and a decrease in depth towards the middle of the region is present in both cases. The mean depth in the focal region for these individuals was 29 times and 33 times the mean depth in the 3kb flanking regions - the variation in the depth increase between these two samples (i.e. 22 times vs. 33 times) could represent copy number/length differences between the samples, or read sampling noise. These samples suggest that presence of a rare, large/high-copy structural variant could explain the depth-spike region in the pools, rather than a smaller but more frequent variant as we had initially thought. A low frequency variant, associated with a large depth spike, likely explains why the two biological replicates of NSZ-107 displayed different patterns in this region since it may only require the inclusion of a single individual carrying this variant within a pool to produce the observed difference between pairs. It may be noted that i) most of the pools in which this variant is putatively detected were healthy, and ii) the 2/692 individually sequenced samples where the variant was detected were healthy (Individual 58 was classified as healthy in Stocks et al. 2019; individual S178C was classified as healthy in 3 consecutive years). However, the variant is at too low a frequency to determine its association with ADB resistance with certainty.

This pattern would also explain why we do not identify the SV in our pangenome. Based on the 692 samples (which represent a seed source from Scotland and a single population in southeast England), the variant has a frequency of <0.3% (and may be less frequent in mainland European samples, that make up a substantial proportion of the pangenome samples). If the depth-spike represents a single structural variant, it is likely ~29-33X the length of region R (~53-61kb). This could represent a long tandem repeat, a high copy number dispersed repeat, or some combination of these scenarios. The likely repetitive nature of the SV makes detection using individual long-reads or de novo assembly potentially challenging.

Supplementary Information: Lines 540-566

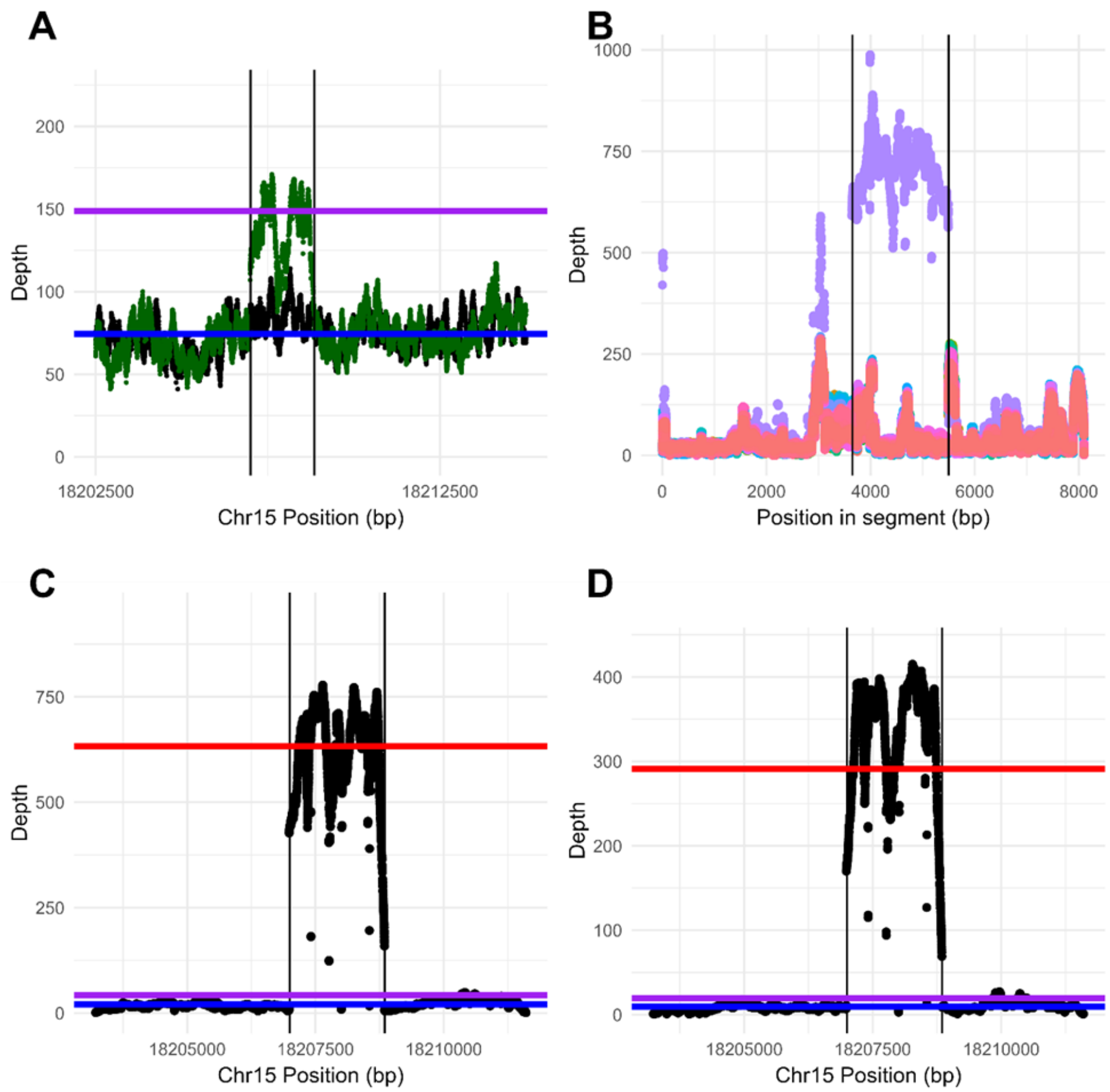

“Supplementary Figure 16 – Read depths across chromosome 15 outlier region from individually sequenced samples

Read depth from reads mapped to the pangenome in the region of the chromosome 15 outlier region for NSZ-302 poolseq data from healthy pool (green) and unhealthy pool (black). B) Read depths from reads from 150 individually sequenced samples from Stocks et al. 2019 mapped to a segment corresponding to the high depth region in A, and 3kb upstream and downstream of this. Depth for each individual is presented in a different colour. Panels C and D represent read depths from Individual 58 from Stocks et al. (2019) (C), and Individual S178C from Metheringham et al. (2025) (D). Vertical lines represent the boundary of the outlier region. Horizontal lines in panels A, C and D represent multiple of the median depth of the flanking region outside the outlier region, for each panel (+/- 3kb) – blue = 1X, purple = 2X, red = 30X. “

2. Whilst a rare, high copy number variant (or combination of variants) seems to be the most plausible explanation for the depth-spikes in the poolseq data, we should stress that without individual sequencing information from the samples in the pools, the exact makeup of the underlying pool is difficult to determine for certain.

We would note this represents an illustration of a fundamental issue in using poolseq data - that copy number variants can be mistaken for allele frequency changes. The presence of a putative low frequency, large structural variant in this case resulted in called SNPs with a depth increase just below our upper limit for filtering. A similarly rare structural variant associated with a ~10-15X depth spike per individual, rather than 29-33X, would likely not result in substantial changes in depth between pools, and remain undetected.

As only a subset of pools showed elevated depth, the average depth of healthy pools was only slightly elevated compared to the genome-wide average (Extended Data Figure 8). However, the standard deviation of depth was more perturbed in the CMH sites with $p < 1 \times 10^{-13}$. We therefore performed post-hoc removal of sites with a standard deviation of depth > 15, which is outside the range of the vast majority of sites in the genome (Extended Data Figure 8), to control for these cases.

In light of the new information presented above, we no longer present the region on Chromosome 15 as a candidate locus for low susceptibility to ADB, and instead focus on a subset of variants showing consistent allele frequency shifts across the majority of pairs of pools - the text of the revised version of the manuscript has been updated throughout to reflect these changes. The issue is discussed in Supplementary Note 1 (Supplementary Material Lines 8-35).

R1.19

7) BUSCO: reconcile genome vs. protein assessments
Assembly BUSCO completeness is ≈98.2–98.3% (genome mode), whereas the protein set shows 94.4% completeness. Please present a small table clarifying genome vs protein BUSCO and discuss why the protein set is lower (conservative consensus across 20 BRAKER runs; tissue bias).

The new annotation increased the protein BUSCO completeness, although there is still some discrepancy between the genome and protein sets. We hope the below illustrates the BUSCO outcomes sufficiently;

Genome: C:98.2%[S:76.5%,D:21.7%],F:0.9%,M:0.9%,n:2326
Proteins: C:98.7%[S:78.6%,D:20.1%],F:0.3%,M:1.0%,n:2326

Small discrepancies between the BUSCO run in protein and genome mode are common (see original BUSCO paper (Simão et al. 2015)*). Duplicated BUSCO genes may result in poorer RNA-seq mapping quality if there is very little sequence divergence between copies, which may hinder annotation and explain why slightly fewer of these are captured in the protein coding genes. Similarly, fragmented BUSCO genes picked up in genome-mode are less likely to be captured during annotation. Equally, it is possible that RNA-seq evidence may resolve a BUSCO gene that was otherwise classified as missing in genome-mode, perhaps due to an uncommon intron sequence. Overall, both BUSCO scores are very high, suggesting that our annotation approach is not overly conservative.*

Figure-specific / presentation comments

R1.20

1. Figure 2 (SV properties).

- **Panel A: Clarify that colors represent different random merge-order replicates (not SV classes).**

This is described in the figure caption. Note that this is now panel B.

Figure 2 Legend: Lines 1040-1045
B) Saturation curves of sequentially merging SVs from different individuals. Each sequential series of SVs was generated by combining individuals in a random order, one at a time, with the total number of SVs in the group being displayed; separate series of random combinations are designated by colour, with points in the same series joined by a line. The X axis represents the number of individuals merged, the Y axis the total number of SVs in those individuals”

R1.21

Panels B/C: Add a sentence explaining the flat INV/TANDUP curves at higher support cutoffs (rarity, class-specific sensitivity, ≥3 filter) and consider adding per-class discovery curves vs. individuals to show whether INS/DEL discovery has plateaued.

The curves for inversions/tandem duplications appear flat when plotted on the same Y axis scale as the much more common insertions and deletions. When each is plotted in a separate panel , the curves look very similar to the insertion/deletion curves - we have added a figure (Supplementary Figure 6, below) to clarify this point. As the number of SVs per individual follows a similar pattern across all SV classes, we think it likely that the per-class discovery curves will also follow a similar pattern.

Figure 2 Caption: Lines 1039-1040

“Note: to aid visualisation, separate plots for inversions and tandem repeats are available in Supplementary Figure 6”

Supplementary Information: Lines 374-383

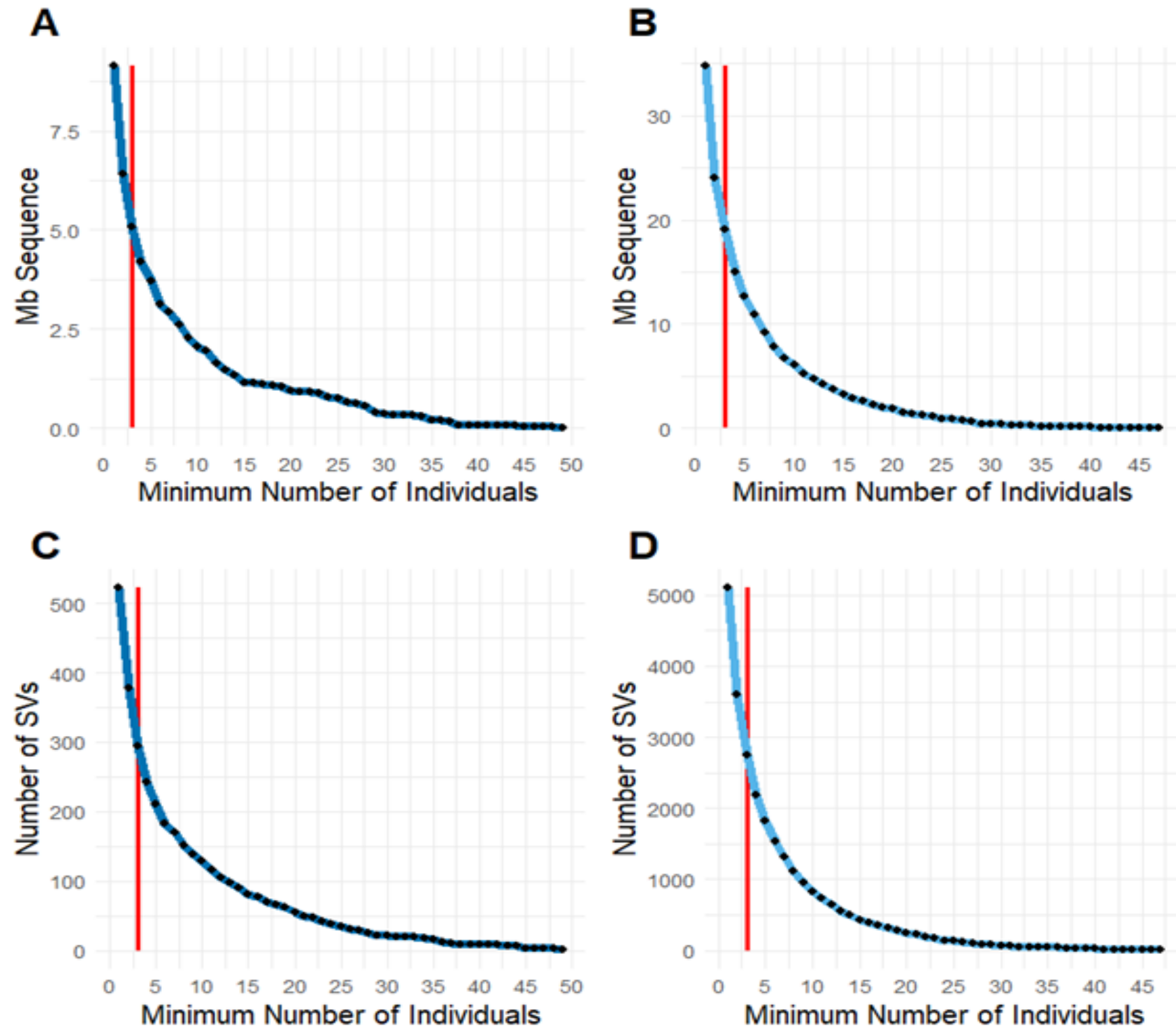

"Supplementary Figure 6 – Inversion and tandem duplicate frequencies

The same data as Figures 2A and 2B are plotted for inversions and tandem duplicates separately to aid visualisation. The total quantity of sequence (A, B) and total number of SVs (C, D) of different SV classes, at different minimum numbers of individuals called from long-read data. Data for inversions are plotted in panels A and C, data for tandem duplications in panels B and D A vertical red line at n = 3 indicates the cutoff for SVs being included in the pangenome."

R1.22

2. Figure 3 (dispensable logic).

- **Rename "F1" in the panel/legend to SV–gene association score (threshold 0.8); clarify in the caption that categories 3 and 5 (no SV overlap) are treated as invariant or discarded, respectively.**

*We have clarified the calculation of the F1 score, see response to general comment 3 (**R1.10**), so have kept F1 in the figure panel/legend for now. The information regarding categories 3 and 5 is in the Figure caption:*

Figure 3 Caption: Lines 1071-1073

"Genes in category 3 are never associated with an SV so are all categorised as invariant. Genes in category 4 with an F1 score > 0.8 are classified as dispensable, those with a score below that are discarded from the analysis."

R1.23

3. Extended Data Figure 1 (SV benchmarking).

- **The methods are independent; lines connecting points imply a trajectory. Please plot points without connecting lines or facet by method.**

The methods are independent, but the purpose of this figure is to aid comparison of the various statistics across the methods. Faceting would make comparisons of statistics between methods more difficult, and with 3 points per facet would not be much more visually interpretable than a table. We tried removing the connecting lines on the original figure, but this made it difficult to interpret visually - we think the line allows quick comparison of each individual statistic between approaches. To avoid the impression of a trajectory, we have replaced the numeric category labels 1-7 with letters A-G to make clear the ordering is arbitrary, we hope this is sufficient.

R1.23

4. Genome-wide SV map.

- **Add a circos/per-chromosome SV density (INS vs DEL) with repeat/gene density to support textual statements and aid biological interpretation.**

*This has been added as Figure 5D: see response to general comment 4 above (**R1.13**).*

R1.24

5. Extended Data numbering.

- **In Fig. 5D you direct readers to ED Fig. 10 for depth plots, but ED Fig. 10 is the pangenome annotation diagram. Please fix the cross-reference.**

This has been corrected.

R1.25

Minor comments (clarity, methods gaps, consistency)

• Define pool-seq, CMH, qGLM, and Fst sliding window on first use; in Fig. 5 caption, note why CMH and Fst are complementary.

Definitions have been added. Figure 5, and the caption referred to, have now been replaced based on the new results.

Introduction: Lines 94-96
"Reanalysis of poolseq data (i.e. sequence data from an a mixture of approximately equal amounts of DNA from multiple individuals)"

Results: Lines 337-341
"We performed a GWAS for low ADB susceptibility using SV allele frequencies estimated from this data, with a Cochran-Mantel Haenszel (CMH) test[73,74]. This test identifies associations of treatments and outcomes while taking into account stratification - it is a commonly used statistic for identifying associations from paired poolseq data[42,71,75–79]**"**

Results: Lines 367-369
"A quasibinomial generalized linear model (qGLM) test - a GLM for proportion/binomial data including an overdispersion parameter - has been shown to be better able to identify consistent allele frequency shifts across heterogeneous pairs of pools[75]**"**

R1.26

• SNP calling (ONT): State per-sample coverage; justify DP filters (DP<8 or >60 set to missing) used for PCA.

Mean depth was 22.7X (assuming a genome size of 840Mb; reported per sample in Supplementary Table 3). Depth filters applied removed sites with depth less than approximately ⅓, or greater than 3x, average read depth, respectively. Sites with excessively high coverage could result from mismapping reads, those with lower depth could be more sensitive to sequencing errors.

Note: in re-examining the SNP calls, we noticed we had erroneously used a vcf produced by Clair3 that does not distinguish reference calls and missing data. This has now been corrected (homozygous reference calls are correctly included) - the overall clustering of the samples is very similar to before, with some rotation relative to the PC axes, and a slight increase in the variance explained by the first two principal components. Previous and current versions of the PCA are displayed below - Figure 1B has been updated with the result of the corrected PCA.

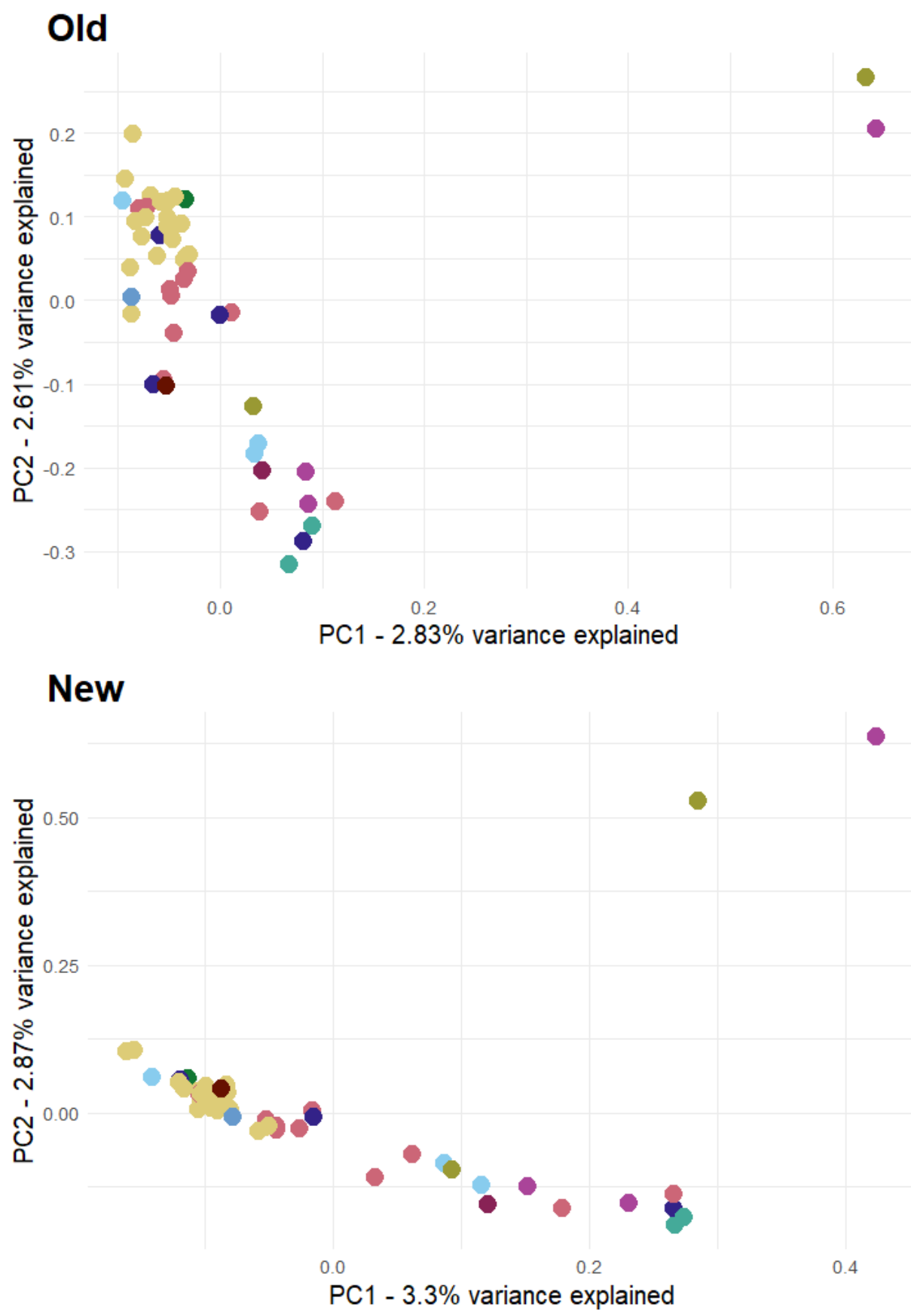

<u>R1.27</u>

• Graph construction: Report basic graph stats (nodes/edges, added sequence per chromosome) and deposit vg indexes alongside VCF/FASTA.

Graph stats have been added. Final versions of the vg indexes, vcfs and fasta will be deposited on Zenodo once revisions are complete.

Results: Lines 226-228

"The set of 362,965 SVs was used to construct a pangenome using vg68 which had 30,710,268 nodes and 31,069,469 edges."

<u>R1.28</u>

• Nomenclature/typos: Standardize "pseudochromosomal" (a few instances read "pseudochromosmal") and use consistent labels ("BATG-1.0" for hap1; "haplotype 2" for hap2).

This has been corrected.

R1.29

• Long-read trimming: You apply Trimmomatic to ONT reads before NanoFilt; this is unusual—please justify or simplify.

We agree this is not usually done, but we believe it is a justifiable step. NanoFilt allows trimming of reads based on length or average read quality, however this does not take into account low quality stretches within otherwise high quality reads - drops in quality towards the beginning and end of ONT data have been observed [Espinosa et al. (2024), Delahaye and Nicolas (2021)]. *Observing the reads using FastQC (see example plots below) showed lower quality bases at the beginning of the reads (we are not aware of software that can easily visualise the ends of the reads in this way, as their length varies substantially). Based on this we decided to quality trim the beginning and end of the ONT reads, a functionality Trimmomatic can perform, resulting in improvement in base quality at the beginning of reads (see below). We do not think any potential simplification by removing this step at the beginning of our pipeline would justify having to repeat the vast majority of analysis in the study.*

FastqQC: Example before Trimmomatic trimming:

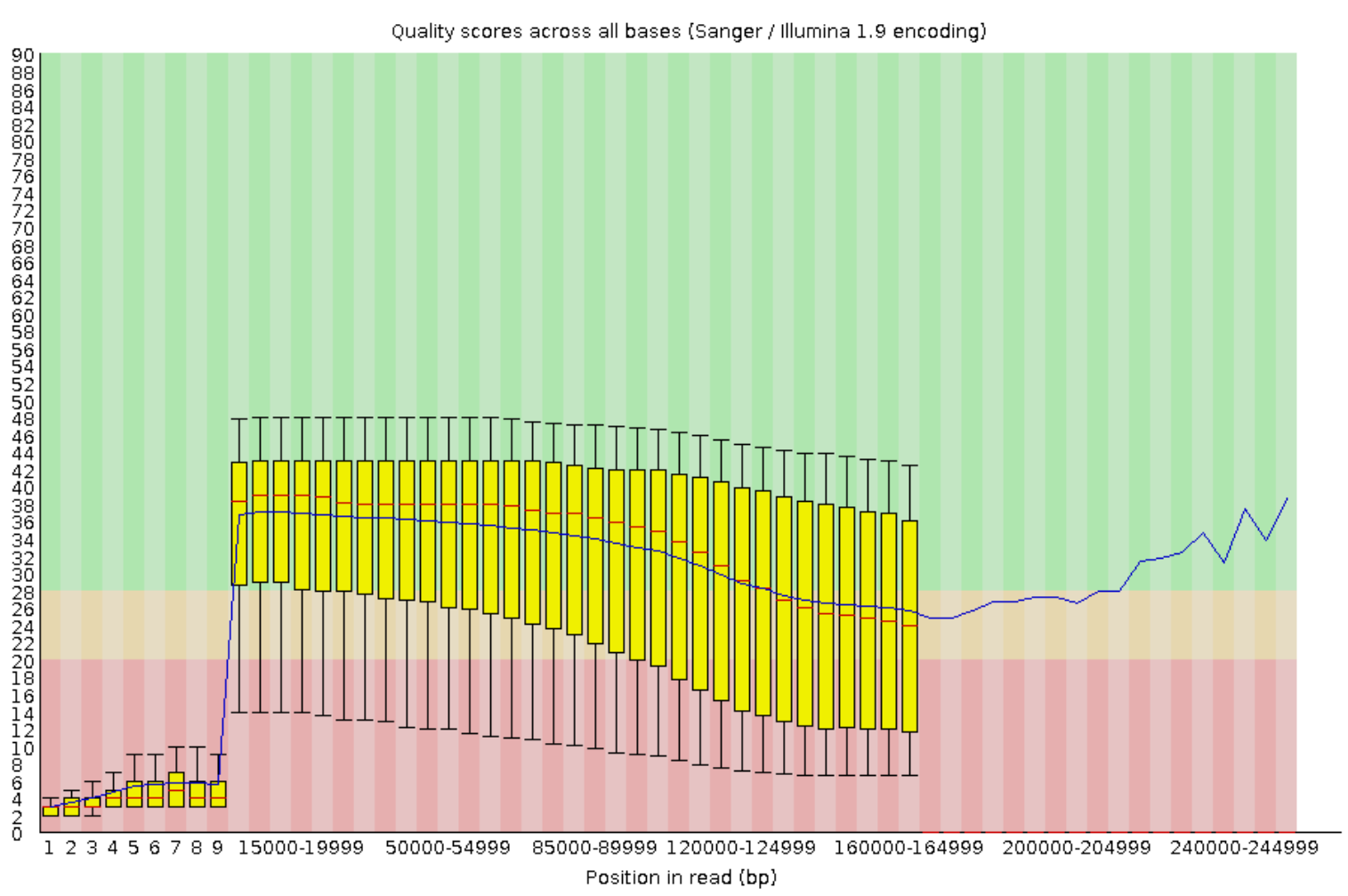

FastQC: Example after Trimmomatic trimming:

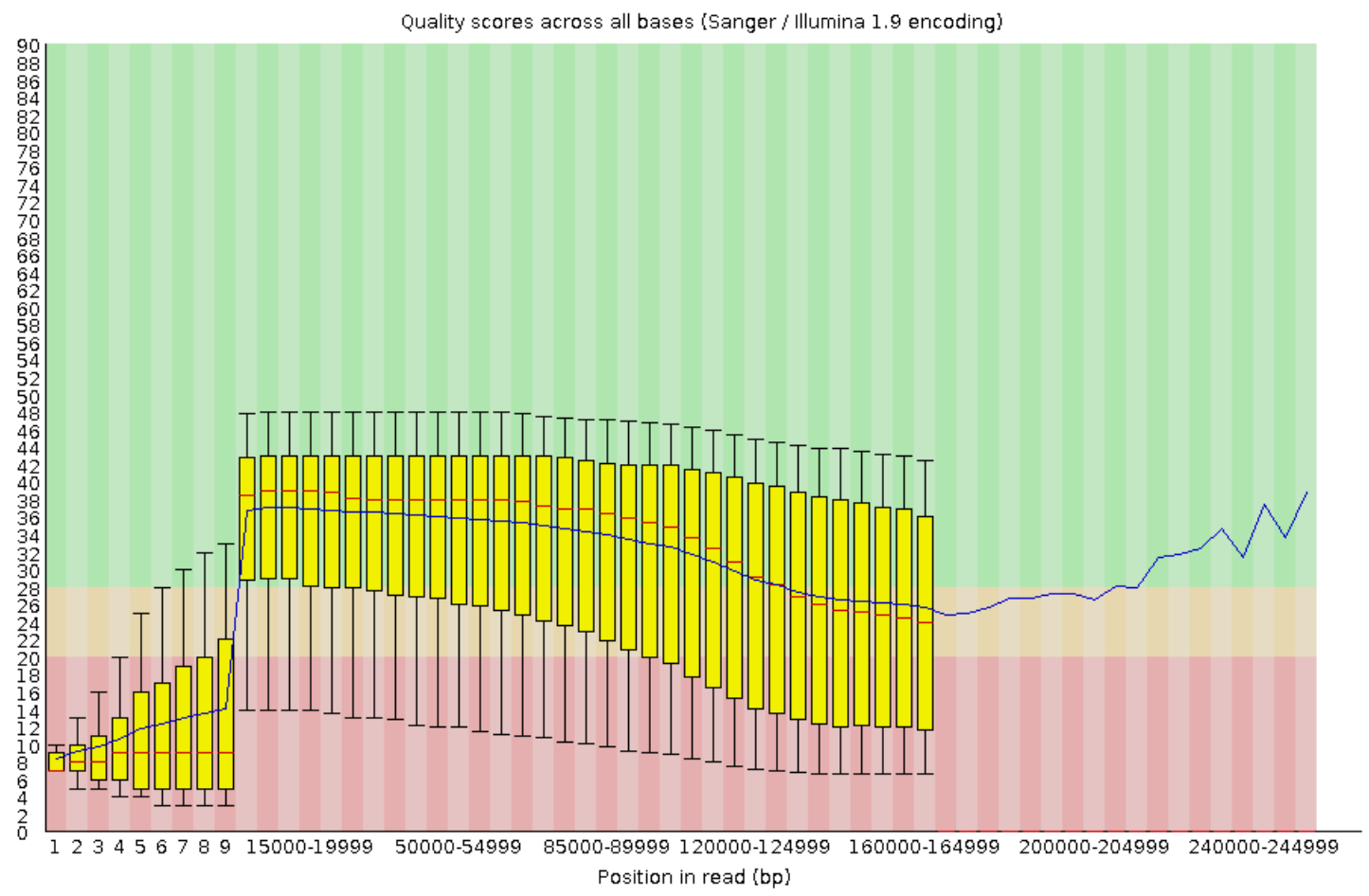

R1.30

• SV thresholds: Explicitly state treatment of partially overlapping SVs within individuals vs across individuals; justify n ≥ 3 and 200 bp merge distance, and add sensitivity.

*We explain the treatment of partially overlapping SVs within and across individuals in response to the reviewers main Comment 2 (**R1.13**), as well as SURVIVOR merge distances, and plotted sensitivity to thresholds.*

*It should be noted that the number of samples required is >3, not >=3 as referred to in the original manuscript (we have corrected this point in the revised version). Reviewer 4 also asked for justification of the frequency based cutoff; further discussion of this point is outlined in the response to their comment (**R4.13**).*

R1.31

• Data/code availability: Replace NCBI accession "XXXXX" with the actual accession; ensure code repo contains pinned environments/containers.

Scripts will be made available at the listed github repo - environments/containers, and final versions of scripts will be uploaded once revisions are complete. Actual NCBI accessions have been added.

Data Availability: Lines 987-989
"Sequencing data is available on NCBI under BioProjects PRJNA906474 (long-read DNA sequence data), PRJNA942609 (long-read RNA sequencing data) and PRJNA1390666 (short-read RNA sequencing data)"

Reviewer #2 (Remarks to the Author):

The study by Wood and coauthors reports the assembly of a pangenome for common ash, with the aim of enhancing the genomic resources available for studying this species, particularly in the context of ash dieback (ADB). This is a highly relevant and timely area of research. The work represents an impressive bioinformatic effort, and the analytical pipelines appear to be both thorough and robust. However, we see four drawbacks that, in our opinion, substantially limit the validity of the results:

R2.1

1) The lack of RNA-seq data for pangenome annotation for any individual other than the linear reference individual and its mother. This data seems very sensitive for a reliable assessment of gene models for individuals across the range and especially dispensable genes not found in the reference individual. Given the strong focus of the work on identifying reliable dispensable genes (see statement already in the abstract at lines 28-30, we "demonstrated that failing to explicitly link SVs…. Can lead to substantial overestimation of dispensable genes"), this lack limits its results and conclusions. Note that gene prediction artefacts could lead to a lack of overlap between dispensable genes and SVs, as suggested also at lines 370-372. Since other RNAseq data is publicly available from other studies it could be a valid option to use it to improve or confirm annotations (even if the individual trees don´t match with the assemblies)?

*We agree with the reviewer - to address this we have significantly expanded the tissues, stressors and genotypes of RNA-seq data included in the annotation, using both publicly available data and newly generated RNA-seq data from individuals included in the pangenome. Reviewer 1 made a similar comment, we have outlined the full details of the revised dataset in response to their comment (**R1.7**). Overall this made very little difference to the final conclusions of the study, giving us confidence that our original conclusions were reliable.*

R2.2

2) We don´t think that the Fst outlier scan approach is the correct analytical approach for ADB susceptibility as not enough time passed since the advent of ADB to justify this approach and we expect limited power, especially given that no study until now suggested the existence of very large-effect loci controlling the response to ADB.

1. We acknowledge that absolute F_{st} may have limited power, although we would note that only relative rather than absolute values are considered (i.e. the top 1% of values), so it should not

be necessary for a variant to have a very large effect to be detected by this approach. We hope it is satisfactory to keep this analysis in the SI, but highlight the notes of caution raised.

Supplementary Information: Lines 67-69
"It should be noted that absolute F_{st} may lack power to detect small-effect SNPs diverging over very short timeframes, but regions in the top 1% of values may nevertheless be candidates for differentiated regions"

2. To further address this point, we performed an additional sliding window measure of differentiation - Fisher's exact test was performed for allele frequency for each individual SNP across corresponding pairs of healthy/unhealthy pools. 500bp/100bp sliding windows containing p-values that were significant after Bonferronni correction ($\alpha = 0.05$) were compared across pools. This approach should not require substantial time having passed to detect outliers.

Ultimately, however, we concluded that neither measure was able to reliably detect outlier regions - the overwhelming majority of windows designated as outlier F_{st} values or contained significant Fisher test SNPs did not overlap across biological replicates. We therefore concluded that any variants shared by subsets of pools, if they exist, likely result in allele frequency shifts too small to accurately detect from this dataset. The presentation of the Fisher's exact test and F_{st} have been moved to the SI, in Supplementary Note 3 (see response to reviewer 1's comment **R1.17** *for further details) We have updated the revised text throughout to reflect this change.*

R2.3
3) It is not clear to us how the same poolseq data from Stocks et al 2019 (SNPs section) which delivered consistent GWAS top candidates using the older reference, does not show consistent results in association outliers across provenances when using the pangenome. Overall, the (inconsistent) results of GWAS using the pangenome is not discussed enough in comparison to the results from Stocks et al 2019. Maybe for this reason, we are not convinced that using the pangenome instead of the linear reference can be defined a substantial improvement.

We are not convinced that Stocks et al. (2019) did deliver consistent top GWAS candidates. Stocks et al. state in their Discussion "we have successfully identified widespread alleles that

are involved in ADB resistance in many populations", but this was not explicitly investigated by examining per-provenance allele frequency shifts in the way we have done in this study. Moreover, this statement was referring to the genomic prediction results, based on using the top 10,000 SNPs from the GWAS, which includes many SNPs that do not pass the statistical significance threshold. In their methods, they state "The Cochran–Mantel–Haenszel test was used to identify significant and consistent allele frequency differences between damaged and healthy trees, with each seed-source pair used as an independent measurement" - this is the purpose of the CMH test, and matches the description of the test in the Popoolation2 documentation, and so (not unreasonably) significant SNPs were taken as evidence of having identified consistent allele frequency changes across many provenances.

*However, the results of Wiberg et al. (2017) and our subsequent reanalysis of the Stocks et al. poolseq data indicate that a SNP showing a significant p-value in the CMH test often does not coincide with consistent allele frequency differences across many provenances. The top SNPs by CMH test p-value in our study often displayed heterogeneity, violating an assumption of the CMH test, resulting in very low p-values (Supplementary Figure 11). Furthermore, we found many sites of the top sites identified by the CMH test displayed elevated depth, which following deeper investigation we concluded likely originates from rare, large/high copy number SVs confounding allele frequency estimation (see response to reviewer 1's comment **R1.18**). Stocks et al. used a much less stringent depth filter than in the present study, potentially making this problem even more severe among their more numerous GWAS hits with very low p-values (192 with $p < 1 \times 10^{-13}$ compared to 92 in this study).*

*New analysis for this revision showed that SNP calling using in the pangenome does not generally appear to be more accurate than using the BATG-1.0 linear reference genome in of itself (see responses to reviewer 1's comment **R1.14**). Indeed, repeating our analysis with BATG-0.5 (the genome assembly used by Stocks et al.) rather than the pangenome, with an identical set of filtering choices, and lifting over the co-ordinates of SNPs passing the 1×10^{-13} p-value threshold, identified a similar set of SNPs as being significant in the CMH test as are found when using the pangenome. However, many of these differed from the 192 SNPs identified as significant in Stocks et al. (outlined in Supplementary Note 4). We therefore*

conclude that variant filtering choices explain most of the differences between these studies, rather than the pangenome per-se. This included the high depth threshold in Stocks et al. described above, as well as different treatment of repeat sequences - if sites overlapped with a repeat sequence or indel in Stocks et al. they were removed, whereas in this study they were retained. We reasoned that the greater contiguity of BATG-1.0, better mapping to a pangenome, and more stringent depth filtering meant this was not necessary. Post-hoc filtering of significant CMH SNPs by violation of heterogeneity, and elevated depth, identified 220 SNPs that showed consistent allele frequency shifts across most provenances tested, and are therefore more likely to be consistently associated with low ADB susceptibility than the top SNPs from Stocks et al. (2019).

*The comparison with Stocks et al. is discussed more explicitly in the revised version of the manuscript, lines 511-529***.** *Whilst the pangenome in of itself may not lead to substantially different SNP calls, we believe our more rigorous analysis of the Stocks et al. poolseq data nevertheless represents a substantial improvement in our understanding of the genetic basis of low ADB susceptibility.*

R2.4

4) The authors explain the lack of genomic regions consistently associated with ADB susceptibility across provenances and pools in the GWAS analyses as a consequence of different genetic backgrounds harbouring different sets of loci involved in susceptibility (lines 34-35, ….). Even if this may be a valid explanation in line with results from other studies (Chano et al 2025, and a preprint from Szukala et al 2024), an additional potential explanation could lie in the way susceptibility was assessed based on the crown damage using a binary assessment (healthy vs susceptible), which is probably affected by a series of variables and may not capture well the quantitative response of the ash trees to the infection. We think that this possibility should be at least discussed also in light of the lack of overlap of top associated loci from GWAS analyses across Europe (Stock et al. 2019; Doonan et al. 2025), which used a similar assessment approach.

In our new analysis, we now identify regions consistently associated with low ADB susceptibility across provenances, and do not believe we are able to detect those behaving more inconsistently using this dataset. In the original manuscript, we focused on SNPs passing the very high p-value threshold of 10^{-13} (the same as used in Stocks et al. 2019) -

*these tended to act inconsistently between replicates, leading us to conclude that the most highly supported SNPs act in a heterogeneous manner. However, many of the SNPs passing the original p-value threshold are likely the result of a rare SV producing erroneous allele frequency estimates (see response to Reviewer 1's comment **R1.18**) - a wider set of SNPs identified as significant after Bonferroni correction were less likely to display this behaviour. We now believe this dataset can only be used to identify SNPs behaving in a broadly consistent manner across pools (see response to the reviewers comment **R2.2** above), and 220 SNPs have been identified that behave in this manner.*

Nevertheless, we agree a visual point estimate of tree health may not fully capture the quantitative response to ash dieback in the long term, and this may make identifying shared SNPs across studies more challenging (although as the samples analysed in this study are the same as in Stocks et al., this is unlikely to explain the differences between our study and that study). We should note that the health assessment was scored on a scale of 1-5, but the poolseq approach effectively requires a binary health score cutoff, as the reviewers suggest. We have added this point to the main text.

Discussion: Lines 520-523
"It may also explain a lack of shared genes between Doonan et al. (2025) and Stocks et al.[44], although we should note that ADB health scores represent a complex phenotype underpinned by several mechanisms, which may reduce the chances of identifying similar underpinning regions across studies."

Methods: Lines 915-919
"Tree health was assessed based on a tree health score designed to assess the overall level of damage from ADB - it should be noted that low susceptibility to ADB is a complex trait, which may not be associated with long-term tree health, and may be underpinned by multiple underlying mechanisms."

R2.5

Minor comments:

Introduction:

1. Lines 48 "the majority of genes" and line 65 "a small fraction of trees" – please be more specific about the amount.

1. Line 48 - quoted studies are those with >50% of genes being dispensable, we have updated the text to reflect this. The exact proportion of dispensable genes across these and other studies is displayed in Supplementary Table 6, referred to in the discussion.

Introduction: Lines 51-53

"These SVs frequently encompass genes, with some studies identifying >50% of genes within a species as dispensable (i.e. not present in all individuals[5,7–9])"

2. "A small fraction of trees" - the exact proportion of trees remaining healthy depends on the population, the definition of healthy, the number of years exposure etc. We have added (~0.5%) to give readers an approximate idea of the numbers of completely healthy trees - this matches the proportion with no visible signs of disease from the 2023 UK Living Ash Project report, but is broadly in line with results from the other studies cited (although towards the lower end - but these studies were performed earlier). We hope this is sufficient.

Introduction: Lines 69-70

"A small fraction of trees (as low as 0.5%[35]) remain healthy under *H. fraxineus* inoculum pressure."

R2.6

Results:

1. Line 98: the smaller size of this assembly compared to previously reported values (almost a 10% difference to 1C = 877 Mb; and a smaller difference to FRAX_001_PL total ungapped length ca 806.3 Mb) even if not unusual probably indicates that the assembly still collapses a good portion of repetitive regions?

*Investigation of reads mapping to the combined phased assemblies did not reveal evidence for collapsed regions in the assembly, suggesting if regions are collapsed they are likely to be highly repetitive - Reviewer 1 raised a similar point, see response to point **R1.2** for full details, and added discussion of variation in assembly sizes.*

Specifically on FRAX_001_PL - we agree our assembly is smaller than FRAX_001_PL. This could be due to biological differences in genome size between individuals, or for technical reasons: as the methods used to construct this assembly have not been published, it is difficult for us to speculate further.

R2.7

2. Lines 130-134; the fact that short reads RNA-seq leads to the discovery of more genes and in general the differences in annotation between short and long reads could be discussed more. Could they be explained by the higher depth of short-reads, which allows for better discovery of genes with low expression? Do short non-coding RNAs detected by short reads but not long reads? Is the BREAKER pipeline more sensitive to short reads –

are long reads collapsed into fewer, longer models? I think these questions highlight the importance of the RNAseq source used for the annotation (see also my first comment).

1. It is difficult to identify the specific technical reasons for differences between the annotation supported by short-read RNA-seq data, and the annotations supported by long-read RNA-seq, in this study, as they are underpinned by data from different biological samples. This disparity is even more pronounced now we have incorporated additional short-read RNA-seq datasets. Most genes are consistently identified across both short and long read datasets. Those genes exclusively identified by the short read datasets had a median length 43% shorter than those exclusively identified by the long reads (See Supplementary Figure 3, below).

Whilst investigating the underlying reasons for these differences is certainly an interesting area, we think this is beyond the scope of this paper - without paired short-read/long-read data from the same biological samples, any conclusions will be highly speculative. We have noted this in the text:

Results: Lines 156-160
"The median gene length of genes exclusively identified by long-reads was 43% longer than those identified only by short reads (Supplementary Figure 3), although different underlying tissue types were used to generate the long-read and short-read RNA-seq data, making these differences challenging to explicitly link to the length of RNA-seq input data."

Supplementary Text: Lines 322-330

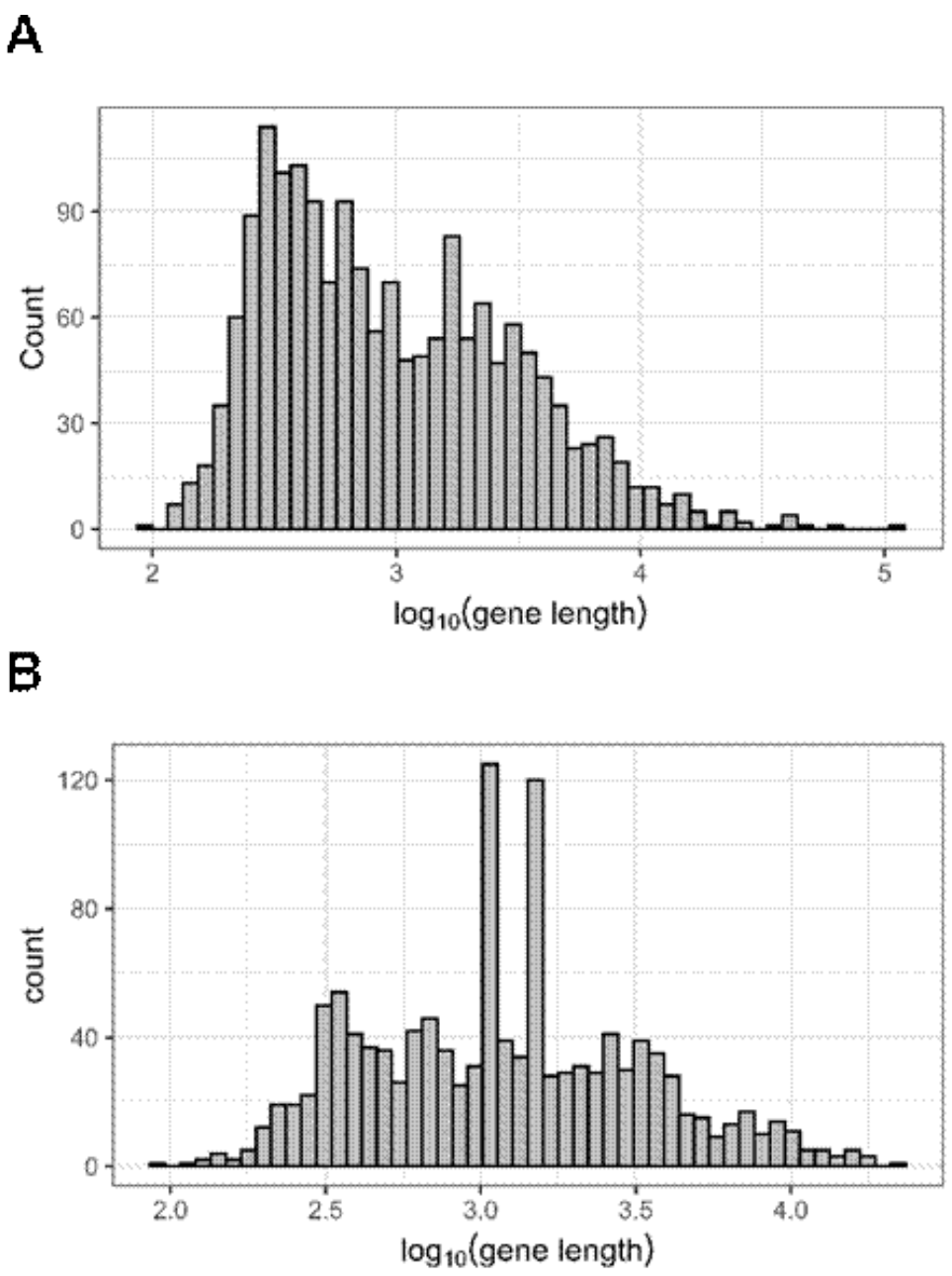

“Supplementary Figure 3 – length distributions for genes identified exclusively with one RNA-seq data type

Histograms of $\log_{10}$ transformed gene length for genes in BATG-1.0 identified exclusively by short-read RNA seq data or long-read RNA seq data. A) Lengths for genes identified in all short-read BRAKER3 runs, but no long-read BRAKER3 runs, and B) genes identified in all long-read BRAKER3 runs, but no short-read BRAKER3 runs.”

2. Considering the complexity of the protein-coding annotation pipeline we felt it better to focus on protein-coding genes in this study, although we agree it would be interesting in future to explore the issue identified on non-coding RNA annotation. We agree the source of RNA is important and with the reviewers’ concerns above, and have expanded the RNA-seq dataset significantly to reflect this (see response to Reviewer 2’s first comment ***R2.1*** *above).*

R2.8

3. Lines 169-173: lack of a plateau in discovered SVs by addition of new individuals. Can you exclude that a technical bias, instead of a biological explanation, lies behind this observation? Can it be that each individual assembly may come with assembly-specific errors (collapsed repeats, misjoins,…) that appear as new SVs?

We agree that assembly-specific errors may produce SVs present only in a few individuals, and that this would be consistent with a lack of a plateau when new individuals are added. This, combined with the analysis of heterozygous SVs in the BATG-1.0 individual (see response to reviewer 4's comment ***R4.13)****, motivated us to exclude SVs present in 3 or fewer individuals. We have added text addressing this point. It should be noted that the number of samples required for an SV to be included in the pangenome was >3, not >=3 as referred to in the original manuscript (we have corrected this point in the revised version).*

Results: Lines 212-215
"It is also possible that sample-specific SVs represent sample-specific assembly or mapping errors - to attempt to control for this, only SVs present in more than three individuals were retained for further analysis (Figure 2A), although this may result in rare SVs being missed."

R2.9
Discussion
1. Lines 329-334 - Improvements of the chromosome-level assembly are reported and highlighted mainly in relation to the BATG-5.0 assembly, but I believe it would be more meaningful to report improvements in relation to pseudochromosomal-level assemblies such as FRAX_001_PL.

We agree with this point. The focus on BATG-0.5 was mainly motivated by the assumption that the material originating from the same individual had been used to construct both BATG-0.5 and BATG-1.0. Further short-read sequencing of the BATG-1.0 individual has revealed that this is not in fact likely to represent the same individual (see Note at the beginning of this document) - we have updated the text to reflect this. We have deleted the particular lines referred to in the Reviewer's comment, as additional discussion of genome size has been added to the results.

R2.10
M & M
1. Lines 466-467: fits better in Results section.

This has been moved to the Results section (Lines 108-111)

R2.11

2. Lines 726-727: 2.2X coverage per individual is very low for GWAS even within the context of poolseq, with much lower resolution and more confounding than individual-level GWAS. Even if this is the same data published in Stocks et al 2019, maybe this low level of resolution should be considered when discussing the GWAS results.

1. Extended Data Figure 6A (see below) indicates that for SNPs there is generally a good correspondence between allele frequencies estimated from the in silico pool (at 2.2X coverage per individual, Y axis), and the allele frequency estimates from analysis of individually sequenced samples (22X per sample, X axis). Whilst we would agree that individual-level GWAS would be desirable, poolseq allows larger sample sizes at reduced cost. Indeed, more recent individual-based GWAS studies had low sample numbers [486 in Doonan et al. (2025), 300 in Meger et al. (2024), compared to 1,250 in Stocks et al. (2019)] and failed to detect significant SNPs associated with low ADB susceptibility, highlighting the tradeoff between sample numbers and per-individual coverage.

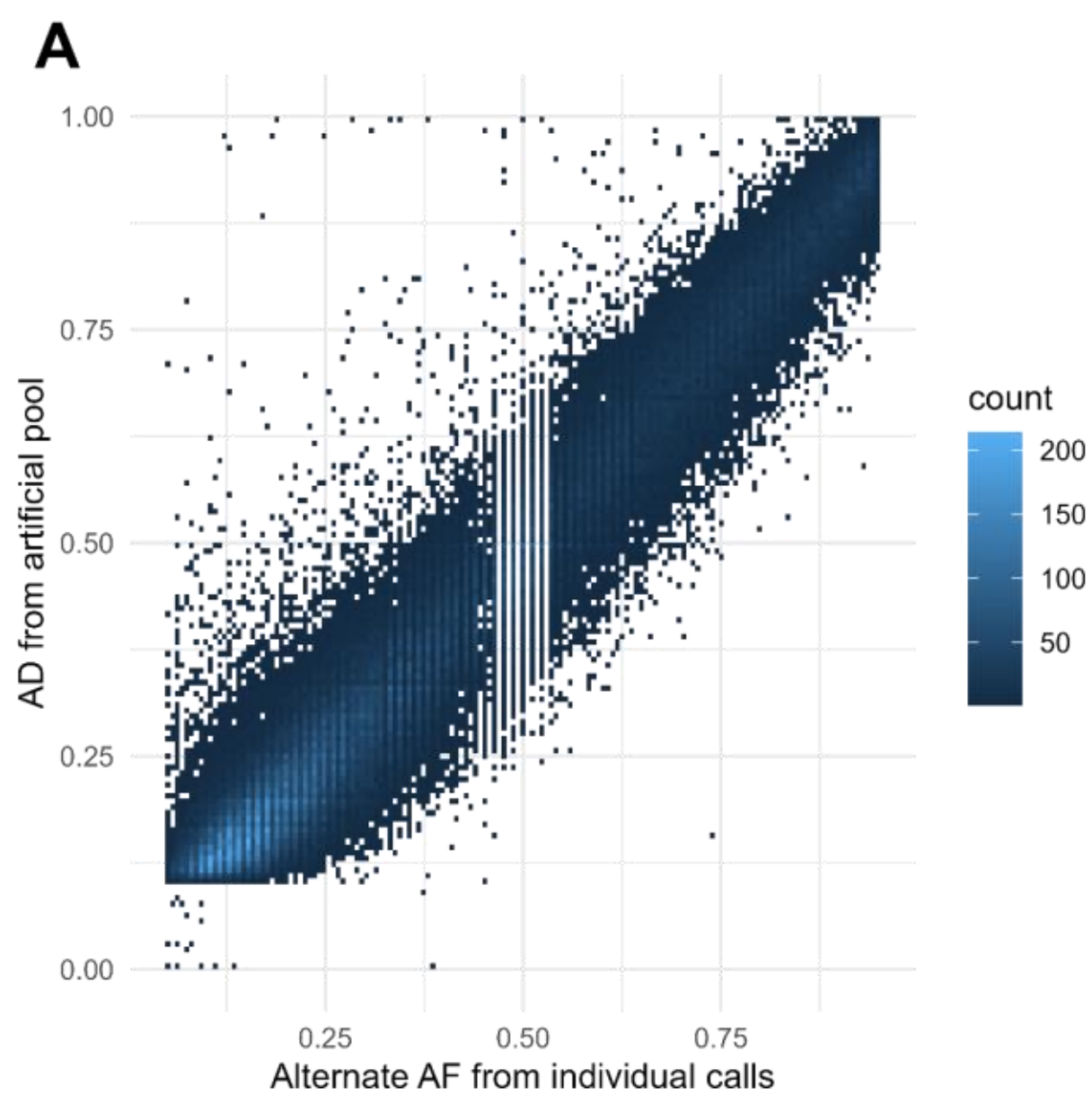

Nevertheless, we agree that a higher coverage per individual, or ideally individual based sequencing of an equivalent number of samples, would give increased accuracy of allele frequency estimation. We have added this point to the main text.

Lines 505-510

"Variation in the frequency of trees with low susceptibility exists between provenances[35,36], suggesting there may be further geographically restricted genetic variation associated with low ADB susceptibility, although we are not able to accurately characterise this in our dataset. Higher coverage per pool, or individual based sequencing, would allow more accurate characterisation of variants restricted to subsets of provenances."

R2.12

3. Line 521: as far as I can see in S Table 3 provenances are from UK, Ireland, Lithuania, France, Denmark, Italy, Czech Republic, Poland, Belgium, Germany, Russia; please report in the text that variation from SE Europe and SW Europe is fully missing.

We have updated the text as requested. The species range, and locations of the origins of samples used in the pangenome, are displayed in Figure 1A.

Methods: Lines 649-651

"We note our sampling does not comprehensively cover the geographic range of *F. excelsior* - in particular, the far south western, south eastern European, west Asian, and far northern areas of the range are unrepresented in our samples (See Figure 1A)."

R2.13

4. Line 568 Since long-reads were used for both genome assembly and RNAseq, pleace specify in the title which long reads.

This has been corrected.

We hope that our remarks help you to improve the manuscript!

We thank the reviewers for their constructive suggestions.

Heino Konrad & Aglaia Szukala
Austrian Research Centre for Forests (BFW), heino.konrad@bfw.gv.at, aglaia.szukala@bfw.gv.at

Aglaia Szukala co-reviewed this manuscript. This is part of the Nature Communications initiative to facilitate training in peer review and to provide appropriate recognition for Early Career Researchers who co-review manuscripts.

Reviewer #3 (Remarks to the Author):

I co-reviewed this manuscript with one of the reviewers who provided the listed reports. This is part of the Nature Communications initiative to facilitate training in peer review

and to provide appropriate recognition for Early Career Researchers who co-review manuscripts.

We thank the reviewer for their contribution.

Reviewer #4 (Remarks to the Author):

R4.1
Thank you for the opportunity to review this manuscript. The authors present a chromosome-scale phased genome assembly and a pangenome for European ash (Fraxinus excelsior), constructed from 50 individuals across its distribution range. The primary aims are to characterize structural variation (SV) and to leverage this genomic resource to investigate the genetic basis of low susceptibility to the devastating ash dieback (ADB) disease.

The generation of the BATG-1.0 assembly and a 50-sample pangenome represents a significant and valuable genomic resource for the community. The methodological approach is generally sound and comprehensive, particularly the multi-tool SV calling pipeline and the rigorous strategy for linking dispensable genes to underlying SVs, which avoids overestimation, a common pitfall in pangenome studies.
However, while the data resource is impressive, the study falls short of providing the mechanistic insight and biological validation expected for a journal of Nature Communications' caliber. The central claims regarding the role of SVs in ADB susceptibility remain largely speculative, supported by correlative evidence from re-analyzed existing datasets rather than novel functional insights. The pool-seq GWAS for SVs is convincingly shown to be unreliable, and the SNP-based analysis reveals a highly polygenic and population-specific architecture without pinpointing clear, validated causal factors.

We are pleased that the reviewer approves of the methods used, and recognises the utility of the resource developed in this study. A powerful feature of pangenomes is the ability to identify novel insights from re-analysing existing datasets. We are glad the reviewer is convinced by our analysis demonstrating GWAS using SV frequencies from poolseq data is unreliable - pangenomics is an emerging field, and in this study we highlight subtle but critical issues in analysing SVs using poolseq datasets with current tools. We therefore avoided making a central claim about the role of SVs in low ADB susceptibility.

Identifying the mechanistic basis of highly polygenic traits is challenging, even in human height, arguably the most comprehensively studied polygenic trait (Yengo et al. 2022). Current

understanding of ADB resistance suggests many different physiological mechanisms are likely to be involved (Schertler et al. 2025). Biologically validating the individual contribution of variants of small effect is both experimentally challenging (requiring many more samples than for a variant of large effect), and of limited value for any given variant (only contributing a tiny amount to the trait of interest). It would be particularly challenging to conduct these experiments in large and slow growing organisms such as trees.

We would argue that identifying genetic variants showing consistent association with ash dieback resistance, rather than identifying the mechanistic basis of low ADB susceptibility, is the more tractable and impactful approach, and should be the priority where time and resources are limited - such as is the case when tackling a novel disease epidemic in a wild species.

Even if the mechanistic basis of resistance was known, without identification of the underlying genetic variants, or those linked to them, this knowledge would be of limited impact in a breeding program or in understanding how natural selection was affecting the trait. A clear understanding of the mechanisms underlying each variant would certainly be useful in predicting the phenotypic outcomes of combining different genetic variants associated with low susceptibility. But in the absence of such a set of variants, an understanding of the mechanisms of resistance would have limited impact in utilising natural variation to produce more resistant trees. Indeed, the approach of genomic prediction, which does not even rely on particularly accurate assessments of the effects of each individual locus, let alone the mechanistic basis of their impact on traits, has proven successful in crop breeding programs and tree species. We believe our identification of SNPs showing broadly consistent association with low ADB susceptibility across provenances, combined with the pangenome data resource, represents an important advance.

Major Concerns

R4.2

1. The entire study is computational. The key conclusion, that SVs and dispensable genes contribute to ADB resistance, lacks any experimental support. The validation of key candidate SVs or candidate dispensable genes in resistant vs. susceptible genotypes is crucial to move from correlation to causation.

In the previous version of the manuscript, we identified a potentially uncharacterised SV, and concluded that it could have a role in low ADB susceptibility. We have revised this conclusion

based on deeper analysis of this particular region, and no longer believe there is compelling evidence to suggest it plays a role in ADB susceptibility (see response to reviewer 1's comment **R1.18***).*

We did not come to specific conclusions about the role of dispensable genes in the level of susceptibility to ADB, but rather highlighted that these encompassed more than a hundred genes with functional annotations related to disease resistance in general.

In the revised version of the manuscript, we identified 211 genes within 10kb of the 220 SNPs associated with low ADB susceptibility, including 16 genes that are dispensable. Experimental validation of these genes' functions could be useful. However, as discussed above, we believe identifying variants that consistently correlate with low ADB susceptibility, even if they are not necessarily causative, is the most efficient way of facilitating ash conservation and breeding programs.

R4.3

2. The results in Extended Data Figures 6 and 7 clearly demonstrate that SV allele frequency estimation from pool-seq data is error-prone, leading to a high false positive rate in the CMH test. This fundamentally undermines the entire SV-based GWAS section. The conclusion that no consistent SV associations were found is based on a flawed method.

*The reviewer is correct that SV allele frequency estimation is error-prone, contributing to a high false-positive rate in the CMH test - this is extensively discussed in the main text (e.g. Lines 280-281 in the original manuscript - "*These results indicate SV allele frequency estimates from poolseq data are insufficiently accurate for a reliable GWAS") *as well as the extended data figures highlighted. We do not believe this undermines this section of the paper - pangenomics is a rapidly developing field, potentially allowing fundamental new insights from the vast array of pre-existing short-read datasets. The section including the SV GWAS highlights that the use of these new tools must be carefully assessed, and in this case more methodological development is required to fully exploit the many existing poolseq datasets using a pangenome framework. We believe this is important information to include that will be of value to the wider pangenomics field.*

We did not conclude that there are no consistent SV associations with low ADB susceptibility - we stated in the results "some of the 531 significant SVs may be truly correlated with low ADB susceptibility" and reiterated in the Discussion "some of the SVs identified as significant from the CMH test are potentially involved in low ADB susceptibility", but ultimately concluded that due to high false positive rates, we cannot be confident in these associations.

R4.4

3. The main finding is that resistance is polygenic and population-specific, which was already known or suspected from previous work (e.g., Stocks et al. 2019, Meger et al. 2024). The pangenome, while a better reference, does not yield transformative new biological insights into the mechanism of resistance in this manuscript.

Based on deeper analysis of the CMH test results, in our revised manuscript we have identified 220 SNPs where we explicitly demonstrate broadly consistent associations with low ADB susceptibility, which does confirm what has been suspected from previous work [whilst Stocks et al. (2019) claimed to have demonstrated this, we do not believe this to be the case, see response to Reviewer 2's comment ***R2.3****].*

*Whilst population-specific variation may exist, we no longer believe it is reliably detectable from this dataset - the manuscript has been revised in light of these findings. Whilst we identify nearby candidate genes worthy of additional investigation, we agree that the study provides limited validated insights into the mechanism of resistance - as outlined in our responses to the reviewers earlier comments (****R4.2, R4.1****) this is not the main focus or value of this study.*

R4.5

4. The sampling is heavily skewed towards UK provenances. This limits the generalizability of conclusions about "diverse genetic backgrounds" across Europe. Population structure was not adequately controlled for in the GWAS, potentially inflating false positives.

1. We agree that the sampling for the pangenome is skewed towards UK provenances and does not represent a comprehensive sampling of European genetic diversity - we should note this partially reflected the fact that the largest short-read datasets for F. excelsior are also

heavily skewed towards UK provenances [e.g. Stocks et al. (2019); Metheringham et al. (2025)]. We have further emphasised this point in the text.

Methods: Lines 649-651
"We note our sampling does not comprehensively cover the geographic range of *F. excelsior* - in particular, the far south western, south eastern European, west Asian, and far northern areas of the range are unrepresented in our samples (See Figure 1A)."

2. The paired structure of the poolseq was designed in order to minimise effects of population structure, as each healthy/unhealthy pair of pools had similar levels of ash dieback damage (so it is unlikely that alleles associated with particular populations would erroneously be associated with high ash dieback damage in this dataset). As far as we are aware, it is not possible to adjust the CMH test to take into account population structure, but the generally consistent allele frequency shifts may be more likely to be biased towards the most densely sampled regions in this dataset (e.g. those sampled from Great Britain). We have highlighted this possibility in the discussion.

Discussion: Lines 499-501
"It should be noted that more densely sampled regions in the Stocks et al.[42] dataset (e.g. Great Britain) may make SNPs consistently shifting in these regions more likely to be identified."

3. We tried to investigate this issue more explicitly via adjustment to the qGLM test, although no positives (true or false) were identified from this test. This is discussed in Supplementary Note 2. As for each SNP we only have 30 data points (allele frequencies for healthy and unhealthy pools across 15 pairs), there are limited degrees of freedom to include additional co-variates that could account for population structure.

Fixed effects for each of the pools (Supplementary Figure 12A) resulted in substantial p-value inflation, as in the CMH test. There is substantial uncertainty in estimating these parameters, and in any case does not take into account relatedness between seed sources. Including the first two principal components of the principal components for SNPs resulted in more modest p-value deflation and identified a single significant SNP.

We also tried i) including pair data as a random effect in a binomial generalized linear mixed model; and ii) also including a random effect of pool itself, to account for high levels of dispersion in poolseq data. Both these approaches resulted in very high p-value inflation (Supplementary Figure 12C, 12D) - we suspect due to difficulties when a single pool has very low/zero alternate allele counts. These results support our initial conclusion, that a quasibinomial GLM approach does not find SNPs consistently associated with tree health across most pools.

Supplementary Material: Lines 486-496

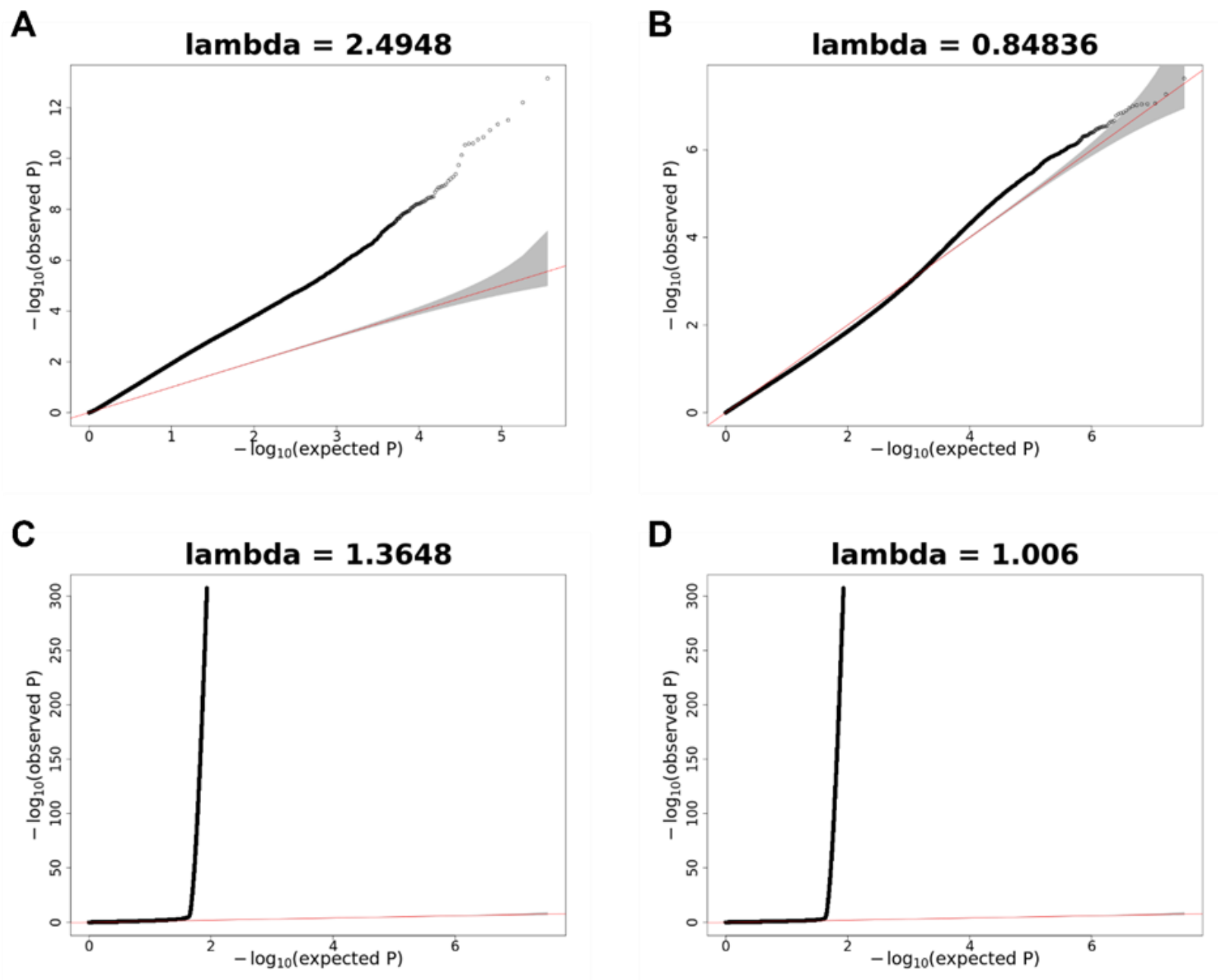

"Supplementary Figure 13 – quantile-quantile plots for quasibinomial generalized linear model tests

Quantile-quantile plots for -$\log_{10}$(p-values) from generalized-linear model (GLM) tests from SNP allele frequencies. Genomic inflation factor (lambda) is indicated at the top of each plot. Panels indicate A) using seed source as a fixed effect in a quasibinomial GLM, B) using the first two principal components from principal component analysis of SNPs from the poolseq data as a fixed effect in a quasibinomial GLM, C) Using seed source as a random effect in a binomial GLM, and D) Using seed source and individual pool identity as random effects in a binomial GLM."

R4.6

5. The strong signal on chromosome 15 is intriguing but poorly characterized. The failure to find the causative SV in the pangenome is noted, but no deeper investigation (e.g., targeted assembly of the region in key individuals) is attempted. The content of the region (mostly repetitive) offers no functional clues.

*We have more deeply characterised this region, in response to the reviewer's comments and those of Reviewer 1. We now believe signals from this region result from a potentially widespread but very rare, large or highly repetitive SV not captured in our pangenome. For full details see response to Reviewer 1's comment **R1.18**.*

R4.7

Specific Comments

6. The approach used for genome assembly (PacBio plus ONT) is sound, but the assembly could benefit from additional computational validation. The assembly metrics (N50, L90, BUSCO) are excellent. Consider a more rigorous assessment for assembly completeness.

*We have added additional metrics for completion - the LTR Assembly Index (LAI) indicated high assembly completeness, and assessing coverage of PacBio data mapped to the assembly did not indicate clear regions of high depth, indicative of collapsed regions. A kmer based estimate of genome size of 823-825.4Mb nevertheless indicated that there may be missing regions in the assembly - it is likely these are highly repetitive and difficult to assemble, potentially including centromere or ribosomal DNA sequence. These are discussed in more detail in response to a similar comment from Reviewer 1, **R1.2.***

R4.8

7. The high percentage of repeats (65.6%) is plausible for ash but could be commented on in relation to assembly completeness. Was this high repeat content well characterized, and does it hinder the identification of novel genes?

1. This high repeat content, coupled with high LTR completeness and a lack of plausibly duplicated regions identified from read mapping, suggests the repetitive content of the genome is well represented. The discrepancy between the kmer estimate of genome size and assembly size suggests some repeat content may remain uncharacterised, however.

2. We have added additional details on the repeat content to the results. Further detail is provided in Supplementary Figure 6 (see below).

Results: Lines 141-150

"65.6% of BATG-1.0 consists of repeats. LTR elements occupy 33.0% of the sequence, most of which comprises Ty1/Copia (15.3% of genome sequence) or Gypsy/DIRS1 (15.9% of genome sequence) families, with DNA transposons covering 6.2% of the sequence. Unclassified repeats covered 19.9% of the sequence. The total repetitive fraction is similar to the estimated repeat content of the assembly of another *F. excelsior* individual (69.12%) and assemblies for some other *Fraxinus* species such as *F. pennsylvanica* from the *Fraxinus* superpangenome[48], but is substantially higher than a separate estimate for *F. pennsylvanica* (48.4%[51])."

Supplementary Material: Lines 384-395

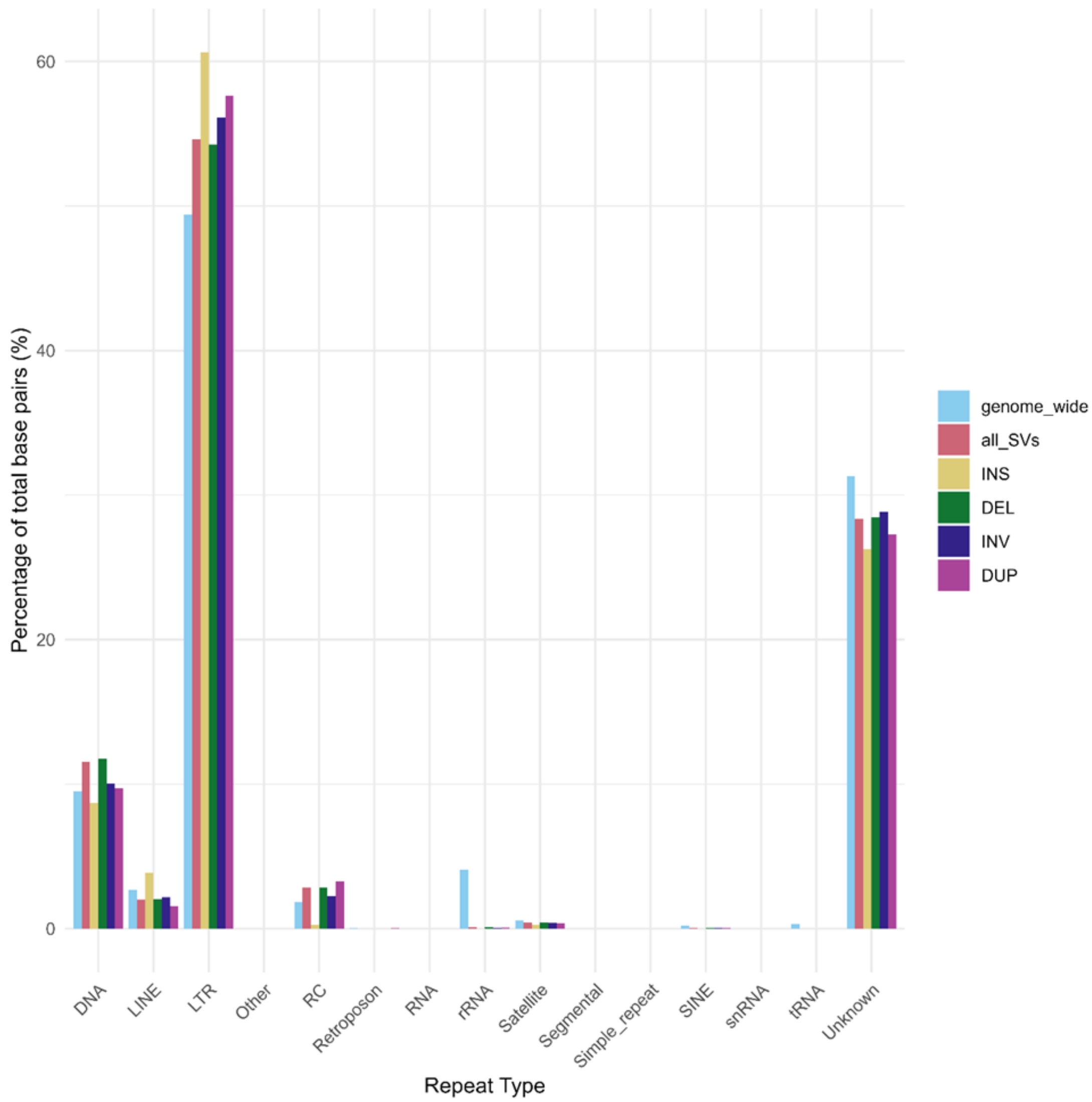

"Supplementary Figure 7 – Classes of repeats overlapping structural variants.

For i) the genome as a whole, ii) all classes of structural variants, iii) insertions (INS), iv) deletions (DEL), v) inversions (INV) and tandem duplications (DUP), the percentage of base pairs overlapping with various classes of repetitive element are plotted, grouped by repetitive element. These are i) DNA transposons, ii) long interspersed nuclear elements (LINEs), iii) miscellaneous classes of element ("Other"), iv) rolling-circles (RC), v) retrotransposons, vi) RNAs, vii) rRNAs, viii) Satellites, ix) segemental duplications, x) simple repeats, xi) short interspersed nuclear elements (SINEs), xii) tRNAs, and xiii) unclassified repeats ("Unknown"). "

3. It is possible that overmasking repetitive elements may hinder the identification of novel genes - we used a curated repeat library for F. excelsior, which should reduce this possibility, although extensive manual curation would be required to rescue gene sequences that have been masked. We have acknowledged this potential limitation in the text.

Discussion: Lines 451-454

"More comprehensive RNA-seq sampling from tissues/conditions across individuals, and manual curation of repetitive elements, may lead to more genes being annotated - including as dispensable - in future studies."

R4.9

8. The annotation protocol using multiple BRAKER3 runs is rigorous but complex. How were conflicts between the short-read and long-read-based annotations ultimately resolved?

*We are glad the reviewer appreciates the rigour of our annotation approach, although we do acknowledge it is complex. We should note the revised version of the manuscript contains some changes to the annotation pipeline, accounting for potential issues we noticed while re-running the analysis - these are outlined in response to reviewer 1's comment **R1.7**. These had minimal impact on the final results.*

The short-read and long-read datasets do identify different gene sets, but we believe these datasets are complementary rather than in conflict. The short-read data originates from a wider range of tissues/genotypes, whereas the long-read data can map less ambiguously, facilitating the annotation of genes with higher repetitive content. For both the BATG-1.0 annotation and pangenome annotation, long-read and short-read data were used separately for annotation, as the BRAKER3 software can currently only run either on short-read data, or long-read data, but not both simultaneously. We then combined these annotations, and genes annotated from this combined set (comprising genes annotated with short-read data only, annotated with long-read data only, and annotated by both) were included in downstream analyses.

<u>R4.10</u>

9. The number of annotated genes (38,276) is lower than in some other F. excelsior assemblies. Please discuss potential reasons (biological vs. methodological).

*We acknowledge that the number of genes we annotated in our linear reference is lower than for some other F. excelsior assemblies (as we also noted in the original version of our manuscript). We have added new RNA-seq data, and changed a step in the annotation pipeline that was potentially leading to similar genes from across BRAKER runs erroneously being categorised as different genes (see response to Reviewer 1's comment **<u>R1.7</u>**), leading to a slighter lower new total of 37,983 genes. We identified 1,471 genes absent from BATG-1.0, but present in other individuals in the pangenome - suggesting differences of up to this number are biologically possible, albeit unlikely. We removed genes with substantial overlaps of CDS (>50%) to avoid erroneously splitting genes, which other studies have not done; this could have reduced gene numbers in our annotation.*

It is possible that biological reasons could underpin differences between BATG-1.0 and BATG-0.5 (the latter of which has 38,948 genes), although it is likely methodological differences could also be important. This genome is highly fragmented, and could contain haplotigs with duplicated genes that may inflate gene content. Comparisons with more high quality recent genomes are likely to be more relevant, however.

The Fexc_ONT_UoY assembly contained 43,392 genes, and the F. excelsior genome from the Fraxinus superpangenome contains 50,327 genes. These differences are likely to be methodological. In both cases a different annotation pipeline is used (using BRAKER1 and BRAKER2 respectively), although it is difficult to say for sure why this led to the annotation of so many more genes than in our study. The annotation pipeline for the superpangenome F. excelsior assembly involved using homology "known Fraxinus proteins", the source of which was not detailed, so the potential impact of this is unknown. The repetitive element content of their F. excelsior genome is similar to, or lower than BATG-1.0 (50.15% for the superpangenome F. excelsior, and 69.12% for Fexc_ONT_UoY) - it seems unlikely that excessive masking in BATG-1.0 is responsible for the lower gene content. Indeed, less RNA-

seq data (and no long-read RNA-seq data) was used compared to the present study - a lack of RNA-seq evidence seems unlikely to explain the lower gene content in BATG-1.0 either.

Without re-running the pipelines on each of the individual genomes, it is difficult to confidently identify the exact reason for these differences. Running OrthoFinder with BATG-0.5, Fexc_ONT_UoY and other plant species identified 7.3% of Fexc_ONT_UoY genes failing to have an ortholog in a different assembly (F. pennsylvanica, Olea europaea, Solanum lycopersicum, Erythranthe guttata, Sesamum indicum and Salvia miltiorrhiza), compared to 1.1% and 0.6% of genes in BATG-1.0 respectively. The higher percentage of Orthogroups unique to the Fexc_ONT_UoY genes may suggest the number of genes in that study is overinflated. However, this is highly speculative, as the true number of unique F. excelsior genes that would not have orthologs outside the species is unknown.

Our assembly is phased, not scaffolded with F. pennsylvanica (unlike the two discussed above), annotated with a curated set of repeat elements for F. excelsior, a more comprehensive RNA-seq dataset, and with the latest version of BRAKER (BRAKER3). We therefore think it is likely our annotation is the most accurate. We have added some brief discussion of this to the text.

Results: Lines 167-171
"These latter two *F. excelsior* assemblies have more genes annotated than are plausibly missing from BATG-1.0 due to being dispensable (see pangenome annotation below), suggesting differences are likely due to different annotation approaches rather than intraspecific variation in gene content."

R4.11
10. The pangenome approach is appropriate for SV discovery. The use of Sniffles and cuteSV provides a solid framework for SV calling, but did these methods capture all variations? Was there an assessment for any missed SVs?

We are glad the reviewer believes the SV discovery framework is appropriate. The recall of heterozygous SVs using the SV discovery method chosen in the individual used to produce BATG-1.0 was 84.4%. We therefore think it very likely that SVs were missed from our samples, as well as in the wider F. excelsior population.

R4.12

11. The saturation curve (Fig. 2B) clearly shows that SV discovery has not plateaued, suggesting more diversity remains to be captured.

We agree - this is referred to in the main text (see below).

As reviewer 2 notes (see comment **R2.8***), this pattern is also consistent with each individual having unique false positives due to assembly errors/read mismapping. We have highlighted this point in the main text.*

Results: Lines 209-212

"Merging samples sequentially resulted in fewer new SVs discovered in the fiftieth sample compared with the first, but the number newly detected did not plateau, indicating that more samples would be required to exhaustively sample SVs in *F. excelsior* (Figure 2B). It is also possible that sample-specific SVs represent sample-specific assembly or mapping errors"

R4.13

12. Why were SVs called in fewer than 3 individuals excluded? While this reduces false positives, it may eliminate rare, functionally important variants.

As the reviewer suggests, removing the rarest structural variants is likely to eliminate false positives, such as those caused by assembly/mapping errors in a small number of individuals, albeit with a cost of removing rare, potentially functionally important variants. It should be noted that the number of samples required is >3, not >=3 as referred to in the original manuscript (we have corrected this point in the revised version).

*Our decision to apply a frequency-based threshold was motivated by 1) our examination of the precision/recall estimates of the SV calling - the best method, (that with the closest agreement to calling SVs in the reference individual using the chromosome-scale haploid assembly) whilst capturing a large number of SVs, achieved a precision of 78% (Extended Data Figure 1), so likely includes a number of putative false positives (i.e. those called by the read mapping + de novo assembly based approach we used, but not by mapping the high quality haplotype 2 haploid assembly to BATG-1.0). We also noted that 2) SV discovery failed to plateau with the 50th sample (Figure 2A) - this is consistent with high genetic diversity in tree species such as ash, but as reviewer 2 noted (***R2.8***) this could also be consistent with sample-specific assembly or mapping errors. These factors led us to implement a frequency-based cutoff.*

Without a pre-existing high-confidence truth set of SVs, it is difficult to assess the frequency of false positive calls, and so to justify a particular frequency as a cutoff - we felt a minimum

number of 4 individuals would provide a high level of support, and still allow many SVs to be included. We recognise this may strike some as overly conservative - a vcf of all putative SVs (i.e. without a frequency cutoff) will be made available on Zenodo, alongside the vcf of SVs used for the pangenome in this study, so downstream users can apply a different frequency threshold if desired.

It should be noted that despite our potentially conservative filtering, we still detect a larger number of SVs than many recent plant pangenome studies, potentially partially due to our study including a relatively large number of samples (Extended Data Figure 9A). Including SVs in more than 8% of our 50 individuals, we can detect SVs at lower frequencies than studies including less than 12 individuals (~38% of the recent plant pangenome studies we compared our study with in the manuscript, see Supplementary Table 7) with no frequency cutoffs. Most pangenome studies include SVs present in single individuals - whilst we suspect this may lead to many false positives, we acknowledge further study is needed to demonstrate this.

We discuss the precision of SV calling in the text already, but we have further acknowledged the points raised above in the text.

Results: Lines 212-215

"It is also possible that sample-specific SVs represent sample-specific assembly or mapping errors - to attempt to control for this, only SVs present in more than three individuals were retained for further analysis (Figure 2A), although this may result in rare SVs being missed."

R4.14

13. It is noted that 69% of SVs overlap with repeats. Was there any analysis of the type of repeats (LTR, LINE, etc.) associated with SVs?

*We have added analysis of repeats associated with SVs - overall the percentage of base pairs overlapping with SVs closely reflects the repeat content of the genome as a whole (plotted in Supplementary Figure 7, displayed in our response to the reviewer's comment **R4.8** above). The exception to this was rRNAs and tRNAs, where their overlaps with SVs made up a smaller proportion of the total overlaps than might be expected from the sequence they occupy in the genome. It seems plausible that SVs disrupting these core components of the cell machinery are less likely to be segregating in populations compared to other classes of repeat. We have added text discussing this.*

Results: Lines 222-226
"69% of SVs overlapped with an annotated repeat in the reference genome; the distribution of base pairs overlapping different SV types closely reflected the makeup of the genome as a whole (i.e. mostly LTRs and DNA transposons), although rRNAs and tRNAs were relatively unlikely to overlap segregating SVs, likely due to their core role in the cell machinery (Supplementary Figure 7)."

R4.15

14. The distribution of SVs across chromosomes (e.g., centromeric vs. telomeric regions) is not shown or discussed.

We have added a circos plot (Figure 2D) and per-chromosome plots (Supplementary File 1) displaying the distribution of SVs across chromosomes in more detail - see response to a similar point raised by reviewer 1, **R1.13.**

R4.16
15. The number of dispensable genes (3,065) identified is notable, but the reasoning for calling a gene dispensable should be carefully assessed. What criteria were used to determine if a gene is dispensable, and how reliable is this categorization?

*1. We define a gene as dispensable in the Abstract (Line 32) as "*dispensable genes (those that vary in their presence/absence between individuals)". *The criteria used to define genes as dispensable is outlined in the methods section:*

Methods - Lines 832-852
"Novel genes not present in the BATG-1.0 genome should overlap with an SV, as should deletions of genes present in the reference genome. For each novel gene that overlapped a SV at least once (i.e. in at least one sample, including the reference individual sequenced with long reads), an F1 score was calculated to identify the strength of the association between the new gene and the SV: 2 × N_Gene_SV / (2 × N_Gene_SV + N_Gene_NSV + N_NGene_SV) where N_Gene_SV is the number of samples with the gene and the SV, N_Gene_NSV is the number of samples with the gene but without the SV, and N_NGene_SV is the number of samples without the gene but with the SV. For novel genes overlapping more than one SV, the SV with the highest F1 score was retained as potentially causal for the novel gene. An equivalent calculation was performed for genes present in the BATG-1.0 genome but absent in at least one sample, where an SV overlapped with the annotation coordinates for the gene in the reference. To account for genes potentially being associated with multiple SVs, we also calculated an F1 score of gene presence/absence with all overlapping SVs - if this scored higher than any individual SV, this score was taken forward. Genes with an F1 score of 0.8 or above were retained in the final pangenome annotation as being dispensable; for those with an

F1 score of < 0.8, those absent in the BATG-1.0 genome were removed, and those present in the BATG-1.0 genome were classified as invariant. Genes that did not overlap an SV in any sample were classified as invariant if they were annotated in BATG-1.0 and were absent in some other samples, and discarded if they were absent in BATG-1.0 but present in some other samples."

2. The reliability of this categorisation approach is difficult to ascertain without a high-confidence truth set. Ultimately comprehensive proteomics of the F. excelsior individuals included in the pangenome would be required for validation of the gene sets identified, but this is beyond the scope of the current paper. However, we would argue that a necessary, if not sufficient, criterion for a gene being dispensable is a consistent sequence difference between individuals putatively possessing a gene, and those putatively lacking it. We show that the standard approach of annotating each individual genome and identifying differences will in many cases not pass even this criterion. We are therefore confident that our categorisation is more reliable than simply annotating individual assemblies, as occurs in most other pangenome studies.

R4.17

16. The drastic reduction in dispensable genes (from 34% to 7.8%) after filtering is a crucial result that should be emphasized more.

We have added this point to the abstract, and have added some extra emphasis in the discussion (although reduction is now from 35.9% to 8.7%).

Abstract: Lines 30-34

"We demonstrated that failing to explicitly link SVs with gene sequences can lead to substantial overestimation of dispensable genes (those that vary in their presence/absence between individuals) due to variability in the annotation process. Controlling for this reduced the fraction of the genome estimated as dispensable from 35.9% to 8.7%"

Discussion: Lines 439-441

"Despite leading to a dramatic reduction in dispensable genes, we believe this filter is justified as variability in the annotation process has previously been found to underlie putatively dispensable gene sets[92,95]"

R4.18

17. How were genes that overlapped with multiple SVs handled? Was the longest SV or the one with the highest F1 score chosen?

The SV with the highest F1 score was chosen as the one most likely to be causatively associated with the gene. Note that in the revised manuscript we also calculate an F1 score for all SVs, not just each individual SV - if the SVs in aggregate have a higher F1 score than any one individual SV, this was chosen as the value reported for that gene (see response to reviewer 1's comment ***R1.7****).*

Methods - Lines 840-846

"For novel genes overlapping more than one SV, the SV with the highest F1 score was retained as potentially causal for the novel gene. An equivalent calculation was performed for genes present in the BATG-1.0 genome but absent in at least one sample, where an SV overlapped with the annotation coordinates for the gene in the reference. To account for genes potentially being associated with multiple SVs, we also calculated an F1 score of gene presence/absence with all overlapping SVs - if this scored higher than any individual SV, this score was taken forward."

R4.19

18. The GO term analysis is somewhat weak. The lack of clear enrichment in the high-confidence dispensable gene set is disappointing but perhaps honest. This could be discussed more.

Other studies, with potentially inflated numbers of dispensable genes, have identified significantly enriched GO terms, suggesting differences in the classes of genes likely to be dispensable vs. indispensable. Our results disagree somewhat with these conclusions - based on what we believe is more appropriate filtering, there is not clear evidence for functional differentiation between dispensable and non-dispensable genes in our study. Rather than being disappointing, we think this is a significant insight. Nevertheless, there is not substantial functional differentiation between putatively dispensable genes we filtered out, and high confidence dispensable genes we included (see Figure 4A, 4B). This suggests that more stringent filtering for dispensable genes is unlikely to result in different sets of GO terms being enriched, although smaller numbers of genes overall make passing a significance threshold unlikely. We have further emphasised this point in the text.

Discussion: Lines 448-451

"It should however be stressed that there was no clear functional distinction between the final set of dispensable genes and those removed during filtering - a smaller set of genes will inherently have lower power to reliably detect enrichment in GO terms."

R4.20

19. For the 133 defense-related dispensable genes, a more detailed analysis is needed (e.g., what protein families do they belong to? Are any NBS-LRR genes?).

We have further characterised these genes – more details of functional annotations for those annotated with defense-related GO terms is outlined in Supplementary Table 5 (available as a separate Excel spreadsheet; see caption below); we note that in the revised version there are 141 defence-related genes, compared with 133 in the original manuscript. The genes belonged to a wide range of protein families, with the most frequently annotated (8) indeed belonging to the NBS-LRR superfamily. Further discussion of this has been added to the text:

Results - Lines 276-280

"Dispensable genes with a "defense response" GO term belonged to a variety of protein superfamilies (Supplementary Table 5), including cytochrome P450s (three genes) and flagellin-sensing 2 (FLS2)-related serine/threonine protein kinases (two), with Nucleotide Binding Site, Leucine Rich Repeat (NBS-LLR) superfamily being the most frequently annotated (eight genes)."

Supplementary Information - Lines 648-662

"Supplementary Table 5 – Functional annotation of key subsets of genes

Spreadsheet of functional annotation of key subsets of genes, each subset in a different tab as follows – i) high-confidence dispensable genes (an SV-Gene F1 association score > 0.8; see Results; tab 5.1) annotated with a "defense response" GO term or a child term of this GO term (GO:0006952), ii) genes that contained one or more of the 220 SNPs associated with low ADB susceptibility (tab 5.2), genes within 10kb of the 220 SNPs associated with low ADB susceptibility (tab 5.3), and the subset of these genes annotated with a "defense response" GO term, as defined above (tab 5.4). Columns indicate i) gene name, ii) the bitscore and iv) p-value of the top hit in the UniProt/SwissProt database for each gene, v) the bitscore and vi) p-value of the top hit in the NCBI NR database for each gene, vi) the PANTHER superfamily classification code and vii) description for each gene, vii) the InterPro family classification code and viii) description for each gene, ix) the bitscore and x) p-value of the best hit from a DIAMOND blastp search of the genes against the Araport 11 representative gene peptides, and xi) the name of the best hit."

R4.21

20. The study identifies 362,965 SVs, a large number. What proportion of these are actually relevant for disease resistance versus other ecological traits?

Based on our GWAS for association with low ADB susceptibility, we identified 531 SVs as passing a significance threshold of 1 x 10^{-13}, but we believe many of these may be false positives. Even if all were true positives, this would indicate <1% of the total number of SVs are associated with low ADB susceptibility, at least as evidenced by the poolseq dataset we reanalysed. Future studies may reveal more of the SVs identified in the pangenome as having a role in resistance. Other SVs may well be involved in ecological traits - by providing a resource for genotyping SVs, future association studies in F. excelsior may provide a greater insight into this question than our study is able to. We have further emphasised this point our concluding paragraph.

Discussion: Lines 530-539

"Overall, our newly developed pangenome captures a diverse set of SVs from across the geographic range of European ash, including a large quantity of previously uncharacterised sequence and dispensable genes, making it an essential resource for full investigation of the role of both SNPs and SVs in a breadth of ecological traits for this important tree species."

R4.22

21. The study links SNPs and SVs with low ADB susceptibility. However, GWAS results for low susceptibility to ADB are somewhat inconclusive. Does the paper provide sufficient justification for the statistical approaches used (e.g., CMH test, qGLM)?

The CMH test is the most commonly used GWAS test used for association in poolseq datasets, and particularly was previously used to analysis the Stocks et al. (2019) dataset. We note the potential statistical issues with this commonly used test that we believe should be more widely known (see lines 358-359; 511-529). The key study highlighting these issues, Wiberg et al. *(2017), found the qGLM test was most able to identify highly consistent allele frequency shifts across all pairs of pools, which we felt justified also using this test. Following the reviewer's earlier comment 5 (**R4.6**) and comments from other reviewers, we more deeply interrogated the results of the GWAS. Following this (see revised Results, Discussion), we identify a set of 220 SNPs that we more conclusively identify as behaving in a manner consistent with being associated with low ADB susceptibility (see Results, Discussion).*

R4.23

22. The quasibinomial GLM test was a good additional analysis to try, and its negative result is important to include.

We appreciate the reviewer's comment.

R4.24

23. The authors claim to identify loci associated with low disease susceptibility. Were these loci replicated across different European populations, or are they specific to the UK or specific provenances?

In our revised methods, we identified a set of 220 SNPs that shift in frequency in a broadly consistent manner between healthy and unhealthy pools. Given the failure of F_{st} and the Fisher test to identify high confidence outliers (see revised Supplementary Note 3), we are now more cautious about drawing high confidence inferences between subsets of pools within our study. We did not test whether these SNPs are segregating across different European populations - as new high quality European sequencing datasets become available, this will be an interesting area for further research.

R4.25

24. The GWAS analysis identifies many significant SNPs, but the authors acknowledge potential false positives. What measures are in place to reduce these false positives, and how can future studies improve the reliability of GWAS findings in this context?

We have substantially changed our interpretation of the outputs of the CMH test - by considering SNPs that have significant p-values after Bonferroni correction, and removing SNPs that i) violate the tests assumptions of homogeneity of odds ratios, and ii) those with high standard deviations of depth, indicative of potential uncharacterised SVs leading to erroneous allele frequency calls, we identified 220 SNPs that are likely to avoid key errors potentially leading to false positives in the CMH test (see revised Results). We hope that future studies would perform similar corrections to avoid drawing erroneous conclusions from the CMH test, as we suspect may have occurred for the smallest p-values in Stocks et al. 2019 (see revised Discussion), and likely many other studies.

R4.26

25. Patterns of linkage decay between SNPs, SVs, and their potential implications for disease resistance are briefly mentioned. Can the authors provide further exploration of how this could guide future breeding programs?

Linkage decayed quickly across the samples considered for all SNPs and SVs (Extended Data Figure 5C), although we should emphasise that the results here are based on a small number of samples, from an unknown number of parent individuals, so we would hesitate to draw wider biological conclusions from this. The linkage analysis presented here was mainly intended to check that SVs behaved in a broadly similar way to SNPs - as there are issues in SV allele frequency estimation from poolseq, we wanted to check that individual based SV calls did not have unusual linkage patterns compared to SNPs, which could have indicated problems with genotyping. Overall patterns were similar between the different variant types. Generally low patterns of linkage disequilibrium in trees may make it more difficult to identify causative genetic markers, as the number of linked sites may be low - although it may mean that identified sites may be closer to causative loci.

We are not entirely sure whether the above covers the point the reviewer wishes us to explore further; we would be happy to add to our response if more clarification were provided.

R4.27

26. While SV genotyping from poolseq data is promising, the authors mention discrepancies. A deeper discussion of these discrepancies and their impact on the overall results is required.

We were unable to ascertain the exact reason for a small number of SVs showing discrepancies between read coverage in the in silico pool, and individual samples, nor identify particular properties predicting SVs showing a discrepancy. Discussion of this issue with the developers of the vg pipeline ended inconclusively (see https://github.com/vgteam/vg/issues/4443) - identification of the issue within their highly specialised software pipeline, and therefore further discussion, is unfortunately beyond our expertise. We further highlighted this issue in the text of our revised manuscript (see below).

In terms of impact on overall results, we concluded that, based on this discrepancy, the SV genotypes from poolseq data are insufficiently accurate for a high degree of confidence in individual SV calls. This does seem to only affect a minority of SV calls - a PCA based on SVs is broadly similar to that reconstructed from SNPs (Extended Data Figure 6C, D). This suggests that reliance on individual poolseq calling is inadvisable, but that overall patterns of diversity in SVs may broadly be inferred using these methods - although obviously development of more accurate methods would be desirable.

Discussion: Lines 470-481

"SV genotyping from short-reads is generally more challenging than SNPs[100–102], and we are not aware of previous studies calling allele frequencies of poolseq data mapped to a pangenome graph. Our results indicate poolseq data can be used to estimate allele frequencies accurately for most SVs (Extended Data Figure 6), but the approach used generates more discrepancies with individual based genotyping than is the case for SNPs. SVs showing these discrepancies were particularly likely to be identified as significant in a GWAS using a CMH test, resulting in many likely false positives. We were unable to identify the cause of these discrepancies (see https://github.com/vgteam/vg/issues/4443). We should emphasise that vg is not designed for analysing poolseq data; the development of dedicated software for estimating SV allele frequencies from poolseq data, equivalent to those available for SNPs, is required in order to make full use of poolseq data in the pangenome era."

R4.28

27. The authors suggest combining variants from different genetic backgrounds for improved resistance. Could this lead to increased heterosis or other unintended consequences? More exploration of the ecological impacts would strengthen the study.

Based on our revised results, we now do not believe this dataset allows accurate assessment of low ADB susceptibility in subsets of the pools analysed. We have therefore removed the text the reviewer refers to from the discussion.

R4.29

28. The suggestion that crossing populations could improve resistance is logical but remains speculative without empirical data.

As with the point above, based on our new results we have removed the discussion the reviewer refers to from the text.

R4.30

29. The inconsistency in the NSZ-107 replicate is concerning and should be discussed as a potential issue with sample pooling or phenotyping.

*We believe the inconsistency in the NSZ-107 replicate for the previously identified region on chromosome 15 is now explained by the confounding presence of a very rare SV - see response to reviewer 1's comment **R1.18**.*

R4.31

30. The failure to find the insertion in the pangenome highlights a limitation of the current sampling strategy.

*We now believe this insertion is much larger, and much rarer, than we had initially thought - occurring only in 2/692 samples [albeit from a single seed source from Stocks et al. (2019), and a single woodland for the samples from Metheringham et al. (2025) - see response to reviewer 1's comment **R1.18** for complete discussion], although it is potentially widely distributed if it is indeed present across multiple provenances sampled in Stocks et al. This does represent a limitation of the current sampling strategy, although capturing such rare, large SVs would be much more challenging than our initial assessment would have suggested.*

Once again, we thank all reviewers for the time taken to review this manuscript, and their constructive suggestions.

References

Audet, Tyler, Joelle Krol, Katie Pelletier, Andrew D. Stewart, and Ian Dworkin. 2024. "Sexually Discordant Selection Is Associated with Trait-Specific Morphological Changes and a Complex Genomic Response." *Evolution; International Journal of Organic Evolution* 78 (8): 1426–1440.

Brennan, Reid S., James A. deMayo, Hans G. Dam, et al. 2022. "Experimental Evolution Reveals the Synergistic Genomic Mechanisms of Adaptation to Ocean Warming and Acidification in a Marine Copepod." *Proceedings of the National Academy of Sciences of the United States of America* 119 (38): e2201521119.

Chano, Víctor, Renata Callegari Ferrari, Tania Domínguez-Flores, et al. 2025. "Transcriptional Time-Course Analysis during Ash Dieback Infection Revealed Different Responses in Tolerant and Susceptible Fraxinus Excelsior Genotypes." *BMC Plant Biology* 25 (1): 107.

Christen, Peter, David J. Hand, and Nishadi Kirielle. 2024. "A Review of the F-Measure: Its

History, Properties, Criticism, and Alternatives.” *ACM Computing Surveys* 56 (3): 1–24.

Clark, Jo. 2024. “Clark 2024. The Living Ash Project - 10 Years on.” *ResearchGate*, November 11. http://dx.doi.org/.

Cochran, William G. 1954a. “Some Methods for Strengthening the Common χ 2 Tests.” *Biometrics* 10 (4): 417.

Cochran, William G. 1954b. “The Combination of Estimates from Different Experiments.” *Biometrics* 10 (1): 101.

Delahaye, Clara, and Jacques Nicolas. 2021. “Sequencing DNA with Nanopores: Troubles and Biases.” *PloS One* 16 (10): e0257521.

Dobin, Alexander, Carrie A. Davis, Felix Schlesinger, et al. 2013. “STAR: Ultrafast Universal RNA-Seq Aligner.” *Bioinformatics* 29 (1): 15–21.

Doonan, James M., Chatchai Kosawang, Michael Eisenring, et al. 2023. “Transcriptome Profiling of Fraxinus Excelsior Genotypes Infested by Emerald Ash Borer.” *Scientific Data* 10 (1): 680.

Espinosa, Elena, Rocio Bautista, Rafael Larrosa, and Oscar Plata. 2024. “Advancements in Long-Read Genome Sequencing Technologies and Algorithms.” *Genomics* 116 (3): 110842.

Franco Ortega, Sara, James A. Bedford, Sally R. James, et al. 2025. “*Fraxinus Excelsior* updated Long-Read Genome Reveals the Importance of MADS-Box Genes in Tolerance Mechanisms against Ash Dieback.” *G3 (Bethesda, Md.)* 15 (5): 2024.12.20.629733.

Gazda, Małgorzata A., Pedro M. Araújo, Ricardo J. Lopes, et al. 2020. “A Genetic Mechanism for Sexual Dichromatism in Birds.” *Science (New York, N.Y.)* 368 (6496): 1270–1274.

Higgins, Julian P. T., Simon G. Thompson, Jonathan J. Deeks, and Douglas G. Altman. 2003. “Measuring Inconsistency in Meta-Analyses.” *BMJ (Clinical Research Ed.)* 327 (7414): 557–560.

Kofler, Robert, Ram Vinay Pandey, and Christian Schlötterer. 2011. “PoPoolation2: Identifying Differentiation between Populations Using Sequencing of Pooled DNA Samples (Pool-Seq).” *Bioinformatics* 27 (24): 3435–3436.

Kossowsky, Joe, Monique C. Pfaltz, Silvia Schneider, Jan Taeymans, Cosima Locher, and Jens Gaab. 2013. “The Separation Anxiety Hypothesis of Panic Disorder Revisited: A Meta-Analysis.” *The American Journal of Psychiatry* 170 (7): 768–781.

Liu, Jian Ning, Liping Yan, Zejia Chai, et al. 2024. “Pan-Genome Analyses of 11 Fraxinus Species Provide Insights into Salt Adaptation in Ash Trees.” *Plant Communications* 0 (101137): 101137.

Metheringham, Carey L., William J. Plumb, William R. M. Flynn, et al. 2025. “Rapid Polygenic Adaptation in a Wild Population of Ash Trees under a Novel Fungal Epidemic.” *Science (New York, N.Y.)* 388 (6754): 1422–1425.

Micheletti, Steven J., and Shawn R. Narum. 2018. “Utility of Pooled Sequencing for Association

Mapping in Nonmodel Organisms." *Molecular Ecology Resources* 18 (4): 825–837.

Schertler, Elisabeth, Valentin Queloz, Federico Monteleone, Michael Eisenring, and Renate Heinzelmann. 2025. "Resistance and Tolerance Mechanisms of Common Ash (*Fraxinus Excelsior*) against the Ash Dieback Pathogen *Hymenoscyphus Fraxineus*: Latest Advances and Knowledge Gaps." *Forestry (London, England)*, June 23, cpaf038.

Simão, Felipe A., Robert M. Waterhouse, Panagiotis Ioannidis, Evgenia V. Kriventseva, and Evgeny M. Zdobnov. 2015. "BUSCO: Assessing Genome Assembly and Annotation Completeness with Single-Copy Orthologs." *Bioinformatics (Oxford, England)* 31 (19): 3210–3212.

Sollars, Elizabeth S. A., Andrea L. Harper, Laura J. Kelly, et al. 2017. "Genome Sequence and Genetic Diversity of European Ash Trees." *Nature* 541 (7636): 212–216.

Stocks, Jonathan J., Richard J. A. Buggs, and Steve J. Lee. 2017. "A First Assessment of Fraxinus Excelsior (common Ash) Susceptibility to Hymenoscyphus Fraxineus (ash Dieback) throughout the British Isles." *Scientific Reports* 7 (1): 16546.

Stocks, Jonathan J., Carey L. Metheringham, William J. Plumb, et al. 2019. "Genomic Basis of European Ash Tree Resistance to Ash Dieback Fungus." *Nature Ecology & Evolution* 3 (12): 1686–1696.

Wiberg, R. Axel W., Oscar E. Gaggiotti, Michael B. Morrissey, and Michael G. Ritchie. 2017. "Identifying Consistent Allele Frequency Differences in Studies of Stratified Populations." *Methods in Ecology and Evolution / British Ecological Society* 8 (12): 1899–1909.

Woolf, B. 1955. "On Estimating the Relation between Blood Group and Disease." *Annals of Human Genetics* 19 (4): 251–253.

Yengo, Loïc, Sailaja Vedantam, Eirini Marouli, et al. 2022. "A Saturated Map of Common Genetic Variants Associated with Human Height." *Nature* 610 (7933): 704–712.

REVIEWER COMMENTS

Reviewer #1 (Remarks to the Author):

R1.1

The authors addressed all of my comments. I don't have further comments.

We are glad the reviewer is satisfied that their comments were addressed, and we thank them again for their review.

Reviewer #2 (Remarks to the Author):

R2.1

The authors did an impressive job in addressing the reviewers comments. We find the answers to our comments and corresponding modifications of their work are overall well done. Among others, they generated quite a lot of new RNA-Seq data for improving the gene models annotation; we believe these changes indeed strengthen the reliability of results and conclusions of the manuscript. The authors also have altered their interpretation of the data in respect to provenance-specific architectures and the limitations of their poolseq data set; they also provide suggestions as to why SNPs previously reported to be associated to ADB tolerance were not detected as candidates in this study. Overall we believe that the current version of the manuscript is fit for publication and provides an important addition in the search for the molecular basis of ADB tolerance.

We are glad reviewers 2 and 3 feel the comments from the previous round of revisions were addressed sufficiently, and are pleased to note that they believe the manuscript is fit for publication and provides an important contribution. We thank them again for their review.

Reviewer #3 (Remarks to the Author):

R3.1

I co-reviewed this manuscript with one of the reviewers who provided the listed reports. This is part of the Nature Communications initiative to facilitate training in peer review and to provide appropriate recognition for Early Career Researchers who co-review manuscripts.

We thank reviewer 3 for their contribution.

Reviewer #4 (Remarks to the Author):

R4.1

I would like to acknowledge the substantial effort the authors have invested in revising this manuscript. The revised version shows clear improvements in several important respects, including more rigorous analyses, additional validation steps, and a more cautious and balanced interpretation of previously highlighted results. These revisions have meaningfully enhanced the technical robustness and transparency of the study. Moreover, the genomic resources generated here, particularly the chromosome-scale assembly and the pangenome dataset, represent a valuable contribution to the field and will likely be of broad interest to the tree genomics and plant-pathogen interaction communities.

We are glad the reviewer recognises the significant effort we went to in revising the manuscript, and that they believe it represents a clear improvement on the previous version, is a valuable contribution to the field, and will be of broad interest.

However, despite these improvements, I remain concerned that the manuscript, in its current form, does not yet provide the level of biological insight or conceptual advance for publication in Nature Communications. In particular, the central objective of establishing a clear link between genomic variation and ash dieback susceptibility remain only partially achieved.

Our central objectives were to construct and characterise the pangenome of Fraxinus excelsior, and use this to identify genetic variants consistently associated with low ash dieback susceptibility. We argue both of these have been achieved (along with a number of methodological insights), and together represent an important biological insight and conceptual advance; we expand on this in response to comment R4.5.

Based on the reviewer's previous comments and those below, we assume their concept of "establishing a clear link between genomic variation and ash dieback susceptibility" involves characterising the mechanistic basis by which these variants are associated with low susceptibility. This was not the objective of this study - we expand more fully on why in response to comment R4.2 below.

R4.2

1. While the study is technically strong and the authors have taken commendable steps to ensure analytical rigor, there remains a notable gap between the quality of the genomic resource and the strength of the biological conclusions. The main findings rely largely on correlative analyses, and although the authors appropriately acknowledge the associated

limitations, the manuscript does not yet provide sufficiently strong evidence to support a clear biological or mechanistic interpretation of disease susceptibility.

We are glad the reviewer appreciates the technical strength of the study, and the quality of the genomic resource generated.

This study did not aim to identify a mechanistic interpretation of disease susceptibility (i.e. the genes, biochemical or developmental pathways, physiological mechanisms etc. that cause low susceptibility - we are unclear whether "biological interpretation" refers to this or something else). Instead we primarily aim to identify genetic variants consistently associated with low ash dieback susceptibility. We assume a non-correlative analysis would involve active manipulation of components in the system to confirm function (e.g. gene knockouts). As we outlined in our response to comment R4.1 in the first round of reviews, we believe working to identify the mechanistic basis of low ash dieback susceptibility is not a high priority area in the context of limited research time and funding. Our rationale is as follows:

Practicality: *The effect size of each individual marker in highly polygenic traits is likely to be small, meaning non-correlative validation of individual markers using knockouts or equivalent would i) consume large amounts of resources (requiring large samples sizes to identify small effects) and ii) be of limited utility (each explaining only a small amount of trait variation). Such an undertaking would be made even more difficult by the nature of the ash dieback disease system, as i) trees are difficult to manipulate experimentally/genetically transform [1], and require multiple years and large amounts of space to grow, ii) the disease is specific to Fraxinus (or its very close relatives), making recapitulation in a model organism implausible, and iii) the disease symptoms themselves are multifaceted, take years to fully develop and are challenging to characterise accurately on an individual basis (therefore requiring an even larger number of samples). Functionally validating how particular genes/pathways produce low ash dieback susceptibility would therefore require a great deal of resources to provide even a partial picture.*

A clear interpretation: *Even if extensive functional characterisation was performed of the candidate genes identified, we are not convinced a "clear…mechanistic interpretation of disease susceptibility" would emerge. Small changes in many pathways are likely involved, from overall tree health, specific phenological patterns, to specific resistance mechanisms at the tissue or cellular level [2]. As the reviewer suggested in comment R4.4, we have further functionally analysed our candidate genes and integrated them with other studies, supporting a diverse set of mechanisms potentially underpinning low susceptibility (see response to R4.4 for details).*

Limited impact: *Even if, following functional validation, the mechanistic basis of low susceptibility was clearly identified, the practical impacts of this knowledge would be limited. Studies of disease susceptibility in humans may place additional emphasis on understanding the mechanistic basis of disease susceptibility as they will be looking for therapeutic targets - this is not a focus of our work; a treatment for ash dieback would be impractical to administer to entire forests. Even if a mechanism was identified, heritable variation in the action of this mechanism would still need to be identified. Identification of genetic variants associated with*

variation in overall disease susceptibility, as we have done here, would therefore still need to be performed to understand the potential for natural selection, or a breeding program, to lead to lower susceptibility to the disease. Using genetic variants to guide breeding programs, without knowledge of how they affect traits mechanistically, has been successful in crop and tree breeding programs - indeed, the genomic prediction approach does not even rely on the effect size of the individual markers being highly accurate [3–6].

We therefore emphasise our earlier point - that understanding the mechanism of disease resistance is not the only, or the most high impact, approach for ensuring the continued survival of keystone forest tree species. We believe the identification of genetic variants associated with low ash dieback susceptibility, which can be directly used to inform the first generation of a breeding program, is substantially more impactful.

R4.3

2. The revised manuscript convincingly highlights important limitations in estimating SV allele frequencies from pool-seq data, which in itself constitutes a valuable methodological contribution. However, this also substantially weaken the SV-based association analyses as a foundation for biological inference. Consequently, the role of structural variation in the trait of interest remains unclear. The manuscript therefore appears to have moved away from what initially a central component of the study, without fully established a coherent alternative framework.

We are glad the reviewer agrees that highlighting the limitations of estimating SV frequencies from poolseq data is a valuable contribution.

As detailed in the original manuscript submitted, and outlined in our response to a similar point the reviewer made in comment R4.3 from their original review, SV calls from poolseq data were not considered sufficiently reliable to perform a GWAS. This was therefore not a central component of the study in the original manuscript, and this section did not change between the original manuscript and the revised manuscript. We are therefore unsure why the reviewer believes we have moved away from an initially "central component of the study". We specified the need for the development of software specifically designed to detect structural variants from poolseq data mapped to pangenomes, which is beyond our expertise to create. We therefore instead focus on analysing SNP allele frequencies from the poolseq data, which are more reliable as the reviewer acknowledges below. This resulted in the identification of genetic variants consistently associated with low ash dieback susceptibility, which was among the primary aims of this study.

R4.4

3. The SNP-based analyses appear more robust, and the identification of variants showing consistent allele frequency shifts is encouraging. Nevertheless, these results remain challenging to interpret biological context. While the findings suggest a highly polygenic architecture, the study does not yet identify clearly interpretable loci, pathways, or mechanisms that would advance understanding of ash dieback resistance. Further development of this aspect, such as deeper functional annotation, integration with prior studies, or additional validation, would be required to enhance the impact of these findings.

We are glad the reviewer believes the revised SNP-based analyses to be robust and encouraging. We would argue that identifying sets of SNPs that consistently shift across replicates, that has not been explicitly identified in previous studies, represents an important advance in our understanding of low susceptibility to ash dieback. We are primarily interested in understanding the genetic component of low ash dieback susceptibility - for which identification of variants consistently associated with the trait is the most important aspect.

However, we do agree that deeper integration with prior studies would support the validity of these results, and would enhance the impact of the work. To this end, as recommended by the reviewer, we have integrated our results with several previous studies that focus on genes with potential roles in low ash dieback susceptibility.

We would note this was made more challenging by the fact that many of these studies used an unpublished annotation that is not publicly available for the FRAX_001_PL genome assembly of Fraxinus excelsior (which is publicly available, but also is not formally described in a publication) - identifying homologs of these genes was performed using reciprocal best BLAST and/or partial information provided in the publications, detailed in our revised methods (see below). Notably, Doonan et al. (2025)[7] identify a series of genes near to SNPs that are the most predictive of ash dieback phenotypes, but do not provide a list of these genes, so beyond those named in the text of that study, these results could not be included in our new analysis.

Nevertheless, we integrated our results with genes i) differentially expressed in response to ash dieback infection in leaf tissue in both high and low susceptibility genotypes from Ferrari et al. (2025)[8]; ii) differentially expressed between high and low susceptibility genotypes in uninfected leaf tissue identified in Franco Ortega (2025)[9] (which repeats the associative transcriptomics analysis using the same RNA-seq data as Sollars et al. (2017)[10] using a new reference genome); iii) differentially expressed between petiole tissue before and after infection, in both low and high susceptibility genotypes from Chano et al. (2025)[11], iv) differentially expressed between bark tissue distal and proximal to artificial inoculation, and in lesions between high and low susceptibility genotypes, and in bark tissue distal to infection between high and low susceptibility genotypes, from Sahraei et al. (2020)[12], v) genes homologous to those with evidence of convergent adaptation to ADB in the wider Fraxinus genus from Kelly et al. (2025)[13]. We demonstrated that genes identified in these studies overlapped with our candidate genes more often than would be expected by chance (Supplementary Figure 15; see below),

consistent with a role for these genes in low ADB susceptibility. While the overlap between these gene sets and the ones we identified is modest, we do not expect every gene within 10 kilobases of the 220 SNPs identified to be involved in conferring low susceptibility, nor for every differentially expressed gene to have an associated SNP within 10 kilobases.

In the revised version of the manuscript, we further discuss the potential functional significance of the genes we identified from our SNP-based GWAS, integrating these more deeply with these prior studies. As expected, we find genes associated with a variety of potential resistance pathways, from those potentially involved in phenology and pathogen avoidance, to detection of pathogens, signalling, and production of antifungal enzymes or chemicals, with many of these genes showing expression pattern differences across multiple studies. These are discussed extensively, and the full set of genes and their overlaps across these studies are detailed in Supplementary Table 7.

Results: Lines 402-426
"These 211 genes formed 206 clusters in OrthoFinder - those containing sequences identified in six other genomic or transcriptomic studies investigating low susceptibility to ADB were identified[42,82–85] (Supplementary Table 7, Supplementary Figure 14). Two clusters contained genes differentially expressed between uninfected leaf tissue of low vs. high susceptibility genotypes[50], four and twelve contained genes differentially expressed between infected and uninfected rachises of i) a low susceptibility genotype, and ii) a high susceptibility genotype, respectively[82]. 37 and 20 clusters contained genes differentially expressed between uninfected and infected petiole tissue in a i) resistant genotype, and ii) a susceptible genotype, respectively[83]. 19 clusters contained genes differentially expressed between infected lesions following artificial inoculation, and distant uninfected bark tissue; one contained genes differentially expressed between lesions in low vs. high susceptibility genotypes; 14 contained genes differentially expressed in distant uninfected bark tissue following artificial inoculation between low and high susceptibility genotypes[84]. None of the clusters contained *F. excelsior* homologues of genes displaying signs of convergent evolution to ADB in other Fraxinus species[85]. 21 of the clusters contained 22 of the 55 genes identified as near to significant SNPs in Stocks et al. (2019)[42]. Excluding those from Stocks et al. (which were identified with the same samples as this study, so do not represent an independent source of evidence), genes identified across all these studies were found in 3,655 gene clusters, of which 57 were among the set of 206 clusters containing our candidate. An overlap of 57/206 was higher than 99.2% of 10,000 sets of randomly selected groups of 3,655 genes (Supplementary Figure 15A). The 3,655 clusters with genes identified from other studies contained 584 dispensable genes in 382 clusters - of 10,000 randomly selected sets of 3,655 clusters, none contained as many dispensable genes (Supplementary Figure 15B)."

Discussion: Lines 546-599
"Whilst no GO terms were significantly enriched in the 211 genes near our candidate SNPs, 57/206 of orthogroup clusters containing these genes also contained genes matching those showing gene expression differences between healthy vs. infected tissue, or low vs. high susceptibility genotypes[50,82–84], an overlap greater than expected by chance (Supplementary

Table 7; Supplementary Figure 15). The 211 genes within 10kb of our candidate SNPs have a variety of potential roles in low ADB susceptibility. Several are homologues of *Arabidopsis* genes associated with changes in vernalisation, flowering or leaf senescence timing: VERNALISATION-INSENSITIVE 3-LIKE, FCS LIKE ZINC FINGER 6, and RETINOL DEHYDROGENASE[108–110]. Changes in phenology such as early leaf flushing and senescence have been implicated in low ADB susceptibility[111], potentially associated with avoidance of pathogen infection. Genes associated with pathogen detection pathways such as an NBS-LRR, a wall-associated kinase, five receptor-like kinases and chitin receptor, some of which showed differential expression in response to ADB infection, were near significant SNPs. Genes involved in the regulation of hormone signalling were detected - including a homologue of *Arabidopsis* Jasmonate-Induced Dioxygenase 1, which regulates jasmonic acid homeostasis in *Arabidopsis*[112] and is differentially expressed in early infection by *H. fraxineus* in rachises and petioles[82,84]. Multiple homologues of cytochrome P450s were detected, some of which were differentially expressed in leaves and/or lesions[82,84]. Cytochrome P450 genes were also near the highest ranked SNPs predicting low ADB damage in Doonan et al. (2025)[44]. Homologues of genes encoding aspartic peptidases and glucanases, which can degrade fungal cell walls[113,114], were identified - a homologue of the aspartic peptidase detected was differentially expressed in infected petioles of a low susceptibility genotype, but not the high susceptibility genotype[83]. Genes producing antifungal compounds were also detected - such as a homologue of glutamate decarboxylase, differentially regulated in lesions, which produces GABA, a compound with antifungal properties[115,116]. A homologue of *Arabidopsis* DIRIGENT PROTEIN 1 was identified - this is involved in the biosynthesis of lignans[117], compounds associated with low ash dieback susceptibility[111,118]. Pectate lyases and pectinase inhibitors may be involved in specific pathogen responses, such as cell wall remodelling, or preventing degradation by the fungus[119], or more indirectly in mediating phenological changes[44]. Doonan et al. identified a pectinesterase inhibitor as being associated with autumn leaf yellowing, but not low ADB crown damage[44]. The pectate lyase was differentially expressed in developing lesions in high but not low susceptibility genotypes[84], as well as between low susceptibility and high susceptibility tissue distant to the site of artificial inoculation[84], and between leaves of high and low susceptibility plants[50]. This suggests pectin metabolism may be involved in both avoidance of and active resistance to ADB. Only two gene clusters overlapped with genes differentially expressed between healthy low vs. high susceptibility individuals[50]. It could be that genetic differences in low ADB susceptibility are more strongly associated with induced than constitutive expression difference in leaves, or that constitutive differences are mediated by trans-acting regulatory processes – several significant SNPs in this study are near transcription factors (Supplementary Table 7). The only gene in this study homologous to those differentially expressed in lesions between low and high susceptibility genotypes was a dispensable RNA polymerase associated with transcriptional silencing; knockouts of the *Arabidopsis* homologue to this gene are more vulnerable to fungal infection[120]. No genes overlap with those identified as candidates for fungal defence response in *Fraxinus* lineages that co-evolved with the pathogen[85] - supporting the suggestion of that study that mechanisms underlying low susceptibility in *F. excelsior* may differ from those in the natural hosts of *H. fraxineus.* This study indicates a variety of mechanisms underpin low ADB susceptibility, increasing the chances that the underlying genetic variants may be additive in

their effects and suggesting natural selection or a breeding program may increase low ADB susceptibility. ”

Methods: Lines 938-955

“Ortholog clusters across assemblies were used to compare genes identified as associated with low ADB damage across studies. BATG-0.5 gene models were used for Stocks et al. (2019)[42] and Kelly et al. (2025)[85], and Fexc_ONT_UoY gene models for Franco Ortega et al. (2025)[50]. Chano et al. (2025)[83] and Ferrari et al. (2025)[82] mapped RNA-seq data to FRAX_001_PL and identified differentially expressed genes using an annotation of that genome that is not publicly available. Sahraei et al. (2020)[84] mapped reads to an earlier version of the BATG-0.5 assembly (BATG-0.4v3), providing fasta sequences of their differentially expressed genes (DEGs) - reciprocal best BLAST hits of these sequences and BATG-0.5 gene models was performed in Kelly et al. (2025)[85], with 81.6% of the DEG sequences having a reciprocal best BLAST hit in the BATG-0.5 annotation; the corresponding BATG-0.5 sequences were used as proxies for the sequences in Sahraei et al (2020). Supplementary Table 2 of Ferrari et al. (2025)[82] identified best hits of the *F. excelsior* gene models to those of other assemblies - 83.2% of these matched genes in the *O. europea, S. indicum, S. lycopersicum* and *E. guttatus* assemblies included in our analysis, including 90.8% of significant genes from Supplementary Tables 3 and 4. Chano et al. used the same annotation files as Ferrari et al., allowing homologues of 79.3% of their DEGs to be identified.”

Methods: Lines 1047-1068

“These genes formed 206 orthogroups (gene clusters) in the Orthofinder run from the pangenome annotation (see “Linear reference and pangenome annotation” above) - genes with sequence matches identified from the expression studies described previously were identified. To calculate the number of shared clusters expected by chance, clusters containing at least one sequence match for DEGs from Sahraei et al. (2020)[84], Ferrari et al. (2025)[82], Chano et al. (2025)[83], Franco-Ortega et al. (2025)[50] and Kelly et al. (2025)[85] were identified (3,655 clusters in total). For random resampling, for each of these clusters, the genome from which the sequence match was identified was recorded (i.e. Fexc_ONT_UoY for Franco-Ortega et al. (2025); BATG-0.5 for Kelly et al. (2025) etc.; see “Linear reference and pangenome annotation” above) - where a cluster contained sequence matches originating from more than one genome, BATG-0.5 was preferentially chosen; if the sequence matches were identified from different genomes, one of those genomes was chosen at random. This resulted in a total of 1,541 genes from BATG-0.5, 1,790 from *O. europaea,* 148 from Fexc_ONT_UoY, 139 from *S. indicum*, 31 from *E. guttatus*, and 6 from *S. lycopersicum* to be randomly resampled*.* For each genome, the respective number of genes was chosen at random (e.g. 1,541 genes from BATG-0.5), for a total of 3,655 genes - the clusters that gene each belonged to were identified, and the number of these clusters that also contained at least one of the 211 candidate genes from this study was identified. This was repeated 10,000 times. A separate set of 10,000 x 3,655 genes was generated to identify how many contained dispensable genes.”

Supplementary Information: Lines 523-561

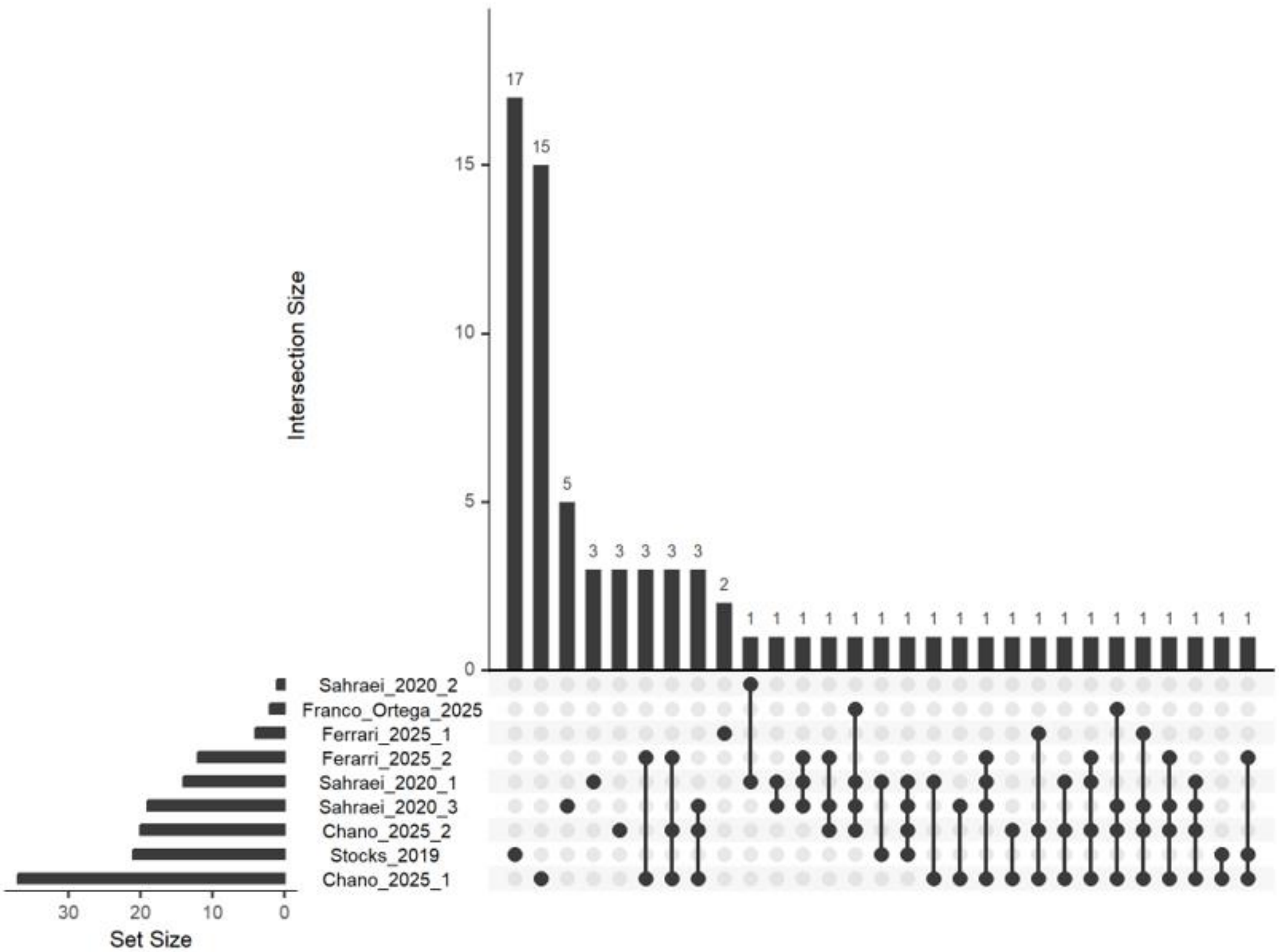

Supplementary Figure 14 – Overlapping gene clusters across datasets

Upset plot for subsets of genes from genomic/transcriptomic studies identifying genes associated with low ash dieback susceptibility, or the response to ash dieback infection, that are in the same 206 gene clusters containing the 211 candidate genes identified in this study. Set size panel corresponds to the total number of genes in each subset (i.e. the total overlap between genes identified in that study, and the current study). In the intersection size panel, single dots correspond to genes unique to that subset that intersect with genes in this study, lines connecting dots represent genes found in multiple subsets that intersect with genes in this study. Subsets are as follows: Overlap with genes identified from Chano et al. (2025); differentially expressed genes (DEGs) between infected and uninfected petiole tissue in i) a low susceptibility genotype (Chano_2025_1) and ii) a high susceptibility genotype (Chano_2025_2); Overlap with genes identified from Ferrari et al. (2025); DEGs between infected and uninfected petiole tissue in i) a low susceptibility genotype (Ferrari_2025_1) and ii) a high susceptibility genotype (Ferrari_2025_2); Overlap with DEGs from Franco-Ortega et al. (2025) between leaf tissue in low susceptibility and high susceptibility genotypes (Franco_Ortega_2025); Overlap with DEGs from Sahraei et al. (2020); i) expressed in distant uninfected bark tissue following

artificial inoculation between low and high susceptibility genotypes (Sahraei_2020_1), ii) between lesions in low vs. high susceptibility genotypes (Sahraei_2020_2), and iii) between infected lesions following artificial inoculation, and distant uninfected bark tissue (Sahraei_2020_3); Genes within 5 kilobases either up or downstream of a SNP identified in Stocks et al. (2019).

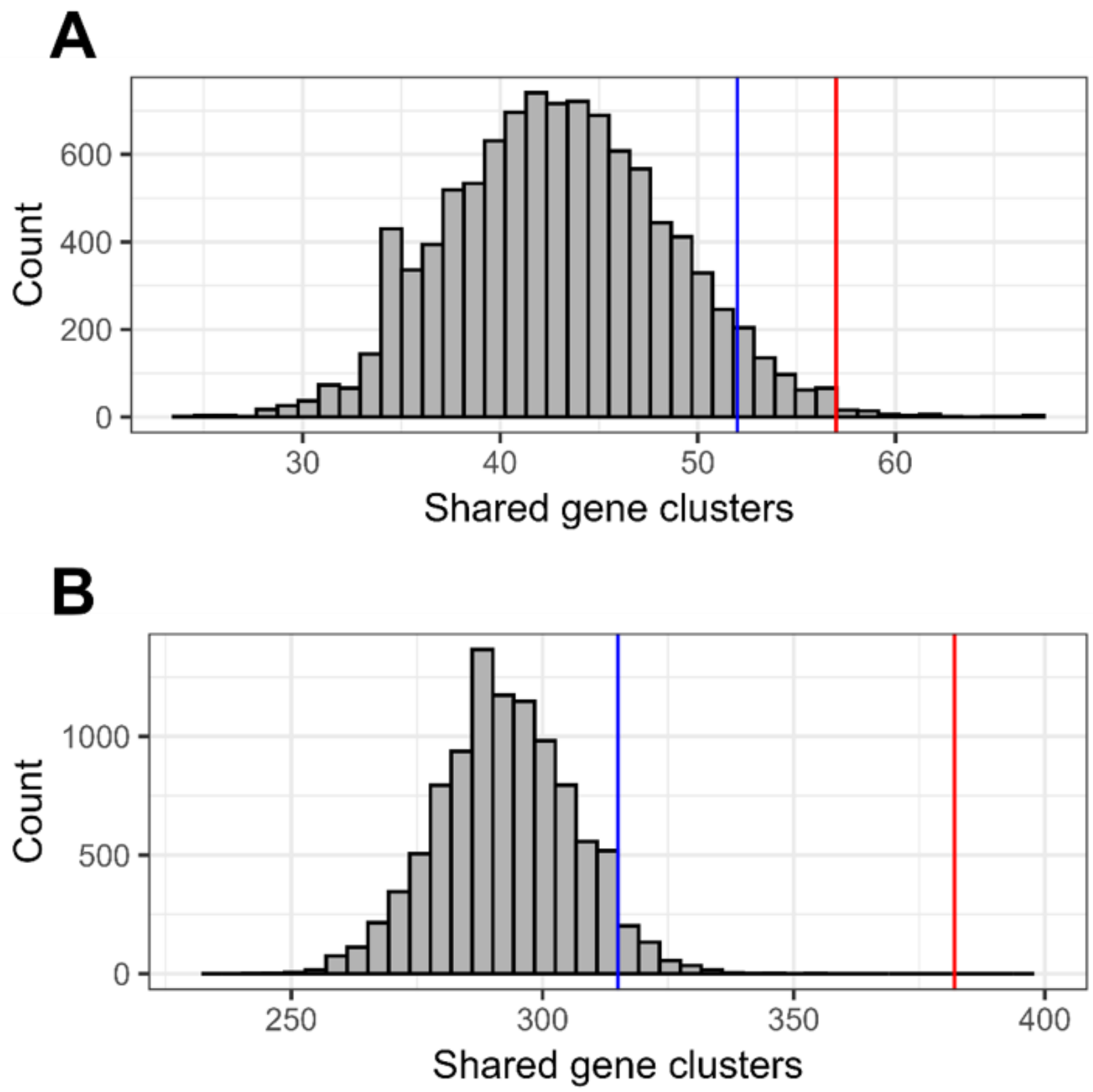

Supplementary Figure 15 – observed vs. expected overlaps in gene clusters.

3,655 gene clusters (Orthogroups) from the OrthoFinder run in this study contained sequence matches to genes associated with ash dieback from a number of studies (see Methods for details). A) 10,000 sets of 3,655 genes were randomly selected, and how many of the 206 clusters containing 211 genes within 10 kilobases of the 220 candidate single nucleotide polymorphisms identified in this study that overlap with these was counted, displayed in a histogram. Blue line represents 95th percentile, red line represents how many of the 206 clusters overlapped with the non-random 3,655 genes (n=57). B) 10,000 sets of 3,655 genes were randomly selected, and the frequency with which these occurred in clusters containing dispensable genes, plotted in a histogram. Blue line represents 95th percentile, red line represents how many of the non-random 3,655 gene clusters contained dispensable genes (n=382).”

R4.5

4. The pangenome resource is of high quality and has been constructed with appropriate methodological care. However, its contribution to biological insight remains somewhat limited. At present, the pangenome analysis primarily refines estimates (e.g., of dispensable gene content) and improves methodological rigor, but does not yet clearly demonstrate how it leads to new conceptual understanding of the biological system under investigation.

We investigated two biological systems in this study - 1) the pangenome of Fraxinus excelsior itself, and 2) the genetic component of low susceptibility to ash dieback, using the pangenome as a tool. We would argue that we have uncovered new conceptual understandings of both.

In investigating the F. excelsior pangenome, we identified variable sequences equalling 22% of the size of the linear reference genome, along with thousands dispensable genes, but also showed that this does not capture the full range of SVs, highlighting the enormous variability in genome content in this widespread forest tree species. We also demonstrate that whilst thousands of dispensable genes are detected, the numbers can potentially be enormously overestimated. As the reviewer noted in their comment R4.17 in the initial round of review, this is a crucial result - whilst gene content does vary between individuals, it may be by substantially less than suggested in other studies. Whilst pangenomes can be a useful tool for investigating other traits, we would argue that the pangenome itself is a biological system worthy of study, and that these are important conceptual advances.

As previously highlighted, we identified issues with estimating SV allele frequencies from poolseq data that prevented us from performing a GWAS based on SVs with the data from Stocks et al. 2019. We found that mismapping of reads from structural variants can lead to erroneous conclusions in SNP-based GWAS - improved read mapping to the pangenome compared to a linear reference genome did not entirely remove these errors, necessitating careful filtering. In the course of the study, we explicitly identified SNPs that shift in direction consistently across provenances, which has not been performed in previous studies, highlighting issues with the commonly used CMH test. Indeed, we believe a widely applicable insight from our study is that care must be taken to mitigate the impact of unaccounted for structural variants when mapping to a reference, even when that reference is a pangenome. We acknowledge that some of this analysis could have been performed using a linear reference genome, but we do not believe this detracts from the significance of the insight, nor that studies using pangenomes should limit themselves exclusively to investigations that are only possible using pangenomes. Nevertheless, these analyses are improved by using a pangenome reference - both by giving a more complete set of genes potentially linked to significant SNPs, and by improving the quality of short read mapping, reducing (but not eliminating) spurious mappings to the reference.

As discussed more extensively in responses to comments above, the main aim of this study was not to explicitly identify the mechanistic basis of ash dieback, nor how the dispensable gene content in F. excelsior relates to this. Nevertheless, we acknowledge that this is an interesting area we could have explored more. We had previously identified that 16 of the 211 genes within 10kb of significant SNPs associated with low ADB susceptibility were dispensable - deeper integration with other studies (detailed above) revealed that this dispensable gene set includes the only gene of the 211 that had evidence of being differentially expressed within lesions between low and high susceptibility genotypes from Sahraei et al. (2020)[12]. This gene is a dispensable RNA polymerase associated with transcriptional silencing; knockouts of the Arabidopsis homologue to this gene are more vulnerable to fungal infection[14].

To further investigate the potential impact of dispensable genes on mechanisms of low susceptibility to ash dieback more broadly (i.e. beyond those genes near a significant SNP in the GWAS), we investigated the overlap between dispensable genes and the total set of 3,655 gene clusters identified from previous studies as potentially involved in the response to ADB infection. Of these clusters, 382 contained dispensable genes, with 584 dispensable genes included in total (over 17% of all dispensable genes identified). This represents a greater overlap than any of 10,000 randomly selected groups of 3,655 gene clusters (Supplementary Figure 14B; see above). Whilst the vast majority of these genes are not near a significant SNP, this shows that many potential genes involved in the response to ash dieback may not be present in all individuals, highlighting the complexity of identifying a common mechanism of resistance in the light of variable gene content between individuals. We have highlighted this point in the text: (see quoted manuscript text for response to R4.4).

R4.6

In summary, I appreciate the substantial improvements made by the authors and recognize the value of the genomic resources presented. Nevertheless, additional work would be required to more convincingly establish the biological significance of the findings and to achieve the level of conceptual or mechanistic advance expected for this journal.

We are glad the reviewer appreciates the improvements made and recognises the value of the resource presented.

As emphasised in our response to R4.5, we believe our results have substantial biological and conceptual significance, in our understanding of pangenomes themselves, their use as tools for investigating the genetic basis of traits, and the genetic basis of low ash dieback susceptibility specifically. Identifying genetic variants consistently associated with low susceptibility to ash dieback is critical for understanding the potential action of natural selection on this trait (see Metheringham et al. (2025)[4]), and the effectiveness of breeding programs for low susceptibility.

As the reviewer suggested, we have performed deeper functional characterisation of candidate genes and integrated our results more fully with other more gene-focused studies of low ash dieback disease susceptibility, providing further evidence of several potentially promising pathways or genes to investigate and indicating that a variety of mechanisms likely underpin low ADB susceptibility, increasing the likelihood that variants underpinning these mechanisms could be additive in their effects. We do not believe further validating these in a non-correlative manner (i.e. using gene knockouts) would be practical or a good use of resources, as we argue above - but the improved characterisation of these genes in the revised manuscript provides potential targets for this work to be done by others, if they desired.

Whilst the extent of the mechanistic characterisation of polygenic traits required for publication in this journal is an editorial decision, we would note that recent studies in this journal that experimentally characterise candidate genes from GWAS are mostly in either crops or humans[15–17] which may have substantial corroborating datasets/experimental resources available, and/or are more straightforward to experimentally manipulate than wild tree species[18]. We would also note that studies relying on identifying genetic variants and genes of interest based on correlative analysis between genetic variants and traits, without necessarily performing non-correlative functional validation of candidate genes (via knockouts or equivalent), are published in this journal[18–22].

Given the numerous and growing challenges wild forest tree species face, we do not believe that limited resources available are best deployed constructing and experimentally validating detailed mechanistic models of low disease susceptibility in these species. Instead, we seek both to provide knowledge of the genetic basis of resistance that will enable acceleration of ash dieback breeding programmes, and to provide a framework to allow this approach to be applied in other species in response to the growing range of threats they face. Identifying genetic variants that are consistently associated with low ash dieback susceptibility is a significant advance towards this goal.

We appreciate the time the reviewer has taken to review our work a second time - we believe engaging with their comments in both rounds of review has led to substantial improvements to the manuscript.

References

1. Rabiey, M. *et al.* Scaling-up to understand tree-pathogen interactions: A steep, tough climb or a walk in the park? *Curr. Opin. Plant Biol.* **68**, 102229 (2022).
2. Schertler, E., Queloz, V., Monteleone, F., Eisenring, M. & Heinzelmann, R. Resistance and tolerance mechanisms of common ash (*Fraxinus excelsior*) against the ash dieback pathogen *Hymenoscyphus fraxineus*: latest advances and knowledge gaps. *Forestry (Lond.)* cpaf038 (2025).
3. Grattapaglia, D. & Resende, M. D. V. Genomic selection in forest tree breeding. *Tree Genet. Genomes* **7**, 241–255 (2011).
4. Metheringham, C. L. *et al.* Rapid polygenic adaptation in a wild population of ash trees under a novel fungal epidemic. *Science* **388**, 1422–1425 (2025).
5. Lobo, A. *et al.* Genomic prediction of superior heartwood traits in pedunculate oak (Quercus robur L.) using GWAS-informed Bayesian models. *For. Ecol. Manage.* **609**, 123644 (2026).
6. Kaler, A. S., Purcell, L. C., Beissinger, T. & Gillman, J. D. Genomic prediction models for traits differing in heritability for soybean, rice, and maize. *BMC Plant Biol.* **22**, 87 (2022).
7. Doonan, J. M. *et al.* Multiple, single trait GWAS and supervised machine learning reveal the genetic architecture of *Fraxinus excelsior* tolerance to ash dieback in Europe. *Plant Cell Environ.* **48**, 3793–3809 (2025).
8. Ferrari, R. C. *et al.* Transcriptional profiling of Fraxinus excelsior leaves during the early infection phase of ash dieback. *J. Plant Dis. Prot. (2006)* **132**, 1–17 (2025).
9. Franco Ortega, S. *et al. Fraxinus excelsior* updated long-read genome reveals the importance of MADS-box genes in tolerance mechanisms against ash dieback. *G3 (Bethesda)* **15**, 2024.12.20.629733 (2025).
10. Sollars, E. S. A. *et al.* Genome sequence and genetic diversity of European ash trees.

Nature **541**, 212–216 (2017).

11. Chano, V. *et al.* Transcriptional time-course analysis during ash dieback infection revealed different responses in tolerant and susceptible Fraxinus excelsior genotypes. *BMC Plant Biol.* **25**, 107 (2025).
12. Sahraei, S. E., Cleary, M., Stenlid, J., Brandström Durling, M. & Elfstrand, M. Transcriptional responses in developing lesions of European common ash (Fraxinus excelsior) reveal genes responding to infection by Hymenoscyphus fraxineus. *BMC Plant Biol.* **20**, 455 (2020).
13. Kelly, L., Coates, S., Lee, S., Rossiter, S. & Buggs, R. Molecular convergence analyses identify candidate genes for low susceptibility to the ash dieback pathogen. *bioRxiv* 2025.02.08.637240 (2025) doi:10.1101/2025.02.08.637240.
14. Ellendorff, U., Fradin, E. F., de Jonge, R. & Thomma, B. P. H. J. RNA silencing is required for Arabidopsis defence against Verticillium wilt disease. *J. Exp. Bot.* **60**, 591–602 (2009).
15. Timsina, J. *et al.* GWAS meta-analysis of cerebrospinal fluid Alzheimer's biomarkers reveals loci regulating lipids, brain volume and autophagy. *Nat. Commun.* 1–24 (2026).
16. Dai, H. *et al.* Genetic landscape and functional exploration of kidney cancer predisposition in cross-ancestral populations. *Nat. Commun.* 1–18 (2026).
17. Li, J. *et al.* Leveraging weighted embedding and Transformer architecture to improve phenotype prediction of complex traits for crops. *Nat. Commun.* **17**, 4427 (2026).
18. Hao, Z. *et al.* Genomic insights into the improvement of Chinese fir from ancient domestication continuum to modern breeding. *Nat. Commun.* 1–15 (2026).
19. You, Q. *et al.* Sub-pangenome analysis reveals structural variants associated with fruit color and bacterial wilt resistance in eggplant. *Nat. Commun.* **17**, 3075 (2026).
20. Ma, Z. *et al.* Population genomics reveals association of transposable elements variants with climatic adaptation in wild Amur grape. *Nat. Commun.* **17**, 3213 (2026).
21. Liu, X. *et al.* Going wild in banana breeding enables Fusarium-resistant hybrids with

improved fruit quality. *Nat. Commun.* **17**, 3524 (2026).

22. Huang, Y. *et al.* Digitaria genome analyses indicate introgression may drive local adaptation and herbicide resistance. *Nat. Commun.* **17**, 2669 (2026).

REVIEWERS' COMMENTS

Reviewer #4 (Remarks to the Author):

The authors have further improved the manuscript in response to the previous round of review. In particular, the revised manuscript now more clearly explains how structural variation, gene presence-absence variation, and SNP-based association signals relate to differences in ash dieback susceptibility. I also appreciate the clearer statement of the intended level of biological interpretation and the strengthened discussion of polygenic architecture.
However, while these changes improve clarity and help place the results in better context, I still view the manuscript as a collection of well-executed but relatively separate genomic analyses that are not yet fully integrated into a single, clear model of disease susceptibility. The relationships between structural variation, dispensable gene dynamics, and SNP-based signals are better described, but remain largely interpretative rather than forming a unified mechanistic or predictive framework for ash dieback resistance.
I agree that no additional experimental validation is needed, and I recognize that the study is not intended to be mechanistic. My main remaining concern therefore relates to the level of conceptual integration and whether the manuscript goes beyond a largely descriptive summary of genomic signals. Further strengthening of the conceptual synthesis would improve the manuscript, although I acknowledge the progress made in this revision.

We are glad the reviewer believes the manuscript is improved, that the main focus of the study is not mechanistic and that no further experimental validation is required. A unified mechanistic framework would be useful, but as outlined previously is not the focus of this research. The diverse potential functions of genes located near our significant SNPs support previous interpretations of ash dieback disease susceptibility as involving a variety of mechanisms, from altered phenology to specific biochemical defences. Whether these are integrated into what could be interpreted as a single mechanism, or separate mechanisms that together contribute to lower susceptibility overall (but may not offer a "unified mechanistic framework") is hard to say, although we suspect the latter. Further conceptual integration of the role of SNPs and SVs (including dispensable genes) would be interesting, but would require accurate identification of SVs associated with low susceptibility, which is not possible with current data/software. Further discussion of this we believe would be overly speculative at this stage, and we are also conscious that the manuscript has grown quite long through the process of addressing the previous reviews, so conclude that this is best left to future studies.

We agree a predictive framework would be useful - whilst we have identified SNPs and potential genes associated with low susceptibility in this study, we believe that fully incorporating SVs will be important in accurately predicting low disease susceptibility. We hope that our study will encourage the development of software capable of accurately estimating SV frequency from

poolseq data - future work may also generate large-scale sequencing data for individuals rather than pools for F. excelsior. This would allow SVs to be genotyped accurately along with SNPs using the pangenome presented here, so that a full genomic prediction model using both SNPs and SVs can be produced.

We thank the reviewer for their comments.

ROUND 2 REVIEWER 3 ATTACHMENT:

Answer to authors

The authors have addressed R2 and R3 comments thoroughly and with great accuracy. The revised manuscript reflects the requested clarifications, and we are satisfied with the responses provided. In our opinion, the manuscript is now suitable for publication. We would recommend a final careful proofreading to correct any remaining typos and stylistic inconsistencies.

R2.1 Thanks for all these efforts to expand the RNA-seq data – our comment was well-adressed; We believe these changes indeed strengthen the reliability of the results and conclusions of the manuscript.

R2.2 This point was well-adressed also in response to **R1.17**

R2.3 We find that more clarity on the comprison between Stocks et al analyses and the present manuscript was made in the new version (especially lines 351-405). The re-analyses of the new version led to substantial changes in the results and conclusions, which point to an improvement when using the pangenome instead of a linear reference and important implications for pipelines using pool-seq data for GWAS.

R2.4 was well-adressed.

R2.6 and **R1.2** were well-adressed and we agree with the authors that we still lack methodology to understand all the causes behind variation across assmeblies.

R2.8 we think this is indeed a better approach: keeping more robust SVs even if rare ones may go undetected.

R2.9 Thanks for this addition, the comment was addressed well.
