# Supplementary material for: European ash pangenome reveals widespread structural variation and genetic basis of low ash dieback susceptibility
Source: Nat Commun. 2026 Sep 17;17:9693. doi: 10.1038/s41467-026-77194-9 (PMC13586383; doi:10.1038/s41467-026-77194-9)
Supplement: Supplementary file 4 [file 41467_2026_77194_MOESM3_ESM.pdf]

Supplementary Data File captions

Supplementary Data File 1 - *Fraxinus excelsior* genome assembly statistics

Details of the assembly statistics for *Fraxinus excelsior* from the present and other studies, including both hapotypes from the phased linear reference genome in this study. Row names refer to i) the assembly name, ii) the source of the assembly, where applicable giving the database name and the identifier, iii) the study associated with each assembly, where applicable, iv) the size of the assembly in Mb, v) the number of contigs, vi) the N50 of the assembly in Mb (50% of the assembly is in fragments this size or larger), vii) the L90 for each assembly (90% of the sequence is present in this many contigs), ix) the number of Ns per 100 kilobases, x) the BUSCO completeness score from the eudicots_odb10 dataset, in the format "% of total complete BUSCOs identified [% single copy, % duplicated], % of fragmented BUSCOs, % missing", xi) the Long Terminal Repeat (LTR) Assembly Index (LAI) score, xii) the number of intact LTRs, and xiii) the number of intact LTRs per megabase.

Supplementary Data File 2 - Reference protein sequences downloaded for Orthofinder

Details the protein sequence data downloaded for species/assemblies used in Orthofinder[8] analysis to identify homology of BATG-1.0/pangenome sequences. Column headings refer to i) scientific name of species used ii) common name iii) database identifier, iv) the database location where the protein sequences were downloaded and v) the doi of the associated publication for each dataset.

Supplementary Data File 3 - Sample information for long-read sequencing.

Sample information for the 50 biological samples sequenced with Oxford Nanopore Technologies (ONT) long-reads and used to construct the pangenome. Column headings refer to i) sample name, ii) provenance name for the samples, iii) material source - the RAP (Realising Ash's Potential) Trial; Forest Research (FR) Mass Screening Trial; the FRAXIGEN Trial; wild collected samples, or the if the sample was from the reference genome plant at Royal Botanic Gardens, Kew, iv) the latitude and v) longitude of the origin of the sample - some of these coordinates are approximate, details of how these were obtained/derived are given in column vi) location information, vii) the collection date of the sample, viii) the number of read pairs for samples that had short-read RNA-seq data generated (NA = no data generated), ix) the mean DNA coverage from ONT dats (assuming a genome size of 840Mb) after read mapping to BATG-1.0 (see Methods). Where centroid coordinates of Native Seed Zone (NSZs) were used to approximate sample origins, shapefiles were downloaded from https://www.data.gov.uk/dataset/cea32716-6efc-4a9e-83f7-b3fc1c0a07f7/forest-reproductive-materials-regions-of-provenance1, which Contains Forestry Commission information licensed under the Open Government Licence v3.0. © Crown copyright 2016.

Supplementary Data File 4 – dispensable gene GO enrichment vs. pangenome genes

Gene Ontology (GO) enrichment of genes identified as dispensable after filtering, against a background of all genes included in the filtered pangenome (dispensable plus invariant). Details of the results of GO term enrichment analysis of gene sets relative to a background set, using topGO[19]. Column headings refer to i) GO identifier, ii) GO term description, iii) Background - the number of genes in the background set annotated with this GO term, iv)

Observed - the number of genes in the set of interest with this annotation, v) Expected - the number of genes expected in the set of interest assuming no enrichment, vi) p-value - the unadjusted p-value of the classic Fisher test (one-sided) for enrichment, vii) Bonferonni adjusted p-value - the p-value adjusted using Bonferroni correction.

Supplementary Data File 5 – excluded putatively dispensable gene GO enrichment vs. pangenome genes

Gene Ontology (GO) enrichment of putatively dispensable genes excluded during filtering for not being consistently associated with an SV, against a background of all the genes included and excluded. Details of the results of GO term enrichment analysis of gene sets relative to a background set, using topGO[19]. Column headings refer to i) GO identifier, ii) GO term description, iii) Background - the number of genes in the background set annotated with this GO term, iv) Observed - the number of genes in the set of interest with this annotation, v) Expected - the number of genes expected in the set of interest assuming no enrichment, vi) p-value - the unadjusted p-value of the classic Fisher test (one-sided) for enrichment, vii) Bonferonni adjusted p-value - the p-value adjusted using Bonferroni correction.

Supplementary Data File 6 – Functional annotation of dispensable genes with “defense response” Gene Ontology term

Functional annotation of high-confidence dispensable genes (an SV-Gene F1 association score > 0.8; see Results) annotated with a “defense response” Gene Ontology (GO) term or a child term of this GO term (GO:0006952). Columns indicate i) gene name, ii) the bitscore and iv) E-value of the top hit in the UniProt/SwissProt database for each gene, v) the bitscore and vi) E-value of the top hit in the NCBI NR database for each gene, vi) the

PANTHER superfamily classification code and vii) description for each gene, vii) the InterPro family classification code and viii) description for each gene, ix) the bitscore and x) E-value of the best hit from a DIAMOND blastp search of the genes against the Araport 11 representative gene peptides, and xi) the name of the best hit.

Supplementary Data File 7 – Functional annotation of genes overlapping single nucleotide polymorphisms associated with low ash dieback disease susceptibility.

Functional annotation of genes that contained one or more of the 220 single nucleotide polymorphisms associated with low ash dieback disease susceptibility. Columns indicate i) gene name, ii) the bitscore and iv) E-value of the top hit in the UniProt/SwissProt database for each gene, v) the bitscore and vi) E-value of the top hit in the NCBI NR database for each gene, vi) the PANTHER superfamily classification code and vii) description for each gene, vii) the InterPro family classification code and viii) description for each gene, ix) the bitscore and x) E-value of the best hit from a DIAMOND blastp search of the genes against the Araport 11 representative gene peptides, and xi) the name of the best hit.

Supplementary Data File 8 – Functional annotation of genes within 10kb of single nucleotide polymorphisms associated with low ash dieback disease susceptibility.

Functional annotation of genes within 10kb of the 220 single nucleotide polymorphisms associated with low ash dieback disease susceptibility. Columns indicate i) gene name, ii) the bitscore and iv) E-value of the top hit in the UniProt/SwissProt database for each gene, v) the bitscore and vi) E-value of the top hit in the NCBI NR database for each gene, vi) the PANTHER superfamily classification code and vii) description for each gene, vii) the InterPro family classification code and viii) description for each gene, ix) the bitscore and x) E-value

of the best hit from a DIAMOND blastp search of the genes against the Araport 11 representative gene peptides, and xi) the name of the best hit.

Supplementary Data File 9 – genes near low ash dieback susceptibility-associated SNPs Gene Ontology enrichment

Gene Ontology (GO) enrichment of genes within 10kb of the 220 SNPs associated with low ADB susceptibility, against a background of all genes included in the pangenome. Details of the results of GO term enrichment analysis of gene sets relative to a background set, using topGO[19]. Column headings refer to i) GO identifier, ii) GO term description, iii) Background - the number of genes in the background set annotated with this GO term, iv) Observed - the number of genes in the set of interest with this annotation, v) Expected - the number of genes expected in the set of interest assuming no enrichment, vi) p-value - the unadjusted p-value of the classic Fisher test (one-sided) for enrichment, vii) Bonferonni adjusted p-value - the p-value adjusted using Bonferroni correction.

Supplementary Data File 10 – genes overlapping low ash dieback susceptibility-associated SNPs Gene Ontology enrichment

Gene Ontology (GO) enrichment of genes overlapping the 220 SNPs associated with low ADB susceptibility, against a background of all genes included in the pangenome. Details of the results of GO term enrichment analysis of gene sets relative to a background set, using topGO[19]. Column headings refer to i) GO identifier, ii) GO term description, iii) Background - the number of genes in the background set annotated with this GO term, iv) Observed - the number of genes in the set of interest with this annotation, v) Expected - the number of genes expected in the set of interest assuming no enrichment, vi) p-value - the unadjusted p-

value of the classic Fisher test (one-sided) for enrichment, vii) Bonferonni adjusted p-value - the p-value adjusted using Bonferroni correction.

Supplementary Data File 11 – Expression patterns of homologs of candidate genes in other studies

Excel table outlining for each of the 211 genes within 10 kilobases of the 220 single nucleotide polymorphisms identified as being consistently associated with low ash dieback susceptibility, expression patterns of homologs of each gene across other studies. Columns correspond to A) the gene name from this study of each of the 211 genes, B) the gene description from Interpro, C) the gene description from PANTHER, D_, the best blast match from searching the protein sequences against the Araport 11 representative gene peptides. D) KEGG pathway annotation, E) Gene cluster (i.e.Orthogroup) from running OrthoFinder in this study. Columns G-O identify whether a homolog of that gene was differentially expressed (Y) or not (N) across a number of comparisons within/across studies. Genes that could not be assigned to an ortholog group have blank values for these columns. Differential gene expression conditions for each of columns G-O are identified by the first 6 rows for these columns, detailing i) the genotype (L = low susceptibility, H = high susceptibility),ii) the tissue sequenced, iii) whether the individuals were infected (I) or uninfected (U) with ash dieback, iv) the study where the expression information was sourced, v) the source within the study where the particular gene list was found, and vi) any notes about how subsets of genes were extracted from tables where applicable. Differential expression comparisons are identified by a “v”; e.g. “L v H”, “Leaf”, “U”, refers to genes differentially expressed in high vs. low susceptibility individuals in uninfected leaf tissue. The final column (Q) contains information on potential roles in defense response for individual genes.

Supplementary Data File 12 - Comparison of key statistics with other recent pangenome studies.

Results from the present study are compared with those from plant pangenome studies published since 2022 (non-exhaustive) - pangenome studies using primarily short-read data, or incorporating multiple species, were excluded. Columns give i) the name of the study used in the labels for Supplementary Figure 24, comprising the common organism name and publication year, ii) the doi of the study, iii) the first author, iv) the publication date, v) the study organism, vi) N_samples, the number of samples used, vii) N_SVs, the number of SVs detected, where provided, viii) Extra sequence (Mb), the additional sequence in megabases incorporated as part of the pangenome, where provided, ix) the linear reference genome size, where provided, x) Extra/Ref, the proportion of additional sequence as a function of the reference genome size, xi) the number of core (non-dispensable) genes reported, xii) the total number of genes reported, xiii), the number of dispensable genes (non-core) as a proportion of the total number of genes. Columns vi) vs. vii), vi) vs.x) and vi) vs. xiii) are plotted in Supplementary Figure 24 panels A, B and C respectively. Ash_2026 (unfiltered) refers to the proportion of dispensable genes prior to filtering in the present study, Ash_2026 (filtered) refers to the proportion after filtering for consistent association with SVs - see Results for further details.

Supplementary Data File 13 – RNA-seq data used to annotate BATG-1.0 assembly

Details of RNA-seq data used to annotate linear reference assembly BATG-1.0. Columns represent i) the NCBI/ENA accession data was downloaded from, where applicable, ii) the RNA sequence length (long or short reads), iii) the study associated with the data, where applicable, iv) the genotype of the individual, v) the tissue type, vi) experimental treatment (where applicable) and vii) notes.

Supplementary Data File 14 – high confidence dispensable gene GO enrichment vs. all putatively dispensable genes

Gene Ontology (GO) enrichment of high confidence dispensable genes, against a background of the high confidence dispensable genes plus the putatively dispensable genes excluded during filtering for not being consistently associated with an SV. Details of the results of GO term enrichment analysis of gene sets relative to a background set, using topGO[19]. Column headings refer to i) GO identifier, ii) GO term description, iii) Background - the number of genes in the background set annotated with this GO term, iv) Observed - the number of genes in the set of interest with this annotation, v) Expected - the number of genes expected in the set of interest assuming no enrichment, vi) p-value - the unadjusted p-value of the classic Fisher test (one-sided) for enrichment, vii) Bonferonni adjusted p-value - the p-value adjusted using Bonferroni correction.
